# Maternal prenatal stress exposure and sex-specific risk of severe infection in offspring

**DOI:** 10.1371/journal.pone.0245747

**Published:** 2021-01-29

**Authors:** Monique Robinson, Kim W. Carter, Craig E. Pennell, Peter Jacoby, Hannah C. Moore, Stephen R. Zubrick, David Burgner

**Affiliations:** 1 Telethon Kids Institute, The University of Western Australia, Perth, Australia; 2 Centre for Child Health Research, The University of Western Australia, Perth, Australia; 3 School of Medicine and Public Health, The University of Newcastle, Newcastle, Australia; 4 Wesfarmers Centre for Vaccines and Infectious Diseases, Telethon Kids Institute, University of Western Australia, Perth, Australia; 5 Murdoch Children’s Research Institute, Parkville, Australia; 6 Department of Paediatrics, University of Melbourne, Parkville, Australia; 7 Department of Paediatrics, Monash University, Clayton, Australia; Aarhus University, DENMARK

## Abstract

**Background:**

Maternal stressful life events during pregnancy have been associated with immune dysregulation and increased risk for asthma and atopy in offspring. Few studies have investigated whether prenatal stress is associated with increased overall or specific infectious diseases in childhood, nor explored sex differences. We sought to examine the relationship between the nature and timing of maternal stress in pregnancy and hospitalisation with infection in offspring.

**Methods:**

Between 1989 and 1992, exposure data on stressful life events were collected from pregnant women (Gen1) in the Raine Study at 18 and 34 weeks’ gestation and linked to statutory state-wide hospital morbidity data. We examined associations between the number, category and timing of maternal prenatal stress events and overall and clinical groups of offspring (Gen2) infection-related hospitalisation until age 16 years, adjusting for maternal age, education, and smoking in pregnancy in addition to the presence of siblings at birth.

**Results:**

Of 2,141 offspring with complete stress in pregnancy data available, 1,089 had at least one infection-related hospitalisation, with upper respiratory tract infections the most common (n = 556). Each additional stressful life event during pregnancy was associated with increased risk in male offspring for hospitalisation with all infection types. There was little evidence of these associations in girls.

**Conclusions:**

Increased exposure to stressful life events *in utero* is associated with sex-specific infection-related hospitalisations in childhood. Prenatal stress may adversely affect early immune development for boys and increase the risk of more severe infections. Mechanistic understanding would inform preventative interventions.

## Introduction

Stressful life events during early childhood are associated with increased risk of adult non-communicable diseases, including cardiovascular disease, type 2 diabetes and cancer [1,2]. The impact of maternal stress during pregnancy on the risk for disease in the offspring is less well demonstrated. Animal and human data suggest that prenatal stress may have pervasive effects on offspring immune function [3]. Human studies of immune-related clinical outcomes predominantly focus on the increased risk of asthma and atopy [4–6], with evidence of sex-specific effects [7].

Prenatal maternal stress is correlated with specific early life infections in offspring [8–11], but longitudinal data through childhood and adolescence are scarce. Maternal bereavement during pregnancy is associated with increased overall and infection-specific mortality in offspring into middle age [12]. In the Danish National Birth Cohort, significant maternal stress during pregnancy, particularly that leading to anxiety and depression, is associated with diverse adverse outcomes in childhood, including infectious diseases [13]. Children whose mothers experienced at least one major stress event (death of a family member or divorce) either during or immediately prior to pregnancy have increased risk for infection-related hospitalisation [14]. Sex differences in the incidence and severity of childhood infection are well-reported and partly reflect immune ontogeny [15,16]. It is further suggested that male and female fetal immune development may be differently impacted by intrauterine insults including prenatal stress [17], with boys more vulnerable to prenatal stress, and girls more affected by cumulative stress across the pre- and postnatal periods [17].

In the current study we use a well-phenotyped prospective prenatal cohort linked to statutory population data to investigate the relationship between the timing and nature of maternal stress exposure during pregnancy and the overall and type-specific infection-related hospitalizations until the age of 16 years.

## Methods

### Cohort selection

The Raine Study was initially established to investigate possible effects of repeated ultrasound imaging in pregnancy [18]. 2900 pregnancies at mean gestational age of 18 weeks were recruited at the sole state tertiary perinatal centre (King Edward Memorial Hospital, Perth, Western Australia) from May 1989 through November 1991. As a state tertiary obstetric facility providing government-sponsored healthcare, the hospital population is disadvantaged relative to the general Western Australian population [19]. Mothers (Gen1) provided detailed demographic and psychosocial data at enrolment and at 34 weeks gestation. Informed written consent was obtained from the mother at enrolment and at each follow-up, including consent to link to statutory data from the Department of Health (Western Australia) health records [20]. The study was approved by The University of Western Australia Human Research Ethics Committee (RA/4/20/5722).

### Prenatal stress

Mothers from the Raine Study were asked at 18 weeks and 34 weeks gestation whether they had experienced any of ten major life stress events (pregnancy problems, death of a close friend or relative, separation or divorce, marital problems, problems with children, job loss (involuntary), partner’s job loss (involuntary), money problems, residential move, or other stressful event) selected from the Tennant and Andrews life stress inventory [21,22]. At 18 weeks gestation, mothers were asked if they had experienced any of the events since becoming pregnant and, at 34 weeks gestation, whether any of the stress events had been experienced in the previous four months. As with previous studies, responses were recorded as “yes” or “no” to maximise effective recall [23]. We calculated a total stressful life events index score throughout pregnancy from the sum of listed events, giving equal weight to each. Our previous work has assessed this method of recording stress events against a weighted scale and found it retains validity [24]. We also calculated the sum of stress exposures reported at 18 weeks’ gestation only, 34 weeks’ gestation only and both 18 and 34 weeks’ gestation. Given one of the largest existing studies examined the type of stress event [14], we also analysed the type of stress event by combining the ten events into six categories: death of a relative or friend (combined), financial problems (combining job loss, partner’s job loss and money problems), relationship problems (combining marital problems, and separation and divorce), residential move, pregnancy problems, and problems related to other children in the family. We calculated the sum of stress exposures reported within each category throughout pregnancy.

### Infection-related hospitalisations

The Western Australian Data Linkage System (WADLS) comprises systematic record linkage of multiple health administrative datasets through probabilistic linkage methods, including hospital morbidity data on all hospital admissions and separations throughout Western Australia [25]. Data pertaining to hospital admissions from children in the Raine Study (Gen2) were extracted and probabilistically linked to Raine cohort study data by WADLS staff. We extracted data on the International Classification of Diseases (ICD, versions 9 and 10 [26])-coded discharge diagnoses for all public and private hospital admissions in Raine Study participants up to 16 years of age. Offspring were classified as having an infection-related hospitalisation if they had an inpatient hospital admission with at least one primary or secondary infectious disease discharge code, more than one day after the birth-related discharge date. The first recorded day of contact with the hospital when patients were hospitalized was defined as the day of onset. Readmissions to hospital for infection within one week were considered as single admissions for the same cause. ICD infection codes were classified *a priori* into six clinical categories: viral, upper respiratory tract (URTI), lower respiratory tract (LRTI), gastrointestinal (GI), skin and soft tissue, and genitourinary infections, and the full list of ICD-9 and ICD-10 AM codes recorded is published elsewhere [27]. We calculated the total number of infection-related hospitalisations and the number of admissions for each infection category and created a binary variable reflecting ever having been admitted for any and for each infectious disease category. Due to lower sample sizes, we combined genitourinary infections, skin and soft tissue infections and other viral infections into one category of ‘other’. To investigate whether the association was more evident in pre-school offspring, which is the most common period of infectious disease hospital admission, we also analysed infection-related hospitalisations in the first 5 years of life.

### Statistical analysis

We analysed the associations between the number, type and timing of prenatal stress events reported by the mother and the association with the likelihood of any childhood (age 0–16) infection-related hospitalisation overall, and for each clinical infection category by binary logistic regression. We first examined male and female offspring together and then stratified the analyses by sex. The models were adjusted for mother’s age at pregnancy (years), mother’s level of education (high school completion), and maternal smoking at 18 weeks’ gestation (any). We did not adjust for family income or the presence of siblings, as financial problems and problems with other children were listed in our stress event scale. We then repeated these models examining the specific types of stress and for outcomes in only the first five years of life. IBM SPSS 24 was used for the analyses.

## Results

**Fig 1:**
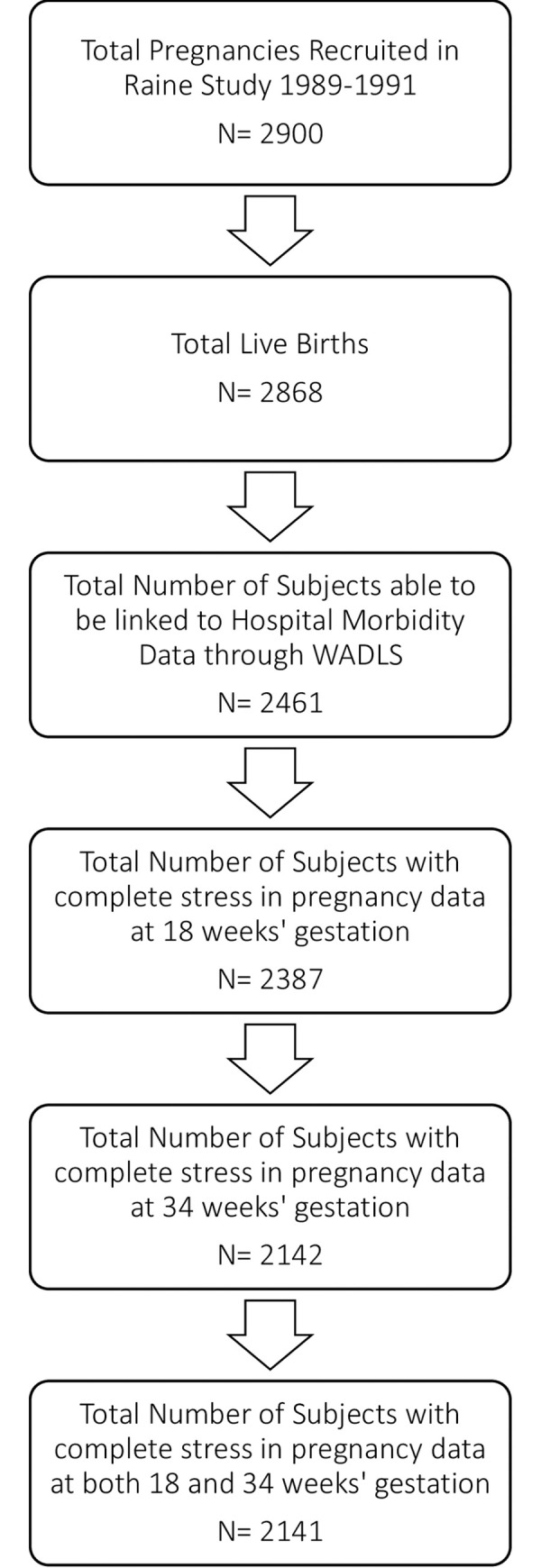
The Western Australian Pregnancy Cohort (Raine) study participation flow diagram describing total subjects with linked data and total stress in pregnancy data available.

Linked data were available for 2,461 offspring (**Fig 1**), and 2,141 of those had full stress in pregnancy data available (1121, 52.4% male; **Table 1**). European-Caucasian was the most common ethnicity reported (1905, 89.0%). Of the 2,141 offspring with full stress event data available, 1,089 offspring had at least one infection-related hospitalisation before the age of 16 years. Upper respiratory tract infections (URTI) were the most common infection-related hospitalisation category (556 offspring had ≥1 hospitalisations), and gastrointestinal infections were the least common (180 offspring had ≥1 hospitalisations). Financial and pregnancy related problems were the most frequently reported stress exposures (**S1 Table**). The prevalence of different types of stress reported at 18 weeks and 34 weeks gestation did not vary substantially and the majority of participants experienced between 0 and 5 stressful life events during pregnancy (**S2 Table**).

**Table 1:** Frequency data for study variables, stress events and infection-related hospitalisations by sex (N = 2141).

		Boys		Girls	
	N	Mean	SD	Mean	SD
**Maternal age at conception (years)**	2138	27.4	5.87	27.4	6.05
**Number of stress events in pregnancy**	2141	2.3	2.09	2.3	2.06
		**N**	**%**	**N**	**%**
**Timing of stress events (>1 event)**					
No stress events	468	247	22.0	221	21.7
<18 weeks only	400	207	18.5	193	18.9
18–34 weeks only	286	143	12.8	143	14.0
Both 18 and 34 weeks	987	524	46.7	463	45.4
**Maternal Education**					
<High school completion	1310	671	59.9	639	63.0
High school completion	825	450	40.1	375	37.0
**Maternal smoking in pregnancy**					
No	1582	838	74.8	744	72.9
Yes	559	283	25.2	276	27.1
	**N**	**N**	**%**	**N**	**%**
**Any infection related hospitalisation**	1089	546	48.7	543	53.2
**Lower Respiratory Tract Infections**	208	112	10.0	96	9.4
**Upper Respiratory Tract Infections**	556	275	24.5	281	27.5
**Gastrointestinal Infections**	180	96	8.6	84	8.2
**Other Infections (incl.genitourinary, skin and soft tissue and other viral infections)**	241	112	10.0	129	12.6

*Missing data not included.

In the adjusted logistic regression model, greater stress exposure during pregnancy was associated with an increased likelihood for gastrointestinal (OR 1.09, 95% CI 1.02, 1.17) and other infection-related hospitalisation in offspring when examining male and female children together (OR 1.09, 95% CI 1.03, 1.16; **Table 2**). When we stratified the cohort by sex, there was consistent evidence for significant risk of hospitalisation with infection in male offspring overall (OR 1.08, 95% CI 1.02, 1.14) and for each of our clinical infection categories; there was no evidence that these risks were increased in female offspring.

**Table 2:** Adjusted binary logistic regression analyses showing the relationship between total number of life stress events and risk for infection-related hospitalisations in childhood (N = 2,141, age 0–16).

	Adjusted odds ratio for any hospital admission (95% confidence interval)				
	Any infection	LRTI	URTI	GI	Other
**Life stress events**					
**Both (n = 2141)**	1.04 (0.99, 1.08)	1.05 (0.98, 1.12)	1.03 (0.98, 1.08)	1.09 (1.02, 1.17)	1.09 (1.03, 1.16)
**Male (n = 1121)**	1.08 (1.02, 1.14)	1.12 (1.03, 1.22)	1.07 (1.00, 1.14)	1.12 (1.02, 1.23)	1.17 (1.08, 1.28)
**Female (n = 1020)**	0.99 (0.93, 1.05)	0.96 (0.87, 1.07)	0.99 (0.92, 1.06)	1.05 (0.95, 1.17)	1.01 (0.92, 1.10)
**Timing of stress events** ≤ 18 weeks’ gestation only					
**Both**	0.97 (0.74, 1.28)	0.66 (0.40, 1.08)	1.03 (0.76, 1.40)	1.10 (0.65, 1.86)	0.95 (0.60, 1.49)
**Male**	1.16 (0.79, 1.69)	0.89 (0.44, 1.76)	1.06 (0.68, 1.65)	1.45 (0.66, 3.18)	1.23 (0.59, 2.56)
**Female**	0.80 (0.54, 1.18)	0.48 (0.24, 0.98)	0.99 (0.64, 1.52)	0.88 (0.43, 1.78)	0.81 (0.45, 1.44)
18–34 weeks’ gestation only					
**Both**	1.02 (0.76, 1.37)	1.09 (0.67, 1.76)	0.90 (0.63, 1.27)	1.63 (0.96, 2.76)	0.86 (0.51, 1.43)
**Male**	1.29 (0.85, 1.96)	1.42 (0.71, 2.83)	0.94 (0.56, 1.55)	2.02 (0.90, 4.53)	1.36 (0.61, 3.01)
**Female**	0.77 (0.50, 1.18)	0.81 (0.41, 1.62)	0.84 (0.52, 1.36)	1.33 (0.66, 2.68)	0.61 (0.31, 1.21)
Both 18 and 34 weeks’ gestation					
**Both**	1.10 (0.88, 1.38)	0.97 (0.66, 1.40)	1.03 (0.80, 1.33)	1.29 (0.84, 1.98)	1.26 (0.88, 1.81)
**Male**	1.54 (1.13, 2.11)	1.25 (0.73, 2.15)	1.20 (0.83, 1.73)	2.04 (1.06, 3.92)	2.11 (1.17, 3.82)
**Female**	0.75 (0.54, 1.05)	0.73 (0.43, 1.22)	0.88 (0.61, 1.26)	0.79 (0.44, 1.43)	0.84 (0.52, 1.35)

^†^odds ratio and 95% confidence intervals. Model adjusted for maternal age at pregnancy, maternal education at pregnancy and maternal smoking in pregnancy. LRTI: Lower respiratory tract infection; URTI: Upper respiratory tract infection; GI: Gastrointestinal infection; Other: Genitourinary infection, skin and soft tissue infection or other viral infection.

### Timing of pregnancy stress exposure

In the analyses of all children, stress experienced before 18 weeks gestation pregnancy only or between 18 and 34 weeks’ gestation only did not show significant associations with infection-related hospitalization (**Table 2**). Stress exposure reported at both time points was associated with any infection-related hospitalization (OR = 1.54, 95%CI = 1.13, 2.11), gastrointestinal infection (OR = 2.04, 95%CI = 1.06, 3.92) and other infection (OR = 2.11, 95%CI = 1.17, 3.82) for boys only.

For all offspring combined, when the stressful events experienced were separated by type of event (reported at any time in pregnancy; **Table 3**), death of a friend or relative showed a significant association with a higher likelihood of gastrointestinal (OR = 1.64, 95%CI = 1.11, 2.41) admissions. Pregnancy problems (OR = 1.25, 95%CI = 1.06, 1.49) were associated with an increased risk for overall infection-related hospitalisations in offspring and other admissions (OR = 1.41, 95%CI = 1.08, 1.83) and relationship problems were associated with gastrointestinal infection hospitalization (OR = 1.55, 95%CI = 1.08, 2.22). Financial problems and residential moves during pregnancy were not significantly associated with infectious disease hospitalisations when all offspring were considered together. Mothers who reported feeling stressed due to problems with other children had offspring with significantly higher rates of hospital admissions for all infectious diseases categories. Again, in the analyses stratified by sex, we observed significant relationships for the male offspring almost exclusively. In boys, the death of a friend or relative during pregnancy was associated with hospitalisation with GI (OR = 1.73, 95%CI = 1.04, 2.89) and financial problems were linked to LRTI (OR = 1.50, 95%CI = 1.01, 2.22), GI (OR = 1.53, 95%CI = 1.00, 2.35) and other admissions (OR = 1.73, 95%CI = 1.16, 2.58). Stress caused by problems with the pregnancy were significantly associated with overall infectious disease admissions (OR = 1.50, 95%CI = 1.18, 1.91) and URTI (OR = 1.47, 95%CI = 1.12, 1.92) and other (OR = 1.55, 95%CI = 1.06, 2.28) admissions in particular. Relationship problems were also associated with hospitalisation with other infection only (OR = 2.02, 95%CI = 1.30, 3.15), while stress due to other children in the family was associated with hospitalisation with any infectious disease, LRTI, URTI, GI and other infections. For girls, maternal stress due to a residential move appeared protective against any infection-related hospitalisation for female children (OR = 0.75, 95%CI = 0.57, 0.99).

**Table 3:** Adjusted binary logistic regression analyses showing the relationship between each additional life stress event during pregnancy by type of event and risk for infection-related hospitalisations during childhood (N = 2,141, age 0–16).

	All infection-related hospitalisations OR (95% CI)^†^	LRTI OR (95% CI)^†^	URTI OR (95% CI)^†^	GI OR (95% CI)^†^	Other OR (95% CI)^†^
**All offspring (n = 2141)**					
Death of a friend/relative	1.04 (0.81, 1.33)	0.92 (0.61, 1.41)	1.12 (0.85, 1.47)	1.64 (1.11, 2.41)	1.30 (0.90, 1.87)
Financial problems	1.13 (0.95, 1.34)	1.15 (0.87, 1.53)	1.01 (0.83, 1.23)	1.25 (0.92, 1.70)	1.23 (0.94, 1.61)
Pregnancy problems	1.25 (1.06, 1.49)	1.17 (0.88, 1.55)	1.20 (0.99, 1.46)	1.21 (0.90, 1.63)	1.41 (1.08, 1.83)
Relationship problems	1.07 (0.85, 1.34)	1.31 (0.93, 1.85)	1.01 (0.78, 1.30)	1.55 (1.08, 2.22)	1.34 (0.97, 1.86)
Residential move	0.89 (0.74, 1.08)	0.89 (0.65, 1.22)	0.84 (0.68, 1.05)	1.16 (0.84, 1.62)	1.08 (0.81, 1.44)
Problems with other children	1.78 (1.35, 2.36)	1.68 (1.13, 2.49)	1.71 (1.28, 2.27)	1.69 (1.11, 2.59)	1.84 (1.24, 2.68)
**Male offspring (n = 1121)**					
Death of a friend/relative	1.11 (0.79, 1.55)	1.00 (0.58, 1.73)	1.33 (0.92, 1.92)	1.73 (1.04, 2.89)	1.27 (0.77, 2.12)
Financial problems	1.25 (0.98, 1.59)	1.50 (1.01, 2.22)	1.05 (0.80, 1.39)	1.53 (1.00, 2.35)	1.73 (1.16, 2.58)
Pregnancy problems	1.50 (1.18, 1.91)	1.43 (0.98, 2.11)	1.47 (1.12, 1.92)	1.32 (0.87, 2.00)	1.55 (1.06, 2.28)
Relationship problems	1.32 (0.97, 1.81)	1.38 (0.86, 2.20)	1.24 (0.88, 1.75)	1.46 (0.89, 2.40)	2.02 (1.30, 3.15)
Residential move	1.04 (0.80, 1.35)	1.17 (0.77, 1.79)	0.88 (0.64, 1.19)	1.27 (0.81, 2.00)	1.42 (0.94, 2.14)
Problems with other children	2.21 (1.49, 3.26)	2.51 (1.52, 4.16)	1.93 (1.30, 2.86)	1.94 (1.09, 3.44)	2.76 (1.66, 4.59)
**Female offspring (n = 1020)**					
Death of a friend/relative	0.97 (0.67, 1.41)	0.82 (0.42, 1.58)	0.92 (0.61, 1.40)	1.48 (0.82, 2.66)	1.31 (0.78, 2.21)
Financial problems	1.01 (0.79, 1.30)	0.85 (0.56, 1.29)	0.97 (0.73, 1.28)	0.99 (0.63, 1.56)	0.88 (0.60, 1.28)
Pregnancy problems	1.02 (0.80, 1.31)	0.93 (0.61, 1.41)	0.97 (0.74, 1.28)	1.08 (0.70, 1.66)	1.26 (0.88, 1.82)
Relationship problems	0.84 (0.61, 1.17)	1.23 (0.73, 2.04)	0.81 (0.56, 1.18)	1.63 (0.97, 2.75)	0.84 (0.51, 1.38)
Residential move	0.75 (0.57, 0.99)	0.63 (0.39, 1.01)	0.80 (0.59, 1.09)	1.02 (0.62, 1.65)	0.81 (0.53, 1.22)
Problems with other children	1.42 (0.94, 2.12)	0.96 (0.49, 1.87)	1.51 (0.99, 2.30)	1.45 (0.77, 2.74)	1.20 (0.69, 2.11)

^†^odds ratio and 95% confidence intervals, each line reflects a separate logistic regression model. Model adjusted for maternal age at pregnancy, maternal education at pregnancy and maternal smoking in pregnancy. LRTI: Lower respiratory tract infection; URTI: Upper respiratory tract infection; GI: Gastrointestinal infection; Other: Genitourinary infection, skin and soft tissue infection or other viral infection.

When analyses were restricted to infection-related hospitalisations before age five years, maternal stress exposure in pregnancy was associated with overall infection-related hospitalisation in offspring and specifically for LRTI, URTI and GI infections (**Table 4**). These results were mostly observed for male children, with female children at higher risk only for URTI admissions before the age of five while each additional stressful event increased the risk of admission for male children for any infectious disease, LRTI, URTI, GI and other infections.

**Table 4:** Binary logistic regression analyses showing the relationship between increasing life stress events and infection-related hospitalisation before the age of 5 years (N = 2,141).

	Life stress events (both) OR (95% CI)^†^	Life stress events (males) OR (95% CI)^†^	Life stress events (females) OR (95% CI)^†^
**Any ID** (n = 696)	1.07 (1.02, 1.18)	1.11 (1.05, 1.18)	1.03 (0.96, 1.09)
**LRTI** (n = 151)	1.08 (1.00, 1.17)	1.17 (1.07, 1.29)	0.95 (0.84, 1.08)
**URTI** (n = 293)	1.11 (1.04, 1.17)	1.12 (1.03, 1.20)	1.09 (1.00, 1.19)
**GI** (n = 127)	1.09 (1.01, 1.18)	1.17 (1.05, 1.30)	1.00 (0.89, 1.14)
**Other Viral** (n = 82)	1.05 (0.95, 1.16)	1.18 (1.03, 1.35)	0.90 (0.76, 1.06)

^†^odds ratio and 95% confidence intervals, Model adjusted for maternal age at pregnancy, maternal education at pregnancy and maternal smoking in pregnancy. ID: Any infectious disease; LRTI: Lower respiratory tract infection; URTI: Upper respiratory tract infection; GI: Gastrointestinal infection; Other: Genitourinary infection, skin and soft tissue infection or other viral infection.

## Discussion

In this large prospective study of well-phenotyped children and adolescents, exposure to prenatal stress was associated with a higher risk for overall and clinical category-specific infection-related hospitalisation in male offspring. There was evidence of a dose-response; more stress events in pregnancy were linked to a greater likelihood for overall infection-related hospitalisations, and specifically with LRTI, URTI, GI and other hospitalisations with a largely viral aetiology. There were associations with the timing of stress and the type of stress and infection-related hospitalisations. The associations between prenatal stress and childhood infection were almost exclusively observed only in male offspring.

Our findings are consistent with and complement recent studies that reported similar associations between stress in pregnancy and infection in offspring [13,14], and our cohort allowed more detailed investigation of exposures and outcomes than larger population-wide studies could provide. We were able to analyse additional stress exposures (in addition to death of an immediate family member and parental divorce—the only data available in prior studies [14]) and the clinical type of infection, in addition to overall infection-related hospitalisations. We provide evidence of association between these common but less severe exposures and specific types of infection-related hospitalisation. Our smaller cohort size does however limit our analyses with regards to less common stress exposures such as death of a family member. In contrast to other studies of maternal stress and adverse childhood outcomes with shorter follow-up [8–11], the associations reported here include a longer follow-up period of childhood and adolescence to age 16 years.

The sex-specific differences regarding the influence of prenatal stress on offspring vulnerability to infectious diseases were striking. It is widely appreciated that males are more susceptible to childhood infections than females (16), which partly reflects differences in immune development and suboptimal responses to pathogens and vaccines in boys [15,28]. The programming role that prenatal stress may play in determining immune ontogeny and infection risk is less well understood. Prior studies have found maternal stress can increase vulnerability for boys [29], with the placenta suggested to contribute to sexually dimorphic immune responses from early in fetal development [30,31]. A widely accepted mechanism contributing to the relationship between prenatal stress and offspring health is the transplacental effects of elevated maternal glucocorticoids on the development of the fetal HPA axis. The placenta has identical chromosomes to the fetus, and while often overlooked, there are clear sex differences in placental size, shape, and function. Sex differences in placental gene expression include 11β-hydroxysteroid dehydrogenases (that convert 11-keto products to active cortisol), which is reduced in male placentae [32]. Consequently male fetuses are exposed to greater surges in transplacental maternal cortisol following stress events in pregnancy [33]. Our findings regarding the heightened risk for boys reveals sex to be a key determinant of later disease vulnerability, given the marked differences between hospital admissions for males and females.

We used infection-related hospitalisation as a marker of clinical severity as it is less prone to bias by parental anxiety and health-seeking behaviour than primary care or emergency department visits [34], however recent analysis of hospitalisations for acute LRI in a population cohort of WA births has shown a linear relationship between increasing levels of socioeconomic disadvantage and increasing rates of infection [35]. Our data were limited to more severe infection resulting in hospitalisation. The associations we observe may be much more marked if our outcomes were able to include out of hospital attendance through emergency departments and primary care. These data were not available for the current study, but would allow more detailed outcome measures in the future. Other studies report that prenatal stress is associated with increased offspring primary care visits until age 35 years.

Our epidemiological observational study was not designed to test underlying mechanisms, but disruption of a number of biological pathways is plausible and many of these putative mechanisms also show sex differences. Firstly intense stress characterised by intrusive thoughts and emotional distress regarding the fetus during pregnancy have been associated with reduced feto-placental volume blood flow in the third trimester, which may contribute to suboptimal offspring immunity by reduced fetal growth [27]. Secondly chronic stress may program a pro-inflammatory phenotype in monocytes and macrophages that increases susceptibility to infection, possibly via the hypothalamic-pituitary axis (HPA) [2]. Stress-induced changes in the developing fetal HPA axis has been posited as one of the main pathways linking prenatal stress to later adverse health and development outcomes for children [36]. Stress exposure in pregnancy may result in high levels of maternal glucocorticoids that overwhelm the protective mechanisms in the placenta, leading to compromised immune development and this may be particularly marked for boys [30,37,38]. Prenatal stress exposure has been associated with altered innate and adaptive immune responses in human cord blood mononuclear cells. This is a suggested mechanism for subsequent increased risk of allergic disease [39], and may be relevant for infection-related outcomes, although it is largely unexplored. Mothers with perinatal depression may have immune suppression due to HPA axis dysregulation and this may directly impair the children’s immune and endocrine development [40]. Prenatal stress has an established link with preterm birth, particularly for male offspring [41], which subsequently can influence vulnerability to later infectious diseases [27]. Therefore it is plausible that preterm birth is a mediator in the relationship between prenatal stress and later hospitalisation for infectious diseases.

There are also psychosocial explanations for the observed associations, which are not mutually exclusive. The strong correlation between increased stress exposure and social disadvantage increases the likelihood that a fetus exposed to stress prenatally is also at increased disadvantage in childhood, including poorer hygiene and health practices [1], which increase infection risk. Stress is also linked to pregnancy behaviours such as cigarette smoking, excessive alcohol intake and abuse of illicit or prescription drugs [38], which may increase the vulnerability of child health postnatally. Mothers who are stressed during pregnancy may be more anxious postnatally, and this may increase health-seeking behaviour when the child is unwell [14], although the use of hospitalisation as an outcome aimed to reduce this. Moreover, a study that adjusted for postnatal maternal stress found a significant relationship between prenatal stress and infection-related hospitalisation [13].

Strengths of our study include the prospective measure of stressful life events and the comprehensive linked hospitalisation data that eliminates the reliance on parental recall of hospital admissions throughout their child’s life. A further strength of the study is availability of data throughout the entire period of childhood to age 16. We acknowledge that our inability to measure postnatal stress and at the time of hospitalisation is a limitation, along with not being able to consider other variables along the pathway to hospital admission. It is possible that our results showing pregnancy problems to be a risk for infection-related hospitalisation were due to the pregnancy problem itself causing compromised immunity rather than the associated stress. We have no way to clarify the nature or severity of reported pregnancy problems to better understand this possibility. We also accept that for some less frequent causes of infection-related hospitalisation (such as invasive bacterial infection) the sample size was too small to draw meaningful conclusions. The cohort is predominantly European-Caucasian and further studies are required before the findings can be generalized to Indigenous Australians and other populations. We adjusted for multiple measures of socioeconomic status, but we cannot exclude residual confounding arising from social gradients in health service access and utilization. As with any observational study, it is possible that measurement error may have influenced our study but we took as much care as possible to ensure that our scales and data were accurately measuring our predictors, confounders and outcomes.

In a cohort of over 2000 children, increased exposure to stressful life events *in utero* is associated with a greater susceptibility for male children to overall and clinical category-specific infection-related hospitalisation, particularly for common viral infections of the respiratory and gastrointestinal tracts. Further, we have identified the types and timing of stress in pregnancy that appear most deleterious. Further studies should explore the underlying immunological mechanisms to inform interventions for those at increased risk of severe childhood infections.

## Supporting information

S1 Table Type of stress experienced at each measured time point in pregnancy (N = 2141). (DOCX)

S2 Table Distribution of stressful life events in pregnancy (N = 2141). (DOCX)

S1 File (PDF)
