# Supplementary material for: Maternal prenatal stress exposure and sex-specific risk of severe infection in offspring
Source: PLoS One. 2021 Jan 29;16(1):e0245747. doi: 10.1371/journal.pone.0245747 (PMC7845992; doi:10.1371/journal.pone.0245747)
Supplement: S1 Table — (DOCX) [file pone.0245747.s001.docx]

**S1 Table: Type of stress experienced at each measured time point in pregnancy (N=2141)**

|  | **18 weeks** | | **34 weeks** | |
| --- | --- | --- | --- | --- |
| **Type of Stress Experienced** | **n** | **%** | **n** | **%** |
| Death of a relative or friend  Financial problems  Pregnancy problems | 156  664  555 | 7.3  31.0  25.9 | 155  636  451 | 7.2  29.7  21.1 |
| Relationship problems  Residential move  Problems with other children | 241  332  136 | 11.3  15.5  6.4 | 209  399  151 | 9.8  18.6  7.1 |
