# Supplementary material for: Maternal prenatal stress exposure and sex-specific risk of severe infection in offspring
Source: PLoS One. 2021 Jan 29;16(1):e0245747. doi: 10.1371/journal.pone.0245747 (PMC7845992; doi:10.1371/journal.pone.0245747)
Supplement: S2 Table — (DOCX) [file pone.0245747.s002.docx]

**S2 Table: Distribution of stressful life events in pregnancy (N=2141)**

|  | **Total** | | | | | | **18 weeks** | | **34 weeks** | |
| --- | --- | --- | --- | --- | --- | --- | --- | --- | --- | --- |
|  | **All** | | **Girls** | | **Boys** | |  | | | |
| **Stress Events Reported** | **n** | **%** | **n** | **%** | **n** | **%** | **n** | **%** | **n** | **%** |
| **0**  **1**  **2**  **3**  **4**  **5**  **6**  **7**  **8**  **9**  **10 11**  **12** | 468  458  399  310  195  136  75  55  23  11  7  2  2 | 21.9  21.4  18.6  14.5  9.1  6.4  3.5  2.6  1.1  0.5  0.3  0.1  0.1 | 221  220  194  147  89  63  41  26  10  3  4  2  0 | 21.7  21.6  19.0  14.4  8.7  6.2  4.0  2.5  1.0  0.3  0.4  0.2  - | 247  238  205  163  106  73  34  29  13  8  3  0  2 | 22.0  21.2  18.3  14.5  9.5  6.5  3.0  2.6  1.2  0.7  0.3  -  0.2 | 838  769  430  218  89  31  9  2  1  -  -  -  - | 35.1  32.2  18.0  9.1  3.7  1.3  0.4  0.1  <0.1  -  -  -  - | 868  624  363  191  67  21  7  1  -  -  -  -  - | 40.5  29.1  16.9  8.9  3.1  1.0  0.3  <0.1  -  -  -  -  - |
