# Supplementary material for: Maternal prenatal stress exposure and sex-specific risk of severe infection in offspring
Source: PLoS One. 2021 Jan 29;16(1):e0245747. doi: 10.1371/journal.pone.0245747 (PMC7845992; doi:10.1371/journal.pone.0245747)
Supplement: S1 File — (PDF) [file pone.0245747.s003.pdf]

THE LANCET Infectious Diseases

Supplementary webappendix

This webappendix formed part of the original submission and has been peer reviewed. We post it as supplied by the authors.

Supplement to: Miller JE, Hammond GC, Strunk T, et al. Association of gestational age and growth measures at birth with infection-related admissions to hospital throughout childhood: a population-based, data-linkage study from Western Australia. *Lancet Infect Dis* 2016; published online April 1. http://dx.doi.org/10.1016/S1473-3099(16)00150-X.

Supplementary Table: ICD codes for all infection diagnostic group

001.0	CHOLERA D/T VIB CHOLERAE (ICD9)
001.1	CHOLERA D/T VIB EL TOR (ICD9)
001.9	CHOLERA NOS (ICD9)
002.0	TYPHOID FEVER (ICD9)
002.1	PARATYPHOID FEVER A (ICD9)
002.2	PARATYPHOID FEVER B (ICD9)
002.3	PARATYPHOID FEVER C (ICD9)
002.9	PARATYPHOID FEVER NOS (ICD9)
003.0	SALMONELLA ENTERITIS (ICD9)
003.1	SALMONELLA SEPTICEMIA (ICD9)
003.20	LOCAL SALMONELLA INF NOS (ICD9)
003.21	SALMONELLA MENINGITIS (ICD9)
003.22	SALMONELLA PNEUMONIA (ICD9)
003.23	SALMONELLA ARTHRITIS (ICD9)
003.24	SALMONELLA OSTEOMYELITIS (ICD9)
003.29	LOCAL SALMONELLA INF NEC (ICD9)
003.8	SALMONELLA INFECTION NEC (ICD9)
003.9	SALMONELLA INFECTION NOS (ICD9)
004.0	SHIGELLA DYSENTERIAE (ICD9)
004.1	SHIGELLA FLEXNERI (ICD9)
004.2	SHIGELLA BOYDII (ICD9)
004.3	SHIGELLA SONNEI (ICD9)
004.8	SHIGELLA INFECTION NEC (ICD9)
004.9	SHIGELLOSIS NOS (ICD9)
005.0	STAPH FOOD POISONING (ICD9)
005.1	BOTULISM (ICD9)
005.2	FOOD POIS D/T C. PERFRIN (ICD9)
005.3	FOOD POIS: CLOSTRID NEC (ICD9)
005.4	FOOD POIS: V. PARAHAEM (ICD9)
005.8	BACT FOOD POISONING NEC (End 1995) (ICD9)
005.81	FOOD POISONING VIBRIO (Begin 1995) (ICD9)
005.89	OTH BACT FOOD POISONING (Begin 1995) (ICD9)
005.9	FOOD POISONING NOS (ICD9)
006.0	AC AMEBIASIS W/O ABSCESS (ICD9)
006.1	CHR AMEBIASIS W/O ABSCES (ICD9)
006.2	AMEBIC NONDYSENT COLITIS (ICD9)
006.3	AMEBIC LIVER ABSCESS (ICD9)
006.4	AMEBIC LUNG ABSCESS (ICD9)
006.5	AMEBIC BRAIN ABSCESS (ICD9)
006.6	AMEBIC SKIN ULCERATION (ICD9)
006.8	AMEBIC INFECTION NEC (ICD9)
006.9	AMEBIASIS NOS (ICD9)
007.0	BALANTIDIASIS (ICD9)
007.1	GIARDIASIS (ICD9)
007.2	COCCIDIOSIS (ICD9)
007.3	INTEST TRICHOMONIASIS (ICD9)
007.4	OT PROTOZ INTEST DX- CRYP (Begin 1997) (ICD9)
007.5	CYCLOSPORIASIS (Begin 2000) (ICD9)
007.8	PROTOZOAL INTEST DIS NEC (ICD9)
007.9	PROTOZOAL INTEST DIS NOS (ICD9)
008.0	E. COLI ENTERITIS (Begin 1980 (ICD9)
008.00	E. COLI ENTERITIS-NOS (Begin 1992) (ICD9)
008.01	E. COLI ENTERITIS-PATH (Begin 1992) (ICD9)
008.02	E. COLI ENTERITIS-TOX (Begin 1992) (ICD9)
008.03	E. COLI ENTERITIS-INVAS (Begin 1992) (ICD9)
008.04	E. COLI ENTERITIS-HEMOR (Begin 1992) (ICD9)
008.09	E. COLI ENTERITIS-OTHER (Begin 1992) (ICD9)
008.1	ARIZONA ENTERITIS (ICD9)

008.2 AEROBACTER ENTERITIS (ICD9)
008.3 PROTEUS ENTERITIS (ICD9)
008.41 STAPHYLOCOCC ENTERITIS (ICD9)
008.42 PSEUDOMONAS ENTERITIS (ICD9)
008.43 CAMPYLOBACTER (Begin 1992) (ICD9)
008.44 YERSINIA (Begin 1992) (ICD9)
008.45 CLOSTRIDIUM DIF (Begin 1992) (ICD9)
008.46 OTHER ANAEROBES (Begin 1992) (ICD9)
008.47 OTH GRAM NEG BACT (Begin 1992) (ICD9)
008.49 BACTERIAL ENTERITIS NEC (ICD9)
008.5 BACTERIAL ENTERITIS NOS (ICD9)
008.6 VIRAL ENTERITIS NEC (Begin 1980 (ICD9)
008.61 ROTAVIRUS ENTERITIS (Begin 1992) (ICD9)
008.62 ADENOVIR ENTERITIS (Begin 1992) (ICD9)
008.63 NORWALK VIR ENTERITIS (Begin 1992) (ICD9)
008.64 SML ROUND VIR ENTERITIS (Begin 1992) (ICD9)
008.65 CALCIVIRUS ENTERITIS (Begin 1992) (ICD9)
008.66 ASTROVIRUS ENTERITIS (Begin 1992) (ICD9)
008.67 ENTERITIS NEC (Begin 1992) (ICD9)
008.69 ENTERITIS NOS (Begin 1992) (ICD9)
008.8 VIRAL ENTERITIS NOS (ICD9)
009.0 INFECTIOUS ENTERITIS NOS (ICD9)
009.1 ENTERITIS OF INFECT ORIG (ICD9)
009.2 INFECTIOUS DIARRHEA NOS (ICD9)
009.3 DIARRHEA OF INFECT ORIG (ICD9)
010.00 PRIM TB COMPLEX-UNSPEC (ICD9)
010.01 PRIM TB COMPLEX-NO EXAM (ICD9)
010.02 PRIM TB COMPLEX-EXM UNKN (ICD9)
010.03 PRIM TB COMPLEX-MICRO DX (ICD9)
010.04 PRIM TB COMPLEX-CULT DX (ICD9)
010.05 PRIM TB COMPLEX-HISTO DX (ICD9)
010.06 PRIM TB COMPLEX-OTH TEST (ICD9)
010.10 PRIM TB PLEURISY-UNSPEC (ICD9)
010.11 PRIM TB PLEURISY-NO EXAM (ICD9)
010.12 PRIM TB PLEUR-EXAM UNKN (ICD9)
010.13 PRIM TB PLEURIS-MICRO DX (ICD9)
010.14 PRIM TB PLEURISY-CULT DX (ICD9)
010.15 PRIM TB PLEURIS-HISTO DX (ICD9)
010.16 PRIM TB PLEURIS-OTH TEST (ICD9)
010.80 PRIM PROG TB NEC-UNSPEC (ICD9)
010.81 PRIM PROG TB NEC-NO EXAM (ICD9)
010.82 PRIM PR TB NEC-EXAM UNKN (ICD9)
010.83 PRIM PRG TB NEC-MICRO DX (ICD9)
010.84 PRIM PROG TB NEC-CULT DX (ICD9)
010.85 PRIM PRG TB NEC-HISTO DX (ICD9)
010.86 PRIM PRG TB NEC-OTH TEST (ICD9)
010.90 PRIMARY TB NOS-UNSPEC (ICD9)
010.91 PRIMARY TB NOS-NO EXAM (ICD9)
010.92 PRIMARY TB NOS-EXAM UNKN (ICD9)
010.93 PRIMARY TB NOS-MICRO DX (ICD9)
010.94 PRIMARY TB NOS-CULT DX (ICD9)
010.95 PRIMARY TB NOS-HISTO DX (ICD9)
010.96 PRIMARY TB NOS-OTH TEST (ICD9)
011.00 TB LUNG INFILTR-UNSPEC (ICD9)
011.01 TB LUNG INFILTR-NO EXAM (ICD9)
011.02 TB LUNG INFILTR-EXM UNKN (ICD9)
011.03 TB LUNG INFILTR-MICRO DX (ICD9)
011.04 TB LUNG INFILTR-CULT DX (ICD9)
011.05 TB LUNG INFILTR-HISTO DX (ICD9)
011.06 TB LUNG INFILTR-OTH TEST (ICD9)

011.10 TB LUNG NODULAR-UNSPEC (ICD9)
011.11 TB LUNG NODULAR-NO EXAM (ICD9)
011.12 TB LUNG NODUL-EXAM UNKN (ICD9)
011.13 TB LUNG NODULAR-MICRO DX (ICD9)
011.14 TB LUNG NODULAR-CULT DX (ICD9)
011.15 TB LUNG NODULAR-HISTO DX (ICD9)
011.16 TB LUNG NODULAR-OTH TEST (ICD9)
011.20 TB LUNG W CAVITY-UNSPEC (ICD9)
011.21 TB LUNG W CAVITY-NO EXAM (ICD9)
011.22 TB LUNG CAVITY-EXAM UNKN (ICD9)
011.23 TB LUNG W CAVIT-MICRO DX (ICD9)
011.24 TB LUNG W CAVITY-CULT DX (ICD9)
011.25 TB LUNG W CAVIT-HISTO DX (ICD9)
011.26 TB LUNG W CAVIT-OTH TEST (ICD9)
011.30 TB OF BRONCHUS-UNSPEC (ICD9)
011.31 TB OF BRONCHUS-NO EXAM (ICD9)
011.32 TB OF BRONCHUS-EXAM UNKN (ICD9)
011.33 TB OF BRONCHUS-MICRO DX (ICD9)
011.34 TB OF BRONCHUS-CULT DX (ICD9)
011.35 TB OF BRONCHUS-HISTO DX (ICD9)
011.36 TB OF BRONCHUS-OTH TEST (ICD9)
011.40 TB LUNG FIBROSIS-UNSPEC (ICD9)
011.41 TB LUNG FIBROSIS-NO EXAM (ICD9)
011.42 TB LUNG FIBROS-EXAM UNKN (ICD9)
011.43 TB LUNG FIBROS-MICRO DX (ICD9)
011.44 TB LUNG FIBROSIS-CULT DX (ICD9)
011.45 TB LUNG FIBROS-HISTO DX (ICD9)
011.46 TB LUNG FIBROS-OTH TEST (ICD9)
011.50 TB BRONCHIECTASIS-UNSPEC (ICD9)
011.51 TB BRONCHIECT-NO EXAM (ICD9)
011.52 TB BRONCHIECT-EXAM UNKN (ICD9)
011.53 TB BRONCHIECT-MICRO DX (ICD9)
011.54 TB BRONCHIECT-CULT DX (ICD9)
011.55 TB BRONCHIECT-HISTO DX (ICD9)
011.56 TB BRONCHIECT-OTH TEST (ICD9)
011.60 TB PNEUMONIA-UNSPEC (ICD9)
011.61 TB PNEUMONIA-NO EXAM (ICD9)
011.62 TB PNEUMONIA-EXAM UNKN (ICD9)
011.63 TB PNEUMONIA-MICRO DX (ICD9)
011.64 TB PNEUMONIA-CULT DX (ICD9)
011.65 TB PNEUMONIA-HISTO DX (ICD9)
011.66 TB PNEUMONIA-OTH TEST (ICD9)
011.70 TB PNEUMOTHORAX-UNSPEC (ICD9)
011.71 TB PNEUMOTHORAX-NO EXAM (ICD9)
011.72 TB PNEUMOTHORX-EXAM UNKN (ICD9)
011.73 TB PNEUMOTHORAX-MICRO DX (ICD9)
011.74 TB PNEUMOTHORAX-CULT DX (ICD9)
011.75 TB PNEUMOTHORAX-HISTO DX (ICD9)
011.76 TB PNEUMOTHORAX-OTH TEST (ICD9)
011.80 PULMONARY TB NEC-UNSPEC (ICD9)
011.81 PULMONARY TB NEC-NO EXAM (ICD9)
011.82 PULMON TB NEC-EXAM UNKN (ICD9)
011.83 PULMON TB NEC-MICRO DX (ICD9)
011.84 PULMON TB NEC-CULT DX (ICD9)
011.85 PULMON TB NEC-HISTO DX (ICD9)
011.86 PULMON TB NEC-OTH TEST (ICD9)
011.90 PULMONARY TB NOS-UNSPEC (ICD9)
011.91 PULMONARY TB NOS-NO EXAM (ICD9)
011.92 PULMON TB NOS-EXAM UNKN (ICD9)
011.93 PULMON TB NOS-MICRO DX (ICD9)

011.94 PULMON TB NOS-CULT DX (ICD9)
011.95 PULMON TB NOS-HISTO DX (ICD9)
011.96 PULMON TB NOS-OTH TEST (ICD9)
012.00 TB PLEURISY-UNSPEC (ICD9)
012.01 TB PLEURISY-NO EXAM (ICD9)
012.02 TB PLEURISY-EXAM UNKN (ICD9)
012.03 TB PLEURISY-MICRO DX (ICD9)
012.04 TB PLEURISY-CULT DX (ICD9)
012.05 TB PLEURISY-HISTOLOG DX (ICD9)
012.06 TB PLEURISY-OTH TEST (ICD9)
012.10 TB THORACIC NODES-UNSPEC (ICD9)
012.11 TB THORAX NODE-NO EXAM (ICD9)
012.12 TB THORAX NODE-EXAM UNKN (ICD9)
012.13 TB THORAX NODE-MICRO DX (ICD9)
012.14 TB THORAX NODE-CULT DX (ICD9)
012.15 TB THORAX NODE-HISTO DX (ICD9)
012.16 TB THORAX NODE-OTH TEST (ICD9)
012.20 ISOL TRACHEAL TB-UNSPEC (ICD9)
012.21 ISOL TRACHEAL TB-NO EXAM (ICD9)
012.22 ISOL TRACH TB-EXAM UNKN (ICD9)
012.23 ISOLAT TRACH TB-MICRO DX (ICD9)
012.24 ISOL TRACHEAL TB-CULT DX (ICD9)
012.25 ISOLAT TRACH TB-HISTO DX (ICD9)
012.26 ISOLAT TRACH TB-OTH TEST (ICD9)
012.30 TB LARYNGITIS-UNSPEC (ICD9)
012.31 TB LARYNGITIS-NO EXAM (ICD9)
012.32 TB LARYNGITIS-EXAM UNKN (ICD9)
012.33 TB LARYNGITIS-MICRO DX (ICD9)
012.34 TB LARYNGITIS-CULT DX (ICD9)
012.35 TB LARYNGITIS-HISTO DX (ICD9)
012.36 TB LARYNGITIS-OTH TEST (ICD9)
012.80 RESP TB NEC-UNSPEC (ICD9)
012.81 RESP TB NEC-NO EXAM (ICD9)
012.82 RESP TB NEC-EXAM UNKN (ICD9)
012.83 RESP TB NEC-MICRO DX (ICD9)
012.84 RESP TB NEC-CULT DX (ICD9)
012.85 RESP TB NEC-HISTO DX (ICD9)
012.86 RESP TB NEC-OTH TEST (ICD9)
013.00 TB MENINGITIS-UNSPEC (ICD9)
013.01 TB MENINGITIS-NO EXAM (ICD9)
013.02 TB MENINGITIS-EXAM UNKN (ICD9)
013.03 TB MENINGITIS-MICRO DX (ICD9)
013.04 TB MENINGITIS-CULT DX (ICD9)
013.05 TB MENINGITIS-HISTO DX (ICD9)
013.06 TB MENINGITIS-OTH TEST (ICD9)
013.10 TUBRCLMA MENINGES-UNSPEC (ICD9)
013.11 TUBRCLMA MENING-NO EXAM (ICD9)
013.12 TUBRCLMA MENIN-EXAM UNKN (ICD9)
013.13 TUBRCLMA MENING-MICRO DX (ICD9)
013.14 TUBRCLMA MENING-CULT DX (ICD9)
013.15 TUBRCLMA MENING-HISTO DX (ICD9)
013.16 TUBRCLMA MENING-OTH TEST (ICD9)
013.20 TUBERCULOMA BRAIN-UNSPEC (ICD9)
013.21 TUBRCLOMA BRAIN-NO EXAM (ICD9)
013.22 TUBRCLMA BRAIN-EXAM UNKN (ICD9)
013.23 TUBRCLOMA BRAIN-MICRO DX (ICD9)
013.24 TUBRCLOMA BRAIN-CULT DX (ICD9)
013.25 TUBRCLOMA BRAIN-HISTO DX (ICD9)
013.26 TUBRCLOMA BRAIN-OTH TEST (ICD9)
013.30 TB BRAIN ABSCESS-UNSPEC (ICD9)

013.31 TB BRAIN ABSCESS-NO EXAM (ICD9)
013.32 TB BRAIN ABSC-EXAM UNKN (ICD9)
013.33 TB BRAIN ABSC-MICRO DX (ICD9)
013.34 TB BRAIN ABSCESS-CULT DX (ICD9)
013.35 TB BRAIN ABSC-HISTO DX (ICD9)
013.36 TB BRAIN ABSC-OTH TEST (ICD9)
013.40 TUBRCLMA SP CORD-UNSPEC (ICD9)
013.41 TUBRCLMA SP CORD-NO EXAM (ICD9)
013.42 TUBRCLMA SP CD-EXAM UNKN (ICD9)
013.43 TUBRCLMA SP CRD-MICRO DX (ICD9)
013.44 TUBRCLMA SP CORD-CULT DX (ICD9)
013.45 TUBRCLMA SP CRD-HISTO DX (ICD9)
013.46 TUBRCLMA SP CRD-OTH TEST (ICD9)
013.50 TB SP CRD ABSCESS-UNSPEC (ICD9)
013.51 TB SP CRD ABSC-NO EXAM (ICD9)
013.52 TB SP CRD ABSC-EXAM UNKN (ICD9)
013.53 TB SP CRD ABSC-MICRO DX (ICD9)
013.54 TB SP CRD ABSC-CULT DX (ICD9)
013.55 TB SP CRD ABSC-HISTO DX (ICD9)
013.56 TB SP CRD ABSC-OTH TEST (ICD9)
013.60 TB ENCEPHALITIS-UNSPEC (ICD9)
013.61 TB ENCEPHALITIS-NO EXAM (ICD9)
013.62 TB ENCEPHALIT-EXAM UNKN (ICD9)
013.63 TB ENCEPHALITIS-MICRO DX (ICD9)
013.64 TB ENCEPHALITIS-CULT DX (ICD9)
013.65 TB ENCEPHALITIS-HISTO DX (ICD9)
013.66 TB ENCEPHALITIS-OTH TEST (ICD9)
013.80 CNS TB NEC-UNSPEC (ICD9)
013.81 CNS TB NEC-NO EXAM (ICD9)
013.82 CNS TB NEC-EXAM UNKN (ICD9)
013.83 CNS TB NEC-MICRO DX (ICD9)
013.84 CNS TB NEC-CULT DX (ICD9)
013.85 CNS TB NEC-HISTO DX (ICD9)
013.86 CNS TB NEC-OTH TEST (ICD9)
013.90 CNS TB NOS-UNSPEC (ICD9)
013.91 CNS TB NOS-NO EXAM (ICD9)
013.92 CNS TB NOS-EXAM UNKN (ICD9)
013.93 CNS TB NOS-MICRO DX (ICD9)
013.94 CNS TB NOS-CULT DX (ICD9)
013.95 CNS TB NOS-HISTO DX (ICD9)
013.96 CNS TB NOS-OTH TEST (ICD9)
014.00 TB PERITONITIS-UNSPEC (ICD9)
014.01 TB PERITONITIS-NO EXAM (ICD9)
014.02 TB PERITONITIS-EXAM UNKN (ICD9)
014.03 TB PERITONITIS-MICRO DX (ICD9)
014.04 TB PERITONITIS-CULT DX (ICD9)
014.05 TB PERITONITIS-HISTO DX (ICD9)
014.06 TB PERITONITIS-OTH TEST (ICD9)
014.80 INTESTINAL TB NEC-UNSPEC (ICD9)
014.81 INTESTIN TB NEC-NO EXAM (ICD9)
014.82 INTEST TB NEC-EXAM UNKN (ICD9)
014.83 INTESTIN TB NEC-MICRO DX (ICD9)
014.84 INTESTIN TB NEC-CULT DX (ICD9)
014.85 INTESTIN TB NEC-HISTO DX (ICD9)
014.86 INTESTIN TB NEC-OTH TEST (ICD9)
015.00 TB OF VERTEBRA-UNSPEC (ICD9)
015.01 TB OF VERTEBRA-NO EXAM (ICD9)
015.02 TB OF VERTEBRA-EXAM UNKN (ICD9)
015.03 TB OF VERTEBRA-MICRO DX (ICD9)
015.04 TB OF VERTEBRA-CULT DX (ICD9)

015.05 TB OF VERTEBRA-HISTO DX (ICD9)
015.06 TB OF VERTEBRA-OTH TEST (ICD9)
015.10 TB OF HIP-UNSPEC (ICD9)
015.11 TB OF HIP-NO EXAM (ICD9)
015.12 TB OF HIP-EXAM UNKN (ICD9)
015.13 TB OF HIP-MICRO DX (ICD9)
015.14 TB OF HIP-CULT DX (ICD9)
015.15 TB OF HIP-HISTO DX (ICD9)
015.16 TB OF HIP-OTH TEST (ICD9)
015.20 TB OF KNEE-UNSPEC (ICD9)
015.21 TB OF KNEE-NO EXAM (ICD9)
015.22 TB OF KNEE-EXAM UNKN (ICD9)
015.23 TB OF KNEE-MICRO DX (ICD9)
015.24 TB OF KNEE-CULT DX (ICD9)
015.25 TB OF KNEE-HISTO DX (ICD9)
015.26 TB OF KNEE-OTH TEST (ICD9)
015.50 TB OF LIMB BONES-UNSPEC (ICD9)
015.51 TB LIMB BONES-NO EXAM (ICD9)
015.52 TB LIMB BONES-EXAM UNKN (ICD9)
015.53 TB LIMB BONES-MICRO DX (ICD9)
015.54 TB LIMB BONES-CULT DX (ICD9)
015.55 TB LIMB BONES-HISTO DX (ICD9)
015.56 TB LIMB BONES-OTH TEST (ICD9)
015.60 TB OF MASTOID-UNSPEC (ICD9)
015.61 TB OF MASTOID-NO EXAM (ICD9)
015.62 TB OF MASTOID-EXAM UNKN (ICD9)
015.63 TB OF MASTOID-MICRO DX (ICD9)
015.64 TB OF MASTOID-CULT DX (ICD9)
015.65 TB OF MASTOID-HISTO DX (ICD9)
015.66 TB OF MASTOID-OTH TEST (ICD9)
015.70 TB OF BONE NEC-UNSPEC (ICD9)
015.71 TB OF BONE NEC-NO EXAM (ICD9)
015.72 TB OF BONE NEC-EXAM UNKN (ICD9)
015.73 TB OF BONE NEC-MICRO DX (ICD9)
015.74 TB OF BONE NEC-CULT DX (ICD9)
015.75 TB OF BONE NEC-HISTO DX (ICD9)
015.76 TB OF BONE NEC-OTH TEST (ICD9)
015.80 TB OF JOINT NEC-UNSPEC (ICD9)
015.81 TB OF JOINT NEC-NO EXAM (ICD9)
015.82 TB JOINT NEC-EXAM UNKN (ICD9)
015.83 TB OF JOINT NEC-MICRO DX (ICD9)
015.84 TB OF JOINT NEC-CULT DX (ICD9)
015.85 TB OF JOINT NEC-HISTO DX (ICD9)
015.86 TB OF JOINT NEC-OTH TEST (ICD9)
015.90 TB BONE/JOINT NOS-UNSPEC (ICD9)
015.91 TB BONE/JT NOS-NO EXAM (ICD9)
015.92 TB BONE/JT NOS-EXAM UNKN (ICD9)
015.93 TB BONE/JT NOS-MICRO DX (ICD9)
015.94 TB BONE/JT NOS-CULT DX (ICD9)
015.95 TB BONE/JT NOS-HISTO DX (ICD9)
015.96 TB BONE/JT NOS-OTH TEST (ICD9)
016.00 TB OF KIDNEY-UNSPEC (ICD9)
016.01 TB OF KIDNEY-NO EXAM (ICD9)
016.02 TB OF KIDNEY-EXAM UNKN (ICD9)
016.03 TB OF KIDNEY-MICRO DX (ICD9)
016.04 TB OF KIDNEY-CULT DX (ICD9)
016.05 TB OF KIDNEY-HISTO DX (ICD9)
016.06 TB OF KIDNEY-OTH TEST (ICD9)
016.10 TB OF BLADDER-UNSPEC (ICD9)
016.11 TB OF BLADDER-NO EXAM (ICD9)

016.12 TB OF BLADDER-EXAM UNKN (ICD9)
016.13 TB OF BLADDER-MICRO DX (ICD9)
016.14 TB OF BLADDER-CULT DX (ICD9)
016.15 TB OF BLADDER-HISTO DX (ICD9)
016.16 TB OF BLADDER-OTH TEST (ICD9)
016.20 TB OF URETER-UNSPEC (ICD9)
016.21 TB OF URETER-NO EXAM (ICD9)
016.22 TB OF URETER-EXAM UNKN (ICD9)
016.23 TB OF URETER-MICRO DX (ICD9)
016.24 TB OF URETER-CULT DX (ICD9)
016.25 TB OF URETER-HISTO DX (ICD9)
016.26 TB OF URETER-OTH TEST (ICD9)
016.30 TB URINARY NEC-UNSPEC (ICD9)
016.31 TB URINARY NEC-NO EXAM (ICD9)
016.32 TB URINARY NEC-EXAM UNKN (ICD9)
016.33 TB URINARY NEC-MICRO DX (ICD9)
016.34 TB URINARY NEC-CULT DX (ICD9)
016.35 TB URINARY NEC-HISTO DX (ICD9)
016.36 TB URINARY NEC-OTH TEST (ICD9)
016.40 TB EPIDIDYMIS-UNSPEC (ICD9)
016.41 TB EPIDIDYMIS-NO EXAM (ICD9)
016.42 TB EPIDIDYMIS-EXAM UNKN (ICD9)
016.43 TB EPIDIDYMIS-MICRO DX (ICD9)
016.44 TB EPIDIDYMIS-CULT DX (ICD9)
016.45 TB EPIDIDYMIS-HISTO DX (ICD9)
016.46 TB EPIDIDYMIS-OTH TEST (ICD9)
016.50 TB MALE GENIT NEC-UNSPEC (ICD9)
016.51 TB MALE GEN NEC-NO EXAM (ICD9)
016.52 TB MALE GEN NEC-EX UNKN (ICD9)
016.53 TB MALE GEN NEC-MICRO DX (ICD9)
016.54 TB MALE GEN NEC-CULT DX (ICD9)
016.55 TB MALE GEN NEC-HISTO DX (ICD9)
016.56 TB MALE GEN NEC-OTH TEST (ICD9)
016.60 TB OVARY & TUBE-UNSPEC (ICD9)
016.61 TB OVARY & TUBE-NO EXAM (ICD9)
016.62 TB OVARY/TUBE-EXAM UNKN (ICD9)
016.63 TB OVARY & TUBE-MICRO DX (ICD9)
016.64 TB OVARY & TUBE-CULT DX (ICD9)
016.65 TB OVARY & TUBE-HISTO DX (ICD9)
016.66 TB OVARY & TUBE-OTH TEST (ICD9)
016.70 TB FEMALE GEN NEC-UNSPEC (ICD9)
016.71 TB FEM GEN NEC-NO EXAM (ICD9)
016.72 TB FEM GEN NEC-EXAM UNKN (ICD9)
016.73 TB FEM GEN NEC-MICRO DX (ICD9)
016.74 TB FEM GEN NEC-CULT DX (ICD9)
016.75 TB FEM GEN NEC-HISTO DX (ICD9)
016.76 TB FEM GEN NEC-OTH TEST (ICD9)
016.90 GU TB NOS-UNSPEC (ICD9)
016.91 GU TB NOS-NO EXAM (ICD9)
016.92 GU TB NOS-EXAM UNKN (ICD9)
016.93 GU TB NOS-MICRO DX (ICD9)
016.94 GU TB NOS-CULT DX (ICD9)
016.95 GU TB NOS-HISTO DX (ICD9)
016.96 GU TB NOS-OTH TEST (ICD9)
017.00 TB SKIN/SUBCUTAN-UNSPEC (ICD9)
017.01 TB SKIN/SUBCUT-NO EXAM (ICD9)
017.02 TB SKIN/SUBCUT-EXAM UNKN (ICD9)
017.03 TB SKIN/SUBCUT-MICRO DX (ICD9)
017.04 TB SKIN/SUBCUT-CULT DX (ICD9)
017.05 TB SKIN/SUBCUT-HISTO DX (ICD9)

017.06 TB SKIN/SUBCUT-OTH TEST (ICD9)
017.10 ERYTHEMA NODOS TB-UNSPEC (ICD9)
017.11 ERYTHEM NODOS TB-NO EXAM (ICD9)
017.12 ERYTHEM NOD TB-EXAM UNKN (ICD9)
017.13 ERYTHEM NOD TB-MICRO DX (ICD9)
017.14 ERYTHEM NODOS TB-CULT DX (ICD9)
017.15 ERYTHEM NOD TB-HISTO DX (ICD9)
017.16 ERYTHEM NOD TB-OTH TEST (ICD9)
017.20 TB PERIPH LYMPH-UNSPEC (ICD9)
017.21 TB PERIPH LYMPH-NO EXAM (ICD9)
017.22 TB PERIPH LYMPH-EXAM UNK (ICD9)
017.23 TB PERIPH LYMPH-MICRO DX (ICD9)
017.24 TB PERIPH LYMPH-CULT DX (ICD9)
017.25 TB PERIPH LYMPH-HISTO DX (ICD9)
017.26 TB PERIPH LYMPH-OTH TEST (ICD9)
017.30 TB OF EYE-UNSPEC (ICD9)
017.31 TB OF EYE-NO EXAM (ICD9)
017.32 TB OF EYE-EXAM UNKN (ICD9)
017.33 TB OF EYE-MICRO DX (ICD9)
017.34 TB OF EYE-CULT DX (ICD9)
017.35 TB OF EYE-HISTO DX (ICD9)
017.36 TB OF EYE-OTH TEST (ICD9)
017.40 TB OF EAR-UNSPEC (ICD9)
017.41 TB OF EAR-NO EXAM (ICD9)
017.42 TB OF EAR-EXAM UNKN (ICD9)
017.43 TB OF EAR-MICRO DX (ICD9)
017.44 TB OF EAR-CULT DX (ICD9)
017.45 TB OF EAR-HISTO DX (ICD9)
017.46 TB OF EAR-OTH TEST (ICD9)
017.50 TB OF THYROID-UNSPEC (ICD9)
017.51 TB OF THYROID-NO EXAM (ICD9)
017.52 TB OF THYROID-EXAM UNKN (ICD9)
017.53 TB OF THYROID-MICRO DX (ICD9)
017.54 TB OF THYROID-CULT DX (ICD9)
017.55 TB OF THYROID-HISTO DX (ICD9)
017.56 TB OF THYROID-OTH TEST (ICD9)
017.60 TB OF ADRENAL-UNSPEC (ICD9)
017.61 TB OF ADRENAL-NO EXAM (ICD9)
017.62 TB OF ADRENAL-EXAM UNKN (ICD9)
017.63 TB OF ADRENAL-MICRO DX (ICD9)
017.64 TB OF ADRENAL-CULT DX (ICD9)
017.65 TB OF ADRENAL-HISTO DX (ICD9)
017.66 TB OF ADRENAL-OTH TEST (ICD9)
017.70 TB OF SPLEEN-UNSPEC (ICD9)
017.71 TB OF SPLEEN-NO EXAM (ICD9)
017.72 TB OF SPLEEN-EXAM UNKN (ICD9)
017.73 TB OF SPLEEN-MICRO DX (ICD9)
017.74 TB OF SPLEEN-CULT DX (ICD9)
017.75 TB OF SPLEEN-HISTO DX (ICD9)
017.76 TB OF SPLEEN-OTH TEST (ICD9)
017.80 TB ESOPHAGUS-UNSPEC (ICD9)
017.81 TB ESOPHAGUS-NO EXAM (ICD9)
017.82 TB ESOPHAGUS-EXAM UNKN (ICD9)
017.83 TB ESOPHAGUS-MICRO DX (ICD9)
017.84 TB ESOPHAGUS-CULT DX (ICD9)
017.85 TB ESOPHAGUS-HISTO DX (ICD9)
017.86 TB ESOPHAGUS-OTH TEST (ICD9)
017.90 TB OF ORGAN NEC-UNSPEC (ICD9)
017.91 TB OF ORGAN NEC-NO EXAM (ICD9)
017.92 TB ORGAN NEC-EXAM UNKN (ICD9)

017.93 TB OF ORGAN NEC-MICRO DX (ICD9)
017.94 TB OF ORGAN NEC-CULT DX (ICD9)
017.95 TB OF ORGAN NEC-HISTO DX (ICD9)
017.96 TB OF ORGAN NEC-OTH TEST (ICD9)
018.00 ACUTE MILIARY TB-UNSPEC (ICD9)
018.01 ACUTE MILIARY TB-NO EXAM (ICD9)
018.02 AC MILIARY TB-EXAM UNKN (ICD9)
018.03 AC MILIARY TB-MICRO DX (ICD9)
018.04 ACUTE MILIARY TB-CULT DX (ICD9)
018.05 AC MILIARY TB-HISTO DX (ICD9)
018.06 AC MILIARY TB-OTH TEST (ICD9)
018.80 MILIARY TB NEC-UNSPEC (ICD9)
018.81 MILIARY TB NEC-NO EXAM (ICD9)
018.82 MILIARY TB NEC-EXAM UNKN (ICD9)
018.83 MILIARY TB NEC-MICRO DX (ICD9)
018.84 MILIARY TB NEC-CULT DX (ICD9)
018.85 MILIARY TB NEC-HISTO DX (ICD9)
018.86 MILIARY TB NEC-OTH TEST (ICD9)
018.90 MILIARY TB NOS-UNSPEC (ICD9)
018.91 MILIARY TB NOS-NO EXAM (ICD9)
018.92 MILIARY TB NOS-EXAM UNKN (ICD9)
018.93 MILIARY TB NOS-MICRO DX (ICD9)
018.94 MILIARY TB NOS-CULT DX (ICD9)
018.95 MILIARY TB NOS-HISTO DX (ICD9)
018.96 MILIARY TB NOS-OTH TEST (ICD9)
020.0 BUBONIC PLAGUE (ICD9)
020.1 CELLULOCUTANEOUS PLAGUE (ICD9)
020.2 SEPTICEMIC PLAGUE (ICD9)
020.3 PRIMARY PNEUMONIC PLAGUE (ICD9)
020.4 SECONDARY PNEUMON PLAGUE (ICD9)
020.5 PNEUMONIC PLAGUE NOS (ICD9)
020.8 OTHER TYPES OF PLAGUE (ICD9)
020.9 PLAGUE NOS (ICD9)
021.0 ULCEROGLANDUL TULAREMIA (ICD9)
021.1 ENTERIC TULAREMIA (ICD9)
021.2 PULMONARY TULAREMIA (ICD9)
021.3 OCULOGLANDULAR TULAREMIA (ICD9)
021.8 TULAREMIA NEC (ICD9)
021.9 TULAREMIA NOS (ICD9)
022.0 CUTANEOUS ANTHRAX (ICD9)
022.1 PULMONARY ANTHRAX (ICD9)
022.2 GASTROINTESTINAL ANTHRAX (ICD9)
022.3 ANTHRAX SEPTICEMIA (ICD9)
022.8 OTHER ANTHRAX MANIFEST (ICD9)
022.9 ANTHRAX NOS (ICD9)
023.0 BRUCELLA MELITENSIS (ICD9)
023.1 BRUCELLA ABORTUS (ICD9)
023.2 BRUCELLA SUIS (ICD9)
023.3 BRUCELLA CANIS (ICD9)
023.8 BRUCELLOSIS NEC (ICD9)
023.9 BRUCELLOSIS NOS (ICD9)
024 GLANDERS (ICD9)
025 MELIOIDOSIS (ICD9)
026.0 SPIRILLARY FEVER (ICD9)
026.1 STREPTOBACILLARY FEVER (ICD9)
026.9 RAT-BITE FEVER NOS (ICD9)
027.0 LISTERIOSIS (ICD9)
027.1 ERYSIPELOTHRIX INFECTION (ICD9)
027.2 PASTEURELLOSIS (ICD9)
027.8 ZOONOTIC BACT DIS NEC (ICD9)

027.9 ZOONOTIC BACT DIS NOS (ICD9)
030.0 LEPROMATOUS LEPROSY (ICD9)
030.1 TUBERCULOID LEPROSY (ICD9)
030.2 INDETERMINATE LEPROSY (ICD9)
030.3 BORDERLINE LEPROSY (ICD9)
030.8 LEPROSY NEC (ICD9)
030.9 LEPROSY NOS (ICD9)
031.0 PULMONARY MYCOBACTERIA (ICD9)
031.1 CUTANEOUS MYCOBACTERIA (ICD9)
031.2 DX DUE TO DISSEM MYCOBACT (Begin 1997) (ICD9)
031.8 MYCOBACTERIAL DIS NEC (ICD9)
031.9 MYCOBACTERIAL DIS NOS (ICD9)
032.0 FAUCIAL DIPHTHERIA (ICD9)
032.1 NASOPHARYNX DIPHTHERIA (ICD9)
032.2 ANT NASAL DIPHTHERIA (ICD9)
032.3 LARYNGEAL DIPHTHERIA (ICD9)
032.81 CONJUNCTIVAL DIPHTHERIA (ICD9)
032.83 DIPHTHERITIC PERITONITIS (ICD9)
032.84 DIPHTHERITIC CYSTITIS (ICD9)
032.85 CUTANEOUS DIPHTHERIA (ICD9)
032.89 DIPHTHERIA NEC (ICD9)
032.9 DIPHTHERIA NOS (ICD9)
033.0 BORDETELLA PERTUSSIS (ICD9)
033.1 BORDETELLA PARAPERTUSSIS (ICD9)
033.8 WHOOPING COUGH NEC (ICD9)
033.9 WHOOPING COUGH NOS (ICD9)
034.0 STREP SORE THROAT (ICD9)
034.1 SCARLET FEVER (ICD9)
035 ERYSIPELAS (ICD9)
036.0 MENINGOCOCCAL MENINGITIS (ICD9)
036.1 MENINGOCOCC ENCEPHALITIS (ICD9)
036.2 MENINGOCOCCEMIA (ICD9)
036.3 MENINGOCOCC ADRENAL SYND (ICD9)
036.40 MENINGOCOCCAL CARDITIS UNSPECIFIED
036.41 MENINGOCOCCAL PERICARDITIS
036.42 MENINGOCOCCAL ENDOCARDITIS
036.43 MENINGOCOCCAL MYOCARDITIS
036.81 MENINGOCOCC OPTIC NEURIT (ICD9)
036.82 MENINGOCOCC ARTHROPATHY (ICD9)
036.89 MENINGOCOCCAL INFECT NEC (ICD9)
036.9 MENINGOCOCCAL INFECT NOS (ICD9)
037 TETANUS (ICD9)
038.0 STREPTOCOCCAL SEPTICEMIA (ICD9)
038.1 STAPHYLOCOCC SEPTICEMIA (End 1997) (ICD9)
038.10 STAPH SEPTICEMIA- UNSPEC (Begin 1997) (ICD9)
038.11 STAPH AUREUS SEPTICEMIA (Begin 1997) (ICD9)
038.12 (no description found)
038.19 OT STAPH SEPTICEMIA (Begin 1997) (ICD9)
038.2 PNEUMOCOCCAL SEPTICEMIA (ICD9)
038.3 ANAEROBIC SEPTICEMIA (ICD9)
038.40 GRAM-NEG SEPTICEMIA NOS (ICD9)
038.41 H. INFLUENAE SEPTICEMIA (ICD9)
038.42 E COLI SEPTICEMIA (ICD9)
038.43 PSEUDOMONAS SEPTICEMIA (ICD9)
038.44 SERRATIA SEPTICEMIA (ICD9)
038.49 GRAM-NEG SEPTICEMIA NEC (ICD9)
038.8 SEPTICEMIA NEC (ICD9)
038.9 SEPTICEMIA NOS (ICD9)
039.0 CUTANEOUS ACTINOMYCOSIS (ICD9)
039.1 PULMONARY ACTINOMYCOSIS (ICD9)

039.2 ABDOMINAL ACTINOMYCOSIS (ICD9)
039.3 CERVICOFAC ACTINOMYCOSIS (ICD9)
039.4 MADURA FOOT (ICD9)
039.8 ACTINOMYCOSIS NEC (ICD9)
039.9 ACTINOMYCOSIS NOS (ICD9)
040.0 GAS GANGRENE (ICD9)
040.1 RHINOSCLEROMA (ICD9)
040.2 WHIPPLE-S DISEASE (ICD9)
040.3 NECROBACILLOSIS (ICD9)
040.41 INFANT BOTULISM (ICD9)
040.42 WOUND BOTULISM (Begin 2007) (ICD9)
040.81 TROPICAL PYOMYOSITIS (ICD9)
040.82 TOXIC SHOCK SYNDROME (Begin 2002) (ICD9)
040.89 BACTERIAL DISEASES NEC (ICD9)
041.0 STREPTOCOCCUS INFECT NOS (Begin 1980 (ICD9)
041.00 STREPTOCOCCUS UNSPEC (Begin 1992) (ICD9)
041.01 GROUP A STREPTOCOCCUS (Begin 1992) (ICD9)
041.02 GROUP B STREPTOCOCCUS (Begin 1992) (ICD9)
041.03 GROUP C STREPTOCOCCUS (Begin 1992) (ICD9)
041.04 GROUP D STREPTOCOCCUS (Begin 1992) (ICD9)
041.05 GROUP G STREPTOCOCCUS (Begin 1992) (ICD9)
041.09 OTHER STREPTOCOCCUS (Begin 1992) (ICD9)
041.1 STAPH INFECTION NOS (Begin 1980 (ICD9)
041.10 STAPH UNSPEC (Begin 1992) (ICD9)
041.11 STAPH AUREUS (Begin 1992) (ICD9)
041.12 (no description found)
041.19 OTHER STAPH (Begin 1992) (ICD9)
041.2 PNEUMOCOCCUS INFECT NOS (ICD9)
041.3 KLEBSIELLA INFECT NOS (ICD9)
041.4 E. COLI INFECT NOS (ICD9)
041.5 H. INFLUENZAE INFECT NOS (ICD9)
041.6 PROTEUS INFECTION NOS (ICD9)
041.7 PSEUDOMONAS INFECT NOS (ICD9)
041.8 BACTERIAL INFECTION NEC (Begin 1980 (ICD9)
041.81 MYCOPLASMA (Begin 1992) (ICD9)
041.82 BACILLUS FRAGILIS (Begin 1992) (ICD9)
041.83 CLOSTRID PERFRINGENS (Begin 1992) (ICD9)
041.84 OTHER ANAEROBES (Begin 1992) (ICD9)
041.85 OTHER GRAM NEG ORGS (Begin 1992) (ICD9)
041.86 HELICOBACTER PYLORI INFECTION (Begin 1995) (ICD9)
041.89 OTHER SPEC BACTERIA (Begin 1992) (ICD9)
041.9 BACTERIAL INFECTION NOS (ICD9)
042 HIV DISEASE (Begin 1994) (ICD9)
042.0 HIV W/SPECIF INFECTIONS (Begin 1986 (ICD9)
042.1 HIV CAUS OTH SPEC INFECT (Begin 1986 (ICD9)
042.2 HIV W/SPEC MALIG NEOPLSM (Begin 1986 (ICD9)
042.9 AIDS- UNSPECIFIED (Begin 1986 (ICD9)
043.0 HIV CAUS LYMPHADENOPATHY (Begin 1986 (ICD9)
043.1 HIV CAUS SP CNS DISEASE (Begin 1986 (ICD9)
043.2 HIV CAUS OT DISOR IMMUNE (Begin 1986 (ICD9)
043.3 HIV CAUS OTH SPECIF COND (Begin 1986 (ICD9)
043.9 ARC- UNSPECIFIED (Begin 1986 (ICD9)
044.0 HIV CAUS ACUTE INFECTION (Begin 1986 (ICD9)
044.9 HIV- UNSPECIFIED (Begin 1986 (ICD9)
045.00 AC BULBAR POLIO-TYPE NOS (ICD9)
045.01 AC BULBAR POLIO-TYPE 1 (ICD9)
045.02 AC BULBAR POLIO-TYPE 2 (ICD9)
045.03 AC BULBAR POLIO-TYPE 3 (ICD9)
045.10 PARAL POLIO NEC-TYPE NOS (ICD9)
045.11 PARAL POLIO NEC-TYPE 1 (ICD9)

045.12 PARAL POLIO NEC-TYPE 2 (ICD9)
045.13 PARAL POLIO NEC-TYPE 3 (ICD9)
045.20 NONPARALY POLIO-TYPE NOS (ICD9)
045.21 NONPARALYT POLIO-TYPE 1 (ICD9)
045.22 NONPARALYT POLIO-TYPE 2 (ICD9)
045.23 NONPARALYT POLIO-TYPE 3 (ICD9)
045.90 AC POLIO NOS-TYPE NOS (ICD9)
045.91 AC POLIO NOS-TYPE 1 (ICD9)
045.92 AC POLIO NOS-TYPE 2 (ICD9)
045.93 AC POLIO NOS-TYPE 3 (ICD9)
046.0 KURU (ICD9)
046.1 JAKOB-CREUTZFELDT DIS (ICD9)
046.11 (no description found)
046.19 (no description found)
046.3 PROG MULTIFOC LEUKOENCEP (ICD9)
046.71 (no description found)
046.72 (no description found)
046.79 (no description found)
046.8 CNS SLOW VIRUS INFEC NEC (ICD9)
046.9 CNS SLOW VIRUS INFEC NOS (ICD9)
047.0 COXSACKIE VIRUS MENING (ICD9)
047.1 ECHO VIRUS MENINGITIS (ICD9)
047.8 VIRAL MENINGITIS NEC (ICD9)
047.9 VIRAL MENINGITIS NOS (ICD9)
048 OTH ENTEROVIRAL CNS DIS (ICD9)
049.0 LYMPHOCYTIC CHORIOMENING (ICD9)
049.1 ADENOVIRAL MENINGITIS (ICD9)
049.8 VIRAL ENCEPHALITIS NEC (ICD9)
049.9 VIRAL ENCEPHALITIS NOS (ICD9)
050.0 VARIOLA MAJOR (ICD9)
050.1 ALASTRIM (ICD9)
050.2 MODIFIED SMALLPOX (ICD9)
050.9 SMALLPOX NOS (ICD9)
051.0 COWPOX (ICD9)
051.01 (no description found)
051.02 (no description found)
051.1 PSEUDOCOWPOX (ICD9)
051.2 CONTAGIOUS PUSTULAR DERM (ICD9)
051.9 PARAVACCINIA NOS (ICD9)
052.0 POSTVARICELLA ENCEPHALIT (ICD9)
052.1 VARICELLA PNEUMONITIS (ICD9)
052.2 POSTVARICELLA MYELITIS (Begin 2006) (ICD9)
052.7 VARICELLA COMPLICAT NEC (ICD9)
052.8 VARICELLA COMPLICAT NOS (ICD9)
052.9 VARICELLA UNCOMPLICATED (ICD9)
053.0 HERPES ZOSTER MENINGITIS (ICD9)
053.10 H ZOSTER NERV SYST NOS (ICD9)
053.11 GENICULATE HERPES ZOSTER (ICD9)
053.12 POSTHERPES TRIGEM NEURAL (ICD9)
053.13 POSTHERPES POLYNEUROPATH (ICD9)
053.14 HERPES ZOSTER MYELITIS (Begin 2006) (ICD9)
053.19 H ZOSTER NERV SYST NEC (ICD9)
053.20 HERPES ZOSTER OF EYELID (ICD9)
053.21 H ZOSTER KERATOCONJUNCT (ICD9)
053.22 H ZOSTER IRIDOCYCLITIS (ICD9)
053.29 HERPES ZOSTER OF EYE NEC (ICD9)
053.71 H ZOSTER OTITIS EXTERNA (ICD9)
053.79 H ZOSTER COMPLICATED NEC (ICD9)
053.8 H ZOSTER COMPLICATED NOS (ICD9)
053.9 HERPES ZOSTER NOS (ICD9)

054.0 ECZEMA HERPETICUM (ICD9)
054.10 GENITAL HERPES NOS (ICD9)
054.11 HERPETIC VULVOVAGINITIS (ICD9)
054.12 HERPETIC ULCER OF VULVA (ICD9)
054.13 HERPETIC INFECT OF PENIS (ICD9)
054.19 GENITAL HERPES NEC (ICD9)
054.2 HERPETIC GINGIVOSTOMAT (ICD9)
054.3 HERPETIC ENCEPHALITIS (ICD9)
054.40 HERPES SIMPLEX EYE NOS (ICD9)
054.41 HERPES SIMPLEX OF EYELID (ICD9)
054.42 DENDRITIC KERATITIS (ICD9)
054.43 H SIMPLEX KERATITIS (ICD9)
054.44 H SIMPLEX IRIDOCYCLITIS (ICD9)
054.49 HERPES SIMPLEX EYE NEC (ICD9)
054.5 HERPETIC SEPTICEMIA (ICD9)
054.6 HERPETIC WHITLOW (ICD9)
054.71 VISCERAL HERPES SIMPLEX (ICD9)
054.72 H SIMPLEX MENINGITIS (ICD9)
054.73 H SIMPLEX OTITIS EXTERNA (ICD9)
054.74 HERPES SIMPLEX MYELITIS (Begin 2006) (ICD9)
054.79 H SIMPLEX COMPLICAT NEC (ICD9)
054.8 H SIMPLEX COMPLICAT NOS (ICD9)
054.9 HERPES SIMPLEX NOS (ICD9)
055.0 POSTMEASLES ENCEPHALITIS (ICD9)
055.1 POSTMEASLES PNEUMONIA (ICD9)
055.2 POSTMEASLES OTITIS MEDIA (ICD9)
055.71 MEASLES KERATITIS (ICD9)
055.79 MEASLES COMPLICATION NEC (ICD9)
055.8 MEASLES COMPLICATION NOS (ICD9)
055.9 MEASLES UNCOMPLICATED (ICD9)
056.00 RUBELLA NERVE COMPL NOS (ICD9)
056.01 RUBELLA ENCEPHALITIS (ICD9)
056.09 RUBELLA NERVE COMPL NEC (ICD9)
056.71 ARTHRITIS DUE TO RUBELLA (ICD9)
056.79 RUBELLA COMPLICATION NEC (ICD9)
056.8 RUBELLA COMPLICATION NOS (ICD9)
056.9 RUBELLA UNCOMPLICATED (ICD9)
057.0 ERYTHEMA INFECTIOSUM (ICD9)
057.8 VIRAL EXANTHEMATA NEC (ICD9)
057.9 VIRAL EXANTHEMATA NOS (ICD9)
058.10 ROSEOLA INFANTUM NOS (Begin 2007) (ICD9)
058.11 ROSEOLA INFANT D/T HHV-6 (Begin 2007) (ICD9)
058.12 ROSEOLA INFANT D/T HHV-7 (Begin 2007) (ICD9)
058.21 HUMAN HERPESVIR 6 ENCEPH (Begin 2007) (ICD9)
058.29 HUMAN HERPESVR ENCPH NEC (Begin 2007) (ICD9)
058.81 HUMAN HERPESVIRUS 6 INFC (Begin 2007) (ICD9)
058.82 HUMAN HERPESVIRUS 7 INFC (Begin 2007) (ICD9)
058.89 HUMAN HERPESVIRS INF NEC (Begin 2007) (ICD9)
059.00 ORTHOPOXVIRUS INFECTION, UNSPECIFIED
059.01 MONKEYPOX
059.09 OTHER ORTHOPOXVIRUS INFECTIONS
059.10 PARAPOXVIRUS INFECTION, UNSPECIFIED
059.11 BOVINE STOMATITIS
059.12 SEALPOX
059.19 OTHER PARAPOXVIRUS INFECTIONS
059.20 YATAPOXVIRUS INFECTION, UNSPECIFIED
059.21 TANAPOX
059.22 YABA MONKEY TUMOR VIRUS
059.8 OTHER POXVIRUS INFECTIONS
059.9 POXVIRUS INFECTIONS, UNSPECIFIED

060.0 SYLVATIC YELLOW FEVER (ICD9)
060.1 URBAN YELLOW FEVER (ICD9)
060.9 YELLOW FEVER NOS (ICD9)
061 DENGUE (ICD9)
062.0 JAPANESE ENCEPHALITIS (ICD9)
062.1 WEST EQUINE ENCEPHALITIS (ICD9)
062.2 EAST EQUINE ENCEPHALITIS (ICD9)
062.3 ST LOUIS ENCEPHALITIS (ICD9)
062.4 AUSTRALIAN ENCEPHALITIS (ICD9)
062.5 CALIFORNIA ENCEPHALITIS (ICD9)
062.8 MOSQUIT-BORNE ENCEPH NEC (ICD9)
062.9 MOSQUIT-BORNE ENCEPH NOS (ICD9)
063.0 RUSSIA SPR-SUMMER ENCEPH (ICD9)
063.1 LOUPING ILL (ICD9)
063.2 CENT EUROPE ENCEPHALITIS (ICD9)
063.8 TICK-BORNE ENCEPH NEC (ICD9)
063.9 TICK-BORNE ENCEPH NOS (ICD9)
064 VIR ENCEPH ARTHROPOD NEC (ICD9)
065.0 CRIMEAN HEMORRHAGIC FEV (ICD9)
065.1 OMSK HEMORRHAGIC FEVER (ICD9)
065.2 KYASANUR FOREST DISEASE (ICD9)
065.3 TICK-BORNE HEM FEVER NEC (ICD9)
065.4 MOSQUITO-BORNE HEM FEVER (ICD9)
065.8 ARTHROPOD HEM FEVER NEC (ICD9)
065.9 ARTHROPOD HEM FEVER NOS (ICD9)
066.0 PHLEBOTOMUS FEVER (ICD9)
066.1 TICK-BORNE FEVER (ICD9)
066.2 VENEZUELAN EQUINE FEVER (ICD9)
066.3 MOSQUITO-BORNE FEVER NEC (ICD9)
066.4 WEST NILE FEVER (Begin 2002 (ICD9)
066.40 WEST NILE FEVER NOS (Begin 2004) (ICD9)
066.41 WEST NILE FEVER W/ENCEPH (Begin 2004) (ICD9)
066.42 WEST NILE NEURO MAN NEC (Begin 2004) (ICD9)
066.49 WEST NILE W COMPLIC NEC (Begin 2004) (ICD9)
066.8 ARTHROPOD VIRUS NEC (ICD9)
066.9 ARTHROPOD VIRUS NOS (ICD9)
070.0 HEPATITIS A WITH COMA (ICD9)
070.1 HEPATITIS A W/O COMA (ICD9)
070.2 HEPATITIS B WITH COMA (Begin 1980 (ICD9)
070.20 VRL HEPAT B CM W/O DELTA (Begin 1991) (ICD9)
070.21 VRL HEPAT B CM W DELTA (Begin 1991) (ICD9)
070.22 CHR HEPAT COMA W/O DELTA (Begin 1994) (ICD9)
070.23 CHR HEPAT COMA W/ DELTA (Begin 1994) (ICD9)
070.3 HEPATITIS B W/O COMA (Begin 1980 (ICD9)
070.30 VRL HPT B W/O CM W/O DLT (Begin 1991) (ICD9)
070.31 VRL HPT B W/O CM W DELTA (Begin 1991) (ICD9)
070.32 CHR HEPAT W/O COMA W/O DELTA (Begin 1994) (ICD9)
070.33 CHR HEPAT W/O COMA W/ DELTA (Begin 1994) (ICD9)
070.4 VIRAL HEPAT NEC W COMA (Begin 1980 (ICD9)
070.41 SPF VRL HPT CM HPT C (Begin 1991) (ICD9)
070.42 SPF VRL HPT CM DLT W/O B (Begin 1991) (ICD9)
070.43 SPF VRL HPT CM HPT E (Begin 1991) (ICD9)
070.44 CHR HEPAT C W/ COMA (Begin 1994) (ICD9)
070.49 SPF VRL HPT CM (Begin 1991) (ICD9)
070.5 VIRAL HEPAT NEC W/O COMA (Begin 1980 (ICD9)
070.51 VRL HPT W/O CM HEPAT C (Begin 1991) (ICD9)
070.52 VRL HPT W/O CM DLT W/O B (Begin 1991) (ICD9)
070.53 VRL HPT W/O CM HEPAT E (Begin 1991) (ICD9)
070.54 CHR HEPAT C W/O COMA (Begin 1994) (ICD9)
070.59 VRL HPT W/O CM (Begin 1991) (ICD9)

070.6 VIRAL HEPAT NOS W COMA (ICD9)
070.70 HPT C W/O HEPAT COMA NOS (Begin 2004) (ICD9)
070.71 HPT C W HEPATIC COMA NOS (Begin 2004) (ICD9)
070.9 VIRAL HEPAT NOS W/O COMA (ICD9)
071 RABIES (ICD9)
072.0 MUMPS ORCHITIS (ICD9)
072.1 MUMPS MENINGITIS (ICD9)
072.2 MUMPS ENCEPHALITIS (ICD9)
072.3 MUMPS PANCREATITIS (ICD9)
072.71 MUMPS HEPATITIS (ICD9)
072.72 MUMPS POLYNEUROPATHY (ICD9)
072.79 MUMPS COMPLICATION NEC (ICD9)
072.8 MUMPS COMPLICATION NOS (ICD9)
072.9 MUMPS UNCOMPLICATED (ICD9)
073.0 ORNITHOSIS PNEUMONIA (ICD9)
073.7 ORNITHOSIS COMPLICAT NEC (ICD9)
073.8 ORNITHOSIS COMPLICAT NOS (ICD9)
073.9 ORNITHOSIS NOS (ICD9)
074.0 HERPANGINA (ICD9)
074.1 EPIDEMIC PLEURODYNIA (ICD9)
074.20 COXSACKIE CARDITIS UNSPECIFIED
074.21 COXSACKIE PERICARDITIS
074.22 COXSACKIE ENDOCARDITIS
074.23 COXSACKIE MYOCARDTIS
074.3 HAND- FOOT & MOUTH DIS (ICD9)
074.8 COXSACKIE VIRUS NEC (ICD9)
075 INFECTIOUS MONONUCLEOSIS (ICD9)
076.0 TRACHOMA- INITIAL STAGE (ICD9)
076.1 TRACHOMA- ACTIVE STAGE (ICD9)
076.9 TRACHOMA NOS (ICD9)
077.0 INCLUSION CONJUNCTIVITIS (ICD9)
077.1 EPIDEM KERATOCONJUNCTIV (ICD9)
077.2 PHARYNGOCONJUNCT FEVER (ICD9)
077.3 ADENOVIRAL CONJUNCT NEC (ICD9)
077.4 EPIDEM HEM CONJUNCTIVIT (ICD9)
077.8 VIRAL CONJUNCTIVITIS NEC (ICD9)
077.9 VIRAL AND CHLAMYDIAL CONJUNCTIVITIS NOS (End 1993) (ICD9)
077.98 DIS OF CONJUNCT DUE TO CHLAMYDIAE (Begin 1993) (ICD9)
077.99 DIS OF CONJUNCT DUE TO VIRUSES (Begin 1993) (ICD9)
078.0 MOLLUSCUM CONTAGIOSUM (ICD9)
078.1 VIRAL WARTS (End 1993) (ICD9)
078.10 VIRAL WARTS UNSPEC (Begin 1993) (ICD9)
078.11 CONDYLOMA ACCUMINATUM (Begin 1993) (ICD9)
078.12 (no description found)
078.19 OTHER SPEC VIRAL WARTS (Begin 1993) (ICD9)
078.2 SWEATING FEVER (ICD9)
078.3 CAT-SCRATCH DISEASE (ICD9)
078.4 FOOT & MOUTH DISEASE (ICD9)
078.5 CYTOMEGAL INCLUSION DIS (ICD9)
078.6 HEM NEPHROSONEPHRITIS (ICD9)
078.7 ARENAVIRAL HEM FEVER (ICD9)
078.81 EPIDEMIC VERTIGO (ICD9)
078.82 EPIDEMIC VOMITING SYND (ICD9)
078.88 OTH SPEC DISEASE DUE TO CHLAMYDIAE (Begin 1993) (ICD9)
078.89 VIRAL DISEASE NEC (ICD9)
079.0 ADENOVIRUS INFECT NOS (ICD9)
079.1 ECHO VIRUS INFECT NOS (ICD9)
079.2 COXSACKIE VIRUS INF NOS (ICD9)
079.3 RHINOVIRUS INFECT NOS (ICD9)
079.4 HUMAN PAPILLOMA VIRUS (Begin 1993) (ICD9)

079.50 UNSPEC RETROVIRUS (Begin 1993) (ICD9)
079.51 HTLV TYPE I (Begin 1993) (ICD9)
079.52 HTLV TYPE II (Begin 1993) (ICD9)
079.53 HIV TYPE 2 (Begin 1993) (ICD9)
079.59 OTH SPEC RETROVIRUS (Begin 1993) (ICD9)
079.6 RESPIR SYNCITIAL VIRUS (Begin 1996) (ICD9)
079.8 VIRAL AND CHLAMYDIAL INFECTION NEC (End 1993) (ICD9)
079.81 HANTAVIRUS INFECTION (Begin 1995) (ICD9)
079.82 SARS-ASSOCIATED CORONAVIRUS (Begin 2003) (ICD9)
079.83 PARVOVIRUS B19 (Begin 2007) (ICD9)
079.88 OTH SPEC CHLAMYDIAL INFECTION (Begin 1993) (ICD9)
079.89 OTH SPEC VIRAL INFECTION (Begin 1993) (ICD9)
079.9 VIRAL AND CHLAMYDIAL INFECTION NOS (End 1993) (ICD9)
079.98 CHLAMYDIAL INFECTION NOS (Begin 1993) (ICD9)
079.99 VIRAL INFECTION NOS (Begin 1993) (ICD9)
080 LOUSE-BORNE TYPHUS (ICD9)
081.0 MURINE TYPHUS (ICD9)
081.1 BRILL-S DISEASE (ICD9)
081.2 SCRUB TYPHUS (ICD9)
081.9 TYPHUS NOS (ICD9)
082.0 SPOTTED FEVERS (ICD9)
082.1 BOUTONNEUSE FEVER (ICD9)
082.2 NORTH ASIAN TICK FEVER (ICD9)
082.3 QUEENSLAND TICK TYPHUS (ICD9)
082.40 EHRLICHIOSIS- UNSPECIFIED (Begin 2000) (ICD9)
082.41 EHRLICHIOSIS CHAFEENSIS (Begin 2000) (ICD9)
082.49 OTHER EHRLICHIOSIS (Begin 2000) (ICD9)
082.8 TICK-BORNE RICKETTS NEC (ICD9)
082.9 TICK-BORNE RICKETTS NOS (ICD9)
083.0 Q FEVER (ICD9)
083.1 TRENCH FEVER (ICD9)
083.2 RICKETTSIALPOX (ICD9)
083.8 RICKETTSIOSES NEC (ICD9)
083.9 RICKETTSIOSIS NOS (ICD9)
084.0 FALCIPARUM MALARIA (ICD9)
084.1 VIVAX MALARIA (ICD9)
084.2 QUARTAN MALARIA (ICD9)
084.3 OVALE MALARIA (ICD9)
084.4 MALARIA NEC (ICD9)
084.5 MIXED MALARIA (ICD9)
084.6 MALARIA NOS (ICD9)
084.7 INDUCED MALARIA (ICD9)
084.8 BLACKWATER FEVER (ICD9)
084.9 MALARIA COMPLICATED NEC (ICD9)
085.0 VISCERAL LEISHMANIASIS (ICD9)
085.1 CUTAN LEISHMANIAS URBAN (ICD9)
085.2 CUTAN LEISHMANIAS ASIAN (ICD9)
085.3 CUTAN LEISHMANIAS ETHIOP (ICD9)
085.4 CUTAN LEISHMANIAS AMER (ICD9)
085.5 MUCOCUTAN LEISHMANIASIS (ICD9)
085.9 LEISHMANIASIS NOS (ICD9)
086.0 CHAGAS DISEASE OF HEART (ICD9)
086.1 CHAGAS DIS OF OTH ORGAN (ICD9)
086.2 CHAGAS DISEASE NOS (ICD9)
086.3 GAMBIAN TRYPANOSOMIASIS (ICD9)
086.4 RHODESIAN TRYPANOSOMIAS (ICD9)
086.5 AFRICAN TRYPANOSOMA NOS (ICD9)
086.9 TRYPANOSOMIASIS NOS (ICD9)
087.0 LOUSE-BORNE RELAPS FEVER (ICD9)
087.1 TICK-BORNE RELAPS FEVER (ICD9)

087.9 RELAPSING FEVER NOS (ICD9)
088.0 BARTONELLOSIS (ICD9)
088.8 ARTHROPOD-BORNE DIS NEC (Begin 1980 (ICD9)
088.81 LYME DISEASE (Begin 1989) (ICD9)
088.82 BABESIOSIS (Begin 1993) (ICD9)
088.89 OTH ARTHROPOD-BORNE DIS (Begin 1989) (ICD9)
088.9 ARTHROPOD-BORNE DIS NOS (ICD9)
090.0 EARLY CONG SYPH SYMPTOM (ICD9)
090.1 EARLY CONGEN SYPH LATENT (ICD9)
090.2 EARLY CONGEN SYPH NOS (ICD9)
090.3 SYPHILITIC KERATITIS (ICD9)
090.40 JUVENILE NEUROSYPH NOS (ICD9)
090.41 CONGEN SYPH ENCEPHALITIS (ICD9)
090.42 CONGEN SYPH MENINGITIS (ICD9)
090.49 JUVENILE NEUROSYPH NEC (ICD9)
090.5 LATE CONGEN SYPH SYMPTOM (ICD9)
090.6 LATE CONGEN SYPH LATENT (ICD9)
090.7 LATE CONGEN SYPH NOS (ICD9)
090.9 CONGENITAL SYPHILIS NOS (ICD9)
091.0 PRIMARY GENITAL SYPHILIS (ICD9)
091.1 PRIMARY ANAL SYPHILIS (ICD9)
091.2 PRIMARY SYPHILIS NEC (ICD9)
091.3 SECONDARY SYPH SKIN (ICD9)
091.4 SYPHILITIC ADENOPATHY (ICD9)
091.50 SYPHILITIC UVEITIS NOS (ICD9)
091.51 SYPHILIT CHORIORETINITIS (ICD9)
091.52 SYPHILITIC IRIDOCYCLITIS (ICD9)
091.61 SYPHILITIC PERIOSTITIS (ICD9)
091.62 SYPHILITIC HEPATITIS (ICD9)
091.69 SECOND SYPH VISCERA NEC (ICD9)
091.7 SECOND SYPHILIS RELAPSE (ICD9)
091.81 ACUTE SYPHIL MENINGITIS (ICD9)
091.82 SYPHILITIC ALOPECIA (ICD9)
091.89 SECONDARY SYPHILIS NEC (ICD9)
091.9 SECONDARY SYPHILIS NOS (ICD9)
092.0 EARLY SYPH LATENT RELAPS (ICD9)
092.9 EARLY SYPHIL LATENT NOS (ICD9)
093.0 AORTIC ANEURYSM- SYPHIL (ICD9)
093.1 SYPHILITIC AORTITIS (ICD9)
093.20 SYPHIL ENDOCARDITIS NOS (ICD9)
093.21 SYPHILITIC MITRAL VALVE (ICD9)
093.22 SYPHILITIC AORTIC VALVE (ICD9)
093.23 SYPHIL TRICUSPID VALVE (ICD9)
093.24 SYPHIL PULMONARY VALVE (ICD9)
093.81 SYPHILITIC PERICARDITIS (ICD9)
093.82 SYPHILITIC MYOCARDITIS (ICD9)
093.89 CARDIOVASCULAR SYPH NEC (ICD9)
093.9 CARDIOVASCULAR SYPH NOS (ICD9)
094.0 TABES DORSALIS (ICD9)
094.1 GENERAL PARESIS (ICD9)
094.2 SYPHILITIC MENINGITIS (ICD9)
094.3 ASYMPTOMAT NEUROSYPHILIS (ICD9)
094.81 SYPHILITIC ENCEPHALITIS (ICD9)
094.82 SYPHILITIC PARKINSONISM (ICD9)
094.83 SYPH DISSEM RETINITIS (ICD9)
094.84 SYPHILITIC OPTIC ATROPHY (ICD9)
094.85 SYPH RETROBULB NEURITIS (ICD9)
094.86 SYPHIL ACOUSTIC NEURITIS (ICD9)
094.87 SYPH RUPT CEREB ANEURYSM (ICD9)
094.89 NEUROSYPHILIS NEC (ICD9)

094.9	NEUROSYPHILIS NOS (ICD9)
095.0	SYPHILITIC EPISCLERITIS (ICD9)
095.1	SYPHILIS OF LUNG (ICD9)
095.2	SYPHILITIC PERITONITIS (ICD9)
095.3	SYPHILIS OF LIVER (ICD9)
095.4	SYPHILIS OF KIDNEY (ICD9)
095.5	SYPHILIS OF BONE (ICD9)
095.6	SYPHILIS OF MUSCLE (ICD9)
095.7	SYPHILIS OF TENDON/BURSA (ICD9)
095.8	LATE SYMPT SYPHILIS NEC (ICD9)
095.9	LATE SYMPT SYPHILIS NOS (ICD9)
096	LATE SYPHILIS LATENT (ICD9)
097.0	LATE SYPHILIS NOS (ICD9)
097.1	LATENT SYPHILIS NOS (ICD9)
097.9	SYPHILIS NOS (ICD9)
098.0	ACUTE GC INFECT LOWER GU (ICD9)
098.10	GC (ACUTE) UPPER GU NOS (ICD9)
098.11	GC CYSTITIS (ACUTE) (ICD9)
098.12	GC PROSTATITIS (ACUTE) (ICD9)
098.13	GC ORCHITIS (ACUTE) (ICD9)
098.14	GC SEM VESICULIT (ACUTE) (ICD9)
098.15	GC CERVICITIS (ACUTE) (ICD9)
098.16	GC ENDOMETRITIS (ACUTE) (ICD9)
098.17	ACUTE GC SALPINGITIS (ICD9)
098.19	GC (ACUTE) UPPER GU NEC (ICD9)
098.2	CHR GC INFECT LOWER GU (ICD9)
098.30	CHR GC UPPER GU NOS (ICD9)
098.31	GC CYSTITIS- CHRONIC (ICD9)
098.32	GC PROSTATITIS- CHRONIC (ICD9)
098.33	GC ORCHITIS- CHRONIC (ICD9)
098.34	GC SEM VESICULITIS- CHR (ICD9)
098.35	GC CERVICITIS- CHRONIC (ICD9)
098.36	GC ENDOMETRITIS- CHRONIC (ICD9)
098.37	GC SALPINGITIS (CHRONIC) (ICD9)
098.39	CHR GC UPPER GU NEC (ICD9)
098.40	GONOCOCCAL CONJUNCTIVIT (ICD9)
098.41	GONOCOCCAL IRIDOCYCLITIS (ICD9)
098.42	GONOCOCCAL ENDOPHTHALMIA (ICD9)
098.43	GONOCOCCAL KERATITIS (ICD9)
098.49	GONOCOCCAL EYE NEC (ICD9)
098.50	GONOCOCCAL ARTHRITIS (ICD9)
098.51	GONOCOCCAL SYNOVITIS (ICD9)
098.52	GONOCOCCAL BURSITIS (ICD9)
098.53	GONOCOCCAL SPONDYLITIS (ICD9)
098.59	GC INFECT JOINT NEC (ICD9)
098.6	GONOCOCCAL INFEC PHARYNX (ICD9)
098.7	GC INFECT ANUS & RECTUM (ICD9)
098.81	GONOCOCCAL KERATOSIS (ICD9)
098.82	GONOCOCCAL MENINGITIS (ICD9)
098.83	GONOCOCCAL PERICARDITIS (ICD9)
098.84	GONOCOCCAL ENDOCARDITIS (ICD9)
098.85	GONOCOCCAL HEART DIS NEC (ICD9)
098.86	GONOCOCCAL PERITONITIS (ICD9)
098.89	GONOCOCCAL INF SITE NEC (ICD9)
099.0	CHANCROID (ICD9)
099.1	LYMPHOGRANULOMA VENEREUM (ICD9)
099.2	GRANULOMA INGUINALE (ICD9)
099.4	NONGONOCOCC URETHRIT NEC (Begin 1980 (ICD9)
099.40	UNSPEC URETHRITIS (Begin 1992) (ICD9)
099.41	CHLAMYDIA URETHRITIS (Begin 1992) (ICD9)

099.49 NONGONOCOCC URETHRIT NEC (Begin 1992) (ICD9)
099.50 CHLAMYDIA-UNSPEC SITE (Begin 1992) (ICD9)
099.51 CHLAMYDIA-PHARYNX (Begin 1992) (ICD9)
099.52 CHLAMYDIA-ANUS RECTUM (Begin 1992) (ICD9)
099.53 CHLAMYDIA-LOWER GU (Begin 1992) (ICD9)
099.54 CHLAMYDIA-OTHER GU (Begin 1992) (ICD9)
099.55 CHLAMYDIA-UNSPEC GU (Begin 1992) (ICD9)
099.56 CHLAMYDIA-PERITONEUM (Begin 1992) (ICD9)
099.59 CHLAMYDIA-NSC (Begin 1992) (ICD9)
099.8 VENEREAL DISEASE NEC (ICD9)
099.9 VENEREAL DISEASE NOS (ICD9)
100.0 LEPTOSPIROS ICTEROHEM (ICD9)
100.81 LEPTOSPIRAL MENINGITIS (ICD9)
100.89 LEPTOSPIRAL INFECT NEC (ICD9)
100.9 LEPTOSPIROSIS NOS (ICD9)
101 VINCENT-S ANGINA (ICD9)
102.0 INITIAL LESIONS YAWS (ICD9)
102.1 MULTIPLE PAPILLOMATA (ICD9)
102.2 EARLY SKIN YAWS NEC (ICD9)
102.3 HYPERKERATOSIS OF YAWS (ICD9)
102.4 GUMMATA AND ULCERS- YAWS (ICD9)
102.5 GANGOSA (ICD9)
102.6 YAWS OF BONE & JOINT (ICD9)
102.7 YAWS MANIFESTATIONS NEC (ICD9)
102.8 LATENT YAWS (ICD9)
102.9 YAWS NOS (ICD9)
103.0 PINTA PRIMARY LESIONS (ICD9)
103.1 PINTA INTERMED LESIONS (ICD9)
103.2 LATE LESIONS OF PINTA
103.3 PINTA MIXED LESIONS (ICD9)
103.9 PINTA NOS (ICD9)
104.0 NONVENEREAL ENDEMIC SYPH (ICD9)
104.8 SPIROCHETAL INFECT NEC (ICD9)
104.9 SPIROCHETAL INFECT NOS (ICD9)
110.0 DERMATOPHYT SCALP/BEARD (ICD9)
110.1 DERMATOPHYTOSIS OF NAIL (ICD9)
110.2 DERMATOPHYTOSIS OF HAND (ICD9)
110.3 DERMATOPHYTOSIS OF GROIN (ICD9)
110.4 DERMATOPHYTOSIS OF FOOT (ICD9)
110.5 DERMATOPHYTOSIS OF BODY (ICD9)
110.6 DEEP DERMATOPHYTOSIS (ICD9)
110.8 DERMATOPHYTOSIS SITE NEC (ICD9)
110.9 DERMATOPHYTOSIS SITE NOS (ICD9)
111.0 PITYRIASIS VERSICOLOR (ICD9)
111.1 TINEA NIGRA (ICD9)
111.2 TINEA BLANCA (ICD9)
111.3 BLACK PIEDRA (ICD9)
111.8 DERMATOMYCOSES NEC (ICD9)
111.9 DERMATOMYCOSIS NOS (ICD9)
112.0 THRUSH (ICD9)
112.1 CANDIDAL VULVOVAGINITIS (ICD9)
112.2 CANDIDIAS UROGENITAL NEC (ICD9)
112.3 CUTANEOUS CANDIDIASIS (ICD9)
112.4 CANDIDIASIS OF LUNG (ICD9)
112.5 DISSEMINATED CANDIDIASIS (ICD9)
112.81 CANDIDAL ENDOCARDITIS
112.82 CANDIDAL OTITIS EXTERNA (ICD9)
112.83 CANDIDAL MENINGITIS (ICD9)
112.84 CANDIDAL ESOPHAGITIS (Begin 1992) (ICD9)
112.85 CANDIDAL ENTERITIS (Begin 1992) (ICD9)

112.89	CANDIDIASIS SITE NEC (ICD9)
112.9	CANDIDIASIS SITE NOS (ICD9)
114.0	PRIMARY COCCIDIOIDOMYCOS (ICD9)
114.1	PRIM CUTAN COCCIDIOID (ICD9)
114.2	COCCIDIOIDAL MENINGITIS (ICD9)
114.3	PROGRESS COCCIDIOID NEC (ICD9)
114.4	CHRONIC PULMON COCCIDIOIDOMYCOSIS (Begin 1993) (ICD9)
114.5	UNSPEC PULMON COCCIDIOIDOMYCOSIS (Begin 1993) (ICD9)
114.9	COCCIDIOIDOMYCOSIS NOS (ICD9)
115.00	HISTOPLASMA CAPSULAT NOS (ICD9)
115.01	HISTOPLASM CAPSUL MENING (ICD9)
115.02	HISTOPLASM CAPSUL RETINA (ICD9)
115.05	HISTOPLASM CAPS PNEUMON (ICD9)
115.09	HISTOPLASMA CAPSULAT NEC (ICD9)
115.10	HISTOPLASMA DUBOISII NOS (ICD9)
115.11	HISTOPLASM DUBOIS MENING (ICD9)
115.12	HISTOPLASM DUBOIS RETINA (ICD9)
115.15	HISTOPLASM DUB PNEUMONIA (ICD9)
115.19	HISTOPLASMA DUBOISII NEC (ICD9)
115.90	HISTOPLASMOSIS NOS (ICD9)
115.91	HISTOPLASMOSIS MENINGIT (ICD9)
115.92	HISTOPLASMOSIS RETINITIS (ICD9)
115.95	HISTOPLASMOSIS PNEUMONIA (ICD9)
115.99	HISTOPLASMOSIS NEC (ICD9)
116.0	BLASTOMYCOSIS (ICD9)
116.1	PARACOCCIDIOIDOMYCOSIS (ICD9)
116.2	LOBOMYCOSIS (ICD9)
117.0	RHINOSPORIDIOSIS (ICD9)
117.1	SPOROTRICHOSIS (ICD9)
117.2	CHROMOBLASTOMYCOSIS (ICD9)
117.3	ASPERGILLOSIS (ICD9)
117.4	MYCOTIC MYCETOMAS (ICD9)
117.5	CRYPTOCOCCOSIS (ICD9)
117.6	ALLESCHERIOSIS (ICD9)
117.7	ZYGOMYCOSIS (ICD9)
117.8	DEMATIACIOUS FUNGI INF (ICD9)
117.9	MYCOSES NEC & NOS (ICD9)
118	OPPORTUNISTIC MYCOSES (ICD9)
120.0	SCHISTOSOMA HAEMATOBIUM (ICD9)
120.1	SCHISTOSOMA MANSONI (ICD9)
120.2	SCHISTOSOMA JAPONICUM (ICD9)
120.3	CUTANEOUS SCHISTOSOMA (ICD9)
120.8	SCHISTOSOMIASIS NEC (ICD9)
120.9	SCHISTOSOMIASIS NOS (ICD9)
121.0	OPISTHORCHIASIS (ICD9)
121.1	CLONORCHIASIS (ICD9)
121.2	PARAGONIMIASIS (ICD9)
121.3	FASCIOLIASIS (ICD9)
121.4	FASCIOLOPSIASIS (ICD9)
121.5	METAGONIMIASIS (ICD9)
121.6	HETEROPHYIASIS (ICD9)
121.8	TREMATODE INFECTION NEC (ICD9)
121.9	TREMATODE INFECTION NOS (ICD9)
122.0	ECHINOCOCC GRANUL LIVER (ICD9)
122.1	ECHINOCOCC GRANUL LUNG (ICD9)
122.2	ECHINOCOCC GRAN THYROID (ICD9)
122.3	ECHINOCOCC GRANUL NEC (ICD9)
122.4	ECHINOCOCC GRANUL NOS (ICD9)
122.5	ECHINOCOC MULTILOC LIVER (ICD9)
122.6	ECHINOCOCC MULTILOC NEC (ICD9)

122.7	ECHINOCOCC MULTILOC NOS (ICD9)
122.8	ECHINOCOCCOSIS NOS LIVER (ICD9)
122.9	ECHINOCOCCOSIS NEC/NOS (ICD9)
123.0	TAENIA SOLIUM INTESTINE (ICD9)
123.1	CYSTICERCOSIS (ICD9)
123.2	TAENIA SAGINATA INFECT (ICD9)
123.3	TAENIASIS NOS (ICD9)
123.4	DIPHYLLOBOTHRIAS INTEST (ICD9)
123.5	SPARGANOSIS (ICD9)
123.6	HYMENOLEPIASIS (ICD9)
123.8	CESTODE INFECTION NEC (ICD9)
123.9	CESTODE INFECTION NOS (ICD9)
124	TRICHINOSIS (ICD9)
125.0	BANCROFTIAN FILARIASIS (ICD9)
125.1	MALAYAN FILARIASIS (ICD9)
125.2	LOIASIS (ICD9)
125.3	ONCHOCERCIASIS (ICD9)
125.4	DIPETALONEMIASIS (ICD9)
125.5	MANSONELLA OZZARDI INFEC (ICD9)
125.6	FILARIASIS NEC (ICD9)
125.7	DRACONTIASIS (ICD9)
125.9	FILARIASIS NOS (ICD9)
126.0	ANCYLOSTOMA DUODENALE (ICD9)
126.1	NECATOR AMERICANUS (ICD9)
126.2	ANCYLOSTOMA BRAZILIENSE (ICD9)
126.3	ANCYLOSTOMA CEYLANICUM (ICD9)
126.8	ANCYLOSTOMA NEC (ICD9)
126.9	ANCYLOSTOMIASIS NOS (ICD9)
127.0	ASCARIASIS (ICD9)
127.1	ANISAKIASIS (ICD9)
127.2	STRONGYLOIDIASIS (ICD9)
127.3	TRICHURIASIS (ICD9)
127.4	ENTEROBIASIS (ICD9)
127.5	CAPILLARIASIS (ICD9)
127.6	TRICHOSTRONGYLIASIS (ICD9)
127.7	INTEST HELMINTHIASIS NEC (ICD9)
127.8	MIXED INTESTINE HELMINTH (ICD9)
127.9	INTEST HELMINTHIASIS NOS (ICD9)
128.0	TOXOCARIASIS (ICD9)
128.1	GNATHOSTOMIASIS (ICD9)
128.8	HELMINTHIASIS NEC (ICD9)
128.9	HELMINTHIASIS NOS (ICD9)
129	INTESTIN PARASITISM NOS (ICD9)
130.0	TOXOPLASM MENINGOENCEPH (ICD9)
130.1	TOXOPLASM CONJUNCTIVITIS (ICD9)
130.2	TOXOPLASM CHORIORETINIT (ICD9)
130.4	TOXOPLASMA PNEUMONITIS (ICD9)
130.5	TOXOPLASMA HEPATITIS (ICD9)
130.7	TOXOPLASMOSIS SITE NEC (ICD9)
130.8	MULTISYSTEM TOXOPLASMOS (ICD9)
130.9	TOXOPLASMOSIS NOS (ICD9)
131.00	UROGENITAL TRICHOMON NOS (ICD9)
131.01	TRICHOMONAL VAGINITIS (ICD9)
131.02	TRICHOMONAL URETHRITIS (ICD9)
131.03	TRICHOMONAL PROSTATITIS (ICD9)
131.09	UROGENITAL TRICHOMON NEC (ICD9)
131.8	TRICHOMONIASIS NEC (ICD9)
131.9	TRICHOMONIASIS NOS (ICD9)
132.0	PEDICULUS CAPITIS (ICD9)
132.1	PEDICULUS CORPORIS (ICD9)

132.2	PHTHIRUS PUBIS (ICD9)
132.3	MIXED PEDICUL & PHTHIRUS (ICD9)
132.9	PEDICULOSIS NOS (ICD9)
133.0	SCABIES (ICD9)
133.8	ACARIASIS NEC (ICD9)
133.9	ACARIASIS NOS (ICD9)
134.0	MYIASIS (ICD9)
134.1	ARTHROPOD INFEST NEC (ICD9)
134.2	HIRUDINIASIS (ICD9)
134.8	INFESTATION NEC (ICD9)
134.9	INFESTATION NOS (ICD9)
135	SARCOIDOSIS (ICD9)
136.0	AINHUM (ICD9)
136.2	FREE-LIVING AMEBA INFECT (ICD9)
136.21	SPECIFIC INFECTION DUE TO ACANTHAMOEBA
136.29	OTHER SPECIFIC INFECTIONS BY FREE-LIVING AMEBAE
136.3	PNEUMOCYSTOSIS (ICD9)
136.4	PSOROSPERMIASIS (ICD9)
136.5	SARCOSPORIDIOSIS (ICD9)
136.8	INFECT/PARASITE DIS NEC (ICD9)
136.9	INFECT/PARASITE DIS NOS (ICD9)
283.11	HEMOLYTIC-UREMIC SYNDROME
320.0	HEMOPHILUS MENINGITIS (ICD9)
320.1	PNEUMOCOCCAL MENINGITIS (ICD9)
320.2	STREPTOCOCCAL MENINGITIS (ICD9)
320.3	STAPHYLOCOCC MENINGITIS (ICD9)
320.7	MENING IN OTH BACT DIS (ICD9)
320.8	BACTERIAL MENINGITIS NEC (Begin 1980 (ICD9)
320.81	ANAEROBIC MENINGITIS (Begin 1992) (ICD9)
320.82	GRAM NEG MENINGITIS NEC (Begin 1992) (ICD9)
320.89	OTH BACTER MENINGITIS (Begin 1992) (ICD9)
320.9	BACTERIAL MENINGITIS NOS (ICD9)
321.0	CRYPTOCOCCAL MENINGITIS (ICD9)
321.1	MENING IN OTH FUNGAL DIS (ICD9)
321.2	MENING IN OTH VIRAL DIS (ICD9)
321.3	TRYPANOSOMIASIS MENINGIT (ICD9)
321.4	MENINGIT D/T SARCOIDOSIS (ICD9)
321.8	MENING IN OTH NONBAC DIS (ICD9)
322.0	NONPYOGENIC MENINGITIS (ICD9)
322.1	EOSINOPHILIC MENINGITIS (ICD9)
322.2	CHRONIC MENINGITIS (ICD9)
322.9	MENINGITIS NOS (ICD9)
323.0	ENCEPHALIT IN VIRAL DIS (ICD9)
323.01	ENCEPH/ENCEPHMYE OTH DIS (Begin 2006) (ICD9)
323.02	MYELITIS-OTH VIRAL DIS (Begin 2006) (ICD9)
323.1	RICKETTSIAL ENCEPHALITIS (ICD9)
323.2	PROTOZOAL ENCEPHALITIS (ICD9)
323.4	OTH ENCEPHALIT D/T INFEC (ICD9)
323.41	ENCEPH/MYELITIS-OTH INF (Begin 2006) (ICD9)
323.42	MYELITIS D/T OTH INFECT (Begin 2006) (ICD9)
323.6	POSTINFECT ENCEPHALITIS (ICD9)
323.61	INF AC DIS ENCEPHALOMYEL (Begin 2006) (ICD9)
323.62	POSTINF ENCEPHALITIS NEC (Begin 2006) (ICD9)
323.63	POSTINFECTIOUS MYELITIS (Begin 2006) (ICD9)
323.8	ENCEPHALITIS NEC (ICD9)
323.81	ENCEPH & ENCEPHLALO NEC (Begin 2006) (ICD9)
323.82	MYELITIS CAUSE NEC (Begin 2006) (ICD9)
323.9	ENCEPHALITIS NOS (ICD9)
324.0	INTRACRANIAL ABSCESS (ICD9)
324.1	INTRASPINAL ABSCESS (ICD9)

324.9	CNS ABSCESS NOS (ICD9)
325	PHLEBITIS AND THROMBOPHLEBITIS OF INTRACRANIAL VENOUS SINUSES
326	LATE EFF CNS ABSCESS (ICD9)
357.0	AC INFECT POLYNEURITIS (ICD9)
360.00	PURULENT ENDOPHTHALM NOS (ICD9)
360.01	ACUTE ENDOPHTHALMITIS (ICD9)
360.02	PANOPHTHALMITIS (ICD9)
360.03	CHRONIC ENDOPHTHALMITIS (ICD9)
360.04	VITREOUS ABSCESS (ICD9)
360.11	SYMPATHETIC UVEITIS (ICD9)
360.12	PANUVEITIS (ICD9)
360.13	PARASITIC ENDOPHTHAL NOS (ICD9)
360.14	OPHTHALMIA NODOSA (ICD9)
360.19	ENDOPHTHALMITIS NEC (ICD9)
363.20	CHORIORETINITIS NOS (ICD9)
364.03	SECONDRY IRITIS- INFECT (ICD9)
364.05	HYPOPYON (ICD9)
370.55	CORNEAL ABSCESS (ICD9)
372.00	ACUTE CONJUNCTIVITIS NOS (ICD9)
372.01	SEROUS CONJUNCTIVITIS (ICD9)
372.02	AC FOLLIC CONJUNCTIVITIS (ICD9)
372.03	MUCOPUR CONJUNCTIVIT NEC (ICD9)
372.04	PSEUDOMEMB CONJUNCTIVIT (ICD9)
372.15	PARASITIC CONJUNCTIVITIS (ICD9)
372.20	BLEPHAROCONJUNCTIVIT NOS (ICD9)
373.00	BLEPHARITIS NOS (ICD9)
373.01	ULCERATIVE BLEPHARITIS (ICD9)
373.02	SQUAMOUS BLEPHARITIS (ICD9)
373.11	HORDEOLUM EXTERNUM (ICD9)
373.12	HORDEOLUM INTERNUM (ICD9)
373.13	ABSCESS OF EYELID (ICD9)
373.2	CHALAZION (ICD9)
373.4	INFECT DERM LID W DEFORM (ICD9)
373.5	INFEC DERMATITIS LID NEC (ICD9)
373.6	PARASITIC INFEST EYELID (ICD9)
376.01	ORBITAL CELLULITIS (ICD9)
376.02	ORBITAL PERIOSTITIS (ICD9)
376.03	ORBITAL OSTEOMYELITIS (ICD9)
376.04	ORBITAL TENONITIS (ICD9)
376.13	PARASITE INFEST- ORBIT (ICD9)
379.60	INFLAM POSTPROC BLEB NOS (Begin 2006) (ICD9)
379.61	INFLAM POSTPROC BLEB (ICD9)
379.62	INFLAM POSTPROC BLEB (ICD9)
379.63	INFLAM POSTPROC BLEB (ICD9)
380.10	INFEC OTITIS EXTERNA NOS (ICD9)
380.11	ACUTE INFECTION OF PINNA (ICD9)
380.12	ACUTE SWIMMERS EAR (ICD9)
380.13	AC INFECT EXTERN EAR NEC (ICD9)
380.14	MALIGNANT OTITIS EXTERNA (ICD9)
380.15	CHR MYCOT OTITIS EXTERNA (ICD9)
380.16	CHR INF OTIT EXTERNA NEC (ICD9)
380.23	CHR OTITIS EXTERNA NEC (ICD9)
381.00	AC NONSUP OTITIS MED NOS (ICD9)
381.01	AC SEROUS OTITIS MEDIA (ICD9)
381.02	AC MUCOID OTITIS MEDIA (ICD9)
381.03	ACUTE SANGUINOUS OTITIS MEDIA (ICD9)
381.10	CHR SEROUS OM SIMP/NOS (ICD9)
381.19	CHR SEROUS OM NEC (ICD9)
381.20	CHR MUCOID OM SIMP/NOS (ICD9)
381.29	CHR MUCOID OM NEC (ICD9)

381.3 CHR NONSUP OM NOS/NEC (ICD9)
381.4 NONSUPP OTITIS MEDIA NOS (ICD9)
382.00 AC SUPP OTITIS MEDIA NOS (ICD9)
382.01 AC SUPP OM W DRUM RUPT (ICD9)
382.02 AC SUPP OM IN OTH DIS (ICD9)
382.1 CHR TUBOTYMPAN SUPPUR OM (ICD9)
382.2 CHR ATTICOANTRAL SUP OM (ICD9)
382.3 CHR SUP OTITIS MEDIA NOS (ICD9)
382.4 SUPPUR OTITIS MEDIA NOS (ICD9)
382.9 OTITIS MEDIA NOS (ICD9)
383.00 AC MASTOIDITIS W/O COMPL (ICD9)
383.01 SUBPERI MASTOID ABSCESS (ICD9)
383.02 AC MASTOIDITIS-COMPL NEC (ICD9)
383.1 CHRONIC MASTOIDITIS (ICD9)
383.20 PETROSITIS NOS (ICD9)
383.21 ACUTE PETROSITIS (ICD9)
383.22 CHRONIC PETROSITIS (ICD9)
383.30 POSTMASTOID COMPL NOS (ICD9)
383.31 POSTMASTOID MUCOSAL CYST (ICD9)
383.32 POSTMASTOID CHOLESTEATMA (ICD9)
383.33 POSTMASTOID GRANULATIONS (ICD9)
383.89 DISORDERS OF MASTOID NEC (ICD9)
383.9 MASTOIDITIS NOS (ICD9)
384.00 ACUTE MYRINGITIS UNSPECIFIED
384.01 BULLOUS MYRINGITIS
384.09 OTHER ACUTE MYRINGITIS WITHOUT OTITIS MEDIA
384.20 PERFORAT TYMPAN MEMB NOS (ICD9)
384.21 CENT PERF TYMPANIC MEMB (ICD9)
384.22 ATTIC PERF TYMPANIC MEMB (ICD9)
384.23 MARGINAL PERF TYMP NEC (ICD9)
384.24 MULT PERF TYMPANIC MEMB (ICD9)
384.25 TOTAL PERF TYMPANIC MEMB (ICD9)
386.3 LABYRINTHITIS
386.30 LABYRINTHITIS UNSPECIFIED
386.31 SEROUS LABYRINTHITIS
386.32 CIRCUMSCRIBED LABYRINTHITIS
386.33 SUPPURATIVE LABYRINTHITIS
386.34 TOXIC LABYRINTHITIS
386.35 VIRAL LABYRINTHITIS
388.6 OTORRHEA
390 RHEUM FEV W/O HRT INVOLV (ICD9)
391.0 ACUTE RHEUMATIC PERICARDITIS (ICD9)
391.1 ACUTE RHEUMATIC ENDOCARDITIS (ICD9)
391.2 ACUTE RHEUMATIC MYOCARDITIS (ICD9)
391.8 OTHER ACUTE RHEUMATIC HEART DISEASE (ICD9)
391.9 ACUTE RHEUMATIC HEART DISEASE UNSPECIFIED (ICD9)
392.0 RHEUMATIC CHOREA WITH HEART INVOLVEMENT (ICD9)
392.9 RHEUMATIC CHOREA NOS (ICD9)
393 CHRONIC RHEUMATIC PERICARDITIS
421.0 ACUTE AND SUBACUTE BACTERIAL ENDOCARDITIS
449 SEPTIC ARTERIAL EMBOLISM (Begin 2007) (ICD9)
460 ACUTE NASOPHARYNGITIS (ICD9)
461.0 AC MAXILLARY SINUSITIS (ICD9)
461.1 AC FRONTAL SINUSITIS (ICD9)
461.2 AC ETHMOIDAL SINUSITIS (ICD9)
461.3 AC SPHENOIDAL SINUSITIS (ICD9)
461.8 OTHER ACUTE SINUSITIS (ICD9)
461.9 ACUTE SINUSITIS NOS (ICD9)
462 ACUTE PHARYNGITIS (ICD9)
463 ACUTE TONSILLITIS (ICD9)

464.0 ACUTE LARYNGITIS (End 2001) (ICD9)
464.00 ACUTE LARYNGITIS- W/O OBSTR (Begin 2001) (ICD9)
464.01 ACUTE LARYNGITIS- W OBSTR (Begin 2001) (ICD9)
464.10 AC TRACHEITIS NO OBSTRUC (ICD9)
464.11 AC TRACHEITIS W OBSTRUCT (ICD9)
464.20 AC LARYNGOTRACH NO OBSTR (ICD9)
464.21 AC LARYNGOTRACH W OBSTR (ICD9)
464.30 AC EPIGLOTTITIS NO OBSTR (ICD9)
464.31 AC EPIGLOTTITIS W OBSTR (ICD9)
464.4 CROUP (ICD9)
464.50 SUPRAGLOTTIS NOS- W/O OBSTR (Begin 2001) (ICD9)
464.51 SUPRAGLOTTIS NOS- W/ OBSTR (Begin 2001) (ICD9)
465.0 ACUTE LARYNGOPHARYNGITIS (ICD9)
465.8 ACUTE URI MULT SITES NEC (ICD9)
465.9 ACUTE URI NOS (ICD9)
466.0 ACUTE BRONCHITIS (ICD9)
466.1 ACUTE BRONCHIOLITIS (End 1996) (ICD9)
466.11 RSV BRONCHIOLITIS (Begin 1996) (ICD9)
466.19 OTH ACUTE BRONCHIOL (Begin 1996) (ICD9)
473.0 CHR MAXILLARY SINUSITIS (ICD9)
473.1 CHR FRONTAL SINUSITIS (ICD9)
473.2 CHR ETHMOIDAL SINUSITIS (ICD9)
473.3 CHR SPHENOIDAL SINUSITIS (ICD9)
473.8 CHRONIC SINUSITIS NEC (ICD9)
473.9 CHRONIC SINUSITIS NOS- (ICD9)
474.0 CHRONIC TONSILLITIS (End 1997) (ICD9)
474.00 CHRON TONSILLITIS (Begin 1997) (ICD9)
474.01 CHRON ADENOIDITIS (Begin 1997) (ICD9)
474.02 CHRON TONSIL ADENOID (Begin 1997) (ICD9)
475 PERITONSILLAR ABSCESS (ICD9)
476.0 CHRONIC LARYNGITIS (ICD9)
476.1 CHRONIC LARYNGOTRACHEITIS (ICD9)
478.21 CELLULITIS OF PHARYNX (ICD9)
478.22 PARAPHARYNGEAL ABSCESS (ICD9)
478.24 RETROPHARYNGEAL ABSCESS (ICD9)
478.29 DISEASE OF PHARYNX NEC (ICD9)
478.71 LARYNGEAL CELLULITIS (ICD9)
480.0 ADENOVIRAL PNEUMONIA (ICD9)
480.1 RESP SYNCYT VIRAL PNEUM (ICD9)
480.2 PARINFLUENZA VIRAL PNEUM (ICD9)
480.3 PNEUMONIA DUE TO SARS-ASSOCIATED CORONAVIRUS (Begin 2003) (ICD9)
480.8 VIRAL PNEUMONIA NEC (ICD9)
480.9 VIRAL PNEUMONIA NOS (ICD9)
481 PNEUMOCOCCAL PNEUMONIA (ICD9)
481.9 (no description found)
482.0 K. PNEUMONIAE PNEUMONIA (ICD9)
482.1 PSEUDOMONAL PNEUMONIA (ICD9)
482.2 H.INFLUENZAE PNEUMONIA (ICD9)
482.3 STREPTOCOCCAL PNEUMONIA (Begin 1980 (ICD9)
482.30 STREP PNEUMONIA UNSPEC (Begin 1992) (ICD9)
482.31 GRP A STREP PNEUMONIA (Begin 1992) (ICD9)
482.32 GRP B STREP PNEUMONIA (Begin 1992) (ICD9)
482.39 OTH STREP PNEUMONIA (Begin 1992) (ICD9)
482.4 STAPHYLOCOCCAL PNEUMONIA (End 1998) (ICD9)
482.40 STAPH PNEUMONIA UNSP (Begin 1998) (ICD9)
482.41 STAPH AUREUS PNEUMON (Begin 1998) (ICD9)
482.42 (no description found)
482.49 STAPH PNEUMON OTH (Begin 1998) (ICD9)
482.8 BACTERIAL PNEUMONIA NEC (Begin 1980 (ICD9)
482.81 ANAEROBIC PNEUMONIA (Begin 1992) (ICD9)

482.82 E COLI PNEUMONIA (Begin 1992) (ICD9)
482.83 OTH GRAM NEG PNEUMONIA (Begin 1992) (ICD9)
482.84 LEGIONNAIRES DX (Begin 1997) (ICD9)
482.89 BACT PNEUMONIA NEC (Begin 1992) (ICD9)
482.9 BACTERIAL PNEUMONIA NOS (ICD9)
483 PNEUMONIA: ORGANISM NEC (Begin 1980 (ICD9)
483.0 MYCOPLASMA PNEUMONIA (Begin 1992) (ICD9)
483.1 CHLAMYDIA PNEUMONIA (Begin 1996) (ICD9)
483.8 OTH SPEC ORG PNEUMONIA (Begin 1992) (ICD9)
484.1 PNEUM W CYTOMEG INCL DIS (ICD9)
484.3 PNEUMONIA IN WHOOP COUGH (ICD9)
484.5 PNEUMONIA IN ANTHRAX (ICD9)
484.6 PNEUM IN ASPERGILLOSIS (ICD9)
484.7 PNEUM IN OTH SYS MYCOSES (ICD9)
484.8 PNEUM IN INFECT DIS NEC (ICD9)
485 BRONCOPNEUMONIA ORG NOS (ICD9)
486 PNEUMONIA- ORGANISM NOS (ICD9)
487.0 INFLUENZA WITH PNEUMONIA (ICD9)
487.1 FLU W RESP MANIFEST NEC (ICD9)
487.8 FLU W MANIFESTATION NEC (ICD9)
488 FLU D/T AVIAN FLU VIRUS (Begin 2007) (ICD9)
488.0 (no description found)
488.01 FLU D/T IDENTIFIED AVIAN FLU VIRUS WITH PNEUMONIA
488.02 FLU D/T IDENTIFIED AVIAN FLU VIRUS WITH OTHER RESPIRATORY MANIFESTATIONS
488.09 FLU D/T IDENTIFIED AVIAN FLU VIRUS WITH OTHER MANIFESTATIONS
488.1 (no description found)
488.11 FLU D/T IDENTIFIED 2009 H1N1 FLU VIRUS WITH PNEUMONIA
488.12 FLU D/T IDENTIFIED 2009 H1N1 FLU VIRUS WITH OTHER RESPIRATORY MANIFESTATIONS
488.19 FLU D/T IDENTIFIED 2009 H1N1 FLU VIRUS WITH OTHER MANIFESTATIONS
488.81 FLU D/T IDENTIFIED NOVEL INFLUENZA A VIRUS W/ PNEUMONIA
488.82 FLU D/T IDENTIFIED NOVEL INFLUENZA A VIRUS W/ OTHER RESP MANIFESTATION
488.89 FLU D/T IDENTIFIED NOVEL INFLUENZA A VIRUS W/ OTHER MANIFESTATIONS
490 BRONCHITIS NOS (ICD9)
491.0 SIMPLE CHR BRONCHITIS (ICD9)
491.1 MUCOPURUL CHR BRONCHITIS (ICD9)
491.2 OBSTRUCT CHR BRONCHITIS (Begin 1980 (ICD9)
491.20 OBS CHR BRNC W/O ACT EXA (Begin 1991) (ICD9)
491.21 OBS CHR BRNC W ACT EXA (Begin 1991) (ICD9)
491.22 OBS CHR BRONC W AC BRONC (Begin 2004) (ICD9)
491.8 CHRONIC BRONCHITIS NEC (ICD9)
491.9 CHRONIC BRONCHITIS NOS (ICD9)
494 BRONCHIECTASIS (End 2000) (ICD9)
494.0 BRONCHIECTASIS W/O ACUTE EXACERBATN (Begin 2000) (ICD9)
494.1 BRONCHIECTASIS W/ACUTE EXACERBATION (Begin 2000) (ICD9)
510.0 EMPYEMA WITH FISTULA (ICD9)
510.9 EMPYEMA W/O FISTULA (ICD9)
513.0 ABSCESS OF LUNG (ICD9)
513.1 ABSCESS OF MEDIASTINUM (ICD9)
517.1 RHEUMATIC PNEUMONIA (ICD9)
519.2 MEDIASTINITIS (ICD9)
519.3 MEDIASTINUM DISEASE NEC (ICD9)
522.4 AC APICAL PERIODONTITIS (ICD9)
522.5 PERIAPICAL ABSCESS (ICD9)
522.6 CHR APICAL PERIODONTITIS (ICD9)
522.7 PERIAPICAL ABSC W SINUS (ICD9)
523.10 CHRONIC GINGIVITIS, PLAQUE INDUCED (ICD9)
523.11 CHRONIC GINGIVITIS, NON-PLAQUE INDUCED (ICD9)

523.3 ACUTE PERIODONTITIS (ICD9)
523.30 AGGRESSIVE PERIODONTITIS, UNSPECIFIED (ICD9)
523.31 AGGRESSIVE PERIODONTITIS, LOCALIZED (ICD9)
523.32 AGGRESSIVE PERIODONTITIS, GENERALIZED (ICD9)
523.33 ACUTE PERIODONTITIS (ICD9)
523.4 CHRONIC PERIODONTITIS (ICD9)
523.40 CHRONIC PERIODONTITIS, UNSPECIFIED (ICD9)
523.41 CHRONIC PERIODONTITIS, LOCALIZED (ICD9)
523.42 CHRONIC PERIODONTITIS, GENERALIZED (ICD9)
523.5 PERIODONTOSIS (ICD9)
527.3 SALIVARY GLAND ABSCESS (ICD9)
528.3 CELLULITIS/ABSCESS MOUTH (ICD9)
558.9 NONINF GASTROENTERIT NEC (ICD9)
566 ABSCESS OF ANAL AND RECTAL REGION (ICD9)
567.0 PERITONITIS IN INFEC DIS (ICD9)
567.1 PNEUMOCOCCAL PERITONITIS (ICD9)
567.2 SUPPURAT PERITONITIS NEC (End 2005) (ICD9)
567.21 PERITONITIS (ACUTE) GEN (Begin 2005) (ICD9)
567.22 PERITONEAL ABSCESS (Begin 2005) (ICD9)
567.23 SPONTAN BACT PERITONITIS (Begin 2005) (ICD9)
567.29 SUPPURAT PERITONITIS NEC (Begin 2005) (ICD9)
567.31 PSOAS MUSCLE ABSCESS (Begin 2005) (ICD9)
567.38 RETROPERITON ABSCESS NEC (Begin 2005) (ICD9)
567.39 RETROPERITON INFECT NEC (Begin 2005) (ICD9)
567.81 CHOLEPERITONITIS (Begin 2005) (ICD9)
567.89 PERITONITIS NEC (Begin 2005) (ICD9)
567.9 PERITONITIS NOS (ICD9)
569.5 INTESTINAL ABSCESS (ICD9)
572.0 ABSCESS OF LIVER (ICD9)
572.1 PORTAL PYEMIA (ICD9)
573.1 HEPATITIS IN VIRAL DIS (ICD9)
573.2 HEPATITIS IN OTH INF DIS (ICD9)
574.11 CHOLELITH/GB INF NEC-OBS (ICD9)
574.30 CHOLEDOCHOLITH/AC GB INF (ICD9)
574.40 CHOLEDOCHLITH/GB INF NEC (ICD9)
575.0 ACUTE CHOLECYSTITIS
590.2 RENAL/PERIRENAL ABSCESS (ICD9)
590.81 PYELITIS OR PYELONEPHRITIS IN DISEASES CLASSIFIED ELSEWHERE
590.9 INFECTION OF KIDNEY UNSPECIFIED
595.0 ACUTE CYSTITIS (ICD9)
595.89 OTHER CYSTITIS, INCL ABSCESS OF BLADDER
597.0 URETHRAL ABSCESS (ICD9)
598.00 URETHR STRICT:INFECT NOS (ICD9)
599.0 URIN TRACT INFECTION NOS (ICD9)
601.0 ACUTE PROSTATITIS (ICD9)
601.2 ABSCESS OF PROSTATE (ICD9)
603.1 INFECTED HYDROCELE (ICD9)
604.0 ORCHITIS WITH ABSCESS (ICD9)
611.0 INFLAMMATORY DISEASE OF BREAST
614.0 AC SALPINGO-OOPHORITIS (ICD9)
614.1 CHR SALPINGO-OOPHORITIS (ICD9)
614.2 SALPINGO-OOPHORITIS NOS (ICD9)
614.3 ACUTE PARAMETRITIS (ICD9)
614.4 CHRONIC PARAMETRITIS (ICD9)
614.5 AC PELV PERITONITIS-FEM (ICD9)
614.6 FEM PELVIC PERITON ADHES (ICD9)
614.7 CHR PELV PERITON NEC-FEM (ICD9)
614.8 FEM PELV INFLAM DIS NEC (ICD9)
614.9 FEM PELV INFLAM DIS NOS (ICD9)
615.0 AC UTERINE INFLAMMATION (ICD9)

615.1 CHR UTERINE INFLAMMATION (ICD9)
615.9 UTERINE INFLAM DIS NOS (ICD9)
616.0 CERVICITIS (ICD9)
616.10 VAGINITIS NOS (ICD9)
616.11 VAGINITIS IN OTH DISEASE (ICD9)
616.2 BARTHOLIN-s GLAND CYST (ICD9)
616.3 BARTHOLIN-s GLND ABSCESS (ICD9)
616.4 ABSCESS OF VULVA NEC (ICD9)
634.00 SPON ABOR W PEL INF-UNSP (ICD9)
634.01 SPON ABOR W PELV INF-INC (ICD9)
634.02 SPON ABOR W PEL INF-COMP (ICD9)
635.00 LEG ABOR W PELV INF-UNSP (ICD9)
635.01 LEG ABOR W PELV INF-INC (ICD9)
635.02 LEG ABOR W PELV INF-COMP (ICD9)
636.00 ILLEG AB W PELV INF-UNSP (ICD9)
636.01 ILLEG AB W PELV INF-INC (ICD9)
636.02 ILLEG AB W PELV INF-COMP (ICD9)
637.00 ABORT NOS W PEL INF-UNSP (ICD9)
637.01 ABORT NOS W PEL INF-INC (ICD9)
637.02 ABORT NOS W PEL INF-COMP (ICD9)
638.0 FAILED ATTEMPTED ABORTION COMPLICATED BY GENITAL TRACT AND PELVIC INFECTION
639.0 POSTABORTION GU INFECT (ICD9)
646.60 GU INFECT IN PREG-UNSPEC (ICD9)
646.61 GU INFECTION-DELIVERED (ICD9)
646.62 GU INFECTION-DELIV W P/P (ICD9)
646.63 GU INFECTION-ANTEPARTUM (ICD9)
646.64 POSTPARTUM INFECTIONS OF GENITOURINARY TRACT
647.00 SYPHILIS OF MOTHER COMPLICATING PREGNANCY CHILDBIRTH OR THE PUERPERIUS UNSPECIFIED AS TO EPISODE OF CARE
647.01 SYPHILIS OF MOTHER COMPLICATING PREGNANCY WITH DELIVERY
647.02 SYPHILIS OF MOTHER COMPLICATING PREGNANCY WITH DELIVERY WITH POSTPARTUM COMPLICATION
647.03 ANTEPARTUM SYPHILIS
647.04 POSTPARTUM SYPHILIS
647.10 GONORRHEA OF MOTHER COMPLICATING PREGNANCY CHILDBIRTH OR THE PUERPERIUM UNSPECIFIED AS TO EPISODE OF CARE
647.11 GONORRHEA OF MOTHER WITH DELIVERY
647.12 GONORRHEA OF MOTHER WITH DELIVERY WITH POSTPARTUM COMPLICATION
647.13 ANTEPARTUM GONORRHEA
647.14 POSTPARTUM GONORRHEA
647.20 OTHER VENEREAL DISEASES OF MOTHER COMPLICATING PREGNANCY CHILDBIRTH OR PUERPERIUM UNSPECIFIED AS TO EPISODE OF CARE
647.21 OTHER VENEREAL DISEASES OF MOTHER WITH DELIVERY
647.22 OTHER VENEREAL DISEASES OF MOTHER WITH DELIVERY WITH POSTPARTUM COMPLICATION
647.23 OTHER ANTEPARTUM VENEREAL DISEASES
647.24 OTHER POSTPARTUM VENEREAL DISEASES
647.30 TB IN PREG-UNSPECIFIED (ICD9)
647.31 TUBERCULOSIS-DELIVERED (ICD9)
647.32 TUBERCULOSIS-DELIV W P/P (ICD9)
647.33 TUBERCULOSIS-ANTEPARTUM (ICD9)
647.34 TUBERCULOSIS-POSTPARTUM (ICD9)
647.40 MALARIA IN PREG-UNSPEC (ICD9)
647.41 MALARIA-DELIVERED (ICD9)
647.42 MALARIA-DELIVERED W P/P (ICD9)
647.43 MALARIA-ANTEPARTUM (ICD9)
647.44 MALARIA-POSTPARTUM (ICD9)
647.50 RUBELLA IN PREG-UNSPEC (ICD9)
647.51 RUBELLA-DELIVERED (ICD9)

647.52 RUBELLA-DELIVERED W P/P (ICD9)
647.53 RUBELLA-ANTEPARTUM (ICD9)
647.54 RUBELLA-POSTPARTUM (ICD9)
647.60 OTH VIRUS IN PREG-UNSPEC (ICD9)
647.61 OTH VIRAL DIS-DELIVERED (ICD9)
647.62 OTH VIRAL DIS-DEL W P/P (ICD9)
647.63 OTH VIRAL DIS-ANTEPARTUM (ICD9)
647.64 OTH VIRAL DIS-POSTPARTUM (ICD9)
647.80 INF DIS IN PREG NEC-UNSP (ICD9)
647.81 INFECT DIS NEC-DELIVERED (ICD9)
647.82 INFECT DIS NEC-DEL W P/P (ICD9)
647.83 INFECT DIS NEC-ANTEPART (ICD9)
647.84 INFECT DIS NEC-POSTPART (ICD9)
647.90 INFECT IN PREG NOS-UNSP (ICD9)
647.91 INFECT NOS-DELIVERED (ICD9)
647.92 INFECT NOS-DELIVER W P/P (ICD9)
647.93 INFECT NOS-ANTEPARTUM (ICD9)
647.94 INFECT NOS-POSTPARTUM (ICD9)
658.40 AMNIOTIC INFECTION-UNSP (ICD9)
658.41 AMNIOTIC INFECTION-DELIV (ICD9)
658.43 AMNIOTIC INFECT-ANTEPART (ICD9)
659.30 SEPTICEMIA IN LABOR-UNSP (ICD9)
659.31 SEPTICEM IN LABOR-DELIV (ICD9)
659.33 SEPTICEM IN LABOR-ANTEPA (ICD9)
670.00 MAJOR PUERP INFECT-UNSP (Begin 1991) (ICD9)
670.02 MAJOR PUERP INF-DEL P/P (Begin 1991) (ICD9)
670.04 MAJOR PUERP INF-POSTPART (Begin 1991) (ICD9)
670.10 PUERP ENDOMETRITIS, UNSP
670.12 PUERP ENDOMETRITIS, DELIVERED WITH MENTION OF POSTPARTUM COMPLICATION
670.14 PUERP ENDOMETRITIS, POSTPARTUM CONDITION OR COMPLICATION
670.20 PUERP SEPSIS, UNSP
670.22 PUERP SEPSIS, DELIVERED WITH MENTION OF POSTPARTUM COMPLICATION
670.24 PUERP SEPSIS, POSTPARTUM CONDITION OR COMPLICATION
670.30 PUERP SEPTIC THROMBOPHLEBITIS, UNSP
670.32 PUERP SEPTIC THROMBOPHLEBITIS, DELIVERED WITH MENTION OF POSTPARTUM COMPLICATION
670.34 PUERP SEPTIC THROMBOPHLEBITIS, POSTPARTUM CONDITION OR COMPLICATION
670.80 OTHER MAJOR PUERP INFECTION, UNSP
670.82 OTHER MAJOR PUERP INFECTION, DELIVERED WITH MENTION OF POSTPARTUM COMPLICATION
670.84 OTHER MAJOR PUERP INFECTION, POSTPARTUM CONDITION OR COMPLICATION
675.00 INFECT NIPPLE PREG-UNSP (ICD9)
675.01 INFECT NIPPLE-DELIVERED (ICD9)
675.02 INFECT NIPPLE-DEL W P/P (ICD9)
675.03 INFECT NIPPLE-ANTEPARTUM (ICD9)
675.04 INFECT NIPPLE-POSTPARTUM (ICD9)
675.10 BREAST ABSCESS PREG-UNSP (ICD9)
675.11 BREAST ABSCESS-DELIVERED (ICD9)
675.12 BREAST ABSCESS-DEL W P/P (ICD9)
675.13 BREAST ABSCESS-ANTEPART (ICD9)
675.14 BREAST ABSCESS-POSTPART (ICD9)
675.20 MASTITIS IN PREG-UNSPEC (ICD9)
675.21 MASTITIS-DELIVERED (ICD9)
675.22 MASTITIS-DELIV W P/P (ICD9)
675.23 MASTITIS-ANTEPARTUM (ICD9)
675.24 MASTITIS-POSTPARTUM (ICD9)
675.80 BREAST INF PREG NEC-UNSP (ICD9)
675.81 BREAST INFECT NEC-DELIV (ICD9)

675.82 BREAST INF NEC-DEL W P/P (ICD9)
675.83 BREAST INF NEC-ANTEPART (ICD9)
675.84 BREAST INF NEC-POSTPART (ICD9)
675.90 BREAST INF PREG NOS-UNSP (ICD9)
675.91 BREAST INFECT NOS-DELIV (ICD9)
675.92 BREAST INF NOS-DEL W P/P (ICD9)
675.93 BREAST INF NOS-ANTEPART (ICD9)
675.94 BREAST INF NOS-POSTPART (ICD9)
680.0 CARBUNCLE OF FACE (ICD9)
680.1 CARBUNCLE OF NECK (ICD9)
680.2 CARBUNCLE OF TRUNK (ICD9)
680.3 CARBUNCLE OF ARM (ICD9)
680.4 CARBUNCLE OF HAND (ICD9)
680.5 CARBUNCLE OF BUTTOCK (ICD9)
680.6 CARBUNCLE OF LEG (ICD9)
680.7 CARBUNCLE OF FURUNCLE OF FOOT (ICD9)
680.8 CARBUNCLE- SITE NEC (ICD9)
680.9 CARBUNCLE NOS (ICD9)
681.00 CELLULITIS- FINGER NOS (ICD9)
681.01 FELON (ICD9)
681.02 ONYCHIA OF FINGER (ICD9)
681.10 CELLULITIS- TOE NOS (ICD9)
681.11 ONYCHIA OF TOE (ICD9)
681.9 CELLULITIS OF DIGIT NOS (ICD9)
682.0 CELLULITIS OF FACE (ICD9)
682.1 CELLULITIS OF NECK (ICD9)
682.2 CELLULITIS OF TRUNK (ICD9)
682.3 CELLULITIS OF ARM (ICD9)
682.4 CELLULITIS OF HAND (ICD9)
682.5 CELLULITIS OF BUTTOCK (ICD9)
682.6 CELLULITIS OF LEG (ICD9)
682.7 CELLULITIS OF FOOT (ICD9)
682.8 CELLULITIS- SITE NEC (ICD9)
682.9 CELLULITIS NOS (ICD9)
683 ACUTE LYMPHADENITIS (ICD9)
684 IMPETIGO (ICD9)
685.0 PILONIDAL CYST W ABSCESS (ICD9)
685.1 PILONIDAL CYST W/O ABSC (ICD9)
686.0 PYODERMA (End 1997) (ICD9)
686.00 PYODERMA NOS (Begin 1997) (ICD9)
686.01 PYODERMA GANGREN (Begin 1997) (ICD9)
686.09 PYODERMA NEC (Begin 1997) (ICD9)
686.1 PYOGENIC GRANULOMA (ICD9)
686.8 LOCAL SKIN INFECTION NEC (ICD9)
686.9 LOCAL SKIN INFECTION NOS (ICD9)
690.8 OTHER ERYTHEMATOSQUAMOUS DERMATOSIS
694.0 DERMATITIS HERPETIFORMIS (ICD9)
694.1 SUBCORNEAL PUST DERMATOS (ICD9)
694.2 JUVEN DERMAT HERPETIFORM (ICD9)
694.3 IMPETIGO HERPETIFORMIS (ICD9)
695.3 ROSACEA (ICD9)
695.81 RITTER’S DISEASE (ICD9)
696.3 PITYRIASIS ROSEA (ICD9)
711.00 PYOGEN ARTHRITIS-UNSPEC (ICD9)
711.01 PYOGEN ARTHRITIS-SHLDER (ICD9)
711.02 PYOGEN ARTHRITIS-UP/ARM (ICD9)
711.03 PYOGEN ARTHRITIS-FOREARM (ICD9)
711.04 PYOGEN ARTHRITIS-HAND (ICD9)
711.05 PYOGEN ARTHRITIS-PELVIS (ICD9)
711.06 PYOGEN ARTHRITIS-L/LEG (ICD9)

711.07 PYOGEN ARTHRITIS-ANKLE (ICD9)
711.08 PYOGEN ARTHRITIS NEC (ICD9)
711.09 PYOGEN ARTHRITIS-MULT (ICD9)
711.30 DYSENTER ARTHRIT-UNSPEC (ICD9)
711.31 DYSENTER ARTHRIT-SHLDER (ICD9)
711.32 DYSENTER ARTHRIT-UP/ARM (ICD9)
711.33 DYSENTER ARTHRIT-FOREARM (ICD9)
711.34 DYSENTER ARTHRIT-HAND (ICD9)
711.35 DYSENTER ARTHRIT-PELVIS (ICD9)
711.36 DYSENTER ARTHRIT-L/LEG (ICD9)
711.37 DYSENTER ARTHRIT-ANKLE (ICD9)
711.38 DYSENTER ARTHRIT NEC (ICD9)
711.39 DYSENTER ARTHRIT-MULT (ICD9)
711.40 BACT ARTHRITIS-UNSPEC (ICD9)
711.41 BACT ARTHRITIS-SHLDER (ICD9)
711.42 BACT ARTHRITIS-UP/ARM (ICD9)
711.43 BACT ARTHRITIS-FOREARM (ICD9)
711.44 BACT ARTHRITIS-HAND (ICD9)
711.45 BACT ARTHRITIS-PELVIS (ICD9)
711.46 BACT ARTHRITIS-L/LEG (ICD9)
711.47 BACT ARTHRITIS-ANKLE (ICD9)
711.48 BACT ARTHRITIS NEC (ICD9)
711.49 BACT ARTHRITIS-MULT (ICD9)
711.50 VIRAL ARTHRITIS-UNSPEC (ICD9)
711.51 VIRAL ARTHRITIS-SHLDER (ICD9)
711.52 VIRAL ARTHRITIS-UP/ARM (ICD9)
711.53 VIRAL ARTHRITIS-FOREARM (ICD9)
711.54 VIRAL ARTHRITIS-HAND (ICD9)
711.55 VIRAL ARTHRITIS-PELVIS (ICD9)
711.56 VIRAL ARTHRITIS-L/LEG (ICD9)
711.57 VIRAL ARTHRITIS-ANKLE (ICD9)
711.58 VIRAL ARTHRITIS NEC (ICD9)
711.59 VIRAL ARTHRITIS-MULT (ICD9)
711.60 MYCOTIC ARTHRITIS-UNSPEC (ICD9)
711.61 MYCOTIC ARTHRITIS-SHLDER (ICD9)
711.62 MYCOTIC ARTHRITIS-UP/ARM (ICD9)
711.63 MYCOTIC ARTHRIT-FOREARM (ICD9)
711.64 MYCOTIC ARTHRITIS-HAND (ICD9)
711.65 MYCOTIC ARTHRITIS-PELVIS (ICD9)
711.66 MYCOTIC ARTHRITIS-L/LEG (ICD9)
711.67 MYCOTIC ARTHRITIS-ANKLE (ICD9)
711.68 MYCOTIC ARTHRITIS NEC (ICD9)
711.69 MYCOTIC ARTHRITIS-MULT (ICD9)
711.70 HELMINTH ARTHRIT-UNSPEC (ICD9)
711.71 HELMINTH ARTHRIT-SHLDER (ICD9)
711.72 HELMINTH ARTHRIT-UP/ARM (ICD9)
711.73 HELMINTH ARTHRIT-FOREARM (ICD9)
711.74 HELMINTH ARTHRIT-HAND (ICD9)
711.75 HELMINTH ARTHRIT-PELVIS (ICD9)
711.76 HELMINTH ARTHRIT-L/LEG (ICD9)
711.77 HELMINTH ARTHRIT-ANKLE (ICD9)
711.78 HELMINTH ARTHRIT NEC (ICD9)
711.79 HELMINTH ARTHRIT-MULT (ICD9)
711.80 INF ARTHRITIS NEC-UNSPEC (ICD9)
711.81 INF ARTHRITIS NEC-SHLDER (ICD9)
711.82 INF ARTHRITIS NEC-UP/ARM (ICD9)
711.83 INF ARTHRIT NEC-FOREARM (ICD9)
711.84 INF ARTHRITIS NEC-HAND (ICD9)
711.85 INF ARTHRITIS NEC-PELVIS (ICD9)
711.86 INF ARTHRITIS NEC-L/LEG (ICD9)

711.87 INF ARTHRITIS NEC-ANKLE (ICD9)
711.88 INF ARTHRIT NEC-OTH SITE (ICD9)
711.89 INF ARTHRITIS NEC-MULT (ICD9)
711.90 INF ARTHRITIS NOS-UNSPEC (ICD9)
711.91 INF ARTHRITIS NOS-SHLDER (ICD9)
711.92 INF ARTHRITIS NOS-UP/ARM (ICD9)
711.93 INF ARTHRIT NOS-FOREARM (ICD9)
711.94 INF ARTHRIT NOS-HAND (ICD9)
711.95 INF ARTHRIT NOS-PELVIS (ICD9)
711.96 INF ARTHRIT NOS-L/LEG (ICD9)
711.97 INF ARTHRIT NOS-ANKLE (ICD9)
711.98 INF ARTHRIT NOS-OTH SITE (ICD9)
711.99 INF ARTHRITIS NOS-MULT (ICD9)
728.0 INFECTIVE MYOSITIS (ICD9)
728.86 NECROTIZING FASCIITIS
730.00 AC OSTEOMYELITIS-UNSPEC (ICD9)
730.01 AC OSTEOMYELITIS-SHLDER (ICD9)
730.02 AC OSTEOMYELITIS-UP/ARM (ICD9)
730.03 AC OSTEOMYELITIS-FOREARM (ICD9)
730.04 AC OSTEOMYELITIS-HAND (ICD9)
730.05 AC OSTEOMYELITIS-PELVIS (ICD9)
730.06 AC OSTEOMYELITIS-L/LEG (ICD9)
730.07 AC OSTEOMYELITIS-ANKLE (ICD9)
730.08 AC OSTEOMYELITIS NEC (ICD9)
730.09 AC OSTEOMYELITIS-MULT (ICD9)
730.10 CHR OSTEOMYELITIS-UNSP (ICD9)
730.11 CHR OSTEOMYELIT-SHLDER (ICD9)
730.12 CHR OSTEOMYELIT-UP/ARM (ICD9)
730.13 CHR OSTEOMYELIT-FOREARM (ICD9)
730.14 CHR OSTEOMYELIT-HAND (ICD9)
730.15 CHR OSTEOMYELIT-PELVIS (ICD9)
730.16 CHR OSTEOMYELIT-L/LEG (ICD9)
730.17 CHR OSTEOMYELIT-ANKLE (ICD9)
730.18 CHR OSTEOMYELIT NEC (ICD9)
730.19 CHR OSTEOMYELIT-MULT (ICD9)
730.20 OSTEOMYELITIS NOS-UNSPEC (ICD9)
730.21 OSTEOMYELITIS NOS-SHLDER (ICD9)
730.22 OSTEOMYELITIS NOS-UP/ARM (ICD9)
730.23 OSTEOMYELIT NOS-FOREARM (ICD9)
730.24 OSTEOMYELITIS NOS-HAND (ICD9)
730.25 OSTEOMYELITIS NOS-PELVIS (ICD9)
730.26 OSTEOMYELITIS NOS-L/LEG (ICD9)
730.27 OSTEOMYELITIS NOS-ANKLE (ICD9)
730.28 OSTEOMYELIT NOS-OTH SITE (ICD9)
730.29 OSTEOMYELITIS NOS-MULT (ICD9)
730.80 BONE INFECT NEC-UNSPEC (ICD9)
730.81 BONE INFECT NEC-SHLDER (ICD9)
730.82 BONE INFECT NEC-UP/ARM (ICD9)
730.83 BONE INFECT NEC-FOREARM (ICD9)
730.84 BONE INFECT NEC-HAND (ICD9)
730.85 BONE INFECT NEC-PELVIS (ICD9)
730.86 BONE INFECT NEC-L/LEG (ICD9)
730.87 BONE INFECT NEC-ANKLE (ICD9)
730.88 BONE INFECT NEC-OTH SITE (ICD9)
730.89 BONE INFECT NEC-MULT (ICD9)
730.90 BONE INFEC NOS-UNSP SITE (ICD9)
730.91 BONE INFECT NOS-SHLDER (ICD9)
730.92 BONE INFECT NOS-UP/ARM (ICD9)
730.93 BONE INFECT NOS-FOREARM (ICD9)
730.94 BONE INFECT NOS-HAND (ICD9)

730.95 BONE INFECT NOS-PELVIS (ICD9)
730.96 BONE INFECT NOS-L/LEG (ICD9)
730.97 BONE INFECT NOS-ANKLE (ICD9)
730.98 BONE INFECT NOS-OTH SITE (ICD9)
730.99 BONE INFECT NOS-MULT (ICD9)
760.2 MATERNAL INFEC AFF NB (ICD9)
762.7 CHORIOAMNIONITIS AFF NB (ICD9)
770.0 CONGENITAL PNEUMONIA (ICD9)
771.0 CONGENITAL RUBELLA (ICD9)
771.1 CONGENITAL CYTOMEGALOVIRUS INFECTION (ICD9)
771.2 OTHER CONGENITAL INFECTIONS SPECIFIED TO THE PERINATAL PERIOD
771.3 TETANUS NEONATORUM (ICD9)
771.4 OMPHALITIS OF NEWBORN (ICD9)
771.5 NEONATAL INFEC MASTITIS (ICD9)
771.6 NEONATAL CONJUNCTIVITIS (ICD9)
771.7 NEONATAL CANDIDA INFECT (ICD9)
771.8 PERINATAL INFECTION NEC (End 2002) (ICD9)
771.81 SEPTICEMIA [SEPSIS] OF NEWBORN (Begin 2002) (ICD9)
771.82 URINARY TRACT INFECTION OF NEWBORN (Begin 2002) (ICD9)
771.83 BACTEREMIA OF NEWBORN (Begin 2002) (ICD9)
771.89 OT INFECTIONS SPEC TO PERINATAL PERIOD (Begin 2002) (ICD9)
785.52 SEPTIC SHOCK (Begin 2003) (ICD9)
790.7 BACTEREMIA NOS (ICD9)
790.8 VIREMIA NOS- (ICD9)
795.05 CERVICAL (HPV) DNA POS (Begin 2004) (ICD9)
795.15 (no description found)
795.19 (no description found)
795.3 POSITIVE CULTURE FINDING (End 2002) (ICD9)
795.31 NONSPECIFIC POSITIVE FINDINGS/ANTHRAX (Begin 2002) (ICD9)
795.39 OTHER NONSPECIFIC POSITIVE CULTURE (Begin 2002) (ICD9)
795.71 NONSPECIFIC SEROLOG HIV (Begin 1994) (ICD9)
795.8 POSITIVE SERO/VIRAL HIV (Begin 1986 (ICD9)
796.75 (no description found)
796.79 (no description found)
910.1 ABRASION OR FRICTION BURN OF FACE NECK AND SCALP EXCEPT EYE INFECTED
910.3 BLISTER OF FACE NECK AND SCALP EXCEPT EYE INFECTED
910.5 INSECT BITE HEAD-INFECT (ICD9)
910.7 SUPERFICIAL FOREIGN BODY (SPLINTER) OF FACE NECK AND SCALP EXCET EYE WITHOUT MAJOR OPEN WOUND INFECTED
910.9 OTHER AND UNSPECIFIED SUPERFICIAL INJURY OF FACE NECK AND SCALP INFECTED
911.1 ABRASION OR FRICTION BURN OF TRUNK INFECTED
911.3 BLISTER OF TRUNK INFECTED
911.5 INSECT BITE NONVENOMOUS OF TRUNK INFECTED
911.7 SUPERFICIAL FOREIGN BODY (SPLINTER) OF TRUNK WITHOUT MAJOR OPEN WOUND INFECTED
911.9 OTHER AND UNSPECIFIED SUPERFICIAL INJURY OF TRUNK INFECTED
912.1 ABRASION OR FRICTION BURN OF SHOULDER AND UPPER ARM INFECTED
912.3 BLISTER OF SHOULDER AND UPPER ARM INFECTED
912.5 INSECT BITE SHLD/ARM-INF (ICD9)
912.7 SUPERFICIAL FOREIGN BODY (SPLINTER) OF SHOULDER AND UPPER ARM WITHOUT MAJOR OPEN WOUND INFECTED
912.9 OTHER AND UNSPECIFIED SUPERFICIAL INJURY OF SHOULDER AND UPPER ARM INFECTED
913.1 ABRASION OR FRICTION BURN OF ELBOW FOREARM AND WRIST INFECTED
913.3 BLISTER OF ELBOW FOREARM AND WRIST INFECTED
913.5 INSECT BITE NONVENOMOUS OF ELBOW FOREARM AND WRIST INFECTED
913.7 SUPERFICIAL FOREIGN BODY (SPLINTER)OF ELBOW FOREARM AND WRIST WITHOUT MAJOR OPEN WOUND INFECTED
913.9 OTHER AND UNSPECIFIED SUPERFICIAL INJURY OF ELBOW FOREARM AND WRIST INFECTED

914.1 ABRASION OR FRICTION BURN OF HAND(S) EXCEPT FINGER(S) ALONE INFECTED
914.3 BLISTER OF HAND(S) EXCEPT FINGER(S) ALONE INFECTED
914.5 INSECT BITE HAND-INFECT (ICD9)
914.7 SUPERFICIAL FOREIGN BODY (SPLINTER) OF HAND(S) EXCEPT FINGER(S) ALONE WITHOUT MAJOR OPEN WOUND INFECTED
914.9 OTHER AND UNSPECIFIED SUPERFICIAL INJURY OF HAND(S) EXCEPT FINGER(S) ALONE INFECTED
915.1 ABRASION OR FRICTION BURN OF FINGERS INFECTED
915.3 BLISTER OF FINGERS INFECTED
915.5 INSECT BITE NONVENOMOUS OF FINGERS INFECTED
915.7 SUPERFICIAL FOREIGN BODY (SPLINTER) OF FINGERS WITHOUT MAJOR OPEN WOUND INFECTED
915.9 OTHER AND UNSPECIFIED SUPERFICIAL INJURY OF FINGERS INFECTED
916.1 ABRASION OR FRICTION BURN OF HIP THIGH LEG AND ANKLE INFECTED
916.3 BLISTER HIP & LEG-INFECT (ICD9)
916.5 INSECT BITE NONVENOMOUS OF HIP THIGH LEG AND ANKLE INFECTED
916.7 SUPERFICIAL FOREIGN BODY (SPLINTER) OF HIP THIGH LEG AND ANKLE WITHOUT MAJOR OPEN WOUND INFECTED
916.9 OTHER AND UNSPECIFIED SUPERFICIAL INJURY OF HIP THIGH LEG AND ANKLE INFECTED
917.1 ABRASION OR FRICTION BURN OF FOOT AND TOE(S) INFECTED
917.3 BLISTER OF FOOT AND TOE(S) INFECTED
917.5 INSECT BITE NONVENOMOUS OF FOOT AND TOE(S) INFECTED
917.7 SUPERFICIAL FOREIGN BODY (SPLINTER) OF FOOT AND TOE(S) WITHOUT MAJOR OPEN WOUND INFECTED
917.9 OTHER AND UNSPECIFIED SUPERFICIAL INJURY OF FOOT AND TOE(S) INFECTED
919.1 ABRASION OR FRICTION BURN OF OTHER MULTIPLE AND UNSPECIFIED SITES INFECTED
919.3 BLISTER NEC-INFECTED (ICD9) OF OTHER MULTIPLE AND UNSPECIFIED SITES INFECTED
919.5 INSECT BITE NONVENOMOUS OF OTHER MULTIPLE AND UNSPECIFIED SITES INFECTED
919.7 SUPERFICIAL FOREIGN BODY (SPLINTER) OF OTHER MULTIPLE AND UNSPECIFIED SITES WITHOUT MAJOR OPEN WOUND INFECTED
919.9 OTHER AND UNSPECIFIED SUPERFICIAL INJURY OF OTHER MULTIPLE AND UNSPECIFIED SITES INFECTED
958.3 POSTTRAUM WND INFEC NEC (ICD9)
996.60 INFECTION AND INFLAMMATORY REACTION DUE TO UNSPECIFIED DEVICE IMPLANT AND GRAFT
996.61 INFECTION AND INFLAMMATORY REACTION DUE TO CARDIAC DEVICE IMPLANT AND GRAFT
996.62 INFECTION AND INFLAMMATORY REACTION DUE TO VASCULAR DEVICE IMPLANT AND GRAFT
996.63 INFECTION AND INFLAMMATORY REACTION DUE TO NERVOUS SYSTEM DEVICE IMPLANT AND GRAFT
996.64 INFECTION AND INFLAMMATORY REACTION DUE TO INDWELLING URINARY CATHETER
996.65 INFECTION AND INFLAMMATORY REACTION DUE TO OTHER GENITOURINARY DEVICE IMPLANT AND GRAFT
996.66 INFECTION AND INFLAMMATORY REACTION DUE TO INTERNAL JOINT PROSTHESIS
996.67 INFECTION AND INFLAMMATORY REACTION DUE TO OTHER INTERNAL ORTHOPEDIC DEVICE IMPLANT AND GRAFT
996.68 INFECTION AND INFLAMMATORY REACTION DUE TO PERITONEAL DIALYSIS CATHETER
996.69 INFECTION AND INFLAMMATORY REACTION DUE TO OTHER INTERNAL PROSTHETIC DEVICE IMPLANT AND GRAT
997.09 (no description available)
997.62 INFECTION AMPUTAT STUMP (ICD9)
998.51 INFECTED POSTOP SEROMA (Begin 1996) (ICD9)
998.59 OTHER POSTOPERATIVE INFECTION
999.3 INFEC COMPL MED CARE NEC (end 2007) (ICD9)
999.31 OTHER AND UNSPEC INFECTION DUE TO CENTRAL VENOUS CATHETER

999.32 BLOODSTREAM INFECTION DUE TO CENTRAL VENOUS CATHETER
999.33 LOCAL INFECTION DUE TO CENTRAL VENOUS CATHETER
999.34 ACUTE INFECTION FOLLOWING TRANSFUSION INFUSION OR INJECTION OF BLOOD AND BLOOD PRODUCTS
999.39 INFECT FOL INFUS/INJ/VAC (Begin 2007) (ICD9)
V08 HIV POSITIVE NOS (Begin 1994) (ICD9)
V09.0 INFEC-PENICILLIN-RESIST ORGS (Begin 1993) (ICD9)
V09.1 INFEC-CEPHALOSPORIN-RESIST ORGS (Begin 1993) (ICD9)
V09.2 INFEC-MACROLIDES-RESIST ORGS (Begin 1993) (ICD9)
V09.3 INFEC-TETRACYCLINE-RESIST ORGS (Begin 1993) (ICD9)
V09.4 INFEC-AMINOGLYCOSIDE-RESIST ORGS (Begin 1993) (ICD9)
V09.50 INFEC-UNRESIST TO MULT QUINOLONES (Begin 1993) (ICD9)
V09.51 INFEC-MULT QUINOLONES-RESIST ORGS (Begin 1993) (ICD9)
V09.6 INFEC-SULFONAMIDES-RESIST ORGS (Begin 1993) (ICD9)
V09.70 INFEC-NOT MULTI-ANTIMYCOBACT-RESIS (Begin 1993) (ICD9)
V09.71 INFEC-MULTI-ANTIMYCOBAC-RESIS ORGS (Begin 1993) (ICD9)
V09.80 INFEC-OTH ORGS NOT MULTIDRUG-RESIS (Begin 1993) (ICD9)
V09.81 INFEC-OTH MULTI-DRUG-RESIST ORGS (Begin 1993) (ICD9)
V09.90 INFEC-UNSP ORGS NOT MULTDRUG-RESIS (Begin 1993) (ICD9)
V09.91 INFEC-UNSPEC MULTIDRUG-RESIST ORGS (Begin 1993) (ICD9)

A00.0 Cholera due to Vibrio cholerae 01, biovar cholerae (ICD10)
A00.1 Cholera due to Vibrio cholerae 01, biovar eltor (ICD10)
A00.9 Cholera, unspecified (ICD10)
A01.0 Typhoid fever (ICD10)
A01.1 Paratyphoid fever A (ICD10)
A01.2 Paratyphoid fever B (ICD10)
A01.3 Paratyphoid fever C (ICD10)
A01.4 Paratyphoid fever, unspecified (ICD10)
A02.0 Salmonella enteritis (ICD10)
A02.1 Salmonella sepsis (ICD10)
A02.2+ Localised salmonella infections (ICD10)
A02.8 Other specified salmonella infections (ICD10)
A02.9 Salmonella infection, unspecified (ICD10)
A03.0 Shigellosis due to Shigella dysenteriae (ICD10)
A03.1 Shigellosis due to Shigella flexneri (ICD10)
A03.2 Shigellosis due to Shigella boydii (ICD10)
A03.3 Shigellosis due to Shigella sonnei (ICD10)
A03.8 Other shigellosis (ICD10)
A03.9 Shigellosis, unspecified (ICD10)
A04.0 Enteropathogenic Escherichia coli infection (ICD10)
A04.1 Enterotoxigenic Escherichia coli infection (ICD10)
A04.2 Enteroinvasive Escherichia coli infection (ICD10)
A04.3 Enterohemorrhagic Escherichia coli infection (ICD10)
A04.4 Other intestinal Escherichia coli infections (ICD10)
A04.5 Campylobacter enteritis (ICD10)
A04.6 Enteritis due to Yersinia enterocolitica (ICD10)
A04.7 Enterocolitis due to Clostridium difficile (ICD10)
A04.8 Other specified bacterial intestinal infections (ICD10)
A04.9 Bacterial intestinal infection, unspecified (ICD10)
A05.0 Foodborne staphylococcal intoxication (ICD10)
A05.1 Botulism food poisoning (ICD10)
A05.2 Foodborne Clostridium perfringens [Clostridium welchii] intoxication (ICD10)
A05.3 Foodborne Vibrio parahaemolyticus intoxication (ICD10)
A05.4 Foodborne Bacillus cereus intoxication (ICD10)
A05.8 Other specified bacterial foodborne intoxications (ICD10)
A05.9 Bacterial foodborne intoxication, unspecified (ICD10)
A06.0 Acute amebic dysentery (ICD10)
A06.1 Chronic intestinal amebiasis (ICD10)

A06.2 Amebic nondysenteric colitis (ICD10)
A06.3 Amoeboma of intestine (ICD10)
A06.4 Amebic liver abscess (ICD10)
A06.5+ Amebic lung abscess (ICD10)
A06.6+ Amebic brain abscess (ICD10)
A06.7 Cutaneous amebiasis (ICD10)
A06.8 Amoebic infection of other sites (ICD10)
A06.9 Amebiasis, unspecified (ICD10)
A07.0 Balantidiasis (ICD10)
A07.1 Giardiasis [lambliasis] (ICD10)
A07.2 Cryptosporidiosis (ICD10)
A07.3 Isosporiasis (ICD10)
A07.8 Other specified protozoal intestinal diseases (ICD10)
A07.9 Protozoal intestinal disease, unspecified (ICD10)
A08.0 Rotaviral enteritis (ICD10)
A08.1 Acute gastroenteropathy due to Norwalk agent (ICD10)
A08.2 Adenoviral enteritis (ICD10)
A08.3 Other viral enteritis (ICD10)
A08.4 Viral intestinal infection, unspecified
A08.5 Other specified intestinal infections (ICD10)
A09 Infectious gastroenteritis and colitis, unspecified (ICD10)
A09.0 Other and unspecified gastroenteritis and colitis of infectious origin
A09.9 Gastroenteritis and colitis of unspecified origin
A15.0 Tuberculosis of lung (ICD10)
A15.1 Tuberculosis of lung, confirmed by culture only (ICD10)
A15.2 Tuberculosis of lung, confirmed histologically (ICD10)
A15.3 Tuberculosis of lung, confirmed by unspecified means (ICD10)
A15.4 Tuberculosis of intrathoracic lymph nodes (ICD10)
A15.5 Tuberculosis of larynx, trachea and bronchus (ICD10)
A15.6 Tuberculous pleurisy (ICD10)
A15.7 Primary respiratory tuberculosis (ICD10)
A15.8 Other respiratory tuberculosis (ICD10)
A15.9 Respiratory tuberculosis unspecified, confirmed bacteriologically and histologically (ICD10)
A16.0 Tuberculosis of lung, bacteriologically and histologically negative
A16.1 Tuberculosis of lung, bacteriological and histological examination not done (ICD10)
A16.2 Tuberculosis of lung, without mention of bacteriological or histological confirmation (ICD10)
A16.3 Tuberculosis of intrathoracic lymph nodes, without mention of bacteriological or histological confir (ICD10)
A16.4 Tuberculosis of larynx, trachea and bronchus, without mention of bacteriological or histological con (ICD10)
A16.5 Tuberculous pleurisy, without mention of bacteriological or histological confirmation (ICD10)
A16.7 Primary respiratory tuberculosis without mention of bacteriological or histological confirmation (ICD10)
A16.8 Other respiratory tuberculosis, without mention of bacteriological or histological confirmation (ICD10)
A16.9 Respiratory tuberculosis unspecified, without mention of bacteriological or histological confirmation
A17.0+ Tuberculous meningitis (ICD10)
A17.1+ Meningeal tuberculoma (ICD10)
A17.8+ Other tuberculosis of nervous system (ICD10)
A17.9+ Tuberculosis of nervous system, unspecified (ICD10)
A18.0+ Tuberculosis of bones and joints (ICD10)
A18.1 Tuberculosis of genitourinary system (ICD10)
A18.1+ Tuberculosis of genitourinary system (ICD10)
A18.2 Tuberculous peripheral lymphadenopathy (ICD10)
A18.3+ (no description found)
A18.4 Tuberculosis of skin and subcutaneous tissue (ICD10)
A18.5+ Tuberculosis of eye (ICD10)
A18.6+ Tuberculosis of (inner) (middle) ear (ICD10)
A18.7+ Tuberculosis of adrenal glands (ICD10)
A18.8+ Tuberculosis of other specified organs (ICD10)
A19.0 Acute miliary tuberculosis of a single specified site
A19.1 Acute miliary tuberculosis of multiple sites

A19.2 Acute miliary tuberculosis, unspecified (ICD10)
A19.8 Other miliary tuberculosis (ICD10)
A19.9 Miliary tuberculosis, unspecified (ICD10)
A20.0 Bubonic plague (ICD10)
A20.1 Cellulocutaneous plague (ICD10)
A20.2 Pneumonic plague (ICD10)
A20.3 Plague meningitis
A20.7 Septicemic plague (ICD10)
A20.8 Other forms of plague (ICD10)
A20.9 Plague, unspecified (ICD10)
A21.0 Ulceroglandular tularemia (ICD10)
A21.1 Oculoglandular tularemia (ICD10)
A21.2 Pulmonary tularemia (ICD10)
A21.3 Gastrointestinal tularemia (ICD10)
A21.7 Generalized tularaemia
A21.8 Other forms of tularemia (ICD10)
A21.9 Tularemia, unspecified (ICD10)
A22.0 Cutaneous anthrax (ICD10)
A22.1 Pulmonary anthrax (ICD10)
A22.2 Gastrointestinal anthrax (ICD10)
A22.7 Anthrax sepsis (ICD10)
A22.8 Other forms of anthrax (ICD10)
A22.9 Anthrax, unspecified (ICD10)
A23.0 Brucellosis due to Brucella melitensis (ICD10)
A23.1 Brucellosis due to Brucella abortus (ICD10)
A23.2 Brucellosis due to Brucella suis (ICD10)
A23.3 Brucellosis due to Brucella canis (ICD10)
A23.8 Other brucellosis (ICD10)
A23.9 Brucellosis, unspecified (ICD10)
A24.0 Glanders (ICD10)
A24.1 Acute and fulminating melioidosis
A24.2 Subacute and chronic melioidosis
A24.3 Other melioidosis
A24.4 Melioidosis, unspecified (ICD10)
A25.0 Spirillosis (ICD10)
A25.1 Streptobacillosis (ICD10)
A25.9 Rat-bite fever, unspecified (ICD10)
A26.0 Cutaneous erysipeloid
A26.7 Erysipelothrix sepsis
A26.8 Other forms of erysipeloid
A26.9 Erysipeloid, unspecified (ICD10)
A27.0 Leptospirosis icterohemorrhagica (ICD10)
A27.8 Other forms of leptospirosis (ICD10)
A27.9 Leptospirosis, unspecified (ICD10)
A28.0 Pasteurellosis (ICD10)
A28.1 Cat-scratch disease (ICD10)
A28.2 Extraintestinal yersiniosis
A28.8 Other specified zoonotic bacterial diseases, not elsewhere classified (ICD10)
A28.9 Zoonotic bacterial disease, unspecified (ICD10)
A30.0 Indeterminate leprosy (ICD10)
A30.1 Tuberculoid leprosy (ICD10)
A30.2 Borderline tuberculoid leprosy
A30.3 Borderline leprosy (ICD10)
A30.4 Borderline lepromatous leprosy
A30.5 Lepromatous leprosy (ICD10)
A30.8 Other forms of leprosy (ICD10)
A30.9 Leprosy, unspecified (ICD10)
A31.0 Pulmonary mycobacterial infection (ICD10)
A31.1 Cutaneous mycobacterial infection (ICD10)
A31.8 Other mycobacterial infections (ICD10)

A31.9 Mycobacterial infection, unspecified (ICD10)
A32.0 Cutaneous listeriosis
A32.1 Listerial meningitis and meningoencephalitis
A32.7 Listerial sepsis
A32.8 Other forms of listeriosis
A32.9 Listeriosis, unspecified (ICD10)
A33 Tetanus neonatorum (ICD10)
A34 Obstetrical tetanus
A35 Other tetanus (ICD10)
A36.0 Pharyngeal diphtheria (ICD10)
A36.1 Nasopharyngeal diphtheria (ICD10)
A36.2 Laryngeal diphtheria (ICD10)
A36.3 Cutaneous diphtheria (ICD10)
A36.8 Other diphtheria (ICD10)
A36.8+ (no description found)
A36.9 Diphtheria, unspecified (ICD10)
A37.0 Whooping cough due to Bordetella pertussis (ICD10)
A37.1 Whooping cough due to Bordetella parapertussis (ICD10)
A37.8 Whooping cough due to other Bordetella species (ICD10)
A37.9 Whooping cough, unspecified (ICD10)
A38 Scarlet fever (ICD10)
A39.0+ Meningococcal meningitis (ICD10)
A39.1+ Waterhouse-Friderichsen syndrome (ICD10)
A39.2 Acute meningococcaemia
A39.3 Chronic meningococcaemia
A39.4 Meningococcemia, unspecified (ICD10)
A39.5 Meningococcal heart disease
A39.8 Other meningococcal infections (ICD10)
A39.8+Other meningococcal infections
A39.9 Meningococcal infection, unspecified (ICD10)
A40.0 Sepsis due to streptococcus, group A
A40.1 Sepsis due to streptococcus, group B
A40.2 Sepsis due to streptococcus, group D
A40.3 Sepsis due to Streptococcus pneumoniae (ICD10)
A40.8 Other streptococcal sepsis
A40.9 Streptococcal sepsis, unspecified (ICD10)
A41.0 Sepsis due to Staphylococcus aureus
A41.1 Sepsis due to other specified staphylococcus
A41.2 Sepsis due to unspecified staphylococcus (ICD10)
A41.3 Sepsis due to Hemophilus influenzae (ICD10)
A41.4 Sepsis due to anaerobes (ICD10)
A41.51 Sepsis due to Escherichia coli [E. Coli] (ICD10)
A41.52 Sepsis due to Pseudomonas (ICD10)
A41.58 Sepsis due to other Gram-negative organisms (ICD10)
A41.8 Other specified septicaemia (ICD10)
A41.9 Sepsis, unspecified (ICD10)
A42.0 Pulmonary actinomycosis (ICD10)
A42.1 Abdominal actinomycosis (ICD10)
A42.2 Cervicofacial actinomycosis (ICD10)
A42.7 Actinomycotic sepsis
A42.8 Other forms of actinomycosis (ICD10)
A42.9 Actinomycosis, unspecified (ICD10)
A43.0 Pulmonary nocardiosis
A43.1 Cutaneous nocardiosis
A43.8 Other forms of nocardiosis
A43.9 Nocardiosis, unspecified
A44.0 Systemic bartonellosis
A44.1 Cutaneous and mucocutaneous bartonellosis
A44.8 Other forms of bartonellosis
A44.9 Bartonellosis, unspecified (ICD10)

A46 Erysipelas (ICD10)
A48.0 Gas gangrene (ICD10)
A48.1 Legionnaires disease
A48.2 Nonpneumonic Legionnaires disease
A48.3 Toxic shock syndrome
A48.4 Brazilian purpuric fever
A48.8 Other specified bacterial diseases (ICD10)
A49.0 Staphylococcal infection, unspecified site
A49.1 Streptococcal infection, unspecified site
A49.2 Haemophilus influenza infection, unspecified site
A49.3 Mycoplasma infection, unspecified site
A49.8 Other bacterial infections of unspecified site
A49.9 Bacterial infection, unspecified (ICD10)
A50.0 Early congenital syphilis, symptomatic (ICD10)
A50.1 Early congenital syphilis, latent (ICD10)
A50.2 Early congenital syphilis, unspecified (ICD10)
A50.3 Late congenital syphilitic oculopathy (ICD10)
A50.4 Late congenital neurosyphilis [juvenile neurosyphilis] (ICD10)
A50.5 Other late congenital syphilis, symptomatic (ICD10)
A50.6 Late congenital syphilis, latent (ICD10)
A50.7 Late congenital syphilis, unspecified (ICD10)
A50.9 Congenital syphilis, unspecified (ICD10)
A51.0 Primary genital syphilis (ICD10)
A51.1 Primary anal syphilis (ICD10)
A51.2 Primary syphilis of other sites (ICD10)
A51.3 Secondary syphilis of skin and mucous membranes (ICD10)
A51.3+ (no description found)
A51.4 Other secondary syphilis (ICD10)
A51.5 Early syphilis, latent (ICD10)
A51.9 Early syphilis, unspecified
A52.0+ Cardiovascular syphilis (I98.0*) (ICD10)
A52.1 Symptomatic neurosyphilis (ICD10)
A52.1+ (no description found)
A52.2 Asymptomatic neurosyphilis (ICD10)
A52.3 Neurosyphilis, unspecified
A52.7 Other symptomatic late syphilis (ICD10)
A52.7+ (no description found)
A52.8 Late syphilis, latent (ICD10)
A52.9 Late syphilis, unspecified (ICD10)
A53.0 Latent syphilis, unspecified as early or late (ICD10)
A53.9 Syphilis, unspecified (ICD10)
A54.0 Gonococcal infection of lower genitourinary tract without periurethral or accessory gland abscess (ICD10)
A54.1 Gonococcal infection of lower genitourinary tract with periurethral and accessory gland abscess (ICD10)
A54.2+ Gonococcal pelviperitonitis and other gonococcal genitourinary infections (ICD10)
A54.3 Gonococcal infection of eye (ICD10)
A54.3+ (no description found)
A54.4+ Gonococcal infection of musculoskeletal system (ICD10)
A54.5 Gonococcal pharyngitis (ICD10)
A54.6 Gonococcal infection of anus and rectum (ICD10)
A54.8 Other gonococcal infections (ICD10)
A54.8+ (no description found)
A54.9 Gonococcal infection, unspecified
A55 Chlamydial lymphogranuloma (venereum) (ICD10)
A56.0 Chlamydial infection of lower genitourinary tract (ICD10)
A56.1 Chlamydial infection of pelviperitoneum and other genitourinary organs (ICD10)
A56.2 Chlamydial infection of genitourinary tract, unspecified (ICD10)
A56.3 Chlamydial infection of anus and rectum (ICD10)
A56.4 Chlamydial infection of pharynx (ICD10)
A56.8 Sexually transmitted chlamydial infection of other sites (ICD10)

A57 Chancroid (ICD10)
A58 Granuloma inguinale (ICD10)
A59.0 Urogenital trichomoniasis (ICD10)
A59.0+ (no description found)
A59.8 Trichomoniasis of other sites (ICD10)
A59.9 Trichomoniasis, unspecified (ICD10)
A60.0 Herpesviral infection of genitalia and urogenital tract (ICD10)
A60.1 Herpesviral infection of perianal skin and rectum
A60.9 Anogenital herpesviral infection, unpsecified
A63.0 Anogenital (venereal) warts (ICD10)
A63.8 Other specified predominantly sexually transmitted diseases (ICD10)
A64 Unspecified sexually transmitted disease (ICD10)
A65 Nonvenereal syphilis (ICD10)
A66.0 Initial lesions of yaws (ICD10)
A66.1 Multiple papillomata and wet crab yaws (ICD10)
A66.2 Other early skin lesions of yaws (ICD10)
A66.3 Hyperkeratosis of yaws (ICD10)
A66.4 Gummata and ulcers of yaws (ICD10)
A66.5 Gangosa (ICD10)
A66.6 Bone and joint lesions of yaws (ICD10)
A66.7 Other manifestations of yaws (ICD10)
A66.8 Latent yaws (ICD10)
A66.9 Yaws, unspecified (ICD10)
A67.0 Primary lesions of pinta (ICD10)
A67.1 Intermediate lesions of pinta (ICD10)
A67.2 Late lesions of pinta
A67.3 Mixed lesions of pinta (ICD10)
A67.9 Pinta, unspecified (ICD10)
A68.0 Louse-borne relapsing fever (ICD10)
A68.1 Tick-borne relapsing fever (ICD10)
A68.9 Relapsing fever, unspecified (ICD10)
A69.0 Necrotizing ulcerative stomatitis
A69.1 Other Vincents infections (ICD10)
A69.2 Lyme disease (ICD10)
A69.8 Other specified spirochetal infections (ICD10)
A69.9 Spirochetal infection, unspecified (ICD10)
A70 Chlamydia psittaci infections (ICD10)
A70+ (no description found)
A71.0 Initial stage of trachoma (ICD10)
A71.1 Active stage of trachoma (ICD10)
A71.9 Trachoma, unspecified (ICD10)
A74.0+ Chlamydial conjunctivitis (ICD10)
A74.8 Other chlamydial diseases (ICD10)
A74.8+ (no description found)
A74.9 Chlamydial infection, unspecified (ICD10)
A75.0 Epidemic louse-borne typhus fever due to Rickettsia prowazekii
A75.1 Recrudescent typhus [Brills disease] (ICD10)
A75.2 Typhus fever due to Rickettsia typhi (ICD10)
A75.3 Typhus fever due to Rickettsia tsutsugamushi (ICD10)
A75.9 Typhus fever, unspecified (ICD10)
A77.0 Spotted fever due to Rickettsia rickettsii (ICD10)
A77.1 Spotted fever due to Rickettsia conorii (ICD10)
A77.2 Spotted fever due to Rickettsia siberica (ICD10)
A77.3 Spotted fever due to Rickettsia australis (ICD10)
A77.8 Other spotted fevers (ICD10)
A77.9 Spotted fever, unspecified (ICD10)
A78 Q fever (ICD10)
A79.0 Trench fever (ICD10)
A79.1 Rickettsialpox due to Rickettsia akari (ICD10)
A79.8 Other specified rickettsioses (ICD10)

A79.9 Rickettsiosis, unspecified (ICD10)
A80.1 Acute paralytic poliomyelitis, wild virus, imported
A80.2 Acute paralytic poliomyelitis, wild virus, indigenous
A80.3 Acute paralytic poliomyelitis, other and unspecified (ICD10)
A80.4 Acute nonparalytic poliomyelitis (ICD10)
A80.9 Acute poliomyelitis, unspecified (ICD10)
A81.0 Creutzfeldt-Jakob disease (ICD10)
A81.1 Subacute sclerosing panencephalitis (ICD10)
A81.2 Progressive multifocal leukoencephalopathy (ICD10)
A81.8 Other atypical virus infections of central nervous system (ICD10)
A81.9 Atypical virus infection of central nervous system, unspecified
A82.0 Sylvatic rabies
A82.1 Urban rabies
A82.9 Rabies, unspecified (ICD10)
A83.0 Japanese encephalitis (ICD10)
A83.1 Western equine encephalitis (ICD10)
A83.2 Eastern equine encephalitis (ICD10)
A83.3 St Louis encephalitis (ICD10)
A83.4 Australian encephalitis (ICD10)
A83.5 California encephalitis (ICD10)
A83.6 Rocio virus disease
A83.8 Other mosquito-borne viral encephalitis (ICD10)
A83.9 Mosquito-borne viral encephalitis, unspecified (ICD10)
A84.0 Far Eastern tick-borne encephalitis [Russian spring-summer encephalitis] (ICD10)
A84.1 Central European tick-borne encephalitis (ICD10)
A84.8 Other tick-borne viral encephalitis (ICD10)
A84.9 Tick-borne viral encephalitis, unspecified (ICD10)
A85.0 Enteroviral encephalitis
A85.1 Adenoviral encephalitis
A85.2 Arthropod-borne viral encephalitis, unspecified (ICD10)
A85.8 Other specified viral encephalitis
A86 Unspecified viral encephalitis
A87.0+ Enteroviral meningitis (ICD10)
A87.1+ Adenoviral meningitis (ICD10)
A87.2 Lymphocytic choriomeningitis (ICD10)
A87.8 Other viral meningitis
A87.9 Viral meningitis, unspecified
A88.0 Enteroviral exanthematous fever
A88.8 Other specified viral infections of central nervous system (ICD10)
A89 Unspecified viral infection of central nervous system (ICD10)
A90 Dengue fever [classical dengue] (ICD10)
A91 Dengue haemorrhagic fever
A92.0 Chikungunya virus disease
A92.1 O'nyong-nyong fever
A92.2 Venezuelan equine fever (ICD10)
A92.3 West Nile virus infection
A92.4 Rift Valley fever
A92.8 Other specified mosquito-borne viral fevers (ICD10)
A92.9 Mosquito-borne viral fever, unspecified
A93.0 Oropouche virus disease
A93.1 Sandfly fever (ICD10)
A93.2 Colorado tick fever (ICD10)
A93.8 Other specified arthropod-borne viral fevers (ICD10)
A94 Unspecified arthropod-borne viral fever (ICD10)
A95.0 Sylvatic yellow fever (ICD10)
A95.1 Urban yellow fever (ICD10)
A95.9 Yellow fever, unspecified (ICD10)
A96.0 Junin haemorrhagic fever
A96.1 Machupo haemorrhagic fever
A96.2 Lassa fever

A96.8 Other arenaviral hemorrhagic fevers (ICD10)
A96.9 Arenaviral haemorrhagic fever, unspecified
A98.0 Crimean-Congo hemorrhagic fever (ICD10)
A98.1 Omsk hemorrhagic fever (ICD10)
A98.2 Kyasanur Forest disease (ICD10)
A98.3 Marburg virus disease
A98.4 Ebola virus disease
A98.5 Hemorrhagic fever with renal syndrome (ICD10)
A98.8 Other specified viral haemorrhagic fevers
A99 Unspecified viral haemorrhagic fever
B00.0 Eczema herpeticum (ICD10)
B00.1 Herpesviral vesicular dermatitis (ICD10)
B00.2 Herpesviral gingivostomatitis and pharyngotonsillitis (ICD10)
B00.3+ Herpesviral meningitis (ICD10)
B00.4+ Herpesviral encephalitis (ICD10)
B00.5+ Herpesviral ocular disease (ICD10)
B00.7 Disseminated herpesviral disease (ICD10)
B00.8 Other forms of herpesviral infection (ICD10)
B00.9 Herpesviral infection, unspecified (ICD10)
B01.0 Varicella meningitis
B01.1+ Varicella encephalitis (G05.1*) (ICD10)
B01.2+ Varicella pneumonia (ICD10)
B01.8 Varicella with other complications (ICD10)
B01.9 Varicella without complication (ICD10)
B02.0 Zoster encephalitis
B02.1+ Zoster meningitis (ICD10)
B02.2+ Zoster with other nervous system involvement (ICD10)
B02.3+ Zoster ocular disease (ICD10)
B02.7 Disseminated zoster
B02.8 Zoster with other complications (ICD10)
B02.9 Zoster without complications (ICD10)
B03 Smallpox (ICD10)
B04 Monkeypox
B05.0+ Measles complicated by encephalitis (ICD10)
B05.1 Measles complicated by meningitis
B05.2+ Measles complicated by pneumonia (ICD10)
B05.3+ Measles complicated by otitis media (ICD10)
B05.4 Measles with intestinal complications
B05.8 Measles with other complications (ICD10)
B05.9 Measles without complication (ICD10)
B06.0+ Rubella with neurological complications (ICD10)
B06.8 Rubella with other complications (ICD10)
B06.9 Rubella without complication (ICD10)
B07 Viral warts (ICD10)
B08.0 Other orthopoxvirus infections (ICD10)
B08.1 Molluscum contagiosum (ICD10)
B08.2 Exanthema subitum [sixth disease]
B08.3 Erythema infectiosum [fifth disease] (ICD10)
B08.4 Enteroviral vesicular stomatitis with exanthem (ICD10)
B08.5 Enteroviral vesicular pharyngitis (ICD10)
B08.8 Other specified viral infections characterized by skin and mucous membrane lesions (ICD10)
B09 Unspecified viral infection characterized by skin and mucous membrane lesions (ICD10)
B15.0 Hepatitis A with hepatic coma (ICD10)
B15.9 Hepatitis A without hepatic coma (ICD10)
B16.0 Acute hepatitis B with delta-agent with hepatic coma (ICD10)
B16.1 Acute hepatitis B with delta-agent without hepatic coma (ICD10)
B16.2 Acute hepatitis B without delta-agent with hepatic coma (ICD10)
B16.9 Acute hepatitis B without delta-agent and without hepatic coma
B17.0 Acute delta-(super) infection of hepatitis B carrier (ICD10)
B17.1 Acute hepatitis C (ICD10)

B17.2 Acute hepatitis E (ICD10)
B17.8 Other specified acute viral hepatitis (ICD10)
B17.9 Acute viral hepatitis, unspecified
B18.0 Chronic viral hepatitis B with delta-agent (ICD10)
B18.1 Chronic viral hepatitis B without delta-agent (ICD10)
B18.2 Chronic viral hepatitis C (ICD10)
B18.8 Other chronic viral hepatitis
B18.9 Chronic viral hepatitis, unspecified
B19.0 Unspecified viral hepatitis with hepatic coma (ICD10)
B19.9 Unspecified viral hepatitis without hepatic coma (ICD10)
B20.0 HIV disease resulting in mycobacterial infection
B20.1 HIV disease resulting in other bacterial infections
B20.2 HIV disease resulting in cytomegaloviral disease
B20.3 HIV disease resulting in other viral infections
B20.4 HIV disease resulting in candidiasis
B20.5 HIV disease resulting in other mycoses
B20.6 HIV disease resulting in Pneumocystis jirovecii pneumonia
B20.7 HIV disease resulting in multiple infections
B20.8 HIV disease resulting in other infectious and parasitic diseases
B20.9 HIV disease resulting in unspecified infectious or parasitic disease
B21.0 HIV disease resulting in Kaposi sarcoma
B21.1 HIV disease resulting in Burkitt lymphoma
B21.2 HIV disease resulting in other types of non-Hodgkin lymphoma
B21.3 HIV disease resulting in other malignant neoplasms of lymphoid, haematopoietic and related tissue
B21.7 HIV disease resulting in multiple malignant neoplasms
B21.8 HIV disease resulting in other malignant neoplasms
B21.9 HIV disease resulting in unspecified malignant neoplasm
B22.0 HIV disease resulting in encephalopathy
B22.1 HIV disease resulting in lymphoid interstitial pneumonitis
B22.2 HIV disease resulting in wasting syndrome
B22.7 HIV disease resulting in multiple diseases classified elsewhere
B23.0 Acute HIV infection syndrome
B23.1 HIV disease resulting in (persistent) generalized lymphadenopathy
B23.2 HIV disease resulting in haematological and immunological abnormalities, not elsewhere classified
B23.8 HIV disease resulting in other specified conditions
B24 Unspecified human immunodeficiency virus [HIV] disease (ICD10)
B25.0 Cytomegaloviral pneumonitis
B25.1 Cytomegaloviral hepatitis
B25.2 Cytomegaloviral pancreatitis
B25.8 Other cytomegaloviral diseases
B25.9 Cytomegaloviral disease, unspecified (ICD10)
B26.0+ Mumps orchitis (ICD10)
B26.1+ Mumps meningitis (ICD10)
B26.2+ Mumps encephalitis (ICD10)
B26.3+ Mumps pancreatitis (ICD10)
B26.8 Mumps with other complications (ICD10)
B26.8+ (no description found)
B26.9 Mumps without complication (ICD10)
B27.0 Gammaherpesviral mononucleosis
B27.1 Cytomegaloviral mononucleosis
B27.8 Other infectious mononucleosis
B27.9 Infectious mononucleosis, unspecified (ICD10)
B30.0+ Keratoconjunctivitis due to adenovirus (ICD10)
B30.1+ Conjunctivitis due to adenovirus (ICD10)
B30.2+ (no description found)
B30.3+ Acute epidemic hemorrhagic conjunctivitis (enteroviral)
B30.8+ Other viral conjunctivitis (ICD10)
B30.9 Viral conjunctivitis, unspecified
B33.0 Epidemic myalgia (ICD10)
B33.1 Ross River disease

B33.2 Viral carditis
B33.3 Retrovirus infections, not elsewhere classified (ICD10)
B33.4 Hantavirus (cardio-) pulmonary syndrome
B33.8 Other specified viral diseases (ICD10)
B34.0 Adenovirus infection, unspecified (ICD10)
B34.1 Enterovirus infection, unspecified (ICD10)
B34.2 Coronavirus infection, unspecified site
B34.3 Parvovirus infection, unspecified site
B34.4 Papovavirus infection, unspecified (ICD10)
B34.8 Other viral infections of unspecified site (ICD10)
B34.9 Viral infection, unspecified (ICD10)
B35.0 Tinea barbae and tinea capitis (ICD10)
B35.1 Tinea unguium (ICD10)
B35.2 Tinea manuum (ICD10)
B35.3 Tinea pedis (ICD10)
B35.4 Tinea corporis (ICD10)
B35.5 Tinea imbricata
B35.6 Tinea cruris (ICD10)
B35.8 Other dermatophytoses (ICD10)
B35.9 Dermatophytosis, unspecified (ICD10)
B36.0 Pityriasis versicolor (ICD10)
B36.1 Tinea nigra (ICD10)
B36.2 White piedra (ICD10)
B36.3 Black piedra (ICD10)
B36.8 Other specified superficial mycoses (ICD10)
B36.9 Superficial mycosis, unspecified (ICD10)
B37.0 Candidal stomatitis (ICD10)
B37.1 Pulmonary candidiasis (ICD10)
B37.2 Candidiasis of skin and nail (ICD10)
B37.3+ Candidiasis of vulva and vagina (ICD10)
B37.4+ Candidiasis of other urogenital sites (ICD10)
B37.5+ Candidal meningitis (ICD10)
B37.6 Candidal endocaritis
B37.7 Candidal sepsis
B37.81 Candidal oesophagitis (ICD10)
B37.88 Candidiasis of other sites (ICD10)
B37.9 Candidiasis, unspecified (ICD10)
B38.0 Acute pulmonary coccidioidomycosis (ICD10)
B38.1 Chronic pulmonary coccidioidomycosis (ICD10)
B38.2 Pulmonary coccidioidomycosis, unspecified (ICD10)
B38.3 Cutaneous coccidioidomycosis (ICD10)
B38.4+ Coccidioidomycosis meningitis (ICD10)
B38.7 Disseminated coccidioidomycosis
B38.8 Other forms of coccidioidomycosis (ICD10)
B38.9 Coccidioidomycosis, unspecified (ICD10)
B39.0 Acute pulmonary histoplasmosis capsulati
B39.1 Chronic pulmonary histoplasmosis capsulati
B39.2 Pulmonary histoplasmosis capsulati, unspecified (ICD10)
B39.3 Disseminated histoplasmosis capsulati
B39.4 Histoplasmosis capsulati, unspecified (ICD10)
B39.5 Histoplasmosis duboisii (ICD10)
B39.9 Histoplasmosis, unspecified (ICD10)
B40.0 Acute pulmonary blastomycosis
B40.1 Chronic pulmonary blatomycosis
B40.2 Pulmonary blastomycosis, unspecified
B40.3 Cutaneous blastomycosis
B40.7 Disseminated blastomycosis
B40.8 Other forms of blastomycosis
B40.9 Blastomycosis, unspecified (ICD10)
B41.0 Pulmonary paracoccidioidomycosis

B41.7 Disseminated paracoccidioidomycosis
B41.8 Other forms of paracoccidioidomycosis
B41.9 Paracoccidioidomycosis, unspecified (ICD10)
B42.0+ Pulmonary sporotrichosis (ICD10)
B42.1 Lymphocutaneous sporotrichosis
B42.7 Disseminated sporotrichosis
B42.8 Other forms of sporotrichosis
B42.9 Sporotrichosis, unspecified
B43.0 Cutaneous chromomycosis
B43.1 Phaeomycotic brain abscess
B43.2 Subcutaneous phaeomycotic abscess and cyst
B43.8 Other forms of chromomycosis
B43.9 Chromomycosis, unspecified (ICD10)
B44.0 Invasive pulmonary aspergillosis
B44.1 Other pulmonary aspergillosis
B44.2 Tonsillary aspergillosis
B44.7 Disseminated aspergillosis
B44.8 Other forms of aspergillosis
B44.9 Aspergillosis, unspecified (ICD10)
B45.0 Pulmonary cryptococcosis
B45.1 Cerebral cryptococcosis
B45.2 Cutaneous cryptococcosis
B45.3 Osseous cryptococcosis
B45.7 Disseminated cryptococcosis
B45.8 Other forms of cryptococcosis
B45.9 Cryptococcosis, unspecified (ICD10)
B46.0 Pulmonary mucormycosis
B46.1 Rhinocerebral mucormycosis
B46.2 Gastrointestinal mucormycosis
B46.3 Cutaneous mucormycosis
B46.4 Disseminated mucormycosis
B46.5 Mucormycosis, unspecified
B46.8 Other zygomycoses
B46.9 Zygomycosis, unspecified (ICD10)
B47.0 Eumycetoma (ICD10)
B47.1 Actinomycetoma
B47.9 Mycetoma, unspecified (ICD10)
B48.0 Lobomycosis (ICD10)
B48.1 Rhinosporidiosis (ICD10)
B48.2 Allescheriasis (ICD10)
B48.3 Geotrichosis
B48.4 Penicillosis
B48.7 Opportunistic mycoses (ICD10)
B48.8 Other specified mycoses (ICD10)
B49 Unspecified mycosis
B50.0 Plasmodium falciparum malaria with cerebral complications
B50.8 Other severe and complicated Plasmodium falciparum malaria (ICD10)
B50.9 Plasmodium falciparum malaria, unspecified (ICD10)
B51.0 Plasmodium vivax malaria with rupture of spleen
B51.8 Plasmodium vivax malaria with other complications
B51.9 Plasmodium vivax malaria without complication (ICD10)
B52.0 Plasmodium malariae malaria with nephropathy
B52.8 Plasmodium malariae malaria with other complications
B52.9 Plasmodium malariae malaria without complication (ICD10)
B53.0 Plasmodium ovale malaria (ICD10)
B53.1 Malaria due to simian plasmodia
B53.8 Other malaria, not elsewhere classified (ICD10)
B54 Unspecified malaria (ICD10)
B55.0 Visceral leishmaniasis (ICD10)
B55.1 Cutaneous leishmaniasis (ICD10)

B55.2 Mucocutaneous leishmaniasis (ICD10)
B55.9 Leishmaniasis, unspecified (ICD10)
B56.0 Gambiense trypanosomiasis (ICD10)
B56.1 Rhodesiense trypanosomiasis (ICD10)
B56.9 African trypanosomiasis, unspecified (ICD10)
B57.0 Acute Chagas disease with heart involvement
B57.1 Acute Chagas disease without heart involvement (ICD10)
B57.2 Chagas disease (chronic) with heart involvement (ICD10)
B57.3 Chagas disease (chronic) with digestive system involvement
B57.4 Chagas disease (chronic) with nervous system involvement
B57.5 Chagasdisease (chronic) with other organ involvement (ICD10)
B58.0+ Toxoplasma oculopathy (ICD10)
B58.1+ Toxoplasma hepatitis (ICD10)
B58.2+ Toxoplasma meningoencephalitis (ICD10)
B58.3+ Pulmonary toxoplasmosis (ICD10)
B58.8 Toxoplasmosis with other organ involvement (ICD10)
B58.9 Toxoplasmosis, unspecified (ICD10)
B59 Pneumocystosis (ICD10)
B60.0 Babesiosis (ICD10)
B60.1 Acanthamoebiasis
B60.2 Naegleriasis
B60.8 Other specified protozoal diseases
B64 Unspecified protozoal disease
B65.0 Schistosomiasis due to Schistosoma haematobium [urinary schistosomiasis] (ICD10)
B65.1 Schistosomiasis due to Schistosoma mansoni [intestinal schistosomiasis] (ICD10)
B65.2 Schistosomiasis due to Schistosoma japonicum (ICD10)
B65.3 Cercarial dermatitis (ICD10)
B65.8 Other schistosomiasis (ICD10)
B65.9 Schistosomiasis, unspecified (ICD10)
B66.0 Opisthorchiasis (ICD10)
B66.1 Clonorchiasis (ICD10)
B66.2 Dicrocoeliasis
B66.3 Fascioliasis (ICD10)
B66.4 Paragonimiasis (ICD10)
B66.5 Fasciolopsiasis (ICD10)
B66.8 Other specified fluke infections (ICD10)
B66.9 Fluke infection, unspecified (ICD10)
B67.0 Echinococcus granulosus infection of liver (ICD10)
B67.1 Echinococcus granulosus infection of lung (ICD10)
B67.2 Echinococcus granulosus infection of bone
B67.3 Echinococcus granulosus infection, other and multiple sites (ICD10)
B67.3+ (no description found)
B67.4 Echinococcus granulosus infection, unspecified (ICD10)
B67.5 Echinococcus multilocularis infection of liver (ICD10)
B67.6 Echinococcus multilocularis infection, other and multiple sites
B67.7 Echinococcus multilocularis infection, unspecified (ICD10)
B67.8 Echinococcosis, unspecified, of liver (ICD10)
B67.9 Echinococcosis, other and unspecified (ICD10)
B68.0 Taenia solium taeniasis (ICD10)
B68.1 Taenia saginata taeniasis (ICD10)
B68.9 Taeniasis, unspecified (ICD10)
B69.0 Cysticercosis of central nervous system
B69.1 Cysticercosis of eye
B69.8 Cysticercosis of other sites
B69.9 Cysticercosis, unspecified (ICD10)
B70.0 Diphyllobothriasis (ICD10)
B70.1 Sparganosis (ICD10)
B71.0 Hymenolepiasis (ICD10)
B71.1 Dipylidiasis
B71.8 Other specified cestode infections (ICD10)

B71.9 Cestode infection, unspecified (ICD10)
B72 Dracunculiasis (ICD10)
B73 Onchocerciasis (ICD10)
B74.0 Filariasis due to Wuchereria bancrofti (ICD10)
B74.1 Filariasis due to Brugia malayi (ICD10)
B74.2 Filariasis due to Brugia timori
B74.3 Loiasis (ICD10)
B74.4 Mansonelliasis (ICD10)
B74.8 Other filariases (ICD10)
B74.9 Filariasis, unspecified (ICD10)
B75 Trichinellosis (ICD10)
B76.0 Ancylostomiasis (ICD10)
B76.1 Necatoriasis (ICD10)
B76.8 Other hookworm diseases
B76.9 Hookworm disease, unspecified
B77.0 Ascariasis with intestinal complications
B77.8 Ascariasis with other complications
B77.9 Ascariasis, unspecified (ICD10)
B78.0 Intestinal strongyloidiasis
B78.1 Cutaneous strongyloidiasis
B78.7 Disseminated strongyloidiasis
B78.9 Strongyloidiasis, unspecified (ICD10)
B79 Trichuriasis (ICD10)
B80 Enterobiasis (ICD10)
B81.0 Anisakiasis (ICD10)
B81.1 Intestinal capillariasis (ICD10)
B81.2 Trichostrongyliasis (ICD10)
B81.3 Intestinal angiostrongyliasis
B81.4 Mixed intestinal helminthiases (ICD10)
B81.8 Other specified intestinal helminthiases (ICD10)
B82.0 Intestinal helminthiasis, unspecified (ICD10)
B82.9 Intestinal parasitism, unspecified (ICD10)
B83.0 Visceral larva migrans (ICD10)
B83.1 Gnathostomiasis (ICD10)
B83.2 Angiostrongyliasis due to Parastrongylus cantonensis
B83.3 Syngamiasis
B83.4 Internal hirudiniasis
B83.8 Other specified helminthiases (ICD10)
B83.9 Helminthiasis, unspecified (ICD10)
B85.0 Pediculosis due to Pediculus humanus capitis (ICD10)
B85.1 Pediculosis due to Pediculus humanus corporis (ICD10)
B85.2 Pediculosis, unspecified (ICD10)
B85.3 Phthiriasis (ICD10)
B85.4 Mixed pediculosis and phthiriasis (ICD10)
B86 Scabies (ICD10)
B87.0 Cutaneous myiasis
B87.1 Wound myiasis
B87.2 Ocular myiasis
B87.3 Nasopharyngeal myiasis
B87.4 Aural myiasis
B87.8 Myiasis of other sites
B87.9 Myiasis, unspecified (ICD10)
B88.0 Other acariasis (ICD10)
B88.1 Tungiasis [sandflea infestation] (ICD10)
B88.2 Other arthropod infestations (ICD10)
B88.3 External hirudiniasis (ICD10)
B88.8 Other specified infestations (ICD10)
B88.9 Infestation, unspecified (ICD10)
B89 Unspecified parasitic disease (ICD10)
B95.0 Streptococcus, group A, as the cause of diseases classified elsewhere (ICD10)

B95.1 Streptococcus, group B, as the cause of diseases classified elsewhere (ICD10)
B95.2 Streptococcus, group D, as the cause of diseases classified elsewhere (ICD10)
B95.3 Streptococcus pneumonia as the cause of diseases classified elsewhere
B95.41 Streptococcus, group C, as the cause of diseases classified to other chapters (ICD10)
B95.42 Streptococcus, group G, as the cause of diseases classified to other chapters (ICD10)
B95.48 Streptococcus, other specified group, as the cause of diseases classified to other chapters (ICD10)
B95.5 Unspecified streptococcus as the cause of diseases classified to other chapters
B95.6 Staphylococcus aureus as the cause of diseases classified elsewhere
B95.7 Other staphylococcus as the cause of diseases classified elsewhere
B95.8 Unspecified staphylococcus as the cause of diseases classified elsewhere (ICD10)
B96.0 Mycoplasma pneumonia as the cause of diseases classified to other chapters
B96.1 Klebsiella pneumonia as the cause of diseases classified to other chapters
B96.2 Escherichia coli [E. coli] as the cause of diseases classified elsewhere (ICD10)
B96.3 Hemophilus influenzae [H. influenzae] as the cause of diseases classified elsewhere (ICD10)
B96.4 Proteus (mirabilis) (morganii) as the cause of diseases classified elsewhere (ICD10)
B96.5 Pseudomonas (aeruginosa) (mallei) (pseudomallei) as the cause of diseases classified elsewhere (ICD10)
B96.6 Bacteroides fragilis [B. fragilis] as the cause of diseases classified elsewhere (ICD10)
B96.7 Clostridium perfringens [C. perfringens] as the cause of diseases classified elsewhere (ICD10)
B96.81 Helicobacter pylori [H. pylori] as the cause of diseases classified to other chapters (ICD10)
B96.88 Other and unspecified bacterial agents as the cause of diseases classified to other chapters (ICD10)
B97.0 Adenovirus as the cause of diseases classified to other chapters
B97.1 Enterovirus as the cause of diseases classified to other chapters
B97.2 Coronavirus as the cause of diseases classified to other chapters
B97.3 Retrovirus as the cause of diseases classified to other chapters
B97.4 Respiratory syncytial virus as the cause of diseases classified to other chapters
B97.5 Reovirus as the cause of diseases classified to other chapters
B97.6 Parvovirus as the cause of diseases classified to other chapters
B97.7 Papillomavirus as the cause of diseases classified to other chapters
B97.8 Other viral agents as the cause of diseases classified to other chapters
B98.0 Helicobacter pylori as the cause of diseases classified to other chapters
B98.1 Vibrio vulnificus as the cause of diseases classified to other chapters
B99 Other and unspecified infectious diseases (ICD10)
D59.3 Haemolytic-uraemic syndrome
G00.0 Haemophilus meningitis (ICD10)
G00.1 Pneumococcal meningitis (ICD10)
G00.1* (no description found)
G00.2 Streptococcal meningitis (ICD10)
G00.3 Staphylococcal meningitis (ICD10)
G00.8 Other bacterial meningitis (ICD10)
G00.9 Bacterial meningitis, unspecified (ICD10)
G01 Meningitis in bacterial diseases classified elsewhere
G02.0* Meningitis in viral diseases classified elsewhere (ICD10)
G02.1* Meningitis in mycoses (ICD10)
G02.8* Meningitis in other specified infectious and parasitic diseases classified elsewhere (ICD10)
G03.0 Nonpyogenic meningitis (ICD10)
G03.1 Chronic meningitis (ICD10)
G03.2 Benign recurrent meningitis
G03.8 Meningitis due to other specified causes
G03.9 Meningitis, unspecified (ICD10)
G04.0 Acute disseminated encephalitis (ICD10)
G04.1 Tropical spastic paraplegia
G04.2 Bacterial meningoencephalitis and meningomyelitis, not elsewhere classified
G04.8 Other encephalitis, myelitis and encephalomyelitis (ICD10)
G04.9 Encephalitis, myelitis and encephalomyelitis, unspecified (ICD10)
G05.0 Encephalitis, myelitis and encephalomyelitis in bacterial diseases classified elsewhere
G05.1* Encephalitis, myelitis and encephalomyelitis in viral diseases classified elsewhere (ICD10)
G05.2* Encephalitis, myelitis and encephalomyelitis in other infectious and parasitic diseases classified e (ICD10)

G05.8 Encephalitis, myelitis and encephalomyelitis in other diseases classified elsewhere
G06.0 Intracranial abscess and granuloma (ICD10)
G06.1 Intraspinal abscess and granuloma (ICD10)
G06.2 Extradural and subdural abscess, unspecified (ICD10)
G07 Intracranial and intraspinal abscess and granuloma in disease classified elsewhere (ICD10)
G08 Intracranial and intraspinal phlebitis and thrombophlebitis
G92 Toxic encephalopathy (ICD10)
G94.0 Hydrocephalus in infectious and parasitic diseases classified elsewhere
H00.0 Hordeolum and other deep inflammation of eyelid (ICD10)
H00.1 Chalazion (ICD10)
H01.0 Blepharitis (ICD10)
H01.8 Other specified inflammations of eyelid (ICD10)
H03.0* Parasitic infestation of eyelid in diseases classified elsewhere (ICD10)
H03.1* Involvement of eyelid in other infectious diseases classified elsewhere (ICD10)
H04.0 Dacryoadenitis (ICD10)
H06.1* Parasitic infestation of orbit in diseases classified elsewhere (ICD10)
H10.0 Mucopurulent conjunctivitis (ICD10)
H10.2 Other acute conjunctivitis (ICD10)
H10.3 Acute conjunctivitis, unspecified (ICD10)
H10.5 Blepharoconjunctivitis (ICD10)
H13.0 Filarial infection of conjunctiva
H13.1* Conjunctivitis in infectious and parasitic diseases classified elsewhere (ICD10)
H19.0 Scleritis and episcleritis in diseases classified elsewhere
H19.1 Herpesviral keratitis and keratoconjunctivitis
H19.2* Keratitis and keratoconjunctivitis in other infectious and parasitic diseases classified elsewhere (ICD10)
H20.0 Acute and subacute iridocyclitis (ICD10)
H22.0 Iridocyclitis in infectious and parasitic diseases classified elsewhere
H32.0 Chorioretinal inflammation in infectious and parasitic diseases classified elsewhere
H44.0 Purulent endophthalmitis (ICD10)
H44.1 Other endophthalmitis (ICD10)
H45.1 Endophthalmitis in diseases classified elsewhere
H48.1 Retrobulbar neuritis in diseases classified elsewhere
H58.8 Other specified diseases of eye and adnexa in diseases classified elsewhere
H60.0 Abscess of external ear
H60.1 Cellulitis of external ear
H60.2 Malignant otitis externa
H60.3 Other infective otitis externa (ICD10)
H62.0 Otitis externa in bacterial diseases classified elsewhere
H62.1 Otitis externa in viral diseases classified elsewhere
H62.2* Otitis externa in mycoses (ICD10)
H62.3 Otitis externa in other infectious and parasitic diseases classified elsewhere
H65.0 Acute serous otitis media (ICD10)
H65.1 Other acute nonsuppurative otitis media (ICD10)
H65.2 Chronic serous otitis media (ICD10)
H65.3 Chronic mucoid otitis media (ICD10)
H65.4 Other chronic nonsuppurative otitis media (ICD10)
H65.9 Nonsuppurative otitis media, unspecified (ICD10)
H66.0 Acute suppurative otitis media (ICD10)
H66.1 Chronic tubotympanic suppurative otitis media (ICD10)
H66.2 Chronic atticoantral suppurative otitis media (ICD10)
H66.3 Other chronic suppurative otitis media (ICD10)
H66.4 Suppurative otitis media, unspecified (ICD10)
H66.9 Otitis media, unspecified (ICD10)
H67.0 Otitis media in bacterial diseases classified elsewhere
H67.1 Otitis media in viral diseases classified elsewhere
H68.0 Eustachian salpingitis
H70.0 Acute mastoiditis (ICD10)
H70.1 Chronic mastoiditis (ICD10)
H70.2 Petrositis (ICD10)

H70.8 Other mastoiditis and related conditions (ICD10)
H70.9 Mastoiditis, unspecified (ICD10)
H72.0 Central perforation of tympanic membrane (ICD10)
H72.1 Attic perforation of tympanic membrane (ICD10)
H72.2 Other marginal perforations of tympanic membrane (ICD10)
H72.8 Other perforations of tympanic membrane (ICD10)
H72.9 Perforation of tympanic membrane, unspecified (ICD10)
H73.0 Acute myringitis
H75.0 Mastoiditis in infectious and parasitic diseases classified elsewhere
H83.0 Labyrinthitis
H92.1 Otorrhoea
H94.0 Acoustic neuritis in infectious and parasitic diseases classified elsewhere
I00 Rheumatic fever without heart involvement (ICD10)
I01.0 Acute rheumatic pericarditis
I01.1 Acute rheumatic endocarditis
I01.2 Acute rheumatic myocarditis
I01.8 Other acute rheumatic heart disease
I01.9 Acute rheumatic heart disease, unspecified
I30.1 Infective pericarditis
I32.0 Pericarditis in bacterial diseases classified elsewhere
I32.1 Pericarditis in other infectious and parasitic diseases classified elsewhere
I33.0 Acute and subacute infective endocarditis
I39.0 Mitral valve disorders in diseases classified elsewhere
I39.1 Aortic valve disorders in diseases classified elsewhere
I39.2 Tricuspid valve disorders in diseases classified elsewhere
I39.3 Pulmonary valve disorders in diseases classified elsewhere
I39.4 Multiple valve disorders in diseases classified elsewhere
I39.8 Endocarditis, valve unspecified, in diseases classified elsewhere
I40.0 Infective myocarditis
I41.0 Myocarditis in bacterial diseases classified elsewhere
I41.1 Myocarditis in viral diseases classified elsewhere
I41.2 Myocarditis in other infectious and parasitic diseases classified elsewhere
I43.0 Cardiomyopathy in diseases classified elsewhere
I52.0 Other heart disorders in bacterial diseases classified elsewhere
I52.1 Other heart disorders in other infectious and parasitic diseases classified elsewhere
I68.1 Cerebral arteritis in infectious and parasitic diseases classified elsewhere
I98.0 Cardiovascular syphilis
I98.1 Cardiovascular disorders in other infectious and parasitic diseases classified elsewhere
J00 Acute nasopharyngitis [common cold] (ICD10)
J01.0 Acute maxillary sinusitis (ICD10)
J01.1 Acute frontal sinusitis (ICD10)
J01.2 Acute ethmoidal sinusitis (ICD10)
J01.3 Acute sphenoidal sinusitis (ICD10)
J01.4 Acute pansinusitis
J01.8 Other acute sinusitis (ICD10)
J01.9 Acute sinusitis, unspecified (ICD10)
J02.0 Streptococcal pharyngitis (ICD10)
J02.8 Acute pharyngitis due to other specified organisms
J02.9 Acute pharyngitis, unspecified (ICD10)
J03.0 Streptococcal tonsillitis
J03.8 Acute tonsillitis due to other specified organisms
J03.9 Acute tonsillitis, unspecified (ICD10)
J04.0 Acute laryngitis (ICD10)
J04.1 Acute tracheitis (ICD10)
J04.2 Acute laryngotracheitis (ICD10)
J05.0 Acute obstructive laryngitis [croup] (ICD10)
J05.1 Acute epiglottitis (ICD10)
J06.0 Acute laryngopharyngitis (ICD10)
J06.8 Other acute upper respiratory infections of multiple sites (ICD10)
J06.9 Acute upper respiratory infection, unspecified (ICD10)

J09 Influenza due to certain identified influenza virus
J10.0 Influenza with pneumonia, influenza virus identified (ICD10)
J10.1 Influenza due to other influenza virus with respiratory manifestations (ICD10)
J10.8 Influenza with other manifestations, influenza virus identified
J11 Influenza, virus not identified (ICD10)
J11.0 Influenza with pneumonia, virus not identified (ICD10)
J11.1 Influenza with other respiratory manifestations, virus not identified (ICD10)
J11.8 Influenza with other manifestations, virus not identified (ICD10)
J12.0 Adenoviral pneumonia (ICD10)
J12.1 Respiratory syncytial virus pneumonia (ICD10)
J12.2 Parainfluenza virus pneumonia (ICD10)
J12.3 Human metapneumovirus pneumonia
J12.8 Other viral pneumonia (ICD10)
J12.9 Viral pneumonia, unspecified (ICD10)
J13 Pneumonia due to Streptococcus pneumoniae (ICD10)
J14 Pneumonia due to Hemophilus influenzae (ICD10)
J15.0 Pneumonia due to Klebsiella pneumoniae (ICD10)
J15.1 Pneumonia due to Pseudomonas (ICD10)
J15.2 Pneumonia due to staphylococcus (ICD10)
J15.3 Pneumonia due to streptococcus, group B (ICD10)
J15.4 Pneumonia due to other streptococci (ICD10)
J15.5 Pneumonia due to Escherichia coli (ICD10)
J15.6 Pneumonia due to other aerobic Gram-negative bacteria (ICD10)
J15.7 Pneumonia due to Mycoplasma pneumoniae (ICD10)
J15.8 Pneumonia due to other specified bacteria (ICD10)
J15.9 Unspecified bacterial pneumonia (ICD10)
J16.0 Chlamydial pneumonia
J16.8 Pneumonia due to other specified infectious organisms (ICD10)
J17.0* Pneumonia in bacterial diseases classified elsewhere (ICD10)
J17.1* Pneumonia in viral diseases classified elsewhere (ICD10)
J17.2* Pneumonia in mycoses (ICD10)
J17.3 Pneumonia in parasitic diseases
J17.8* Pneumonia in other diseases classified elsewhere (ICD10)
J18.0 Bronchopneumonia, unspecified organism (ICD10)
J18.1 Lobar pneumonia, unspecified
J18.8 Other pneumonia, unspecified organism (ICD10)
J18.9 Pneumonia, unspecified
J20.0 Acute bronchitis due to Mycoplasma pneumoniae (ICD10)
J20.1 Acute bronchitis due to Hemophilus influenzae (ICD10)
J20.2 Acute bronchitis due to streptococcus (ICD10)
J20.3 Acute bronchitis due to coxsackievirus (ICD10)
J20.4 Acute bronchitis due to parainfluenza virus (ICD10)
J20.5 Acute bronchitis due to respiratory syncytial virus (ICD10)
J20.6 Acute bronchitis due to rhinovirus (ICD10)
J20.7 Acute bronchitis due to echovirus (ICD10)
J20.8 Acute bronchitis due to other specified organisms (ICD10)
J20.9 Acute bronchitis, unspecified (ICD10)
J21.0 Acute bronchiolitis due to respiratory syncytial virus
J21.1 Acute bronchiolitis due to human megapneumovirus
J21.8 Acute bronchiolitis due to other specified organisms
J21.9 Acute bronchiolitis, unspecified (ICD10)
J22 Unspecified acute lower respiratory infection (ICD10)
J32.0 Chronic maxillary sinusitis (ICD10)
J32.1 Chronic frontal sinusitis (ICD10)
J32.2 Chronic ethmoidal sinusitis (ICD10)
J32.3 Chronic sphenoidal sinusitis (ICD10)
J32.4 Chronic pansinusitis
J32.8 Other chronic sinusitis (ICD10)
J32.9 Chronic sinusitis, unspecified (ICD10)
J34.0 Abscess, furuncle and carbuncle of nose

J35.0 Chronic tonsillitis (ICD10)
J36 Peritonsillar abscess (ICD10)
J39.0 Retropharyngeal and parapharyngeal abscess (ICD10)
J39.1 Other abscess of pharynx (ICD10)
J39.2 Other diseases of pharynx (ICD10)
J40 Bronchitis, not specified as acute or chronic (ICD10)
J41.0 Simple chronic bronchitis (ICD10)
J41.1 Mucopurulent chronic bronchitis (ICD10)
J41.8 Mixed simple and mucopurulent chronic bronchitis
J42 Unspecified chronic bronchitis (ICD10)
J44.0 Chronic obstructive pulmonary disease with acute lower respiratory infection
J47 Bronchiectasis (ICD10)
J65 Pneumoconiosis associated with tuberculosis
J85.0 Gangrene and necrosis of lung
J85.1 Abscess of lung with pneumonia
J85.2 Abscess of lung without pneumonia (ICD10)
J85.3 Abscess of mediastinum
J86.0 Pyothorax with fistula (ICD10)
J86.9 Pyothorax without fistula (ICD10)
J99.8 Respiratory disorders in other diseases classified elsewhere
K04.4 Acute apical periodontitis of pulpal origin (ICD10)
K04.5 Chronic apical periodontitis (ICD10)
K04.6 Periapical abscess with sinus (ICD10)
K04.7 Periapical abscess without sinus (ICD10)
K05.2 Acute periodontitis (ICD10)
K05.3 Chronic periodontitis (ICD10)
K05.4 Periodontosis (ICD10)
K11.3 Abscess of salivary gland (ICD10)
K12.2 Cellulitis and abscess of mouth (ICD10)
K52.8 Other specified noninfective gastroenteritis and colitis (ICD10)
K61.0 Anal abscess
K61.1 Rectal abscess
K61.2 Anorectal abscess
K61.3 Ischiorectal abscess
K61.4 Intrashincteric abscess
K63.0 Abscess of intestine (ICD10)
K65.0 Generalized (acute) peritonitis (ICD10)
K65.8 Other peritonitis (ICD10)
K65.9 Peritonitis, unspecified (ICD10)
K67.0 Chlamydial peritonitis
K67.1 Gonococcal peritonitis
K67.2 Syphilitic peritonitis
K67.3 Tuberculous peritonitis
K67.8* Other disorders of peritoneum in infectious diseases classified elsewhere (ICD10)
K75.0 Abscess of liver (ICD10)
K75.1 Phlebitis of portal vein (ICD10)
K77.0* Liver disorders in infectious and parasitic diseases classified elsewhere (ICD10)
K81.0 Acute cholecystitis
K87.1 Disorders of pancreas in diseases classified elsewhere
K90.8+ (no description found)
K93.0 Tuberculous disorders of intestines, peritoneum and mesenteric glands
K93.1 Megacolon in Chagas disease
L00 Staphylococcal scalded skin syndrome
L01.0 Impetigo [any organism] [any site] (ICD10)
L01.1 Impetiginization of other dermatoses
L02.0 Cutaneous abscess, furuncle and carbuncle of face (ICD10)
L02.1 Cutaneous abscess, furuncle and carbuncle of neck (ICD10)
L02.2 Cutaneous abscess, furuncle and carbuncle of trunk (ICD10)
L02.3 Cutaneous abscess, furuncle and carbuncle of buttock (ICD10)
L02.4 Cutaneous abscess, furuncle and carbuncle of limb (ICD10)

L02.8 Cutaneous abscess, furuncle and carbuncle of other sites (ICD10)
L02.9 Cutaneous abscess, furuncle and carbuncle, unspecified (ICD10)
L03.0 Cellulitis of finger and toe
L03.01 Cellulitis of finger (ICD10)
L03.02 Cellulitis of toe (ICD10)
L03.1 Cellulitis of other parts of limb
L03.10 Cellulitis of upper limb (ICD10)
L03.11 Cellulitis of lower limb (ICD10)
L03.2 Cellulitis of face (ICD10)
L03.3 Cellulitis of trunk (ICD10)
L03.8 Cellulitis of other sites (ICD10)
L03.9 Cellulitis, unspecified (ICD10)
L04.0 Acute lymphadenitis of face, head and neck
L04.1 Acute lymphadenitis of trunk
L04.2 Acute lymphadenitis of upper limb
L04.3 Acute lymphadenitis of lower limb
L04.8 Acute lymphadenitis of other sites
L04.9 Acute lymphadenitis, unspecified (ICD10)
L05.0 Pilonidal cyst with abscess (ICD10)
L05.9 Pilonidal cyst without abscess (ICD10)
L08.0 Pyoderma (ICD10)
L08.1 Erythrasma
L08.8 Other specified local infections of skin and subcutaneous tissue
L08.9 Local infection of the skin and subcutaneous tissue, unspecified
L13.0 Dermatitis herpetiformis (ICD10)
L30.3 Infective dermatitis
L54.0 Erythema marginatum in acute rheumatic fever
L88 Pyoderma gangrenosum
M00.0 Staphylococcal arthritis and polyarthritis
M00.1 Pneumococcal arthritis and polyarthritis
M00.2 Other streptococcal arthritis and polyarthritis
M00.8 Arthritis and polyarthritis due to other specified bacterial agents
M00.90 Pyogenic arthritis, unspecified, multiple sites (ICD10)
M00.91 Pyogenic arthritis, unspecified, shoulder region (ICD10)
M00.92 Pyogenic arthritis, unspecified, upper arm (ICD10)
M00.93 Pyogenic arthritis, unspecified, forearm (ICD10)
M00.94 Pyogenic arthritis, unspecified, hand (ICD10)
M00.95 Pyogenic arthritis, unspecified, pelvic region and thigh
M00.96 Pyogenic arthritis, unspecified, lower leg (ICD10)
M00.97 Pyogenic arthritis, unspecified, ankle and foot (ICD10)
M00.98 Pyogenic arthritis, unspecified, other site (ICD10)
M00.99 Pyogenic arthritis, unspecified, site unspecified (ICD10)
M01.0 Meningococcal arthritis
M01.1 Tuberculous arthritis
M01.2 Arthritis in Lyme disease
M01.30* Arthritis in other bacterial diseases classified elsewhere, multiple sites (ICD10)
M01.31* Arthritis in other bacterial diseases classified elsewhere, shoulder region (ICD10)
M01.32* Arthritis in other bacterial diseases classified elsewhere, upper arm (ICD10)
M01.33* Arthritis in other bacterial diseases classified elsewhere, forearm (ICD10)
M01.34* Arthritis in other bacterial diseases classified elsewhere, hand (ICD10)
M01.35* Arthritis in other bacterial diseases classified elsewhere, pelvic region and thigh (ICD10)
M01.36* Arthritis in other bacterial diseases classified elsewhere, lower leg (ICD10)
M01.37* Arthritis in other bacterial diseases classified elsewhere, ankle and foot (ICD10)
M01.38* Arthritis in other bacterial diseases classified elsewhere, other site (ICD10)
M01.39* Arthritis in other bacterial diseases classified elsewhere, site unspecified (ICD10)
M01.4 Rubella arthritis
M01.50* Arthritis in other viral diseases classified elsewhere, multiple sites (ICD10)
M01.51* Arthritis in other viral diseases classified elsewhere, shoulder region (ICD10)
M01.52* Arthritis in other viral diseases classified elsewhere, upper arm (ICD10)
M01.53* Arthritis in other viral diseases classified elsewhere, forearm (ICD10)

M01.54* Arthritis in other viral diseases classified elsewhere, hand
M01.55* Arthritis in other viral diseases classified elsewhere, pelvic region and thigh (ICD10)
M01.56* Arthritis in other viral diseases classified elsewhere, lower leg (ICD10)
M01.57* Arthritis in other viral diseases classified elsewhere, ankle and foot (ICD10)
M01.58* Arthritis in other viral diseases classified elsewhere, other site (ICD10)
M01.59* Arthritis in other viral diseases classified elsewhere, site unspecified (ICD10)
M01.60* Arthritis in mycoses, multiple sites (B35-B49+) (ICD10)
M01.61* Arthritis in mycoses, shoulder region (B35-B49+) (ICD10)
M01.62* Arthritis in mycoses, upper arm (B35-B49+) (ICD10)
M01.63* Arthritis in mycoses, forearm (B35-B49+) (ICD10)
M01.64* Arthritis in mycoses, hand (B35-B49+) (ICD10)
M01.65* Arthritis in mycoses, pelvic region and thigh (B35-B49+)
M01.66 Arthritis in mycoses, lower leg (B35-B49+) (ICD10)
M01.67* Arthritis in mycoses, ankle and foot (B35-B49+) (ICD10)
M01.68 Arthritis in mycoses, other site (B35-B49+) (ICD10)
M01.69* Arthritis in mycoses, site unspecified (B35-B49+) (ICD10)
M01.80* Arthritis in other infectious and parasitic diseases classified elsewhere, multiple sites (ICD10)
M01.81* Arthritis in other infectious and parasitic diseases classified elsewhere, shoulder region (ICD10)
M01.82* Arthritis in other infectious and parasitic diseases classified elsewhere, upper arm (ICD10)
M01.83 Arthritis in other infectious and parasitic diseases classified elsewhere, forearm (ICD10)
M01.83* Arthritis in other infectious and parasitic diseases classified elsewhere, forearm (ICD10)
M01.84* Arthritis in other infectious and parasitic diseases classified elsewhere, hand (ICD10)
M01.85* Arthritis in other infectious and parasitic diseases classified elsewhere, pelvic region and thigh (ICD10)
M01.86* Arthritis in other infectious and parasitic diseases classified elsewhere, lower leg (ICD10)
M01.87* Arthritis in other infectious and parasitic diseases classified elsewhere, ankle and foot (ICD10)
M01.88* Arthritis in other infectious and parasitic diseases classified elsewhere, other site (ICD10)
M01.89* Arthritis in other infectious and parasitic diseases classified elsewhere, site unspecified (ICD10)
M46.2 Osteomyelitis of vertebra
M46.3 Infection of intervertebral disc (pyogenic)
M46.4 Discitis, unspecified
M46.5 Other infective spondylopathies
M49.0 Tuberculosis of spine
M49.1 Brucella spondylitis
M49.2 Enterobacterial spondylitis
M49.3 Spondylopathy in other infectious and parasitic diseases classified elsewhere
M60.0 Infective myositis
M60.09 Infective myositis, multiple sites (ICD10)
M63.0 Myositis in bacterial diseases classified elsewhere
M63.1 Myositis in protozoal and parasitic infections classified elsewhere
M63.2 Myositis in other infectious diseases classified elsewhere
M65.0 Abscess of tendon sheath
M65.1 Other infective (teno)synovitis
M68.0 Synovitis and tenosynovitis in bacterial diseases classified elsewhere
M71.0 Abscess of bursa
M71.1 Other infective bursitis
M72.6 Necrotizing fasciitis
M72.8 Other fibroblastic disorders
M73.0 Gonococcal bursitis
M73.1 Syphilitic bursitis
M86.0 Acute haematogenous osteomyelitis
M86.10 Other acute osteomyelitis, unspecified site (ICD10)
M86.11 Other acute osteomyelitis, shoulder region (ICD10)
M86.12 Other acute osteomyelitis, upper arm (ICD10)
M86.13 Other acute osteomyelitis, forearm (ICD10)
M86.14 Other acute osteomyelitis, hand (ICD10)
M86.15 Other acute osteomyelitis, pelvic region and thigh (ICD10)
M86.16 Other acute osteomyelitis, lower leg (ICD10)
M86.17 Other acute osteomyelitis, ankle and foot (ICD10)
M86.18 Other acute osteomyelitis, other site (ICD10)

M86.19 Other acute osteomyelitis, multiple sites (ICD10)
M86.2 Subacute osteomyelitis
M86.4 Chronic osteomyelitis with draining sinus
M86.5 Other chronic osteomyelitis
M86.60 Other chronic osteomyelitis, unspecified site (ICD10)
M86.67 Other chronic osteomyelitis, ankle and foot (ICD10)
M86.68 Other chronic osteomyelitis, other site (ICD10)
M86.69 Other chronic osteomyelitis, multiple sites (ICD10)
M86.8 Other osteomyelitis
M86.90 Unspecified osteomyelitis, multiple sites (ICD10)
M86.91 Unspecified osteomyelitis, shoulder region (ICD10)
M86.92 Unspecified osteomyelitis, upper arm (ICD10)
M86.93 Unspecified osteomyelitis, forearm (ICD10)
M86.94 Unspecified osteomyelitis, hand (ICD10)
M86.95 Unspecified osteomyelitis, pelvic region and thigh (ICD10)
M86.96 Unspecified osteomyelitis, lower leg (ICD10)
M86.97 Unspecified osteomyelitis, ankle and foot (ICD10)
M86.98 Unspecified osteomyelitis, other site (ICD10)
M86.99 Unspecified osteomyelitis, site unspecified (ICD10)
M90.0 Tuberculosis of bone
M90.1 Periostitis in other infectious diseases classified elsewhere
N08.0 Glomerular disorders in infectious and parasitic diseases classified elsewhere
N08.8 Glomerular disorders in other diseases classified elsewhere
N13.6 Pyonephrosis
N15.1 Renal and perinephric abscess (ICD10)
N15.9 Renal tubulo-interstitial disease, unspecified
N16.0 Renal tubulo-interstitial disorders in infectious and parasitic diseases classified elsewhere
N22.0 Urinary calculus in schistosomiasis
N29.1 Other disorders of kidney and ureter in infectious and parasitic diseases classified elsewhere
N30.0 Acute cystitis
N30.8 Other cystitis, abscess of bladder (ICD10)
N33.0 Tuberculous cystitis
N33.8 Bladder disorders in other diseases classified elsewhere
N34.0 Urethral abscess (ICD10)
N35.1 Postinfective urethral stricture, not elsewhere classified (ICD10)
N39.0 Urinary tract infection, site not specified (ICD10)
N41.0 Acute prostatitis (ICD10)
N41.2 Abscess of prostate (ICD10)
N43.1 Infected hydrocele (ICD10)
N45.0 Orchitis, epididymitis and epididymo-orchitis with abscess (ICD10)
N45.9 Orchitis, epididymitis and epididymo-orchits without abscess
N51.0 Disorders of prostate in diseases classified elsewhere
N51.1 Disorders of testis and epididymis in diseases classified elsewhere
N51.2 Balanitis in diseases classified elsewhere
N51.8 Other disorders of male genital organs in diseases classified elsewhere
N61 Inflammatory disorders of breast
N70.0 Acute salpingitis and oophoritis (ICD10)
N73.0 Acute parametritis and pelvic cellulitis (ICD10)
N73.1 Chronic parametritis and pelvic cellulitis (ICD10)
N73.2 Unspecified parametritis and pelvic cellulitis
N73.3 Female acute pelvic peritonitis (ICD10)
N73.4 Female chronic pelvic peritonitis (ICD10)
N73.5 Female pelvic peritonitis, unspecified
N73.6 Female pelvic peritoneal adhesions (postinfective) (ICD10)
N73.8 Other specified female pelvic inflammatory diseases (ICD10)
N73.9 Female pelvic inflammatory disease, unspecified (ICD10)
N74.0 Tuberculous infection of cervix uteri
N74.1 Female tuberculous pelvic inflammatory disease
N74.2 Female syphilitic pelvic inflammatory disease
N74.3 Female gonococcal pelvic inflammatory disease

N74.4 Female chlamydial pelvic inflammatory disease
N74.8 Female pelvic inflammatory disorders in other diseases classified elsewhere
N75.0 Cyst of Bartholins gland (ICD10)
N75.1 Abscess of Bartholins gland (ICD10)
N76.0 Acute vaginitis (ICD10)
N76.4 Abscess of vulva (ICD10)
N76.6 Ulceration of vulva (ICD10)
N76.8 Other specified inflammation of vagina and vulva (ICD10)
N77.0 Ulceration of vulva in infectious and parasitic diseases classified elsewhere
N77.1* Vaginitis, vulvitis and vulvovaginitis in infectious and parasitic diseases classified elsewhere (ICD10)
O03.0 Spontaneous abortion, incomplete, complicated by genital tract and pelvic infection (ICD10)
O03.5 Spontaneous abortion, complete or unspecified, complicated by genital tract and pelvic infection (ICD10)
O04.0 Medical abortion, incomplete, complicated by genital tract and pelvic infection (ICD10)
O04.5 Medical abortion, complete or unspecified, complicated by genital tract and pelvic infection (ICD10)
O05.0 Other abortion, incomplete, complicated by genital tract and pelvic infection (ICD10)
O05.5 Other abortion, complete or unspecified, complicated by genital tract and pelvic infection (ICD10)
O06.0 Unspecified abortion, incomplete, complicated by genital tract and pelvic infection (ICD10)
O06.5 Unspecified abortion, complete or unspecified, complicated by genital tract and pelvic infection (ICD10)
O07.0 Failed medical abortion, complicated by genital tract and pelvic infection
O07.5 Other and unspecified failed attempted abortion, complicated by genital tract and pelvic infection
O08.0 Genital tract and pelvic infection following ectopic and molar pregnancy (ICD10)
O23.0 Infections of kidney in pregnancy
O23.1 Infections of bladder in pregnancy
O23.2 Infections of urethra in pregnancy
O23.3 Infections of other parts of urinary tract in pregnancy
O23.4 Unspecified infection of urinary tract in pregnancy
O23.5 Infections of the genital tract in pregnancy
O23.9 Other and unspecified genitourinary tract infection in pregnancy
O26.4 Herpes gestationis
O41.1 Infection of amniotic sac and membranes (ICD10)
O75.3 Other infection during labor (ICD10)
O85 Puerperal sepsis (ICD10)
O86.0 Infection of obstetric surgical wound
O86.1 Other infection of genital tract following delivery
O86.2 Urinary tract infection following delivery
O86.3 Other genitourinary tract infections following delivery
O86.4 Pyrexia of unknown origin following delivery
O86.8 Other specified puerperal infections
O91.00 Infection of nipple associated with childbirth, without mention of attachment difficulty (ICD10)
O91.10 Abscess of breast associated with childbirth, without mention of attachment difficulty (ICD10)
O91.20 Nonpurulent mastitis associated with childbirth, without mention of attachment difficulty (ICD10)
O98.0 Tuberculosis complicating pregnancy, childbirth and the puerperium
O98.1 Syphilis complicating pregnancy, childbirth and the puerperium
O98.2 Gonorrhea complicating pregnancy, childbirth and the puerperium
O98.3 Other infections with a predominantly sexual mode of transmission complicating pregnancy, childbirth and the puerperium
O98.4 Viral hepatitis complicating pregnancy, childbirth and the puerperium
O98.5 Other viral diseases complicating pregnancy, childbirth and the puerperium (ICD10)
O98.6 Protozoal diseases complicating pregnancy, childbirth and the puerperium (ICD10)
O98.7 Human immunodeficiency disease complicating pregnancy, childbirth and the puerperium
O98.8 Other maternal infectious and parasitic diseases complicating pregnancy, childbirth and the puerperi (ICD10)
O98.9 Unspecified maternal infectious or parasitic disease complicating pregnancy, childbirth and the puer (ICD10)
P00.2 Newborn (suspected to be) affected by maternal infectious and parasitic diseases (ICD10)
P02.7 Newborn (suspected to be) affected by chorioamnionitis (ICD10)
P23.0 Congenital pneumonia due to viral agent
P23.1 Congenital pneumonia due to Chlamydia

P23.2 Congenital pneumonia due to staphylococcus
P23.3 Congenital pneumonia due to staphylococcus, group B
P23.4 Congenital pneumonia due to Escherichia coli
P23.5 Congenital pneumonia due to Pseudomonas
P23.6 Congenital pneumonia due to other bacterial agents
P23.8 Congenital pneumonia due to other organisms
P23.9 Congenital pneumonia, unspecified
P35.0 Congenital rubella syndrome
P35.1 Congenital cytomegalovirus infection
P35.2 Congenital herpesviral [herpes simples] infection
P35.3 Congenital viral hepatitis
P35.8 Other congenital viral diseases
P35.9 Congenital viral disease, unspecified
P36.0 Sepsis of newborn due to streptococcus, group B
P36.1 Sepsis of newborn due to other and unspecified streptococci
P36.2 Sepsis of newborn due to Staphylococcus aureus
P36.3 Sepsis of newborn due to other and unspecified staphylococci
P36.4 Sepsis of newborn due to Escherichia coli
P36.5 Sepsis of newborn due to anaerobes
P36.8 Other bacterial sepsis of newborn
P36.9 Bacterial sepsis of newborn, unspecified
P37.0 Congenital tuberculosis
P37.1 Congenital toxoplasmosis
P37.2 Neonatal (disseminated) listeriosis
P37.3 Congenital falciparum malaria
P37.4 Other congenital malaria
P37.5 Neonatal candidiasis (ICD10)
P37.8 Other specified congenital infectious and parasitic diseases
P37.9 Congenital infectious and parasitic disease, unspecified
P38 Omphalitis of newborn with or without mild haemorrhage (ICD10)
P39.0 Neonatal infective mastitis (ICD10)
P39.1 Neonatal conjunctivitis and dacryocystitis (ICD10)
P39.2 Intra-amniotic infection of fetus, not elsewhere classified
P39.3 Neonatal urinary tract infection
P39.4 Neonatal skin infection
P39.8 Other specified infections specific to the perinatal period (ICD10)
P39.9 Infection specific to the perinatal period, unspecified
Z21 Asymptomatic human immunodeficiency virus [HIV] infection status

Supplementary Table: ICD codes for invasive bacterial infection diagnostic group

003.1	SALMONELLA SEPTICEMIA (ICD9)
003.21	SALMONELLA MENINGITIS (ICD9)
003.23	SALMONELLA ARTHRITIS (ICD9)
003.24	SALMONELLA OSTEOMYELITIS (ICD9)
006.5	AMEBIC BRAIN ABSCESS (ICD9)
013.00	TB MENINGITIS-UNSPEC (ICD9)
013.01	TB MENINGITIS-NO EXAM (ICD9)
013.02	TB MENINGITIS-EXAM UNKN (ICD9)
013.03	TB MENINGITIS-MICRO DX (ICD9)
013.04	TB MENINGITIS-CULT DX (ICD9)
013.05	TB MENINGITIS-HISTO DX (ICD9)
013.06	TB MENINGITIS-OTH TEST (ICD9)
013.10	TUBRCLMA MENINGES-UNSPEC (ICD9)
013.11	TUBRCLMA MENING-NO EXAM (ICD9)
013.12	TUBRCLMA MENIN-EXAM UNKN (ICD9)
013.13	TUBRCLMA MENING-MICRO DX (ICD9)
013.14	TUBRCLMA MENING-CULT DX (ICD9)
013.15	TUBRCLMA MENING-HISTO DX (ICD9)
013.16	TUBRCLMA MENING-OTH TEST (ICD9)
013.20	TUBERCULOMA BRAIN-UNSPEC (ICD9)
013.21	TUBRCLOMA BRAIN-NO EXAM (ICD9)
013.22	TUBRCLMA BRAIN-EXAM UNKN (ICD9)
013.23	TUBRCLOMA BRAIN-MICRO DX (ICD9)
013.24	TUBRCLOMA BRAIN-CULT DX (ICD9)
013.25	TUBRCLOMA BRAIN-HISTO DX (ICD9)
013.26	TUBRCLOMA BRAIN-OTH TEST (ICD9)
013.30	TB BRAIN ABSCESS-UNSPEC (ICD9)
013.31	TB BRAIN ABSCESS-NO EXAM (ICD9)
013.32	TB BRAIN ABSC-EXAM UNKN (ICD9)
013.33	TB BRAIN ABSC-MICRO DX (ICD9)
013.34	TB BRAIN ABSCESS-CULT DX (ICD9)
013.35	TB BRAIN ABSC-HISTO DX (ICD9)
013.36	TB BRAIN ABSC-OTH TEST (ICD9)
013.40	TUBRCLMA SP CORD-UNSPEC (ICD9)
013.41	TUBRCLMA SP CORD-NO EXAM (ICD9)
013.42	TUBRCLMA SP CD-EXAM UNKN (ICD9)
013.43	TUBRCLMA SP CRD-MICRO DX (ICD9)
013.44	TUBRCLMA SP CORD-CULT DX (ICD9)
013.45	TUBRCLMA SP CRD-HISTO DX (ICD9)
013.46	TUBRCLMA SP CRD-OTH TEST (ICD9)
013.50	TB SP CRD ABSCESS-UNSPEC (ICD9)
013.51	TB SP CRD ABSC-NO EXAM (ICD9)
013.52	TB SP CRD ABSC-EXAM UNKN (ICD9)
013.53	TB SP CRD ABSC-MICRO DX (ICD9)
013.54	TB SP CRD ABSC-CULT DX (ICD9)
013.55	TB SP CRD ABSC-HISTO DX (ICD9)
013.56	TB SP CRD ABSC-OTH TEST (ICD9)
013.60	TB ENCEPHALITIS-UNSPEC (ICD9)
013.61	TB ENCEPHALITIS-NO EXAM (ICD9)
013.62	TB ENCEPHALIT-EXAM UNKN (ICD9)
013.63	TB ENCEPHALITIS-MICRO DX (ICD9)
013.64	TB ENCEPHALITIS-CULT DX (ICD9)
013.65	TB ENCEPHALITIS-HISTO DX (ICD9)
013.66	TB ENCEPHALITIS-OTH TEST (ICD9)
013.80	CNS TB NEC-UNSPEC (ICD9)
013.81	CNS TB NEC-NO EXAM (ICD9)
013.82	CNS TB NEC-EXAM UNKN (ICD9)
013.83	CNS TB NEC-MICRO DX (ICD9)

013.84 CNS TB NEC-CULT DX (ICD9)
013.85 CNS TB NEC-HISTO DX (ICD9)
013.86 CNS TB NEC-OTH TEST (ICD9)
013.90 CNS TB NOS-UNSPEC (ICD9)
013.91 CNS TB NOS-NO EXAM (ICD9)
013.92 CNS TB NOS-EXAM UNKN (ICD9)
013.93 CNS TB NOS-MICRO DX (ICD9)
013.94 CNS TB NOS-CULT DX (ICD9)
013.95 CNS TB NOS-HISTO DX (ICD9)
013.96 CNS TB NOS-OTH TEST (ICD9)
015.00 TB OF VERTEBRA-UNSPEC (ICD9)
015.01 TB OF VERTEBRA-NO EXAM (ICD9)
015.02 TB OF VERTEBRA-EXAM UNKN (ICD9)
015.03 TB OF VERTEBRA-MICRO DX (ICD9)
015.04 TB OF VERTEBRA-CULT DX (ICD9)
015.05 TB OF VERTEBRA-HISTO DX (ICD9)
015.06 TB OF VERTEBRA-OTH TEST (ICD9)
015.10 TB OF HIP-UNSPEC (ICD9)
015.11 TB OF HIP-NO EXAM (ICD9)
015.12 TB OF HIP-EXAM UNKN (ICD9)
015.13 TB OF HIP-MICRO DX (ICD9)
015.14 TB OF HIP-CULT DX (ICD9)
015.15 TB OF HIP-HISTO DX (ICD9)
015.16 TB OF HIP-OTH TEST (ICD9)
015.20 TB OF KNEE-UNSPEC (ICD9)
015.21 TB OF KNEE-NO EXAM (ICD9)
015.22 TB OF KNEE-EXAM UNKN (ICD9)
015.23 TB OF KNEE-MICRO DX (ICD9)
015.24 TB OF KNEE-CULT DX (ICD9)
015.25 TB OF KNEE-HISTO DX (ICD9)
015.26 TB OF KNEE-OTH TEST (ICD9)
015.50 TB OF LIMB BONES-UNSPEC (ICD9)
015.51 TB LIMB BONES-NO EXAM (ICD9)
015.52 TB LIMB BONES-EXAM UNKN (ICD9)
015.53 TB LIMB BONES-MICRO DX (ICD9)
015.54 TB LIMB BONES-CULT DX (ICD9)
015.55 TB LIMB BONES-HISTO DX (ICD9)
015.56 TB LIMB BONES-OTH TEST (ICD9)
015.60 TB OF MASTOID-UNSPEC (ICD9)
015.61 TB OF MASTOID-NO EXAM (ICD9)
015.62 TB OF MASTOID-EXAM UNKN (ICD9)
015.63 TB OF MASTOID-MICRO DX (ICD9)
015.64 TB OF MASTOID-CULT DX (ICD9)
015.65 TB OF MASTOID-HISTO DX (ICD9)
015.66 TB OF MASTOID-OTH TEST (ICD9)
015.70 TB OF BONE NEC-UNSPEC (ICD9)
015.71 TB OF BONE NEC-NO EXAM (ICD9)
015.72 TB OF BONE NEC-EXAM UNKN (ICD9)
015.73 TB OF BONE NEC-MICRO DX (ICD9)
015.74 TB OF BONE NEC-CULT DX (ICD9)
015.75 TB OF BONE NEC-HISTO DX (ICD9)
015.76 TB OF BONE NEC-OTH TEST (ICD9)
015.80 TB OF JOINT NEC-UNSPEC (ICD9)
015.81 TB OF JOINT NEC-NO EXAM (ICD9)
015.82 TB JOINT NEC-EXAM UNKN (ICD9)
015.83 TB OF JOINT NEC-MICRO DX (ICD9)
015.84 TB OF JOINT NEC-CULT DX (ICD9)
015.85 TB OF JOINT NEC-HISTO DX (ICD9)
015.86 TB OF JOINT NEC-OTH TEST (ICD9)
015.90 TB BONE/JOINT NOS-UNSPEC (ICD9)

015.91 TB BONE/JT NOS-NO EXAM (ICD9)
015.92 TB BONE/JT NOS-EXAM UNKN (ICD9)
015.93 TB BONE/JT NOS-MICRO DX (ICD9)
015.94 TB BONE/JT NOS-CULT DX (ICD9)
015.95 TB BONE/JT NOS-HISTO DX (ICD9)
015.96 TB BONE/JT NOS-OTH TEST (ICD9)
020.0 BUBONIC PLAGUE (ICD9)
020.2 SEPTICEMIC PLAGUE (ICD9)
020.8 OTHER TYPES OF PLAGUE (ICD9)
020.9 PLAGUE NOS (ICD9)
021.8 TULAREMIA NEC (ICD9)
021.9 TULAREMIA NOS (ICD9)
022.3 ANTHRAX SEPTICEMIA (ICD9)
023.0 BRUCELLA MELITENSIS (ICD9)
023.1 BRUCELLA ABORTUS (ICD9)
023.2 BRUCELLA SUIS (ICD9)
023.3 BRUCELLA CANIS (ICD9)
023.8 BRUCELLOSIS NEC (ICD9)
023.9 BRUCELLOSIS NOS (ICD9)
025 MELIOIDOSIS (ICD9)
026.0 SPIRILLARY FEVER (ICD9)
026.1 STREPTOBACILLARY FEVER (ICD9)
026.9 RAT-BITE FEVER NOS (ICD9)
027.0 LISTERIOSIS (ICD9)
031.2 DX DUE TO DISSEM MYCOBACT (Begin 1997) (ICD9)
036.1 MENINGOCOCC ENCEPHALITIS (ICD9)
036.2 MENINGOCOCCEMIA (ICD9)
036.3 MENINGOCOCC ADRENAL SYND (ICD9)
036.40 MENINGOCOCCAL CARDITIS UNSPECIFIED
036.41 MENINGOCOCCAL PERICARDITIS
036.42 MENINGOCOCCAL ENDOCARDITIS
036.43 MENINGOCOCCAL MYOCARDITIS
036.81 MENINGOCOCC OPTIC NEURIT (ICD9)
036.82 MENINGOCOCC ARTHROPATHY (ICD9)
036.89 MENINGOCOCCAL INFECT NEC (ICD9)
036.9 MENINGOCOCCAL INFECT NOS (ICD9)
038.0 STREPTOCOCCAL SEPTICEMIA (ICD9)
038.1 STAPHYLOCOCC SEPTICEMIA (End 1997) (ICD9)
038.10 STAPH SEPTICEMIA- UNSPEC (Begin 1997) (ICD9)
038.11 STAPH AUREUS SEPTICEMIA (Begin 1997) (ICD9)
038.12 (no description found)
038.19 OT STAPH SEPTICEMIA (Begin 1997) (ICD9)
038.2 PNEUMOCOCCAL SEPTICEMIA (ICD9)
038.3 ANAEROBIC SEPTICEMIA (ICD9)
038.40 GRAM-NEG SEPTICEMIA NOS (ICD9)
038.41 H. INFLUENAE SEPTICEMIA (ICD9)
038.42 E COLI SEPTICEMIA (ICD9)
038.43 PSEUDOMONAS SEPTICEMIA (ICD9)
038.44 SERRATIA SEPTICEMIA (ICD9)
038.49 GRAM-NEG SEPTICEMIA NEC (ICD9)
038.8 SEPTICEMIA NEC (ICD9)
038.9 SEPTICEMIA NOS (ICD9)
040.3 NECROBACILLOSIS (ICD9)
040.82 TOXIC SHOCK SYNDROME (Begin 2002) (ICD9)
041.02 GROUP B STREPTOCOCCUS (Begin 1992) (ICD9)
077.98 DIS OF CONJUNCT DUE TO CHLAMYDIAE (Begin 1993) (ICD9)
078.88 OTH SPEC DISEASE DUE TO CHLAMYDIAE (Begin 1993) (ICD9)
079.88 OTH SPEC CHLAMYDIAL INFECTION (Begin 1993) (ICD9)
079.98 CHLAMYDIAL INFECTION NOS (Begin 1993) (ICD9)
083.0 Q FEVER (ICD9)

083.1 TRENCH FEVER (ICD9)
083.2 RICKETTSIALPOX (ICD9)
083.8 RICKETTSIOSES NEC (ICD9)
083.9 RICKETTSIOSIS NOS (ICD9)
091.3 SECONDARY SYPH SKIN
091.4 SYPHILITIC ADENOPATHY
091.5 SYPHILITIC UVEITIS NOS
091.51 SYPHILITIC CHORIORETINITIS
091.52 SYPHILITIC IRIDOCYCLITIS
091.61 SYPHILITIC PERIOSTITIS
091.62 SYPHILITIC HEPATITIS
091.69 SECOND SYPH VISCERA NEC
091.7 SECOND SYPHILIS RELAPSE
091.81 ACUTE SYPHIL MENINGITIS
091.82 SYPHILITIC ALOPECIA
091.89 SECONDARY SYPHILIS NEC
091.9 SECONDARY SYPHILIS NOS
092.0 EARLY SYPH LATENT RELAPSE
092.9 EARLY SYPH LATENT NOS
093.0 AORTIC ANEURYSM-SYPHIL
093.1 SYPHILITIC AORTITIS
093.2 SYPHIL ENDOCARDITIS NOS
093.21 SYPHILITIC MITRAL VALVE
093.22 SYPHILITIC AORTIC VALVE
093.23 SYPHILITIC TRICUSPID VALVE
093.24 SYPHILITIC PULMONARY VALVE
093.81 SYPHILITIC PERICARDITIS
093.82 SYPHILITIC MYOCARDITIS
093.89 CARDIOVASCULAR SYPH NEC
093.9 CARDIOVASCULAR SYPH NOS
094.0 TABES DORSALIS
094.1 GENERAL PARESIS
094.2 SYPHILITIC MENINGITIS
094.3 ASYMPTOMATIC NEUROSYPHILIS
094.81 SYPHILITIC ENCEPHALITIS
094.82 SYPHILITIC PARKINSONISM
094.83 SYPH DISSEM RETINITIS
094.84 SYPHILITIC OPTIC ATROPHY
094.85 SYPH RETROBULB NEURITIS
094.86 SYPHIL ACOUSTIC NEURITIS
094.87 SYPH RUPT CEREB ANEURYSM
094.89 NEUROSYPHILIS NEC
094.9 NEUROSYPHILIS NOS
095.0 SYPHILITIC EPISCLERITIS
095.1 SYPHILIS OF LUNG
095.2 SYPHILITIC PERITONITIS
095.3 SYPHILIS OF LIVER
095.4 SYPHILIS OF KIDNEY
095.5 SYPHILIS OF BONE
095.6 SYPHILIS OF MUSCLE
095.7 SYPHILIS OF TENDON/BURSA
095.8 LATE SYMPT SYPHILIS NEC
095.9 LATE SYMPT SYPHILIS NOS
096 LATE SYPHILIS LATENT
097.0 LATE SYPHILIS NOS
097.1 LATENT SYPHILIS NOS
097.9 SYPHILIS NOS
098.40 GONOCOCCAL CONJUNCTIVITIS
098.41 GONOCOCCAL IRIDOCYCLITIS
098.42 GONOCOCCAL ENDOPHTHALMIA

098.43 GONOCOCCAL KERATITIS
098.49 GONOCOCCAL EYE NEC
098.50 GONOCOCCAL ARTHRITIS
098.51 GONOCOCCAL SYNOVITIS
098.52 GONOCOCCAL BURSITIS
098.53 GONOCOCCAL SPONDYLITIS
098.59 GC INFECT JOINT NEC
098.6 GONOCOCCAL INFEC PHARYNX
098.7 GC INFECT ANUS & RECTUM
098.81 GONOCOCCAL KERATOSIS
098.82 GONOCOCCAL MENINGITIS
098.83 GONOCOCCAL PERICARDITIS
098.84 GONOCOCCAL ENDOCARDITIS
098.85 GONOCOCCAL HEART DIS NEC
098.86 GONOCOCCAL PERITONITIS
098.89 GONOCOCCAL INF SITE NEC
320.0 HEMOPHILUS MENINGITIS (ICD9)
320.1 PNEUMOCOCCAL MENINGITIS (ICD9)
320.2 STREPTOCOCCAL MENINGITIS (ICD9)
320.3 STAPHYLOCOCC MENINGITIS (ICD9)
320.7 MENING IN OTH BACT DIS (ICD9)
320.8 BACTERIAL MENINGITIS NEC (Begin 1980 (ICD9)
320.81 ANAEROBIC MENINGITIS (Begin 1992) (ICD9)
320.82 GRAM NEG MENINGITIS NEC (Begin 1992) (ICD9)
320.89 OTH BACTER MENINGITIS (Begin 1992) (ICD9)
320.9 BACTERIAL MENINGITIS NOS (ICD9)
322.2 CHRONIC MENINGITIS (ICD9)
323.1 RICKETTSIAL ENCEPHALITIS (ICD9)
324.0 INTRACRANIAL ABSCESS (ICD9)
324.1 INTRASPINAL ABSCESS (ICD9)
324.9 CNS ABSCESS NOS (ICD9)
421.0 ACUTE AND SUBACUTE BACTERIAL ENDOCARDITIS
449 SEPTIC ARTERIAL EMBOLISM (Begin 2007) (ICD9)
519.2 MEDIASTINITIS (ICD9)
659.30 SEPTICEMIA IN LABOR-UNSP (ICD9)
659.31 SEPTICEM IN LABOR-DELIV (ICD9)
659.33 SEPTICEM IN LABOR-ANTEPA (ICD9)
711.00 PYOGEN ARTHRITIS-UNSPEC (ICD9)
711.01 PYOGEN ARTHRITIS-SHLDER (ICD9)
711.02 PYOGEN ARTHRITIS-UP/ARM (ICD9)
711.03 PYOGEN ARTHRITIS-FOREARM (ICD9)
711.04 PYOGEN ARTHRITIS-HAND (ICD9)
711.05 PYOGEN ARTHRITIS-PELVIS (ICD9)
711.06 PYOGEN ARTHRITIS-L/LEG (ICD9)
711.07 PYOGEN ARTHRITIS-ANKLE (ICD9)
711.08 PYOGEN ARTHRITIS NEC (ICD9)
711.09 PYOGEN ARTHRITIS-MULT (ICD9)
711.40 BACT ARTHRITIS-UNSPEC (ICD9)
711.41 BACT ARTHRITIS-SHLDER (ICD9)
711.42 BACT ARTHRITIS-UP/ARM (ICD9)
711.43 BACT ARTHRITIS-FOREARM (ICD9)
711.44 BACT ARTHRITIS-HAND (ICD9)
711.45 BACT ARTHRITIS-PELVIS (ICD9)
711.46 BACT ARTHRITIS-L/LEG (ICD9)
711.47 BACT ARTHRITIS-ANKLE (ICD9)
711.48 BACT ARTHRITIS NEC (ICD9)
711.49 BACT ARTHRITIS-MULT (ICD9)
730.00 AC OSTEOMYELITIS-UNSPEC (ICD9)
730.01 AC OSTEOMYELITIS-SHLDER (ICD9)
730.02 AC OSTEOMYELITIS-UP/ARM (ICD9)

730.03 AC OSTEOMYELITIS-FOREARM (ICD9)
730.04 AC OSTEOMYELITIS-HAND (ICD9)
730.05 AC OSTEOMYELITIS-PELVIS (ICD9)
730.06 AC OSTEOMYELITIS-L/LEG (ICD9)
730.07 AC OSTEOMYELITIS-ANKLE (ICD9)
730.08 AC OSTEOMYELITIS NEC (ICD9)
730.09 AC OSTEOMYELITIS-MULT (ICD9)
730.10 CHR OSTEOMYELITIS-UNSP (ICD9)
730.11 CHR OSTEOMYELIT-SHLDER (ICD9)
730.12 CHR OSTEOMYELIT-UP/ARM (ICD9)
730.13 CHR OSTEOMYELIT-FOREARM (ICD9)
730.14 CHR OSTEOMYELIT-HAND (ICD9)
730.15 CHR OSTEOMYELIT-PELVIS (ICD9)
730.16 CHR OSTEOMYELIT-L/LEG (ICD9)
730.17 CHR OSTEOMYELIT-ANKLE (ICD9)
730.18 CHR OSTEOMYELIT NEC (ICD9)
730.19 CHR OSTEOMYELIT-MULT (ICD9)
730.20 OSTEOMYELITIS NOS-UNSPEC (ICD9)
730.21 OSTEOMYELITIS NOS-SHLDER (ICD9)
730.22 OSTEOMYELITIS NOS-UP/ARM (ICD9)
730.23 OSTEOMYELIT NOS-FOREARM (ICD9)
730.24 OSTEOMYELITIS NOS-HAND (ICD9)
730.25 OSTEOMYELITIS NOS-PELVIS (ICD9)
730.26 OSTEOMYELITIS NOS-L/LEG (ICD9)
730.27 OSTEOMYELITIS NOS-ANKLE (ICD9)
730.28 OSTEOMYELIT NOS-OTH SITE (ICD9)
730.29 OSTEOMYELITIS NOS-MULT (ICD9)
730.80 BONE INFECT NEC-UNSPEC (ICD9)
730.81 BONE INFECT NEC-SHLDER (ICD9)
730.82 BONE INFECT NEC-UP/ARM (ICD9)
730.83 BONE INFECT NEC-FOREARM (ICD9)
730.84 BONE INFECT NEC-HAND (ICD9)
730.85 BONE INFECT NEC-PELVIS (ICD9)
730.86 BONE INFECT NEC-L/LEG (ICD9)
730.87 BONE INFECT NEC-ANKLE (ICD9)
730.88 BONE INFECT NEC-OTH SITE (ICD9)
730.89 BONE INFECT NEC-MULT (ICD9)
730.90 BONE INFEC NOS-UNSP SITE (ICD9)
730.91 BONE INFECT NOS-SHLDER (ICD9)
730.92 BONE INFECT NOS-UP/ARM (ICD9)
730.93 BONE INFECT NOS-FOREARM (ICD9)
730.94 BONE INFECT NOS-HAND (ICD9)
730.95 BONE INFECT NOS-PELVIS (ICD9)
730.96 BONE INFECT NOS-L/LEG (ICD9)
730.97 BONE INFECT NOS-ANKLE (ICD9)
730.98 BONE INFECT NOS-OTH SITE (ICD9)
730.99 BONE INFECT NOS-MULT (ICD9)
771.81 SEPTICEMIA [SEPSIS] OF NEWBORN (Begin 2002) (ICD9)
771.83 BACTEREMIA OF NEWBORN (Begin 2002) (ICD9)
785.52 SEPTIC SHOCK (Begin 2003) (ICD9)
790.7 BACTEREMIA NOS (ICD9)
A02.1 Salmonella sepsis (ICD10)
A17.0+ Tuberculous meningitis (ICD10)
A17.1+ Meningeal tuberculoma (ICD10)
A17.8+ Other tuberculosis of nervous system (ICD10)
A17.9+ Tuberculosis of nervous system, unspecified (ICD10)
A18.0+ Tuberculosis of bones and joints (ICD10)
A19.0 Acute miliary tuberculosis of a single specified site
A19.1 Acute miliary tuberculosis of multiple sites
A20.3 Plague meningitis

A20.7 Septicemic plague (ICD10)
A21.7 Generalized tularaemia
A22.7 Anthrax sepsis (ICD10)
A23.0 Brucellosis due to Brucella melitensis (ICD10)
A23.1 Brucellosis due to Brucella abortus (ICD10)
A23.2 Brucellosis due to Brucella suis (ICD10)
A23.3 Brucellosis due to Brucella canis (ICD10)
A23.8 Other brucellosis (ICD10)
A23.9 Brucellosis, unspecified (ICD10)
A24.1 Acute and fulminating melioidosis
A25.0 Spirillosis (ICD10)
A25.1 Streptobacillosis (ICD10)
A25.9 Rat-bite fever, unspecified (ICD10)
A32.1 Listerial meningitis and meningoencephalitis
A32.7 Listerial sepsis
A39.0+ Meningococcal meningitis (ICD10)
A39.1+ Waterhouse-Friderichsen syndrome (ICD10)
A39.2 Acute meningococcaemia
A39.3 Chronic meningococcaemia
A39.4 Meningococcemia, unspecified (ICD10)
A39.5 Meningococcal heart disease
A39.8 Other meningococcal infections (ICD10)
A39.8+Other meningococcal infections
A39.9 Meningococcal infection, unspecified (ICD10)
A40.0 Sepsis due to streptococcus, group A
A40.1 Sepsis due to streptococcus, group B
A40.2 Sepsis due to streptococcus, group D
A40.3 Sepsis due to Streptococcus pneumoniae (ICD10)
A40.8 Other streptococcal sepsis
A40.9 Streptococcal sepsis, unspecified (ICD10)
A41.0 Sepsis due to Staphylococcus aureus
A41.1 Sepsis due to other specified staphylococcus
A41.2 Sepsis due to unspecified staphylococcus (ICD10)
A41.3 Sepsis due to Hemophilus influenzae (ICD10)
A41.4 Sepsis due to anaerobes (ICD10)
A41.51 Sepsis due to Escherichia coli [E. Coli] (ICD10)
A41.52 Sepsis due to Pseudomonas (ICD10)
A41.58 Sepsis due to other Gram-negative organisms (ICD10)
A41.8 Other specified septicaemia (ICD10)
A41.9 Sepsis, unspecified (ICD10)
A44.0 Systemic bartonellosis
A48.3 Toxic shock syndrome
A52.0+ Cardiovascular syphilis (I98.0*) (ICD10)
A52.1 Symptomatic neurosyphilis (ICD10)
A52.1+ (no description found)
A52.2 Asymptomatic neurosyphilis (ICD10)
A52.3 Neurosyphilis, unspecified
A52.7 Other symptomatic late syphilis (ICD10)
A52.7+ (no description found)
A52.8 Late syphilis, latent (ICD10)
A65 Nonvenereal syphilis (ICD10)
A74.0 Chlamydial conjunctivitis (ICD10)
A74.8 Other chlamydial diseases (ICD10)
A78 Q fever (ICD10)
A79.0 Trench fever (ICD10)
A79.1 Rickettsialpox due to Rickettsia akari (ICD10)
A79.8 Other specified rickettsioses (ICD10)
A79.9 Rickettsiosis, unspecified (ICD10)
B95.1 Streptococcus, group B, as the cause of diseases classified elsewhere (ICD10)
G00.0 Haemophilus meningitis (ICD10)

G00.1 Pneumococcal meningitis (ICD10)
G00.1* (no description found)
G00.2 Streptococcal meningitis (ICD10)
G00.3 Staphylococcal meningitis (ICD10)
G00.8 Other bacterial meningitis (ICD10)
G00.9 Bacterial meningitis, unspecified (ICD10)
G01 Meningitis in bacterial diseases classified elsewhere
G03.1 Chronic meningitis (ICD10)
G05.0 Encephalitis, myelitis and encephalomyelitis in bacterial diseases classified elsewhere
G06.0 Intracranial abscess and granuloma (ICD10)
G06.1 Intraspinal abscess and granuloma (ICD10)
G06.2 Extradural and subdural abscess, unspecified (ICD10)
G07 Intracranial and intraspinal abscess and granuloma in disease classified elsewhere (ICD10)
I30.1 Infective pericarditis
I32.0 Pericarditis in bacterial diseases classified elsewhere
I33.0 Acute and subacute infective endocarditis
I41.0 Myocarditis in bacterial diseases classified elsewhere
J39.0 Retropharyngeal and parapharyngeal abscess (ICD10)
J39.1 Other abscess of pharynx (ICD10)
J85.3 Abscess of mediastinum
M00.90 Pyogenic arthritis, unspecified, multiple sites (ICD10)
M00.91 Pyogenic arthritis, unspecified, shoulder region (ICD10)
M00.92 Pyogenic arthritis, unspecified, upper arm (ICD10)
M00.93 Pyogenic arthritis, unspecified, forearm (ICD10)
M00.94 Pyogenic arthritis, unspecified, hand (ICD10)
M00.95 Pyogenic arthritis, unspecified, pelvic region and thigh
M00.96 Pyogenic arthritis, unspecified, lower leg (ICD10)
M00.97 Pyogenic arthritis, unspecified, ankle and foot (ICD10)
M00.98 Pyogenic arthritis, unspecified, other site (ICD10)
M00.99 Pyogenic arthritis, unspecified, site unspecified (ICD10)
M01.0 Meningococcal arthritis
M01.1 Tuberculous arthritis
M46.2 Osteomyelitis of vertebra
M46.3 Infection of intervertebral disc (pyogenic)
M46.4 Discitis, unspecified
M46.5 Other infective spondylopathies
M49.0 Tuberculosis of spine
M49.1 Brucella spondylitis
M49.2 Enterobacterial spondylitis
M49.3 Spondylopathy in other infectious and parasitic diseases classified elsewhere
M72.6 Necrotizing fasciitis
M73.0 Gonococcal bursitis
M73.1 Syphilitic bursitis
M86.0 Acute haematogenous osteomyelitis
M86.10 Other acute osteomyelitis, unspecified site (ICD10)
M86.11 Other acute osteomyelitis, shoulder region (ICD10)
M86.12 Other acute osteomyelitis, upper arm (ICD10)
M86.13 Other acute osteomyelitis, forearm (ICD10)
M86.14 Other acute osteomyelitis, hand (ICD10)
M86.15 Other acute osteomyelitis, pelvic region and thigh (ICD10)
M86.16 Other acute osteomyelitis, lower leg (ICD10)
M86.17 Other acute osteomyelitis, ankle and foot (ICD10)
M86.18 Other acute osteomyelitis, other site (ICD10)
M86.19 Other acute osteomyelitis, multiple sites (ICD10)
M86.2 Subacute osteomyelitis
M86.4 Chronic osteomyelitis with draining sinus
M86.5 Other chronic osteomyelitis
M86.60 Other chronic osteomyelitis, unspecified site (ICD10)
M86.67 Other chronic osteomyelitis, ankle and foot (ICD10)
M86.68 Other chronic osteomyelitis, other site (ICD10)

M86.69 Other chronic osteomyelitis, multiple sites (ICD10)
M86.8 Other osteomyelitis
M86.90 Unspecified osteomyelitis, multiple sites (ICD10)
M86.91 Unspecified osteomyelitis, shoulder region (ICD10)
M86.92 Unspecified osteomyelitis, upper arm (ICD10)
M86.93 Unspecified osteomyelitis, forearm (ICD10)
M86.94 Unspecified osteomyelitis, hand (ICD10)
M86.95 Unspecified osteomyelitis, pelvic region and thigh (ICD10)
M86.96 Unspecified osteomyelitis, lower leg (ICD10)
M86.97 Unspecified osteomyelitis, ankle and foot (ICD10)
M86.98 Unspecified osteomyelitis, other site (ICD10)
M86.99 Unspecified osteomyelitis, site unspecified (ICD10)
M90.0 Tuberculosis of bone
P36.0 Sepsis of newborn due to streptococcus, group B
P36.1 Sepsis of newborn due to other and unspecified streptococci
P36.2 Sepsis of newborn due to Staphylococcus aureus
P36.3 Sepsis of newborn due to other and unspecified staphylococci
P36.4 Sepsis of newborn due to Escherichia coli
P36.5 Sepsis of newborn due to anaerobes
P36.8 Other bacterial sepsis of newborn
P36.9 Bacterial sepsis of newborn, unspecified
P37.0 Congenital tuberculosis
P37.2 Neonatal (disseminated) listeriosis

Supplementary Table: ICD codes for gastrointestinal infection diagnostic group

001.0 CHOLERA D/T VIB CHOLERAE (ICD9)
001.1 CHOLERA D/T VIB EL TOR (ICD9)
001.9 CHOLERA NOS (ICD9)
002.0 TYPHOID FEVER (ICD9)
002.1 PARATYPHOID FEVER A (ICD9)
002.2 PARATYPHOID FEVER B (ICD9)
002.3 PARATYPHOID FEVER C (ICD9)
002.9 PARATYPHOID FEVER NOS (ICD9)
003.0 SALMONELLA ENTERITIS (ICD9)
003.20 LOCAL SALMONELLA INF NOS (ICD9)
003.29 LOCAL SALMONELLA INF NEC (ICD9)
003.8 SALMONELLA INFECTION NEC (ICD9)
003.9 SALMONELLA INFECTION NOS (ICD9)
004.0 SHIGELLA DYSENTERIAE (ICD9)
004.1 SHIGELLA FLEXNERI (ICD9)
004.2 SHIGELLA BOYDII (ICD9)
004.3 SHIGELLA SONNEI (ICD9)
004.8 SHIGELLA INFECTION NEC (ICD9)
004.9 SHIGELLOSIS NOS (ICD9)
005.0 STAPH FOOD POISONING (ICD9)
005.1 BOTULISM (ICD9)
005.2 FOOD POIS D/T C. PERFRIN (ICD9)
005.3 FOOD POIS: CLOSTRID NEC (ICD9)
005.4 FOOD POIS: V. PARAHAEM (ICD9)
005.8 BACT FOOD POISONING NEC (End 1995) (ICD9)
005.81 FOOD POISONING VIBRIO (Begin 1995) (ICD9)
005.89 OTH BACT FOOD POISONING (Begin 1995) (ICD9)
005.9 FOOD POISONING NOS (ICD9)
006.0 AC AMEBIASIS W/O ABSCESS (ICD9)
006.1 CHR AMEBIASIS W/O ABSCES (ICD9)
006.2 AMEBIC NONDYSENT COLITIS (ICD9)
006.3 AMEBIC LIVER ABSCESS (ICD9)
006.8 AMEBIC INFECTION NEC
006.9 AMEBIASIS NOS (ICD9)
007.0 BALANTIDIASIS (ICD9)
007.1 GIARDIASIS (ICD9)
007.2 COCCIDIOSIS (ICD9)
007.3 INTEST TRICHOMONIASIS (ICD9)
007.4 OT PROTOZ INTEST DX- CRYP (Begin 1997) (ICD9)
007.5 CYCLOSPORIASIS (Begin 2000) (ICD9)
007.8 PROTOZOAL INTEST DIS NEC (ICD9)
007.9 PROTOZOAL INTEST DIS NOS (ICD9)
008.0 E. COLI ENTERITIS (Begin 1980 (ICD9)
008.00 E. COLI ENTERITIS-NOS (Begin 1992) (ICD9)
008.01 E. COLI ENTERITIS-PATH (Begin 1992) (ICD9)
008.02 E. COLI ENTERITIS-TOX (Begin 1992) (ICD9)
008.03 E. COLI ENTERITIS-INVAS (Begin 1992) (ICD9)
008.04 E. COLI ENTERITIS-HEMOR (Begin 1992) (ICD9)
008.09 E. COLI ENTERITIS-OTHER (Begin 1992) (ICD9)
008.1 ARIZONA ENTERITIS (ICD9)
008.2 AEROBACTER ENTERITIS (ICD9)
008.3 PROTEUS ENTERITIS (ICD9)
008.41 STAPHYLOCOCC ENTERITIS (ICD9)
008.42 PSEUDOMONAS ENTERITIS (ICD9)
008.43 CAMPYLOBACTER (Begin 1992) (ICD9)
008.44 YERSINIA (Begin 1992) (ICD9)
008.45 CLOSTRIDIUM DIF (Begin 1992) (ICD9)
008.46 OTHER ANAEROBES (Begin 1992) (ICD9)

008.47	OTH GRAM NEG BACT (Begin 1992) (ICD9)
008.49	BACTERIAL ENTERITIS NEC (ICD9)
008.5	BACTERIAL ENTERITIS NOS (ICD9)
008.6	VIRAL ENTERITIS NEC (Begin 1980 (ICD9)
008.61	ROTAVIRUS ENTERITIS (Begin 1992) (ICD9)
008.62	ADENOVIR ENTERITIS (Begin 1992) (ICD9)
008.63	NORWALK VIR ENTERITIS (Begin 1992) (ICD9)
008.64	SML ROUND VIR ENTERITIS (Begin 1992) (ICD9)
008.65	CALCIVIRUS ENTERITIS (Begin 1992) (ICD9)
008.66	ASTROVIRUS ENTERITIS (Begin 1992) (ICD9)
008.67	ENTERITIS NEC (Begin 1992) (ICD9)
008.69	ENTERITIS NOS (Begin 1992) (ICD9)
008.8	VIRAL ENTERITIS NOS (ICD9)
009.0	INFECTIOUS ENTERITIS NOS (ICD9)
009.1	ENTERITIS OF INFECT ORIG (ICD9)
009.2	INFECTIOUS DIARRHEA NOS (ICD9)
009.3	DIARRHEA OF INFECT ORIG (ICD9)
021.1	ENTERIC TULAREMIA (ICD9)
022.2	GASTROINTESTINAL ANTHRAX (ICD9)
032.83	DIPHTHERITIC PERITONITIS (ICD9)
039.2	ABDOMINAL ACTINOMYCOSIS (ICD9)
040.2	WHIPPLE-S DISEASE
041.86	HELICOBACTER PYLORI INFECTION (Begin 1995) (ICD9)
078.82	EPIDEMIC VOMITING SYND (ICD9)
112.85	CANDIDAL ENTERITIS (Begin 1992) (ICD9)
112.89	CANDIDIASIS SITE NEC (ICD9)
123.6	HYMENOLEPIASIS (ICD9)
123.9	CESTODE INFECTION NOS (ICD9)
126.0	ANCYLOSTOMA DUODENALE (ICD9)
126.9	ANCYLOSTOMIASIS NOS (ICD9)
127.1	ANISAKIASIS (ICD9)
127.2	STRONGYLOIDIASIS (ICD9)
558.9	NONINF GASTROENTERIT NEC (ICD9)
A00.0	Cholera due to Vibrio cholerae 01, biovar cholerae (ICD10)
A00.1	Cholera due to Vibrio cholerae 01, biovar eltor (ICD10)
A00.9	Cholera, unspecified (ICD10)
A01.0	Typhoid fever (ICD10)
A01.1	Paratyphoid fever A (ICD10)
A01.2	Paratyphoid fever B (ICD10)
A01.3	Paratyphoid fever C (ICD10)
A01.4	Paratyphoid fever, unspecified (ICD10)
A02.0	Salmonella enteritis (ICD10)
A02.2+	Localised salmonella infections (ICD10)
A02.8	Other specified salmonella infections (ICD10)
A02.9	Salmonella infection, unspecified (ICD10)
A03.0	Shigellosis due to Shigella dysenteriae (ICD10)
A03.1	Shigellosis due to Shigella flexneri (ICD10)
A03.2	Shigellosis due to Shigella boydii (ICD10)
A03.3	Shigellosis due to Shigella sonnei (ICD10)
A03.8	Other shigellosis (ICD10)
A03.9	Shigellosis, unspecified (ICD10)
A04.0	Enteropathogenic Escherichia coli infection (ICD10)
A04.1	Enterotoxigenic Escherichia coli infection (ICD10)
A04.2	Enteroinvasive Escherichia coli infection (ICD10)
A04.3	Enterohemorrhagic Escherichia coli infection (ICD10)
A04.4	Other intestinal Escherichia coli infections (ICD10)
A04.5	Campylobacter enteritis (ICD10)
A04.6	Enteritis due to Yersinia enterocolitica (ICD10)
A04.7	Enterocolitis due to Clostridium difficile (ICD10)
A04.8	Other specified bacterial intestinal infections (ICD10)

A04.9 Bacterial intestinal infection, unspecified (ICD10)
A05.0 Foodborne staphylococcal intoxication (ICD10)
A05.1 Botulism food poisoning (ICD10)
A05.2 Foodborne Clostridium perfringens [Clostridium welchii] intoxication (ICD10)
A05.3 Foodborne Vibrio parahaemolyticus intoxication (ICD10)
A05.4 Foodborne Bacillus cereus intoxication (ICD10)
A05.8 Other specified bacterial foodborne intoxications (ICD10)
A05.9 Bacterial foodborne intoxication, unspecified (ICD10)
A06.0 Acute amebic dysentery (ICD10)
A06.1 Chronic intestinal amebiasis (ICD10)
A06.2 Amebic nondysenteric colitis (ICD10)
A06.3 Amoeboma of intestine (ICD10)
A06.4 Amebic liver abscess (ICD10)
A06.9 Amebiasis, unspecified (ICD10)
A07.0 Balantidiasis (ICD10)
A07.1 Giardiasis [lambliasis] (ICD10)
A07.2 Cryptosporidiosis (ICD10)
A07.3 Isosporiasis (ICD10)
A07.8 Other specified protozoal intestinal diseases (ICD10)
A07.9 Protozoal intestinal disease, unspecified (ICD10)
A08.0 Rotaviral enteritis (ICD10)
A08.1 Acute gastroenteropathy due to Norwalk agent (ICD10)
A08.2 Adenoviral enteritis (ICD10)
A08.3 Other viral enteritis (ICD10)
A08.4 Viral intestinal infection, unspecified
A08.5 Other specified intestinal infections (ICD10)
A09 Infectious gastroenteritis and colitis, unspecified (ICD10)
A09.0 Other and unspecified gastroenteritis and colitis of infectious origin
A09.9 Gastroenteritis and colitis of unspecified origin
A21.3 Gastrointestinal tularemia (ICD10)
A22.2 Gastrointestinal anthrax (ICD10)
B37.88 Candidiasis of other sites (ICD10)
B71.0 Hymenolepiasis (ICD10)
B71.9 Cestode infection, unspecified (ICD10)
B76.0 Ancylostomiasis (ICD10)
B78.9 Strongyloidiasis, unspecified (ICD10)
B81.0 Anisakiasis (ICD10)
B96.81 Helicobacter pylori [H. pylori] as the cause of diseases classified to other chapters (ICD10)
K52.8 Other specified noninfective gastroenteritis and colitis (ICD10)

Supplementary Table: ICD codes for lower respiratory infection diagnostic group

003.22	SALMONELLA PNEUMONIA (ICD9)
006.4	AMEBIC LUNG ABSCESS (ICD9)
010.00	PRIM TB COMPLEX-UNSPEC (ICD9)
010.01	PRIM TB COMPLEX-NO EXAM (ICD9)
010.02	PRIM TB COMPLEX-EXM UNKN (ICD9)
010.03	PRIM TB COMPLEX-MICRO DX (ICD9)
010.04	PRIM TB COMPLEX-CULT DX (ICD9)
010.05	PRIM TB COMPLEX-HISTO DX (ICD9)
010.06	PRIM TB COMPLEX-OTH TEST (ICD9)
010.10	PRIM TB PLEURISY-UNSPEC (ICD9)
010.11	PRIM TB PLEURISY-NO EXAM (ICD9)
010.12	PRIM TB PLEUR-EXAM UNKN (ICD9)
010.13	PRIM TB PLEURIS-MICRO DX (ICD9)
010.14	PRIM TB PLEURISY-CULT DX (ICD9)
010.15	PRIM TB PLEURIS-HISTO DX (ICD9)
010.16	PRIM TB PLEURIS-OTH TEST (ICD9)
010.80	PRIM PROG TB NEC-UNSPEC
010.81	PRIM PROG TB NEC-NO EXAM
010.82	PRIM PR TIB NEC-EXAM UNKN
010.83	PRIM PRG TB NEC-MICRO DX
010.84	PRIM PROG TB NEC-CULT DX
010.85	PRIM PRG TB NEC-HITO DX
010.86	PRIM PRG TB NEC-OTH TEST
010.90	PRIMARY TB NOS-UNSPEC
010.91	PRIMARY TB NOS-NO EXAM
010.92	PRIMARY TB NOS-EXAM UNKN
010.93	PRIMARY TB NOS-MICRO DX
010.94	PRIMARY TB NOS-CULT DX
010.95	PRIMARY TB NOS-HISTO DX
010.96	PRIMARY TB NOS-OTH TEST
011.00	TB LUNG INFILTR-UNSPEC
011.01	TB LUNG INFILTR-NO EXAM
011.02	TB LUNG INFILTR-EXM UNKN
011.03	TB LUNG INFILTR-MICRO DX
011.04	TB LUNG INFILTR-CULT DX
011.05	TB LUNG INFILTR-HISTO DX
011.06	TB LUNG INFILTR-OTH TEST
011.10	TB LUNG NODULAR-UNSPEC
011.11	TB LUNG NODULAR-NO EXAM
011.12	TB LUNG NODULAR-EXAM UNKN
011.13	TB LUNG NODULAR-MICRO DX
011.14	TB LUNG NODULAR-CULT DX
011.15	TB LUNG NODULAR-HISTO DX
011.16	TB LUNG NODULAR-OTH TEST
011.20	TB LUNG W CAVITY-UNSPEC
011.21	TB LUNG W CAVITY-NO EXAM
011.22	TB LUNG W CAVITY-EXAM UNKN
011.23	TB LUNG W CAVITY-MICRO DX
011.24	TB LUNG W CAVITY-CULT DX
011.25	TB LUNG W CAVITY-HISTO DX
011.26	TB LUNG W CAVITY-OTH TEST
011.30	TB OF BRONCHUS-UNSPEC
011.31	TB OF BRONCHUS- NO EXAM
011.32	TB OF BRONCHUS-EXAM UNKN
011.33	TB OF BRONCHUS- MICRO DX
011.34	TB OF BRONCHUS- CULT DX
011.35	TB OF BRONCHUS- HISTO DX

011.36 TB OF BRONCHUS- OTH TEST
011.40 TB LUNG FIBROSIS- UNSPEC
011.41 TB LUNG FIBROSIS- NO EXAM
011.42 TB LUNG FIBROSIS- EXAM UNKN
011.43 TB LUNG FIBROSIS- MICRO DX
011.44 TB LUNG FIBROSIS- CULT DX
011.45 TB LUNG FIBROSIS- HISTO DX
011.46 TB LUNG FIBROSIS- OTH TEST
011.50 TB BRONCHIECTASIS- UNSPEC
011.51 TB BRONCHIECTASIS- NO EXAM
011.52 TB BRONCHIECTASIS- EXAM UNKN
011.53 TB BRONCHIECTASIS- MICRO DX
011.54 TB BRONCHIECTASIS- CULT DX
011.55 TB BRONCHIECTASIS- HISTO DX
011.56 TB BRONCHIECTASIS- OTH TEST
011.60 TB PNEUMONIA- UNSPEC
011.61 TB PNEUMONIA- NO EXAM
011.62 TB PNEUMONIA- EXAM UNKN
011.63 TB PNEUMONIA- MICRO DX
011.64 TB PNEUMONIA- CULT DX
011.65 TB PNEUMONIA- HISTO DX
011.66 TB PNEUMONIA- OTH TEST
011.70 TB PNEUMOTHORAX- UNSPEC
011.71 TB PNEUMOTHORAX- NO EXAM
011.72 TB PNEUMOTHORAX- EXAM UNKN
011.73 TB PNEUMOTHORAX- MICRO DX
011.74 TB PNEUMOTHORAX- CULT DX
011.75 TB PNEUMOTHORAX- HISTO DX
011.76 TB PNEUMOTHORAX- OTH TEST
011.80 PULMONARY TB NEC- UNSPEC
011.81 PULMONARY TB NEC- NO EXAM
011.82 PULMONARY TB NEC- EXAM UNKN
011.83 PULMONARY TB NEC- MICRO DX
011.84 PULMONARY TB NEC- CULT DX
011.85 PULMONARY TB NEC- HISTO DX
011.86 PULMONARY TB NEC- OTH TEST
011.90 PULMONARY TB NOS- UNSPEC
011.91 PULMONARY TB NOS- NO EXAM
011.92 PULMONARY TB NOS- EXAM UNKN
011.93 PULMONARY TB NOS- MICRO DX
011.94 PULMONARY TB NOS- CULT DX
011.95 PULMONARY TB NOS- HISTO DX
011.96 PULMONARY TB NOS- OTH TEST
012.00 TB PLEURISY- UNSPEC
012.01 TB PLEURISY- NO EXAM
012.02 TB PLEURISY- EXAM UNKN
012.03 TB PLEURISY- MICRO DX
012.04 TB PLEURISY- CULT DX
012.05 TB PLEURISY- HISTO DX
012.06 TB PLEURISY- OTH TEST
012.10 TB THORACIC NODES- UNSPEC
012.11 TB THORACIC NODES- NO EXAM
012.12 TB THORACIC NODES- EXAM UNKN
012.13 TB THORACIC NODES- MICRO DX
012.14 TB THORACIC NODES- CULT DX
012.15 TB THORACIC NODES- HISTO DX
012.16 TB THORACIC NODES- OTH TEST
018.00 ACUTE MILIARY TB- UNSPEC
018.01 ACUTE MILIARY TB- NO EXAM
018.02 ACUTE MILIARY TB- EXAM UNKN

018.03 ACUTE MILIARY TB- MICRO DX
018.04 ACUTE MILIARY TB- CULT DX
018.05 ACUTE MILIARY TB- HISTO DX
018.06 ACUTE MILIARY TB- OTH TEST
018.80 MILIARY TB NEC- UNSPEC
018.81 MILIARY TB NEC- NO EXAM
018.82 MILIARY TB NEC- EXAM UNKN
018.83 MILIARY TB NEC- MICRO DX
018.84 MILIARY TB NEC- CULT DX
018.85 MILIARY TB NEC- HISTO DX
018.86 MILIARY TB NEC- OTH TEST
018.90 MILIARY TB NOS- UNSPEC
018.91 MILIARY TB NOS-NO EXAM
018.92 MILIARY TB NOS- EXAM UNKN
018.93 MILIARY TB NOS-MICRO DX
018.94 MILIARY TB NOS- CULT DX
018.95 MILIARY TB NOS-HISTO DX
018.96 MILIARY TB NOS- OTH TEST
020.3 PRIMARY PNEUMONIC PLAGUE (ICD9)
020.4 SECONDARY PNEUMON PLAGUE (ICD9)
020.5 PNEUMONIC PLAGUE NOS (ICD9)
021.2 PULMONARY TULAREMIA (ICD9)
022.1 PULMONARY ANTHRAX (ICD9)
024 GLANDERS
031.0 PULMONARY MYCOBACTERIA (ICD9)
033.0 BORDETELLA PERTUSSIS (ICD9)
033.1 BORDETELLA PARAPERTUSSIS (ICD9)
033.8 WHOOPING COUGH NEC (ICD9)
033.9 WHOOPING COUGH NOS (ICD9)
039.1 PULMONARY ACTINOMYCOSIS (ICD9)
052.1 VARICELLA PNEUMONITIS (ICD9)
055.1 POSTMEASLES PNEUMONIA (ICD9)
073.0 ORNITHOSIS PNEUMONIA (ICD9)
073.7 ORNITHOSIS COMPLICAT NEC (ICD9)
073.8 ORNITHOSIS COMPLICAT NOS (ICD9)
073.9 ORNITHOSIS NOS (ICD9)
079.0 ADENOVIRUS INFECT NOS (ICD9)
079.6 RESPIR SYNCITIAL VIRUS (Begin 1996) (ICD9)
079.82 SARS-ASSOCIATED CORONAVIRUS (Begin 2003) (ICD9)
112.4 CANDIDIASIS OF LUNG (ICD9)
114.0 PRIMARY COCCIDIOIDOMYCOS (ICD9)
114.4 CHRONIC PULMON COCCIDIOIDOMYCOSIS (Begin 1993) (ICD9)
114.5 UNSPEC PULMON COCCIDIOIDOMYCOSIS (Begin 1993) (ICD9)
115.05 HISTOPLASM CAPS PNEUMON (ICD9)
115.10 HISTOPLASMA DUBOISII NOS (ICD9)
115.15 HISTOPLASM DUB PNEUMONIA (ICD9)
115.90 HISTOPLASMOSIS NOS (ICD9)
115.95 HISTOPLASMOSIS PNEUMONIA (ICD9)
130.4 TOXOPLASMA PNEUMONITIS (ICD9)
136.3 PNEUMOCYSTOSIS (ICD9)
464.4 CROUP (ICD9)
466.0 ACUTE BRONCHITIS (ICD9)
466.1 ACUTE BRONCHIOLITIS (End 1996) (ICD9)
466.11 RSV BRONCHIOLITIS (Begin 1996) (ICD9)
466.19 OTH ACUTE BRONCHIOL (Begin 1996) (ICD9)
480.0 ADENOVIRAL PNEUMONIA (ICD9)
480.1 RESP SYNCYT VIRAL PNEUM (ICD9)
480.2 PARINFLUENZA VIRAL PNEUM (ICD9)
480.3 PNEUMONIA DUE TO SARS-ASSOCIATED CORONAVIRUS (Begin 2003) (ICD9)
480.8 VIRAL PNEUMONIA NEC (ICD9)

480.9 VIRAL PNEUMONIA NOS (ICD9)
481 PNEUMOCOCCAL PNEUMONIA (ICD9)
481.9 (no description found)
482.0 K. PNEUMONIAE PNEUMONIA (ICD9)
482.1 PSEUDOMONAL PNEUMONIA (ICD9)
482.2 H.INFLUENZAE PNEUMONIA (ICD9)
482.3 STREPTOCOCCAL PNEUMONIA (Begin 1980 (ICD9)
482.30 STREP PNEUMONIA UNSPEC (Begin 1992) (ICD9)
482.31 GRP A STREP PNEUMONIA (Begin 1992) (ICD9)
482.32 GRP B STREP PNEUMONIA (Begin 1992) (ICD9)
482.39 OTH STREP PNEUMONIA (Begin 1992) (ICD9)
482.4 STAPHYLOCOCCAL PNEUMONIA (End 1998) (ICD9)
482.40 STAPH PNEUMONIA UNSP (Begin 1998) (ICD9)
482.41 STAPH AUREUS PNEUMON (Begin 1998) (ICD9)
482.42 (no description found)
482.49 STAPH PNEUMON OTH (Begin 1998) (ICD9)
482.8 BACTERIAL PNEUMONIA NEC (Begin 1980 (ICD9)
482.81 ANAEROBIC PNEUMONIA (Begin 1992) (ICD9)
482.82 E COLI PNEUMONIA (Begin 1992) (ICD9)
482.83 OTH GRAM NEG PNEUMONIA (Begin 1992) (ICD9)
482.84 LEGIONNAIRES DX (Begin 1997) (ICD9)
482.89 BACT PNEUMONIA NEC (Begin 1992) (ICD9)
482.9 BACTERIAL PNEUMONIA NOS (ICD9)
483 PNEUMONIA: ORGANISM NEC (Begin 1980 (ICD9)
483.0 MYCOPLASMA PNEUMONIA (Begin 1992) (ICD9)
483.1 CHLAMYDIA PNEUMONIA (Begin 1996) (ICD9)
483.8 OTH SPEC ORG PNEUMONIA (Begin 1992) (ICD9)
484.1 PNEUM W CYTOMEG INCL DIS (ICD9)
484.3 PNEUMONIA IN WHOOP COUGH (ICD9)
484.5 PNEUMONIA IN ANTHRAX (ICD9)
484.6 PNEUM IN ASPERGILLOSIS (ICD9)
484.7 PNEUM IN OTH SYS MYCOSES (ICD9)
484.8 PNEUM IN INFECT DIS NEC (ICD9)
485 BRONCOPNEUMONIA ORG NOS (ICD9)
486 PNEUMONIA- ORGANISM NOS (ICD9)
487.0 INFLUENZA WITH PNEUMONIA (ICD9)
487.1 FLU W RESP MANIFEST NEC (ICD9)
487.8 FLU W MANIFESTATION NEC (ICD9)
488.01 FLU D/T IDENTIFIED AVIAN FLU VIRUS WITH PNEUMONIA
488.02 FLU D/T IDENTIFIED AVIAN FLU VIRUS WITH OTHER RESPIRATORY MANIFESTATIONS
488.11 FLU D/T IDENTIFIED 2009 H1N1 FLU VIRUS WITH PNEUMONIA
488.12 FLU D/T IDENTIFIED 2009 H1N1 FLU VIRUS WITH OTHER RESPIRATORY MANIFESTATIONS
488.81 FLU D/T IDENTIFIED NOVEL INFLUENZA A VIRUS W/ PNEUMONIA
488.82 FLU D/T IDENTIFIED NOVEL INFLUENZA A VIRUS W/ OTHER RESP MANIF
490 BRONCHITIS NOS
491.0 SIMPLE CHR BRONCHITIS (ICD9)
491.1 MUCOPURUL CHR BRONCHITIS (ICD9)
491.2 OBSTRUCT CHR BRONCHITIS (Begin 1980 (ICD9)
491.20 OBS CHR BRNC W/O ACT EXA (Begin 1991) (ICD9)
491.21 OBS CHR BRNC W ACT EXA (Begin 1991) (ICD9)
491.22 OBS CHR BRONC W AC BRONC (Begin 2004) (ICD9)
491.8 CHRONIC BRONCHITIS NEC (ICD9)
491.9 CHRONIC BRONCHITIS NOS (ICD9)
494 BRONCHIECTASIS (End 2000) (ICD9)
494.0 BRONCHIECTASIS W/O ACUTE EXACERBATN (Begin 2000) (ICD9)
494.1 BRONCHIECTASIS W/ACUTE EXACERBATION (Begin 2000) (ICD9)
510.0 EMPYEMA WITH FISTULA
510.9 EMPYEMA W/O FISTULA

513.0 ABSCESS OF LUNG (ICD9)
513.1 ABSCESS OF MEDIASTINUM
517.1 RHEUMATIC PNEUMONIA (ICD9)
770.0 CONGENITAL PNEUMONIA

A06.5+ Amebic lung abscess (ICD10)
A15.0 Tuberculosis of lung
A15.1 Tuberculosis of lung, confirmed by culture only
A15.2 Tuberculosis of lung, confirmed histologically
A15.3 Tuberculosis of lung, confirmed by unspecified means
A15.4 Tuberculosis of intrathoracic lymph nodes
A15.5 Tuberculosis of larynx, trachea and bronchus
A15.6 Tuberculosis pleurisy
A15.7 Primary respiratory tuberculosis
A15.8 Other respiratory tuberculosis
A15.9 Respiratory tuberculosis unspecified, confirmed bacteriologically and histologically
A16.0 Tuberculosis of lung, bacteriologically and histologically negative
A16.1 Tuberculosis of lung, bacteriological and histological examination not done
A16.2 Tuberculosis of lung, without mention of bacteriological or histological confirmation
A16.3 Tuberculosis of intrathoracic lymph nodes, without mention of bacteriological or histological confirmation
A16.4 Tuberculosis of larynx, trachea and bronchus, without mention of bacteriological or histological confirmation
A16.5 Tuberculous pleurisy, without mention of bacteriological or histological confirmation
A16.7 Primary respiratory tuberculosis, without mention of bacteriological or histological confirmation
A16.8 Other respiratory tuberculosis, without mention of bacteriological or histological confirmation
A16.9 Respiratory tuberculosis unspecified, without mention of bacteriological or histological confirmation
A19.2 Acute miliary tuberculosis, unspecified (ICD10)
A19.8 Other miliary tuberculosis (ICD10)
A19.9 Miliary tuberculosis, unspecified (ICD10)
A20.2 Pneumonic plague (ICD10)
A21.2 Pulmonary tularemia (ICD10)
A22.1 Pulmonary anthrax (ICD10)
A24.0 Glanders
A31.0 Pulmonary mycobacterial infection (ICD10)
A37.0 Whooping cough due to Bordetella pertussis (ICD10)
A37.1 Whooping cough due to Bordetella parapertussis (ICD10)
A37.8 Whooping cough due to other Bordetella species (ICD10)
A37.9 Whooping cough, unspecified (ICD10)
A42.0 Pulmonary actinomycosis (ICD10)
A48.1 Legionnaires disease
A70 Chlamydia psittaci infections (ICD10)
A70+ (no description found)
B01.2+ Varicella pneumonia (ICD10)
B05.2+ Measles complicated by pneumonia (ICD10)
B34.0 Adenovirus infection, unspecified (ICD10)
B37.1 Pulmonary candidiasis (ICD10)
B38.0 Acute pulmonary coccidioidomycosis (ICD10)
B38.1 Chronic pulmonary coccidioidomycosis (ICD10)
B38.2 Pulmonary coccidioidomycosis, unspecified (ICD10)
B39.0 Acute pulmonary histoplasmosis capsulati
B39.1 Chronic pulmonary histoplasmosis capsulati
B39.2 Pulmonary histoplasmosis capsulati, unspecified (ICD10)
B39.3 Disseminated histoplasmosis capsulati
B39.4 Histoplasmosis capsulati, unspecified
B39.5 Histoplasmosis duboisii (ICD10)
B39.9 Histoplasmosis, unspecified (ICD10)
B40.0 Acute pulmonary blastomycosis
B40.1 Chronic pulmonary blastomycosis
B40.2 Pulmonary blastomycosis, unspecified
B40.3 Cutaneous blastomycosis

B40.7 Disseminated blastomycosis
B40.8 Other forms of blastomycosis
B41.0 Pulmonary paracoccidioidomycosis
B42.0+ Pulmonary sporotrichosis
B44.0 Invasive pulmonary aspergillosis
B44.1 Other pulmonary aspergillosis
B45.0 Pulmonary cryptococcosis
B58.3+ Pulmonary toxoplasmosis (ICD10)
B59 Pneumocystosis (ICD10)
J05.0 Acute obstructive laryngitis [croup] (ICD10)
J09 Influenza due to certain identified influenza virus
J10.0 Influenza with pneumonia, influenza virus identified (ICD10)
J10.1 Influenza due to other influenza virus with respiratory manifestations (ICD10)
J11.0 Influenza with pneumonia, virus not identified (ICD10)
J11.1 Influenza with other respiratory manifestations, virus not identified (ICD10)
J12.0 Adenoviral pneumonia (ICD10)
J12.1 Respiratory syncytial virus pneumonia (ICD10)
J12.2 Parainfluenza virus pneumonia (ICD10)
J12.3 Human metapneumovirus pneumonia
J12.8 Other viral pneumonia (ICD10)
J12.9 Viral pneumonia, unspecified (ICD10)
J13 Pneumonia due to Streptococcus pneumoniae (ICD10)
J14 Pneumonia due to Hemophilus influenzae (ICD10)
J15.0 Pneumonia due to Klebsiella pneumoniae (ICD10)
J15.1 Pneumonia due to Pseudomonas (ICD10)
J15.2 Pneumonia due to staphylococcus (ICD10)
J15.3 Pneumonia due to streptococcus, group B (ICD10)
J15.4 Pneumonia due to other streptococci (ICD10)
J15.5 Pneumonia due to Escherichia coli (ICD10)
J15.6 Pneumonia due to other aerobic Gram-negative bacteria (ICD10)
J15.7 Pneumonia due to Mycoplasma pneumoniae (ICD10)
J15.8 Pneumonia due to other specified bacteria (ICD10)
J15.9 Unspecified bacterial pneumonia (ICD10)
J16.0 Chlamydial pneumonia
J16.8 Pneumonia due to other specified infectious organisms (ICD10)
J17.0* Pneumonia in bacterial diseases classified elsewhere (ICD10)
J17.1* Pneumonia in viral diseases classified elsewhere (ICD10)
J17.2* Pneumonia in mycoses (ICD10)
J17.3 Pneumonia in parasitic diseases
J17.8* Pneumonia in other diseases classified elsewhere (ICD10)
J18.0 Bronchopneumonia, unspecified organism (ICD10)
J18.1 Lobar pneumonia, unspecified
J18.8 Other pneumonia, unspecified organism (ICD10)
J18.9 Pneumonia, unspecified
J20.0 Acute bronchitis due to Mycoplasma pneumoniae (ICD10)
J20.1 Acute bronchitis due to Hemophilus influenzae (ICD10)
J20.2 Acute bronchitis due to streptococcus (ICD10)
J20.3 Acute bronchitis due to coxsackievirus (ICD10)
J20.4 Acute bronchitis due to parainfluenza virus (ICD10)
J20.5 Acute bronchitis due to respiratory syncytial virus (ICD10)
J20.6 Acute bronchitis due to rhinovirus (ICD10)
J20.7 Acute bronchitis due to echovirus (ICD10)
J20.8 Acute bronchitis due to other specified organisms (ICD10)
J20.9 Acute bronchitis, unspecified (ICD10)
J21.0 Acute bronchiolitis due to respiratory syncytial virus
J21.1 Acute bronchiolitis due to human megapneumovirus
J21.8 Acute bronchiolitis due to other specified organisms
J21.9 Acute bronchiolitis, unspecified (ICD10)
J22 Unspecified acute lower respiratory infection (ICD10)
J22.0 (no description found)

J40 Bronchitis, not specified as acute or chronic
J41.0 Simple chronic bronchitis (ICD10)
J41.1 Mucopurulent chronic bronchitis (ICD10)
J41.8 Mixed simple and mucopurulent chronic bronchitis
J42 Unspecified chronic bronchitis (ICD10)
J44.0 Chronic obstructive pulmonary disease with acute lower respiratory infection
J47 Bronchiectasis (ICD10)
J65 Pneumoconiosis associated with tuberculosis
J85.0 Gangrene and necrosis of lung
J85.1 Abscess of lung with pneumonia
J85.2 Abscess of lung without pneumonia (ICD10)
J86.0 Pyothorax with fistula
J86.9 Pyothorax without fistula
P23.0 Congenital pneumonia due to viral agent
P23.2 Congenital pneumonia due to staphylococcus
P23.3 Congenital pneumonia due to staphylococcus, group B
P23.4 Congenital pneumonia due to Escherichia coli
P23.5 Congenital pneumonia due to Pseudomonas
P23.6 Congenital pneumonia due to other bacterial agents
P23.8 Congenital pneumonia due to other organisms
P23.9 Congenital pneumonia, unspecified

Supplementary Table: ICD codes for skin and soft tissue infection diagnostic group

006.6 AMEBIC SKIN ULCERATION (ICD9)
020.1 CELLULOCUTANEOUS PLAGUE
022.0 CUTANEOUS ANTHRAX
031.1 CUTANEOUS MYCOBACTERIA (ICD9)
032.85 CUTANEOUS DIPTHERIA
035 ERYSIPELAS (ICD9)
039.0 CUTANEOUS ACTINOMYCOSIS
039.4 MADURA FOOT
040.0 GAS GANGRENE
040.42 WOUND BOTULISM
040.81 TROPICAL PYOMYOSITIS
054.0 ECZEMA HERPETICUM (ICD9)
110.0 DERMATOPHYT SCALP/BEARD
110.1 DERMATOPHYTOSIS OF NAIL
110.2 DERMATOPHYTOSIS OF HAND
110.3 DERMATOPHYTOSIS OF GROIN
110.4 DERMATOPHYTOSIS OF FOOT
110.5 DERMATOPHYTOSIS OF BODY
110.6 DEEP DERMATOPHYTOSIS
110.8 DERMATOPHYTOSIS SITE NEC
110.9 DERMATOPHYTOSIS SITE NOS (ICD9)
111.0 PITYRIASIS VERSICOLOR
111.1 TINEA NIGRA
111.2 TINEA BLANCA
111.3 BLACK PIEDRA
111.8 DERMATOMYCOSES NEC
111.9 DERMATOMYCOSES NOS
112.3 CUTANEOUS CANDIDIASIS (ICD9)
132.0 PEDICULUS CAPITIS (ICD9)
132.1 PEDICULUS CORPORIS (ICD9)
132.2 PHTHIRUS PUBIS (ICD9)
132.3 MIXED PEDICUL & PHTHIRUS (ICD9)
132.9 PEDICULOSIS NOS (ICD9)
133.0 SCABIES (ICD9)
133.8 ACARIASIS NEC (ICD9)
133.9 ACARIASIS NOS (ICD9)
134.0 MYIASIS (ICD9)
134.1 ARTHROPOD INFEST NEC (ICD9)
134.2 HIRUDINIASIS (ICD9)
134.8 INFESTATION NEC (ICD9)
134.9 INFESTATION NOS (ICD9)
376.01 ORBITAL CELLULITIS (ICD9)
380.10 INFEC OTITIS EXTERNA NOS (ICD9)
380.11 ACUTE INFECTION OF PINNA (ICD9)
675.00 INFECT NIPPLE PREG-UNSP
675.01 INFECT NIPPLE-DELIVERED
675.02 INFECT NIPPLE-DEL W P/P
675.03 INFECT NIPPLE-ANTEPARTUM
675.04 INFECT NIPPLE-POSTPARTUM
675.10 BREAST ABSCESS PREG-UNSP
675.11 BREAST ABSCESS-DELIVERED
675.12 BREAST ABSCESS-DEL W P/P
675.13 BREAST ABSCESS-ANTEPARTUM
675.14 BREAST ABSCESS-POSTPARTUM
675.20 MASTITIS IN PREG- UNSP
675.21 MASTITIS- DELIVERED
675.22 MASTITIS- DEL W P/P
675.23 MASTITIS- ANTEPARTUM

675.24 MASTITIS- POSTPARTUM
675.80 BREAST INF PREG NEC-UNSP
675.81 BREAST INF NEC- DELIVERED
675.82 BREAST INF NEC- DEL W P/P
675.83 BREAST INF NEC- ANTEPARTUM
675.84 BREAST INF NEC- POSTPARTUM
675.90 BREAST INF PREG NOS-UNSP
675.91 BREAST INF NOS- DELIVERED
675.92 BREAST INF NOS- DEL W P/P
675.93 BREAST INF NOS- ANTEPARTUM
675.94 BREAST INF NOS- POSTPARTUM
680.0 CARBUNCLE OF FACE (ICD9)
680.1 CARBUNCLE OF NECK (ICD9)
680.2 CARBUNCLE OF TRUNK (ICD9)
680.3 CARBUNCLE OF ARM (ICD9)
680.4 CARBUNCLE OF HAND
680.5 CARBUNCLE OF BUTTOCK (ICD9)
680.6 CARBUNCLE OF LEG (ICD9)
680.7 CARBUNCLE OF FURUNCLE OF FOOT
680.8 CARBUNCLE- SITE NEC (ICD9)
680.9 CARBUNCLE NOS (ICD9)
681.00 CELLULITIS- FINGER NOS (ICD9)
681.01 FELON
681.02 ONYCHIA OF FINGER
681.10 CELLULITIS- TOE NOS (ICD9)
681.11 ONYCHIA OF TOE
681.9 CELLULITIS OF DIGIT NOS
682.0 CELLULITIS OF FACE (ICD9)
682.1 CELLULITIS OF NECK (ICD9)
682.2 CELLULITIS OF TRUNK (ICD9)
682.3 CELLULITIS OF ARM (ICD9)
682.4 CELLULITIS OF HAND (ICD9)
682.5 CELLULITIS OF BUTTOCK (ICD9)
682.6 CELLULITIS OF LEG (ICD9)
682.7 CELLULITIS OF FOOT (ICD9)
682.8 CELLULITIS- SITE NEC (ICD9)
682.9 CELLULITIS NOS (ICD9)
684 IMPETIGO (ICD9)
685.0 PILONIDAL CYST W ABSCESS
685.1 PILONIDAL CYST W/O ABSC (ICD9)
686.0 PYODERMA (End 1997) (ICD9)
686.00 PYODERMA NOS
686.01 PYODERMA GANGREN
686.09 PYODERMA NEC
686.1 PYOGENIC GRANULOMA
686.8 LOCAL SKIN INFECTION NEC
686.9 LOCAL SKIN INFECTION NOS (ICD9)
690.8 OTHER ERYTHEMATOSQUAMOUS DERMATOSIS
694.0 DERMATITIS HERPETIFORMIS
694.1 SUBCORNEAL PUST DERMATOS
694.2 JUVEN DERMAT HERPETIFORM
694.3 IMPETIGO HERPETIFORMIS
771.5 NEONATAL INFEC MASTITISA06.7 Cutaneous amebiasis (ICD10)
A31.1 Cutaneous mycobacterial infection (ICD10)
A46 Erysipelas (ICD10)
B00.0 Eczema herpeticum (ICD10)
B35.0 Tinea barbae and tinea capitis
B35.1 Tinea unguium
B35.2 Tinea manuum
B35.3 Tinea pedis

B35.4 Tinea corporis
B35.5 Tinea imbricate
B35.6 Tinea cruris
B35.8 Other dermatophytoses
B35.9 Dermatophytosis, unspecified (ICD10)
B36.0 Pityriasis versicolour
B36.1 Tinea nigra
B36.2 White piedra
B36.3 Black piedra
B36.8 Other specified superficial mycoses
B37.2 Candidiasis of skin and nail (ICD10)
B85.0 Pediculosis due to Pediculus humanus capitis (ICD10)
B85.1 Pediculosis due to Pediculus humanus corporis (ICD10)
B85.2 Pediculosis, unspecified (ICD10)
B85.3 Phthiriasis (ICD10)
B85.4 Mixed pediculosis and phthiriasis (ICD10)
B86 Scabies (ICD10)
B87.9 Myiasis, unspecified (ICD10)
B88.0 Other acariasis (ICD10)
B88.1 Tungiasis [sandflea infestation] (ICD10)
B88.2 Other arthropod infestations (ICD10)
B88.3 External hirudiniasis (ICD10)
B88.8 Other specified infestations (ICD10)
B88.9 Infestation, unspecified (ICD10)
H60.3 Other infective otitis externa (ICD10)
L00 Staphylococcal scalded skin syndrome
L01.0 Impetigo [any organism] [any site] (ICD10)
L02.0 Cutaneous abscess, furuncle and carbuncle of face (ICD10)
L02.1 Cutaneous abscess, furuncle and carbuncle of neck (ICD10)
L02.2 Cutaneous abscess, furuncle and carbuncle of trunk (ICD10)
L02.3 Cutaneous abscess, furuncle and carbuncle of buttock (ICD10)
L02.4 Cutaneous abscess, furuncle and carbuncle of limb (ICD10)
L02.8 Cutaneous abscess, furuncle and carbuncle of other sites (ICD10)
L02.9 Cutaneous abscess, furuncle and carbuncle, unspecified (ICD10)
L03.01 Cellulitis of finger (ICD10)
L03.02 Cellulitis of toe (ICD10)
L03.10 Cellulitis of upper limb (ICD10)
L03.11 Cellulitis of lower limb (ICD10)
L03.2 Cellulitis of face (ICD10)
L03.3 Cellulitis of trunk (ICD10)
L03.8 Cellulitis of other sites (ICD10)
L03.9 Cellulitis, unspecified (ICD10)
L05.0 Pilonidal cyst with abscess
L05.9 Pilonidal cyst without abscess (ICD10)
L08.0 Pyoderma (ICD10)
L08.1 Erythrasma
L08.8 Other specified local infections of skin and subcutaneous tissue
L08.9 Local infection of the skin and subcutaneous tissue, unspecified
L13.0 Dermatitis herpetiformis
L30.3 Infective dermatitis
L88 Pyoderma gangrenosum
M60.09 Infective myositis, multiple sites
M63.0 Myositis in bacterial diseases classified elsewhere
M63.1 Myositis in protozoal and parasitic infections classified elsewhere
M63.2 Myositis in other infectious diseases classified elsewhere
M65.0 Abscess of tendon sheath
M65.1 Other infective (teno)synovitis
M68.0 Synovitis and tenosynovitis in bacterial diseases classified elsewhere
M71.0 Abscess of bursa
M71.1 Other infective bursitis

O91.00 Infection of nipple associated with childbirth, without mention of attachment difficulty
O91.10 Abscess of breast associated with childbirth, without mention of attachment difficulty
P39.0 Neonatal infective mastitis
P39.4 Neonatal skin infection

Supplementary Table: ICD codes for upper respiratory infection diagnostic group

032.0 FAUCIAL DIPHTHERIA (ICD9)
032.1 NASOPHARYNX DIPHTHERIA (ICD9)
032.2 ANT NASAL DIPHTHERIA (ICD9)
032.3 LARYNGEAL DIPHTHERIA (ICD9)
034.0 STREP SORE THROAT (ICD9)
040.1 RHINOSCLEROMA
055.2 POSTMEASLES OTITIS MEDIA (ICD9)
079.3 RHINOVIRUS INFECT NOS (ICD9)
381.00 AC NONSUP OTITIS MED NOS (ICD9)
381.01 AC SEROUS OTITIS MEDIA (ICD9)
381.02 AC MUCOID OTITIS MEDIA (ICD9)
381.03 ACUTE SANGUINOUS OTITIS MEDIA
381.10 CHR SEROUS OM SIMP/NOS (ICD9)
381.19 CHR SEROUS OM NEC (ICD9)
381.20 CHR MUCOID OM SIMP/NOS (ICD9)
381.29 CHR MUCOID OM NEC (ICD9)
381.3 CHR NONSUP OM NOS/NEC (ICD9)
381.4 NONSUPP OTITIS MEDIA NOS (ICD9)
382.00 AC SUPP OTITIS MEDIA NOS (ICD9)
382.01 AC SUPP OM W DRUM RUPT (ICD9)
382.02 AC SUPP OM IN OTH DIS (ICD9)
382.1 CHR TUBOTYMPAN SUPPUR OM (ICD9)
382.2 CHR ATTICOANTRAL SUP OM (ICD9)
382.3 CHR SUP OTITIS MEDIA NOS (ICD9)
382.4 SUPPUR OTITIS MEDIA NOS (ICD9)
382.9 OTITIS MEDIA NOS (ICD9)
383.00 AC MASTOIDITIS W/O COMPL (ICD9)
383.01 SUBPERI MASTOID ABSCESS (ICD9)
383.02 AC MASTOIDITIS-COMPL NEC (ICD9)
383.1 CHRONIC MASTOIDITIS (ICD9)
383.20 PETROSITIS NOS (ICD9)
383.21 ACUTE PETROSITIS (ICD9)
383.22 CHRONIC PETROSITIS (ICD9)
383.30 POSTMASTOID COMPL NOS (ICD9)
383.31 POSTMASTOID MUCOSAL CYST (ICD9)
383.32 POSTMASTOID CHOLESTEATMA (ICD9)
383.33 POSTMASTOID GRANULATIONS (ICD9)
383.89 DISORDERS OF MASTOID NEC (ICD9)
383.9 MASTOIDITIS NOS (ICD9)
384.00 ACUTE MYRINGITIS UNSPECIFIED
384.01 BULLOUS MYRINGITIS
384.09 OTHER ACUTE MYRINGITIS WITHOUT OTITIS MEDIA
384.20 PERFORAT TYMPAN MEMB NOS (ICD9)
384.21 CENT PERF TYMPANIC MEMB (ICD9)
384.22 ATTIC PERF TYMPANIC MEMB (ICD9)
384.23 MARGINAL PERF TYMP NEC (ICD9)
384.24 MULT PERF TYMPANIC MEMB (ICD9)
384.25 TOTAL PERF TYMPANIC MEMB (ICD9)
386.3 LABYRINTHITIS
386.30 LABYRINTHITIS UNSPECIFIED
386.31 SEROUS LABYRINTHITIS
386.32 CIRCUMSCRIBED LABYRINTHITIS
386.33 SUPPURATIVE LABYRINTHITIS
386.34 TOXIC LABYRINTHITIS
386.35 VIRAL LABYRINTHITIS
388.6 OTORRHEA
460 ACUTE NASOPHARYNGITIS (ICD9)
461.0 AC MAXILLARY SINUSITIS (ICD9)

461.1 AC FRONTAL SINUSITIS (ICD9)
461.2 AC ETHMOIDAL SINUSITIS (ICD9)
461.3 AC SPHENOIDAL SINUSITIS (ICD9)
461.8 OTHER ACUTE SINUSITIS (ICD9)
461.9 ACUTE SINUSITIS NOS (ICD9)
462 ACUTE PHARYNGITIS (ICD9)
463 ACUTE TONSILLITIS (ICD9)
464.0 ACUTE LARYNGITIS (End 2001) (ICD9)
464.00 ACUTE LARYNGITIS- W/O OBSTR (Begin 2001) (ICD9)
464.01 ACUTE LARYNGITIS- W OBSTR (Begin 2001) (ICD9)
464.10 AC TRACHEITIS NO OBSTRUC (ICD9)
464.11 AC TRACHEITIS W OBSTRUCT (ICD9)
464.20 AC LARYNGOTRACH NO OBSTR (ICD9)
464.21 AC LARYNGOTRACH W OBSTR (ICD9)
464.30 AC EPIGLOTTITIS NO OBSTR (ICD9)
464.31 AC EPIGLOTTITIS W OBSTR (ICD9)
464.50 SUPRAGLOTTIS NOS- W/O OBSTR (Begin 2001) (ICD9)
464.51 SUPRAGLOTTIS NOS- W/ OBSTR (Begin 2001) (ICD9)
465.0 ACUTE LARYNGOPHARYNGITIS (ICD9)
465.8 ACUTE URI MULT SITES NEC (ICD9)
465.9 ACUTE URI NOS (ICD9)
473.0 CHR MAXILLARY SINUSITIS (ICD9)
473.1 CHR FRONTAL SINUSITIS (ICD9)
473.2 CHR ETHMOIDAL SINUSITIS (ICD9)
473.3 CHR SPHENOIDAL SINUSITIS (ICD9)
473.8 CHRONIC SINUSITIS NEC (ICD9)
473.9 CHRONIC SINUSITIS NOS- (ICD9)
474.0 CHRONIC TONSILLITIS (End 1997) (ICD9)
474.00 CHRON TONSILLITIS (Begin 1997) (ICD9)
474.01 CHRON ADENOIDITIS (Begin 1997) (ICD9)
474.02 CHRON TONSIL ADENOID (Begin 1997) (ICD9)
475 PERITONSILLAR ABSCESS (ICD9)
476.0 CHRONIC LARYNGITIS
476.1 CHRONIC LARYNGOTRACHEITIS
478.21 CELLULITIS OF PHARYNX
478.22 PARAPHARYNGEAL ABSCESS
478.24 RETROPHARYNGEAL ABSCESS
478.29 DISEASE OF PHARYNX NEC
478.71 LARYNGEAL CELLULITIS

A36.0 Pharyngeal diphtheria (ICD10)
A36.1 Nasopharyngeal diphtheria (ICD10)
A36.2 Laryngeal diphtheria (ICD10)
B05.3+ Measles complicated by otitis media (ICD10)
H65.0 Acute serous otitis media (ICD10)
H65.1 Other acute nonsuppurative otitis media (ICD10)
H65.2 Chronic serous otitis media (ICD10)
H65.3 Chronic mucoid otitis media (ICD10)
H65.4 Other chronic nonsuppurative otitis media (ICD10)
H65.9 Nonsuppurative otitis media, unspecified (ICD10)
H66.0 Acute suppurative otitis media (ICD10)
H66.1 Chronic tubotympanic suppurative otitis media (ICD10)
H66.2 Chronic atticoantral suppurative otitis media (ICD10)
H66.3 Other chronic suppurative otitis media (ICD10)
H66.4 Suppurative otitis media, unspecified (ICD10)
H66.9 Otitis media, unspecified (ICD10)
H67.0 Otitis media in bacterial diseases classified elsewhere
H67.8* Otitis media in other diseases classified elsewhere (ICD10)
H68.0 Eustachian salpingitis
H70.0 Acute mastoiditis (ICD10)

H70.1 Chronic mastoiditis (ICD10)
H70.2 Petrositis (ICD10)
H70.8 Other mastoiditis and related conditions (ICD10)
H70.9 Mastoiditis, unspecified (ICD10)
H72.0 Central perforation of tympanic membrane (ICD10)
H72.1 Attic perforation of tympanic membrane (ICD10)
H72.2 Other marginal perforations of tympanic membrane (ICD10)
H72.8 Other perforations of tympanic membrane (ICD10)
H72.9 Perforation of tympanic membrane, unspecified (ICD10)
H73.0 Acute myringitis
H75.0 Mastoiditis in infectious and parasitic diseases classified elsewhere
H83.0 Labyrinthitis
H92.1 Otorrhoea
J00 Acute nasopharyngitis [common cold] (ICD10)
J01.0 Acute maxillary sinusitis (ICD10)
J01.1 Acute frontal sinusitis (ICD10)
J01.2 Acute ethmoidal sinusitis (ICD10)
J01.3 Acute sphenoidal sinusitis (ICD10)
J01.4 Acute pansinusitis
J01.8 Other acute sinusitis (ICD10)
J01.9 Acute sinusitis, unspecified (ICD10)
J02.0 Streptococcal pharyngitis (ICD10)
J02.8 Acute pharyngitis due to other specified organisms
J02.9 Acute pharyngitis, unspecified (ICD10)
J03.0 Streptococcal tonsillitis
J03.8 Acute tonsillitis due to other specified organisms
J03.9 Acute tonsillitis, unspecified (ICD10)
J04.0 Acute laryngitis (ICD10)
J04.1 Acute tracheitis (ICD10)
J04.2 Acute laryngotracheitis (ICD10)
J05.1 Acute epiglottitis (ICD10)
J06.0 Acute laryngopharyngitis (ICD10)
J06.8 Other acute upper respiratory infections of multiple sites (ICD10)
J06.9 Acute upper respiratory infection, unspecified (ICD10)
J32.0 Chronic maxillary sinusitis (ICD10)
J32.1 Chronic frontal sinusitis (ICD10)
J32.2 Chronic ethmoidal sinusitis (ICD10)
J32.3 Chronic sphenoidal sinusitis (ICD10)
J32.4 Chronic pansinusitis
J32.8 Other chronic sinusitis (ICD10)
J32.9 Chronic sinusitis, unspecified (ICD10)
J34.0 Abscess, furuncle and carbuncle of nose
J35.0 Chronic tonsillitis (ICD10)
J36 Peritonsillar abscess (ICD10)

Supplementary Table: ICD codes for genito-urinary infection diagnostic group

032.84 DIPHTHERITIC CYSTITIS (ICD9)
091.0 PRIMARY GENITAL SYPHILIS
091.1 PRIMARY ANAL SYPHILIS
091.2 PRIMARY SYPHILIS NEC
098.0 ACUTE GC INFECT LOWER GU (ICD9)
098.10 GC (ACUTE) UPPER GU NOS
098.11 GC CYSTITIS (ACUTE)
098.12 GC PROSTATITIS (ACUTE)
098.13 GC ORCHITIS (ACUTE)
098.14 GC SEM VESICULIT (ACUTE)
098.15 GC CERVICITIS (ACUTE)
098.16 GC ENDOMETRITIS (ACUTE)
098.17 ACUTE GC SALPINGITIS
098.19 GC (ACUTE) UPPER GU NEC
098.2 CHR GC INFECT LOWER GU
098.30 CHR GC UPPER GU NOS
098.31 GC CYSTITIS-CHRONIC
098.32 GC PROSTATITIS-CHRONIC
098.33 GC ORCHITIS-CHRONIC
098.34 GC SEM VESICULITIS-CHR
098.35 GC CERVICITIS-CHRONIC
098.36 GC ENDOMETRITIS-CHRONIC
098.37 GC SALPINGITIS (CHRONIC)
098.39 CHR GC UPPER GU NEC
099.0 CHANCROID
099.1 LYMPHOGRANULOMA VENEREUM
099.2 GRANULOMA INGUINALE
099.41 CHLAMYDIA-URETHRITIS
099.49 NONGONOCOCC URETHRIT NEC
099.50 CHLAMYDIA-UNSPEC SITE
099.51 CHLAMYDIA-PHARYNX
099.52 CHLAMYDIA-ANUS RECTUM
099.53 CHLAMYDIA-LOWER GU
099.54 CHLAMYDIA-OTHER GU
099.55 CHLAMYDIA-UNSPEC GU
099.56 CHLAMYDIA-PERITONEUM
099.59 CHLAMYDIA-NSC
112.1 CANDIDAL VULVOVAGINITIS
112.2 CANDIDIAS UROGENITAL NEC
590.2 RENAL/PERIRENAL ABSCESS (ICD9)
590.81 PYELITIS OR PYELONEPHRITIS IN DISEASES CLASSIFIED ELSEWHERE
590.9 INFECTION OF KIDNEY UNSPECIFIED
595.0 ACUTE CYSTITIS (ICD9)
595.89 OTHER CYSTITIS, INCL ABSCESS OF BLADDER (ICD9)
597.0 URETHRAL ABSCESS (ICD9)
598.00 URETHR STRICT:INFECT NOS (ICD9)
599.0 URIN TRACT INFECTION NOS (ICD9)
601.0 ACUTE PROSTATITIS (ICD9)
601.2 ABSCESS OF PROSTATE (ICD9)
603.1 INFECTED HYDROCELE (ICD9)
604.0 ORCHITIS WITH ABSCESS (ICD9)
614.0 AC SALPINGO-OOPHORITIS (ICD9)
614.1 CHR SALPINGO-OOPHORITIS (ICD9)
614.2 SALPINGO-OOPHORITIS NOS (ICD9)
614.3 ACUTE PARAMETRITIS (ICD9)
614.4 CHRONIC PARAMETRITIS (ICD9)
614.5 AC PELV PERITONITIS-FEM (ICD9)
614.6 FEM PELVIC PERITON ADHES (ICD9)

614.7 CHR PELV PERITON NEC-FEM (ICD9)
614.8 FEM PELV INFLAM DIS NEC (ICD9)
614.9 FEM PELV INFLAM DIS NOS (ICD9)
615.0 AC UTERINE INFLAMMATION (ICD9)
615.1 CHR UTERINE INFLAMMATION (ICD9)
615.9 UTERINE INFLAM DIS NOS (ICD9)
616.0 CERVICITIS (ICD9)
616.10 VAGINITIS NOS (ICD9)
616.11 VAGINITIS IN OTH DISEASE (ICD9)
616.2 BARTHOLIN-s GLAND CYST (ICD9)
616.3 BARTHOLIN-s GLND ABSCESS (ICD9)
616.4 ABSCESS OF VULVA NEC (ICD9)
634.00 SPON ABOR W PEL INF-UNSP (ICD9)
634.01 SPON ABOR W PELV INF-INC (ICD9)
634.02 SPON ABOR W PEL INF-COMP (ICD9)
646.60 GU INFECT IN PREG-UNSPEC (ICD9)
646.61 GU INFECTION-DELIVERED (ICD9)
646.62 GU INFECTION-DELIV W P/P (ICD9)
646.63 GU INFECTION-ANTEPARTUM (ICD9)
646.64 POSTPARTUM INFECTIONS OF GENITOURINARY TRACT
647.00 SYPHILIS OF MOTHER COMPLICATING PREGNANCY CHILDBIRTH OR THE PUERPERIUS UNSPECIFIED AS TO EPISODE OF CARE
647.01 SYPHILIS OF MOTHER COMPLICATING PREGNANCY WITH DELIVERY
647.02 SYPHILIS OF MOTHER COMPLICATING PREGNANCY WITH DELIVERY WITH POSTPARTUM COMPLICATION
647.03 ANTEPARTUM SYPHILIS
647.04 POSTPARTUM SYPHILIS
647.10 GONORRHEA OF MOTHER COMPLICATING PREGNANCY CHILDBIRTH OR THE PUERPERIUM UNSPECIFIED AS TO EPISODE OF CARE
647.11 GONORRHEA OF MOTHER WITH DELIVERY
647.12 GONORRHEA OF MOTHER WITH DELIVERY WITH POSTPARTUM COMPLICATION
647.13 ANTEPARTUM GONORRHEA
647.14 POSTPARTUM GONORRHEA
647.20 OTHER VENEREAL DISEASES OF MOTHER COMPLICATING PREGNANCY CHILDBIRTH OR PUERPERIUM UNSPECIFIED AS TO EPISODE OF CARE
647.21 OTHER VENEREAL DISEASES OF MOTHER WITH DELIVERY
647.22 OTHER VENEREAL DISEASES OF MOTHER WITH DELIVERY WITH POSTPARTUM COMPLICATION
647.23 OTHER ANTEPARTUM VENEREAL DISEASES
647.24 OTHER POSTPARTUM VENEREAL DISEASES
771.82 URINARY TRACT INFECTION OF NEWBORN (Begin 2002) (ICD9)
A36.8+ (no description found)
A51.0 Primary genital syphilis (ICD10)
A51.1 Primary anal syphilis (ICD10)
A51.2 Primary syphilis of other sites (ICD10)
A51.3 Secondary syphilis of skin and mucous membranes (ICD10)
A51.3+ (no description found)
A51.4 Other secondary syphilis (ICD10)
A51.5 Early syphilis, latent (ICD10)
A51.9 Early syphilis, unspecified
A52.9 Late syphilis, unspecified (ICD10)
A53.0 Latent syphilis, unspecified as early or late (ICD10)
A53.9 Syphilis, unspecified (ICD10)
A54.0 Gonococcal infection of lower genitourinary tract without periurethral or accessory gland abscess (ICD10)
A54.1 Gonococcal infection of lower genitourinary tract with periurethral and accessory gland abscess (ICD10)
A54.2+ Gonococcal pelviperitonitis and other gonococcal genitourinary infections (ICD10)
A54.3 Gonococcal infection of eye (ICD10)
A54.3+ (no description found)
A54.4+ Gonococcal infection of musculoskeletal system (ICD10)

A54.5 Gonococcal pharyngitis (ICD10)
A54.6 Gonococcal infection of anus and rectum (ICD10)
A54.8 Other gonococcal infections (ICD10)
A54.8+ (no description found)
A54.9 Gonococcal infection, unspecified
A55 Chlamydial lymphogranuloma (venereum) (ICD10)
A56.0 Chlamydial infection of lower genitourinary tract (ICD10)
A56.1 Chlamydial infection of pelviperitoneum and other genitourinary organs (ICD10)
A56.2 Chlamydial infection of genitourinary tract, unspecified (ICD10)
A56.3 Chlamydial infection of anus and rectum (ICD10)
A56.4 Chlamydial infection of pharynx (ICD10)
A56.8 Sexually transmitted chlamydial infection of other sites (ICD10)
A57 Chancroid (ICD10)
A58 Granuloma inguinale (ICD10)
A59.0 Urogenital trichomoniasis (ICD10)
A59.0+ (no description found)
A60.0 Herpesviral infection of genitalia and urogenital tract (ICD10)
A60.1 Herpesviral infection of perianal skin and rectum
A60.9 Anogenital herpesviral infection, unpsecified
A63.0 Anogenital (venereal) warts (ICD10)
A63.8 Other specified predominantly sexually transmitted diseases (ICD10)
A64 Unspecified sexually transmitted disease (ICD10)
B37.3+ Candidiasis of vulva and vagina (ICD10)
B37.4+ Candidiasis of other urogenital sites (ICD10)
N13.6 Pyonephrosis
N15.1 Renal and perinephric abscess (ICD10)
N30.0 Acute cystitis (ICD10)
N30.8 Other cystitis, abscess of bladder (ICD10)
N34.0 Urethral abscess (ICD10)
N35.1 Postinfective urethral stricture, not elsewhere classified (ICD10)
N39.0 Urinary tract infection, site not specified (ICD10)
N41.0 Acute prostatitis (ICD10)
N41.2 Abscess of prostate (ICD10)
N43.1 Infected hydrocele (ICD10)
N45.0 Orchitis, epididymitis and epididymo-orchitis with abscess (ICD10)
N45.9 Orchitis, epididymitis and epididymo-orchits without abscess
N51.2 Balanitis in diseases classified elsewhere
N70.0 Acute salpingitis and oophoritis (ICD10)
N73.0 Acute parametritis and pelvic cellulitis (ICD10)
N73.1 Chronic parametritis and pelvic cellulitis (ICD10)
N73.2 Unspecified parametritis and pelvic cellulitis
N73.3 Female acute pelvic peritonitis (ICD10)
N73.4 Female chronic pelvic peritonitis (ICD10)
N73.9 Female pelvic inflammatory disease, unspecified (ICD10)
N74.2 Female syphilitic pelvic inflammatory disease
N74.3 Female gonococcal pelvic inflammatory disease
N74.4 Female chlamydial pelvic inflammatory disease
N75.0 Cyst of Bartholins gland (ICD10)
N75.1 Abscess of Bartholins gland (ICD10)
N76.0 Acute vaginitis (ICD10)
N76.4 Abscess of vulva (ICD10)
O03.0 Spontaneous abortion, incomplete, complicated by genital tract and pelvic infection (ICD10)
O03.5 Spontaneous abortion, complete or unspecified, complicated by genital tract and pelvic infection (ICD10)
O08.0 Genital tract and pelvic infection following ectopic and molar pregnancy (ICD10)
O26.4 Herpes gestationis
O86.2 Urinary tract infection following delivery
O98.1 Syphilis complicating pregnancy, childbirth and the puerperium
O98.2 Gonorrhea complicating pregnancy, childbirth and the puerperium
P39.3 Neonatal urinary tract infection

Supplementary Table: ICD codes for viral infection diagnostic group

042	HIV DISEASE (Begin 1994) (ICD9)
042.0	HIV W/SPECIF INFECTIONS (Begin 1986 (ICD9)
042.1	HIV CAUS OTH SPEC INFECT (Begin 1986 (ICD9)
042.2	HIV W/SPEC MALIG NEOPLSM (Begin 1986 (ICD9)
042.9	AIDS- UNSPECIFIED (Begin 1986 (ICD9)
043.0	HIV CAUS LYMPHADENOPATHY (Begin 1986 (ICD9)
043.1	HIV CAUS SP CNS DISEASE (Begin 1986 (ICD9)
043.2	HIV CAUS OT DISOR IMMUNE (Begin 1986 (ICD9)
043.3	HIV CAUS OTH SPECIF COND (Begin 1986 (ICD9)
043.9	ARC- UNSPECIFIED (Begin 1986 (ICD9)
044.0	HIV CAUS ACUTE INFECTION (Begin 1986 (ICD9)
044.9	HIV- UNSPECIFIED (Begin 1986 (ICD9)
045.00	AC BULBAR POLIO-TYPE NOS (ICD9)
045.01	AC BULBAR POLIO-TYPE 1 (ICD9)
045.02	AC BULBAR POLIO-TYPE 2 (ICD9)
045.03	AC BULBAR POLIO-TYPE 3 (ICD9)
045.10	PARAL POLIO NEC-TYPE NOS (ICD9)
045.11	PARAL POLIO NEC-TYPE 1 (ICD9)
045.12	PARAL POLIO NEC-TYPE 2 (ICD9)
045.13	PARAL POLIO NEC-TYPE 3 (ICD9)
045.20	NONPARALY POLIO-TYPE NOS (ICD9)
045.21	NONPARALYT POLIO-TYPE 1 (ICD9)
045.22	NONPARALYT POLIO-TYPE 2 (ICD9)
045.23	NONPARALYT POLIO-TYPE 3 (ICD9)
045.90	AC POLIO NOS-TYPE NOS (ICD9)
045.91	AC POLIO NOS-TYPE 1 (ICD9)
045.92	AC POLIO NOS-TYPE 2 (ICD9)
045.93	AC POLIO NOS-TYPE 3 (ICD9)
046.8	CNS SLOW VIRUS INFEC NEC (ICD9)
046.9	CNS SLOW VIRUS INFEC NOS (ICD9)
047.0	COXSACKIE VIRUS MENING (ICD9)
047.1	ECHO VIRUS MENINGITIS (ICD9)
047.8	VIRAL MENINGITIS NEC (ICD9)
047.9	VIRAL MENINGITIS NOS (ICD9)
048	OTH ENTEROVIRAL CNS DIS (ICD9)
049.0	LYMPHOCYTIC CHORIOMENING (ICD9)
049.1	ADENOVIRAL MENINGITIS (ICD9)
049.8	VIRAL ENCEPHALITIS NEC (ICD9)
049.9	VIRAL ENCEPHALITIS NOS (ICD9)
050.0	VARIOLA MAJOR (ICD9)
050.1	ALASTRIM (ICD9)
050.2	MODIFIED SMALLPOX (ICD9)
050.9	SMALLPOX NOS (ICD9)
051.0	COWPOX (ICD9)
051.01	(no description found)
051.02	(no description found)
051.1	PSEUDOCOWPOX (ICD9)
051.2	CONTAGIOUS PUSTULAR DERM (ICD9)
051.9	PARAVACCINIA NOS (ICD9)
052.0	POSTVARICELLA ENCEPHALIT (ICD9)
052.2	POSTVARICELLA MYELITIS (Begin 2006) (ICD9)
052.7	VARICELLA COMPLICAT NEC (ICD9)
052.8	VARICELLA COMPLICAT NOS (ICD9)
052.9	VARICELLA UNCOMPLICATED (ICD9)
053.0	HERPES ZOSTER MENINGITIS (ICD9)
053.10	H ZOSTER NERV SYST NOS (ICD9)
053.11	GENICULATE HERPES ZOSTER (ICD9)

053.12 POSTHERPES TRIGEM NEURAL (ICD9)
053.13 POSTHERPES POLYNEUROPATH (ICD9)
053.14 HERPES ZOSTER MYELITIS (Begin 2006) (ICD9)
053.19 H ZOSTER NERV SYST NEC (ICD9)
053.20 HERPES ZOSTER OF EYELID (ICD9)
053.21 H ZOSTER KERATOCONJUNCT (ICD9)
053.22 H ZOSTER IRIDOCYCLITIS (ICD9)
053.29 HERPES ZOSTER OF EYE NEC (ICD9)
053.71 H ZOSTER OTITIS EXTERNA (ICD9)
053.79 H ZOSTER COMPLICATED NEC (ICD9)
053.8 H ZOSTER COMPLICATED NOS (ICD9)
053.9 HERPES ZOSTER NOS (ICD9)
054.10 GENITAL HERPES NOS (ICD9)
054.11 HERPETIC VULVOVAGINITIS (ICD9)
054.12 HERPETIC ULCER OF VULVA (ICD9)
054.13 HERPETIC INFECT OF PENIS (ICD9)
054.19 GENITAL HERPES NEC (ICD9)
054.2 HERPETIC GINGIVOSTOMAT (ICD9)
054.3 HERPETIC ENCEPHALITIS (ICD9)
054.40 HERPES SIMPLEX EYE NOS (ICD9)
054.41 HERPES SIMPLEX OF EYELID (ICD9)
054.42 DENDRITIC KERATITIS (ICD9)
054.43 H SIMPLEX KERATITIS (ICD9)
054.44 H SIMPLEX IRIDOCYCLITIS (ICD9)
054.49 HERPES SIMPLEX EYE NEC (ICD9)
054.5 HERPETIC SEPTICEMIA (ICD9)
054.6 HERPETIC WHITLOW (ICD9)
054.71 VISCERAL HERPES SIMPLEX (ICD9)
054.72 H SIMPLEX MENINGITIS (ICD9)
054.73 H SIMPLEX OTITIS EXTERNA (ICD9)
054.74 HERPES SIMPLEX MYELITIS (Begin 2006) (ICD9)
054.79 H SIMPLEX COMPLICAT NEC (ICD9)
054.8 H SIMPLEX COMPLICAT NOS (ICD9)
054.9 HERPES SIMPLEX NOS (ICD9)
055.0 POSTMEASLES ENCEPHALITIS (ICD9)
055.71 MEASLES KERATITIS (ICD9)
055.79 MEASLES COMPLICATION NEC (ICD9)
055.8 MEASLES COMPLICATION NOS (ICD9)
055.9 MEASLES UNCOMPLICATED (ICD9)
056.00 RUBELLA NERVE COMPL NOS (ICD9)
056.01 RUBELLA ENCEPHALITIS (ICD9)
056.09 RUBELLA NERVE COMPL NEC (ICD9)
056.71 ARTHRITIS DUE TO RUBELLA (ICD9)
056.79 RUBELLA COMPLICATION NEC (ICD9)
056.8 RUBELLA COMPLICATION NOS (ICD9)
056.9 RUBELLA UNCOMPLICATED (ICD9)
057.0 ERYTHEMA INFECTIOSUM (ICD9)
057.8 VIRAL EXANTHEMATA NEC (ICD9)
057.9 VIRAL EXANTHEMATA NOS (ICD9)
058.10 ROSEOLA INFANTUM NOS (Begin 2007) (ICD9)
058.11 ROSEOLA INFANT D/T HHV-6 (Begin 2007) (ICD9)
058.12 ROSEOLA INFANT D/T HHV-7 (Begin 2007) (ICD9)
058.21 HUMAN HERPESVIR 6 ENCEPH (Begin 2007) (ICD9)
058.29 HUMAN HERPESVR ENCPH NEC (Begin 2007) (ICD9)
058.81 HUMAN HERPESVIRUS 6 INFC (Begin 2007) (ICD9)
058.82 HUMAN HERPESVIRUS 7 INFC (Begin 2007) (ICD9)
058.89 HUMAN HERPESVIRS INF NEC (Begin 2007) (ICD9)
059.00 ORTHOPOXVIRUS INFECTION, UNSPECIFIED
059.01 MONKEYPOX
059.09 OTHER ORTHOPOXVIRUS INFECTIONS

059.10 PARAPOXVIRUS INFECTION, UNSPECIFIED
059.11 BOVINE STOMATITIS
059.12 SEALPOX
059.19 OTHER PARAPOXVIRUS INFECTIONS
059.20 YATAPOXVIRUS INFECTION, UNSPECIFIED
059.21 TANAPOX
059.22 YABA MONKEY TUMOR VIRUS
059.8 OTHER POXVIRUS INFECTIONS
059.9 POXVIRUS INFECTIONS, UNSPECIFIED
060.0 SYLVATIC YELLOW FEVER (ICD9)
060.1 URBAN YELLOW FEVER (ICD9)
060.9 YELLOW FEVER NOS (ICD9)
061 DENGUE (ICD9)
062.0 JAPANESE ENCEPHALITIS (ICD9)
062.1 WEST EQUINE ENCEPHALITIS (ICD9)
062.2 EAST EQUINE ENCEPHALITIS (ICD9)
062.3 ST LOUIS ENCEPHALITIS (ICD9)
062.4 AUSTRALIAN ENCEPHALITIS (ICD9)
062.5 CALIFORNIA ENCEPHALITIS (ICD9)
062.8 MOSQUIT-BORNE ENCEPH NEC (ICD9)
062.9 MOSQUIT-BORNE ENCEPH NOS (ICD9)
063.0 RUSSIA SPR-SUMMER ENCEPH (ICD9)
063.1 LOUPING ILL (ICD9)
063.2 CENT EUROPE ENCEPHALITIS (ICD9)
063.8 TICK-BORNE ENCEPH NEC (ICD9)
063.9 TICK-BORNE ENCEPH NOS (ICD9)
064 VIR ENCEPH ARTHROPOD NEC (ICD9)
065.0 CRIMEAN HEMORRHAGIC FEV (ICD9)
065.1 OMSK HEMORRHAGIC FEVER (ICD9)
065.2 KYASANUR FOREST DISEASE (ICD9)
065.3 TICK-BORNE HEM FEVER NEC (ICD9)
065.4 MOSQUITO-BORNE HEM FEVER (ICD9)
065.8 ARTHROPOD HEM FEVER NEC (ICD9)
065.9 ARTHROPOD HEM FEVER NOS (ICD9)
066.0 PHLEBOTOMUS FEVER (ICD9)
066.1 TICK-BORNE FEVER (ICD9)
066.2 VENEZUELAN EQUINE FEVER
066.3 MOSQUITO-BORNE FEVER NEC (ICD9)
066.4 WEST NILE FEVER (Begin 2002 (ICD9)
066.40 WEST NILE FEVER NOS (Begin 2004) (ICD9)
066.41 WEST NILE FEVER W/ENCEPH (Begin 2004) (ICD9)
066.42 WEST NILE NEURO MAN NEC (Begin 2004) (ICD9)
066.49 WEST NILE W COMPLIC NEC (Begin 2004) (ICD9)
066.8 ARTHROPOD VIRUS NEC (ICD9)
066.9 ARTHROPOD VIRUS NOS (ICD9)
070.0 HEPATITIS A WITH COMA (ICD9)
070.1 HEPATITIS A W/O COMA (ICD9)
070.2 HEPATITIS B WITH COMA (Begin 1980 (ICD9)
070.20 VRL HEPAT B CM W/O DELTA (Begin 1991) (ICD9)
070.21 VRL HEPAT B CM W DELTA (Begin 1991) (ICD9)
070.22 CHR HEPAT COMA W/O DELTA (Begin 1994) (ICD9)
070.23 CHR HEPAT COMA W/ DELTA (Begin 1994) (ICD9)
070.3 HEPATITIS B W/O COMA (Begin 1980 (ICD9)
070.30 VRL HPT B W/O CM W/O DLT (Begin 1991) (ICD9)
070.31 VRL HPT B W/O CM W DELTA (Begin 1991) (ICD9)
070.32 CHR HEPAT W/O COMA W/O DELTA (Begin 1994) (ICD9)
070.33 CHR HEPAT W/O COMA W/ DELTA (Begin 1994) (ICD9)
070.4 VIRAL HEPAT NEC W COMA (Begin 1980 (ICD9)
070.41 SPF VRL HPT CM HPT C (Begin 1991) (ICD9)
070.42 SPF VRL HPT CM DLT W/O B (Begin 1991) (ICD9)

070.43 SPF VRL HPT CM HPT E (Begin 1991) (ICD9)
070.44 CHR HEPAT C W/ COMA (Begin 1994) (ICD9)
070.49 SPF VRL HPT CM (Begin 1991) (ICD9)
070.5 VIRAL HEPAT NEC W/O COMA (Begin 1980 (ICD9)
070.51 VRL HPT W/O CM HEPAT C (Begin 1991) (ICD9)
070.52 VRL HPT W/O CM DLT W/O B (Begin 1991) (ICD9)
070.53 VRL HPT W/O CM HEPAT E (Begin 1991) (ICD9)
070.54 CHR HEPAT C W/O COMA (Begin 1994) (ICD9)
070.59 VRL HPT W/O CM (Begin 1991) (ICD9)
070.6 VIRAL HEPAT NOS W COMA (ICD9)
070.70 HPT C W/O HEPAT COMA NOS (Begin 2004) (ICD9)
070.71 HPT C W HEPATIC COMA NOS (Begin 2004) (ICD9)
070.9 VIRAL HEPAT NOS W/O COMA (ICD9)
071 RABIES (ICD9)
072.0 MUMPS ORCHITIS (ICD9)
072.1 MUMPS MENINGITIS (ICD9)
072.2 MUMPS ENCEPHALITIS (ICD9)
072.3 MUMPS PANCREATITIS (ICD9)
072.71 MUMPS HEPATITIS (ICD9)
072.72 MUMPS POLYNEUROPATHY (ICD9)
072.79 MUMPS COMPLICATION NEC (ICD9)
072.8 MUMPS COMPLICATION NOS (ICD9)
072.9 MUMPS UNCOMPLICATED (ICD9)
074.0 HERPANGINA (ICD9)
074.1 EPIDEMIC PLEURODYNIA (ICD9)
074.20 COXSACKIE CARDITIS UNSPECIFIED
074.21 COXSACKIE PERICARDITIS
074.22 COXSACKIE ENDOCARDITIS
074.23 COXSACKIE MYOCARDTIS
074.3 HAND- FOOT & MOUTH DIS (ICD9)
074.8 COXSACKIE VIRUS NEC (ICD9)
075 INFECTIOUS MONONUCLEOSIS (ICD9)
077.1 EPIDEM KERATOCONJUNCTIV (ICD9)
077.2 PHARYNGOCONJUNCT FEVER (ICD9)
077.3 ADENOVIRAL CONJUNCT NEC (ICD9)
077.4 EPIDEM HEM CONJUNCTIVIT (ICD9)
077.8 VIRAL CONJUNCTIVITIS NEC (ICD9)
077.99 DIS OF CONJUNCT DUE TO VIRUSES (Begin 1993) (ICD9)
078.0 MOLLUSCUM CONTAGIOSUM (ICD9)
078.1 VIRAL WARTS (End 1993) (ICD9)
078.10 VIRAL WARTS UNSPEC (Begin 1993) (ICD9)
078.11 CONDYLOMA ACCUMINATUM (Begin 1993) (ICD9)
078.12 (no description found)
078.19 OTHER SPEC VIRAL WARTS (Begin 1993) (ICD9)
078.2 SWEATING FEVER(ICD9)
078.4 FOOT & MOUTH DISEASE (ICD9)
078.5 CYTOMEGAL INCLUSION DIS (ICD9)
078.6 HEM NEPHROSONEPHRITIS (ICD9)
078.7 ARENAVIRAL HEM FEVER (ICD9)
078.81 EPIDEMIC VERTIGO (ICD9)
078.89 VIRAL DISEASE NEC (ICD9)
079.1 ECHO VIRUS INFECT NOS (ICD9)
079.2 COXSACKIE VIRUS INF NOS (ICD9)
079.4 HUMAN PAPILLOMA VIRUS (Begin 1993) (ICD9)
079.50 UNSPEC RETROVIRUS (Begin 1993) (ICD9)
079.51 HTLV TYPE I (Begin 1993) (ICD9)
079.52 HTLV TYPE II (Begin 1993) (ICD9)
079.53 HIV TYPE 2 (Begin 1993) (ICD9)
079.59 OTH SPEC RETROVIRUS (Begin 1993) (ICD9)
079.81 HANTAVIRUS INFECTION (Begin 1995) (ICD9)

079.83 PARVOVIRUS B19 (Begin 2007) (ICD9)
079.89 OTH SPEC VIRAL INFECTION (Begin 1993) (ICD9)
079.99 VIRAL INFECTION NOS (Begin 1993) (ICD9)
323.0 ENCEPHALIT IN VIRAL DIS (ICD9)
323.02 MYELITIS-OTH VIRAL DIS (Begin 2006) (ICD9)
488.09 FLU D/T IDENTIFIED AVIAN FLU VIRUS WITH OTHER MANIFESTATIONS
488.19 FLU D/T IDENTIFIED 2009 H1N1 FLU VIRUS WITH OTHER MANIFESTATIONS
488.89 FLU D/T IDENTIFIED NOVEL INFLUENZA A VIRUS W/ OTHER MANIFESTATIONS
573.1 HEPATITIS IN VIRAL DIS (ICD9)
647.50 RUBELLA IN PREG-UNSPEC (ICD9)
647.51 RUBELLA-DELIVERED (ICD9)
647.52 RUBELLA-DELIVERED W P/P (ICD9)
647.53 RUBELLA-ANTEPARTUM (ICD9)
647.54 RUBELLA-POSTPARTUM (ICD9)
647.60 OTH VIRUS IN PREG-UNSPEC (ICD9)
647.61 OTH VIRAL DIS-DELIVERED (ICD9)
647.62 OTH VIRAL DIS-DEL W P/P (ICD9)
647.63 OTH VIRAL DIS-ANTEPARTUM (ICD9)
647.64 OTH VIRAL DIS-POSTPARTUM (ICD9)
695.3 ROSACEA (ICD9)
711.50 VIRAL ARTHRITIS-UNSPEC (ICD9)
711.51 VIRAL ARTHRITIS-SHLDER (ICD9)
711.52 VIRAL ARTHRITIS-UP/ARM (ICD9)
711.53 VIRAL ARTHRITIS-FOREARM (ICD9)
711.54 VIRAL ARTHRITIS-HAND (ICD9)
711.55 VIRAL ARTHRITIS-PELVIS (ICD9)
711.56 VIRAL ARTHRITIS-L/LEG (ICD9)
711.57 VIRAL ARTHRITIS-ANKLE (ICD9)
711.58 VIRAL ARTHRITIS NEC (ICD9)
711.59 VIRAL ARTHRITIS-MULT (ICD9)
771.0 CONGENITAL RUBELLA (ICD9)
771.1 CONGENITAL CYTOMEGALOVIRUS INFECTION (ICD9)
790.8 VIREMIA NOS- (ICD9)
795.8 POSITIVE SERO/VIRAL HIV (Begin 1986 (ICD9)
V08 HIV POSITIVE NOS (Begin 1994) (ICD9)

A80.1 Acute paralytic poliomyelitis, wild virus, imported
A80.2 Acute paralytic poliomyelitis, wild virus, indigenous
A80.3 Acute paralytic poliomyelitis, other and unspecified (ICD10)
A80.4 Acute nonparalytic poliomyelitis (ICD10)
A80.9 Acute poliomyelitis, unspecified (ICD10)
A81.8 Other atypical virus infections of central nervous system (ICD10)
A81.9 Atypical virus infection of central nervous system, unspecified
A82.0 Sylvatic rabies
A82.1 Urban rabies
A82.9 Rabies, unspecified (ICD10)
A83.0 Japanese encephalitis (ICD10)
A83.1 Western equine encephalitis (ICD10)
A83.2 Eastern equine encephalitis (ICD10)
A83.3 St Louis encephalitis (ICD10)
A83.4 Australian encephalitis (ICD10)
A83.5 California encephalitis (ICD10)
A83.6 Rocio virus disease
A83.8 Other mosquito-borne viral encephalitis (ICD10)
A83.9 Mosquito-borne viral encephalitis, unspecified (ICD10)
A84.0 Far Eastern tick-borne encephalitis [Russian spring-summer encephalitis] (ICD10)
A84.1 Central European tick-borne encephalitis (ICD10)
A84.8 Other tick-borne viral encephalitis (ICD10)
A84.9 Tick-borne viral encephalitis, unspecified (ICD10)
A85.0 Enteroviral encephalitis

A85.1 Adenoviral encephalitis
A85.2 Arthropod-borne viral encephalitis, unspecified (ICD10)
A85.8 Other specified viral encephalitis
A86 Unspecified viral encephalitis
A87.0+ Enteroviral meningitis (ICD10)
A87.1+ Adenoviral meningitis (ICD10)
A87.2 Lymphocytic choriomeningitis (ICD10)
A87.8 Other viral meningitis
A87.9 Viral meningitis, unspecified
A88.0 Enteroviral exanthematous fever
A88.8 Other specified viral infections of central nervous system (ICD10)
A89 Unspecified viral infection of central nervous system (ICD10)
A90 Dengue fever [classical dengue] (ICD10)
A91 Dengue haemorrhagic fever
A92.0 Chikungunya virus disease
A92.1 O'nyong-nyong fever
A92.2 Venezuelan equine fever (ICD10)
A92.3 West Nile virus infection
A92.4 Rift Valley fever
A92.8 Other specified mosquito-borne viral fevers (ICD10)
A92.9 Mosquito-borne viral fever, unspecified
A93.0 Oropouche virus disease
A93.2 Colorado tick fever (ICD10)
A93.8 Other specified arthropod-borne viral fevers (ICD10)
A94 Unspecified arthropod-borne viral fever (ICD10)
A95.0 Sylvatic yellow fever (ICD10)
A95.1 Urban yellow fever (ICD10)
A95.9 Yellow fever, unspecified (ICD10)
A96.0 Junin haemorrhagic fever
A96.1 Machupo haemorrhagic fever
A96.2 Lassa fever
A96.8 Other arenaviral hemorrhagic fevers (ICD10)
A96.9 Arenaviral haemorrhagic fever, unspecified
A98.0 Crimean-Congo hemorrhagic fever (ICD10)
A98.1 Omsk hemorrhagic fever (ICD10)
A98.2 Kyasanur Forest disease (ICD10)
A98.3 Marburg virus disease
A98.4 Ebola virus disease
A98.5 Hemorrhagic fever with renal syndrome (ICD10)
A98.8 Other specified viral haemorrhagic fevers
A99 Unspecified viral haemorrhagic fever
B00.1 Herpesviral vesicular dermatitis (ICD10)
B00.2 Herpesviral gingivostomatitis and pharyngotonsillitis (ICD10)
B00.3+ Herpesviral meningitis (ICD10)
B00.4+ Herpesviral encephalitis (ICD10)
B00.5+ Herpesviral ocular disease (ICD10)
B00.7 Disseminated herpesviral disease (ICD10)
B00.8 Other forms of herpesviral infection (ICD10)
B00.9 Herpesviral infection, unspecified (ICD10)
B01.0 Varicella meningitis
B01.1+ Varicella encephalitis (G05.1*) (ICD10)
B01.8 Varicella with other complications (ICD10)
B01.9 Varicella without complication (ICD10)
B02.0 Zoster encephalitis
B02.1+ Zoster meningitis (ICD10)
B02.2+ Zoster with other nervous system involvement (ICD10)
B02.3+ Zoster ocular disease (ICD10)
B02.7 Disseminated zoster
B02.8 Zoster with other complications (ICD10)
B02.9 Zoster without complications (ICD10)

B03 Smallpox (ICD10)
B04 Monkeypox
B05.0+ Measles complicated by encephalitis (ICD10)
B05.1 Measles complicated by meningitis
B05.4 Measles with intestinal complications
B05.8 Measles with other complications (ICD10)
B05.9 Measles without complication (ICD10)
B06.0+ Rubella with neurological complications (ICD10)
B06.8 Rubella with other complications (ICD10)
B06.9 Rubella without complication (ICD10)
B07 Viral warts (ICD10)
B08.0 Other orthopoxvirus infections (ICD10)
B08.1 Molluscum contagiosum (ICD10)
B08.2 Exanthema subitum [sixth disease]
B08.3 Erythema infectiosum [fifth disease] (ICD10)
B08.4 Enteroviral vesicular stomatitis with exanthem (ICD10)
B08.5 Enteroviral vesicular pharyngitis (ICD10)
B08.8 Other specified viral infections characterized by skin and mucous membrane lesions (ICD10)
B09 Unspecified viral infection characterized by skin and mucous membrane lesions (ICD10)
B15.0 Hepatitis A with hepatic coma (ICD10)
B15.9 Hepatitis A without hepatic coma (ICD10)
B16.0 Acute hepatitis B with delta-agent with hepatic coma (ICD10)
B16.1 Acute hepatitis B with delta-agent without hepatic coma (ICD10)
B16.2 Acute hepatitis B without delta-agent with hepatic coma (ICD10)
B16.9 Acute hepatitis B without delta-agent and without hepatic coma
B17.0 Acute delta-(super) infection of hepatitis B carrier (ICD10)
B17.1 Acute hepatitis C (ICD10)
B17.2 Acute hepatitis E (ICD10)
B17.8 Other specified acute viral hepatitis (ICD10)
B17.9 Acute viral hepatitis, unspecified
B18.0 Chronic viral hepatitis B with delta-agent (ICD10)
B18.1 Chronic viral hepatitis B without delta-agent (ICD10)
B18.2 Chronic viral hepatitis C (ICD10)
B18.8 Other chronic viral hepatitis
B18.9 Chronic viral hepatitis, unspecified
B19.0 Unspecified viral hepatitis with hepatic coma (ICD10)
B19.9 Unspecified viral hepatitis without hepatic coma (ICD10)
B20.0 HIV disease resulting in mycobacterial infection
B20.1 HIV disease resulting in other bacterial infections
B20.2 HIV disease resulting in cytomegaloviral disease
B20.3 HIV disease resulting in other viral infections
B20.4 HIV disease resulting in candidiasis
B20.5 HIV disease resulting in other mycoses
B20.6 HIV disease resulting in Pneumocystis jirovecii pneumonia
B20.7 HIV disease resulting in multiple infections
B20.8 HIV disease resulting in other infectious and parasitic diseases
B20.9 HIV disease resulting in unspecified infectious or parasitic
B21.0 HIV disease resulting in Kaposi sarcoma
B21.1 HIV disease resulting in Burkitt lymphoma
B21.2 HIV disease resulting in other types of non-Hodgkin lymphoma
B21.3 HIV disease resulting in other malignant neoplasms of lymphoid, haematopoietic and related tissue
B21.7 HIV disease resulting in multiple malignant neoplasms
B21.8 HIV disease resulting in other malignant neoplasms
B21.9 HIV disease resulting in unspecified malignant neoplasm
B22.0 HIV disease resulting in encephalopathy
B22.1 HIV disease resulting in lymphoid interstitial pneumonitis
B22.2 HIV disease resulting in wasting syndrome
B22.7 HIV disease resulting in multiple diseases classified elsewhere
B23.0 Acute HIV infection syndrome
B23.1 HIV disease resulting in (persistent) generalized lymphadenopathy

B23.2 HIV disease resulting in haematological and immunological abnormalities, not elsewhere classified
B23.8 HIV disease resulting in other specified conditions
B24 Unspecified human immunodeficiency virus [HIV] disease (ICD10)
B25.0 Cytomegaloviral pneumonitis
B25.1 Cytomegaloviral hepatitis
B25.2 Cytomegaloviral pancreatitis
B25.8 Other cytomegaloviral diseases
B25.9 Cytomegaloviral disease, unspecified (ICD10)
B26.0+ Mumps orchitis (ICD10)
B26.1+ Mumps meningitis (ICD10)
B26.2+ Mumps encephalitis (ICD10)
B26.3+ Mumps pancreatitis (ICD10)
B26.8 Mumps with other complications (ICD10)
B26.8+ (no description found)
B26.9 Mumps without complication (ICD10)
B27.0 Gammaherpesviral mononucleosis
B27.1 Cytomegaloviral mononucleosis
B27.8 Other infectious mononucleosis
B27.9 Infectious mononucleosis, unspecified (ICD10)
B30.0+ Keratoconjunctivitis due to adenovirus (ICD10)
B30.1+ Conjunctivitis due to adenovirus (ICD10)
B30.2+ (no description found)
B30.3+ Acute epidemic hemorrhagic conjunctivitis (enteroviral)
B30.8+ Other viral conjunctivitis (ICD10)
B30.9 Viral conjunctivitis, unspecified
B33.0 Epidemic myalgia (ICD10)
B33.1 Ross River disease
B33.2 Viral carditis
B33.3 Retrovirus infections, not elsewhere classified (ICD10)
B33.4 Hantavirus (cardio-) pulmonary syndrome
B33.8 Other specified viral diseases (ICD10)
B34.1 Enterovirus infection, unspecified (ICD10)
B34.2 Coronavirus infection, unspecified site
B34.3 Parvovirus infection, unspecified site
B34.4 Papovavirus infection, unspecified (ICD10)
B34.8 Other viral infections of unspecified site (ICD10)
B34.9 Viral infection, unspecified (ICD10)
B97.0 Adenovirus as the cause of diseases classified to other chapters
B97.1 Enterovirus as the cause of diseases classified to other chapters
B97.2 Coronavirus as the cause of diseases classified to other chapters
B97.3 Retrovirus as the cause of diseases classified to other chapters
B97.4 Respiratory syncytial virus as the cause of diseases classified to other chapters
B97.5 Reovirus as the cause of diseases classified to other chapters
B97.6 Parvovirus as the cause of diseases classified to other chapters
B97.7 Papillomavirus as the cause of diseases classified to other chapters
B97.8 Other viral agents as the cause of diseases classified to other chapters
G02.0* Meningitis in viral diseases classified elsewhere (ICD10)
G05.1* Encephalitis, myelitis and encephalomyelitis in viral diseases classified elsewhere (ICD10)
H19.1 Herpesviral keratitis and keratoconjunctivitis
I41.1 Myocarditis in viral diseases classified elsewhere
J10.8 Influenza with other manifestations, influenza virus identified
J11 Influenza, virus not identified (ICD10)
J11.8 Influenza with other manifestations, virus not identified (ICD10)
M01.4 Rubella arthritis
M01.50* Arthritis in other viral diseases classified elsewhere, multiple sites (ICD10)
M01.51* Arthritis in other viral diseases classified elsewhere, shoulder region (ICD10)
M01.52* Arthritis in other viral diseases classified elsewhere, upper arm (ICD10)
M01.53* Arthritis in other viral diseases classified elsewhere, forearm (ICD10)
M01.54* Arthritis in other viral diseases classified elsewhere, hand (ICD10)
M01.55* Arthritis in other viral diseases classified elsewhere, pelvic region and thigh (ICD10)

M01.56* Arthritis in other viral diseases classified elsewhere, lower leg (ICD10)
M01.57* Arthritis in other viral diseases classified elsewhere, ankle and foot (ICD10)
M01.58* Arthritis in other viral diseases classified elsewhere, other site (ICD10)
M01.59* Arthritis in other viral diseases classified elsewhere, site unspecified (ICD10)
O98.4 Viral hepatitis complicating pregnancy, childbirth and the puerperium
O98.5 Other viral diseases complicating pregnancy, childbirth and the puerperium (ICD10)
P35.0 Congenital rubella syndrome
P35.1 Congenital cytomegalovirus infection
P35.2 Congenital herpesviral [herpes simples] infection
P35.3 Congenital viral hepatitis
P35.8 Other congenital viral diseases
P35.9 Congenital viral disease, unspecified
Z21 Asymptomatic human immunodeficiency virus [HIV] infection status

Supplementary Table: Numbers of participants followed within each age stratum and the rate of infection-related hospitalisation in infection categories.

			Age period specific rate per 1000py							
Age Period	Number of individual followed	Person years contributed	Any infection	Invasive Bacterial	Gastrointestinal	Lower Respiratory Tract	Skin and Soft Tissue	Upper Respiratory Tract	Genitourinary	Other Viral
0-28d	711408	37992.6	148.2	9.0	14.8	30.4	10.6	21.2	15.4	19.8
1-3mos	716828	177250.0	112.6	2.5	16.6	49.6	3.1	20.1	11.0	15.0
4-6mos	709550	173918.8	96.8	1.8	16.8	45.3	2.8	22.4	6.6	10.5
7-12mos	702352	295414.1	105.6	1.8	21.9	37.0	3.2	35.8	4.4	11.5
1-2yr	689714	676796.7	99.0	1.7	17.9	23.2	3.4	45.6	2.3	10.9
2-5yr	661124	1860369.6	55.0	0.7	6.1	9.6	2.0	30.3	1.1	4.6
5-10yr	579952	2610814.5	30.0	0.4	1.9	3.2	1.4	18.5	0.6	2.6
10-14yr	466066	1681040.4	13.9	0.4	1.2	1.3	1.2	6.8	0.4	1.9
14-18yr	373978	1311430.5	16.2	0.5	1.0	1.2	2.1	6.8	1.4	2.1

Supplementary Table: Change in rate for gestational age, birthweight, and birth length by infection category.

Rate increase for 1 week decrease in gestational age from 39-40 weeks			
	IRR	95% CI	%increase
Any infection	1.12	1.12 - 1.13	12.34
Invasive Bacterial	1.10	1.08 - 1.13	10.31
Gastrointestinal	1.12	1.10 - 1.13	11.67
Lower Respiratory Tract	1.20	1.19 - 1.21	19.86
Skin and Soft Tissue	1.08	1.06 - 1.10	8.34
Upper Respiratory Tract	1.10	1.10 - 1.11	10.36
Genitourinary	1.13	1.10 - 1.16	13.09
Other Viral	1.13	1.12 - 1.14	13.1

Rate increase for 500g decrease in birthweight from 3000-3500g			
	IRR	95% CI	%increase
Any infection	1.20	1.18 - 1.21	19.69
Invasive Bacterial	1.22	1.16 - 1.29	22.45
Gastrointestinal	1.20	1.18 - 1.23	20.49
Lower Respiratory Tract	1.33	1.31 - 1.35	32.99
Skin and Soft Tissue	1.16	1.11 - 1.2	15.60
Upper Respiratory Tract	1.16	1.14 - 1.18	16.04
Genitourinary	1.20	1.15 - 1.26	20.23
Other Viral	1.21	1.18 - 1.23	20.91

Rate increase for 5cm decrease in birth length from 46-50cm			
	IRR	95% CI	%increase
Any infection	1.41	1.38 - 1.45	41.04
Invasive Bacterial	1.44	1.3 - 1.61	44.48
Gastrointestinal	1.39	1.33 - 1.46	39.12
Lower Respiratory Tract	1.71	1.64 - 1.78	70.83
Skin and Soft Tissue	1.33	1.21 - 1.46	33.02
Upper Respiratory Tract	1.33	1.29 - 1.37	32.81
Genitourinary	1.43	1.27 - 1.61	43.12
Other Viral	1.44	1.38 - 1.51	44.16

Adjusted for maternal age (<20,20-24,25-29,30-24, >=35 years), parity (no previous birth/yes previous birth), birth cohort (2 year blocks), season of birth, sex, mode of delivery (vaginal/caesarean), 5 minute APGAR score (0-7,8-10), bronchopulmonary dysplasia (present/absent), socioeconomic status (percentile: >90 (highest), 75-90, 50-75, 25-50, 10-25, <10).

Supplementary Table: Overall adjusted childhood infection-related hospitalisation rate ratio and 95% confidence interval in total population and in children not considered to be at high risk.

	Total Population		No neurodevelopmental disability		No Complications during Pregnancy		No Medical Conditions during Pregnancy	
**Gestational Age (wks)**	n=719,311		n=259,752		n=482,036		n=570,428	
<28	2.91	(2.55-3.33)	3.47	(2.84-4.23)	2.12	(1.47-3.06)	2.86	(2.40-3.42)
28-29	2.49	(2.24-2.78)	2.78	(2.32-3.32)	2.52	(1.84-3.44)	2.45	(2.13-2.82)
30-31	2.29	(2.07-2.53)	2.48	(2.15-2.86)	1.92	(1.50-2.46)	2.17	(1.91-2.46)
32-34	1.72	(1.64-1.81)	1.71	(1.59-1.85)	1.56	(1.35-1.79)	1.65	(1.56-1.74)
35	1.57	(1.49-1.65)	1.55	(1.44-1.67)	1.5	(1.36-1.67)	1.53	(1.44-1.63)
36	1.44	(1.39-1.49)	1.43	(1.35-1.51)	1.41	(1.33-1.51)	1.4	(1.35-1.46)
37	1.31	(1.28-1.34)	1.29	(1.25-1.34)	1.29	(1.25-1.34)	1.32	(1.28-1.36)
38	1.15	(1.13-1.17)	1.14	(1.12-1.17)	1.14	(1.12-1.16)	1.15	(1.12-1.17)
39-40 (ref)	1.00	--	1.00	--	1.00	--	1.00	--
41	0.94	(0.92-0.96)	0.89	(0.86-0.92)	0.96	(0.94-0.98)	0.95	(0.93-0.97)
≥42	0.99	(0.94-1.04)	0.88	(0.78-0.98)	1	(0.95-1.06)	0.97	(0.92-1.03)
**Birthweight (g)**								
≤1000	2.51	(2.21-2.85)	2.8	(2.32-3.38)	1.83	(1.19-2.79)	2.55	(2.15-3.02)
>1000-1500	2.30	(2.10-2.51)	2.37	(2.06-2.73)	2.35	(1.84-3.01)	2.25	(2.03-2.49)
>1500-2000	1.82	(1.71-1.94)	1.78	(1.62-1.96)	1.75	(1.44-2.13)	1.73	(1.59-1.88)
>2000-2500	1.42	(1.37-1.47)	1.27	(1.20-1.35)	1.42	(1.33-1.52)	1.41	(1.35-1.47)
>2500-3000	1.14	(1.12-1.16)	1.06	(1.03-1.09)	1.11	(1.09-1.14)	1.13	(1.10-1.15)
>3000-3500 (ref)	1.00	--	1.00	--	1.00	--	1.00	--
>3500-4000	0.94	(0.93-0.95)	0.95	(0.93-0.97)	0.95	(0.94-0.97)	0.94	(0.92-0.95)
>4000-4500	0.90	(0.88-0.92)	0.91	(0.88-0.94)	0.93	(0.9-0.95)	0.9	(0.88-0.92)
>4500	0.93	(0.89-0.98)	0.95	(0.88-1.03)	0.96	(0.9-1.01)	0.95	(0.90-1.00)
**Birth Length (cm)**								
≤30	2.38	(1.85 - 3.07)	3.23	(2.06-5.06)	1.51	(0.79-2.88)	2.45	(1.72-3.50)
>30-35	2.17	(1.91 - 2.47)	2.72	(2.21-3.35)	1.17	(0.85-1.61)	2.11	(1.78-2.50)
>35-40	2.11	(1.93 - 2.29)	2.14	(1.87-2.45)	1.75	(1.40-2.20)	1.99	(1.80-2.20)
>40-45	1.45	(1.40 - 1.50)	1.39	(1.31-1.46)	1.31	(1.24-1.39)	1.4	(1.34-1.46)
>45-50 (ref)	1.00	--	1.00	--	1.00	--	1.00	--
>50-55	0.89	(0.88 - 0.91)	0.92	(0.91-0.94)	0.92	(0.91-0.93)	0.9	(0.89-0.91)
>55-60	0.88	(0.84 - 0.92)	0.9	(0.83-0.96)	0.92	(0.87-0.97)	0.88	(0.83-0.93)
>60-65	1.09	(0.70 - 1.70)	1.08	(0.68-1.72)	1.1	(0.61-1.96)	1.38	(0.86-2.21)

Adjusted for maternal age (<20,20-24,25-29,30-24, >=35 years), parity (no previous birth/yes previous birth), birth cohort (2 year blocks), season of birth, sex, mode of delivery (vaginal/caesarean), 5 minute APGAR score (0-7,8-10), bronchopulmonary dysplasia (present/absent), socioeconomic status (percentile: >90 (highest), 75-90, 50-75, 25-50, 10-25, <10).

'No neurodevelopmental disability' group was defined by children without a diagnosed intellectual disability, severe intellectual disability of unknown aetiology, birth defects, cerebral palsy, and whose mothers reported no smoking during pregnancy (where data were available).

'No complications during pregnancy' group was defined by children whose mothers had no recorded complications for the variable 'complications during pregnancy' in the Midwives' Notification System

'No medical conditions during pregnancy' group was defined by children whose mothers had no recorded medical conditions for the variable 'medical conditions during pregnancy' in the Midwives' Notification System

Supplementary Table: Overall adjusted childhood infection-related hospitalisation rate ratio and 95% confidence interval with and without adjustments for socioeconomic status and smoking during pregnancy in model.

	Fully adjusted		Fully adjusted + smoking status		Socioeconomic status not included in model	
**Gestational Age (wks)**	HR	95% CI	HR	95% CI	RR	95%CI
<28	2.91	(2.55-3.33)	2.96	(2.53-3.47)	2.93	(2.60-3.30)
28-29	2.49	(2.24-2.78)	2.82	(2.44-3.25)	2.47	(2.25-2.71)
30-31	2.29	(2.07-2.53)	2.68	(2.35-3.04)	2.24	(2.06-2.45)
32-34	1.72	(1.64-1.81)	1.89	(1.77-2.02)	1.71	(1.64-1.79)
35	1.57	(1.49-1.65)	1.7	(1.58-1.84)	1.55	(1.48-1.63)
36	1.44	(1.39-1.49)	1.53	(1.45-1.61)	1.41	(1.37-1.46)
37	1.31	(1.28-1.34)	1.35	(1.30-1.40)	1.3	(1.27-1.33)
38	1.15	(1.13-1.17)	1.17	(1.15-1.20)	1.14	(1.12-1.15)
39-40 (ref)	1.00	--	1.00	--	1.00	--
41	0.94	(0.92-0.96)	0.90	(0.88-0.93)	0.94	(0.93-0.96)
≥42	0.99	(0.94-1.04)	0.91	(0.82-1.01)	1.02	(0.99-1.06)
**Birthweight (g)**						
≤1000	2.51	(2.21-2.85)	2.63	(2.26-3.07)	2.61	(2.32-2.94)
>1000-1500	2.30	(2.10-2.51)	2.51	(2.23-2.81)	2.3	(2.12-2.49)
>1500-2000	1.82	(1.71-1.94)	2.01	(1.84-2.19)	1.87	(1.77-1.98)
>2000-2500	1.42	(1.37-1.47)	1.42	(1.35-1.50)	1.43	(1.39-1.48)
>2500-3000	1.14	(1.12-1.16)	1.12	(1.09-1.15)	1.14	(1.12-1.16)
>3000-3500 (ref)	1.00	--	1.00	--	1.00	--
>3500-4000	0.94	(0.93-0.95)	0.93	(0.91-0.95)	0.94	(0.93-0.95)
>4000-4500	0.90	(0.88-0.92)	0.90	(0.87-0.93)	0.90	(0.89-0.92)
>4500	0.93	(0.89-0.98)	0.91	(0.84-0.97)	0.93	(0.89-0.97)
**Birth Length (cm)**						
≤30	2.38	(1.85 - 3.07)	2.52	(1.86-3.42)	2.51	(1.95-3.21)
>30-35	2.17	(1.91 - 2.47)	2.64	(2.24-3.11)	2.17	(1.94-2.43)
>35-40	2.11	(1.93 - 2.29)	2.25	(2.02-2.50)	2.11	(1.95-2.28)
>40-45	1.45	(1.40 - 1.50)	1.45	(1.39-1.52)	1.46	(1.42-1.51)
>45-50 (ref)	1.00	--	1.00	--	1.00	--
>50-55	0.89	(0.88 - 0.91)	0.89	(0.88-0.91)	0.90	(0.89-0.91)
>55-60	0.88	(0.84 - 0.92)	0.88	(0.82-0.95)	0.90	(0.87-0.94)
>60-65	1.09	(0.70 - 1.70)	0.96	(0.61-1.49)	1.04	(0.75-1.44)

*Fully adjusted model includes maternal age (<20,20-24,25-29,30-24, >=35 years), parity (no previous birth/yes previous birth), birth cohort (2 year blocks), season of birth, sex, mode of delivery (vaginal/caesarean), 5 minute APGAR score (0-7,8-10), bronchopulmonary dysplasia (present/absent), socioeconomic status (percentile: >90 (highest), 75-90, 50-75, 25-50, 10-25, <10).

Supplementary Figure

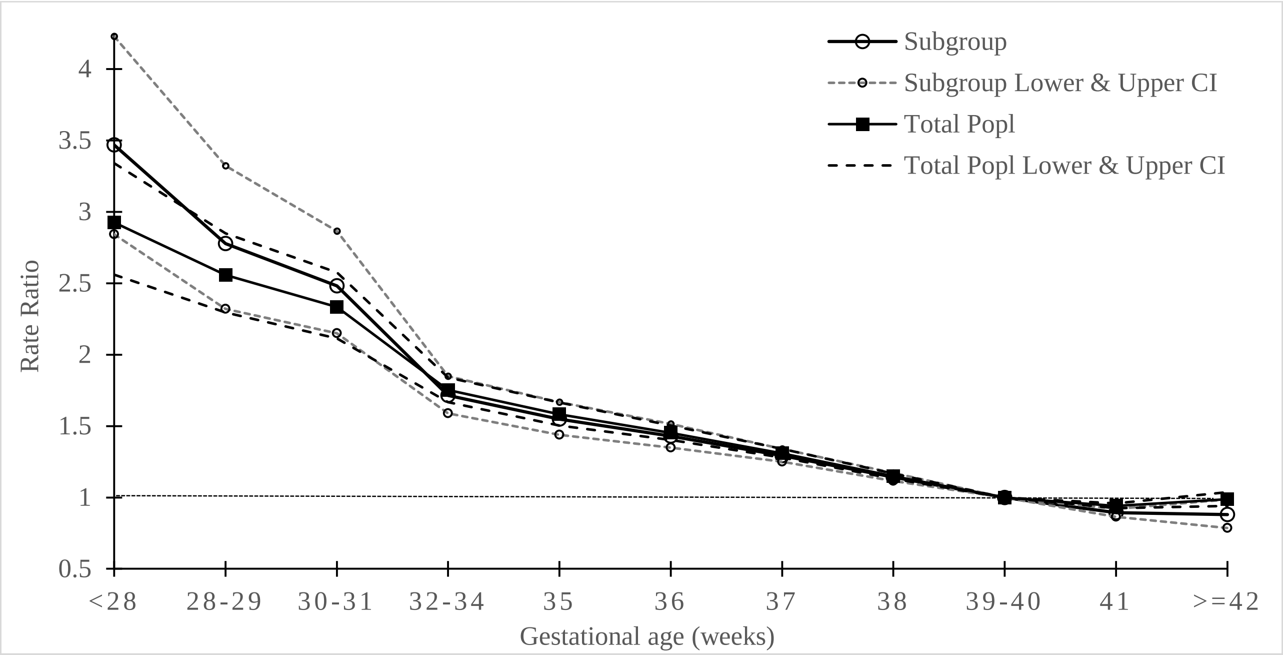

Figure adjusted for maternal age (<20,20-24,25-29,30-24, >=35 years), parity (no previous birth/yes previous birth), birth cohort (2 year blocks), season of birth, sex, mode of delivery (vaginal/caesarean), 5 minute APGAR score (0-7,8-10), bronchopulmonary dysplasia (present/absent), socioeconomic status (percentile: >90 (highest), 75-90, 50-75, 25-50, 10-25, <10).

Supplementary Figure

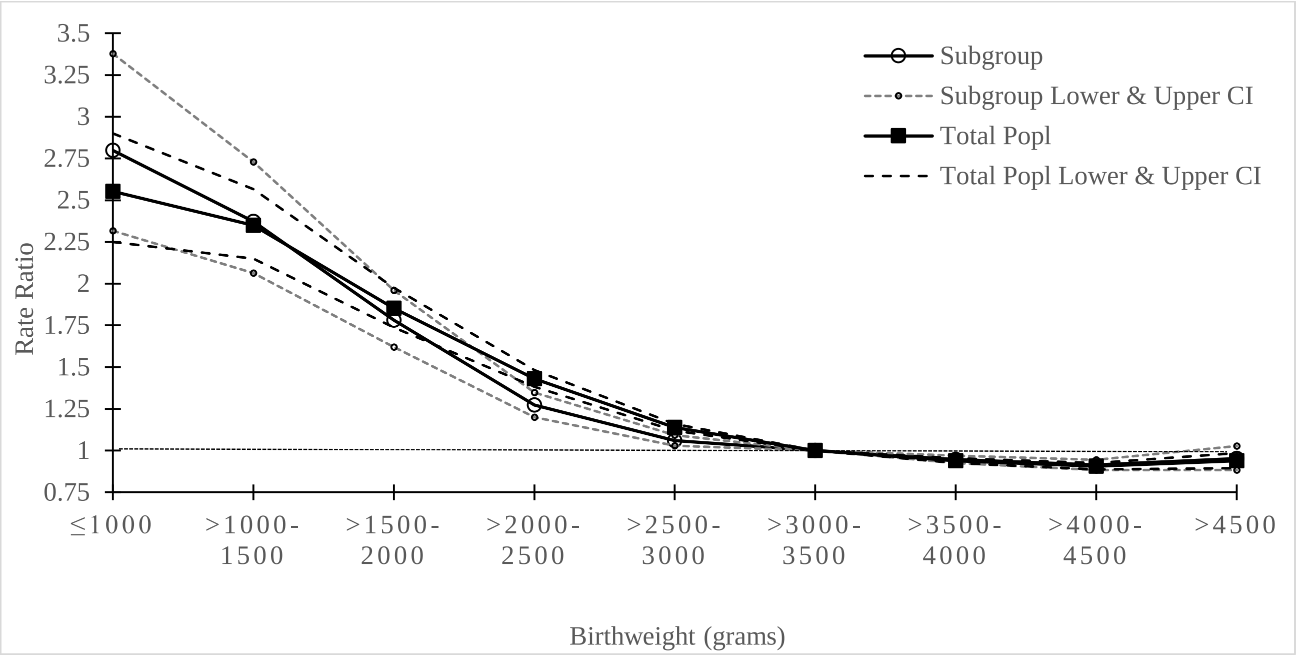

Figure adjusted for maternal age (<20,20-24,25-29,30-24, >=35 years), parity (no previous birth/yes previous birth), birth cohort (2 year blocks), season of birth, sex, mode of delivery (vaginal/caesarean), 5 minute APGAR score (0-7,8-10), bronchopulmonary dysplasia (present/absent), socioeconomic status (percentile: >90 (highest), 75-90, 50-75, 25-50, 10-25, <10).

Supplementary Figure

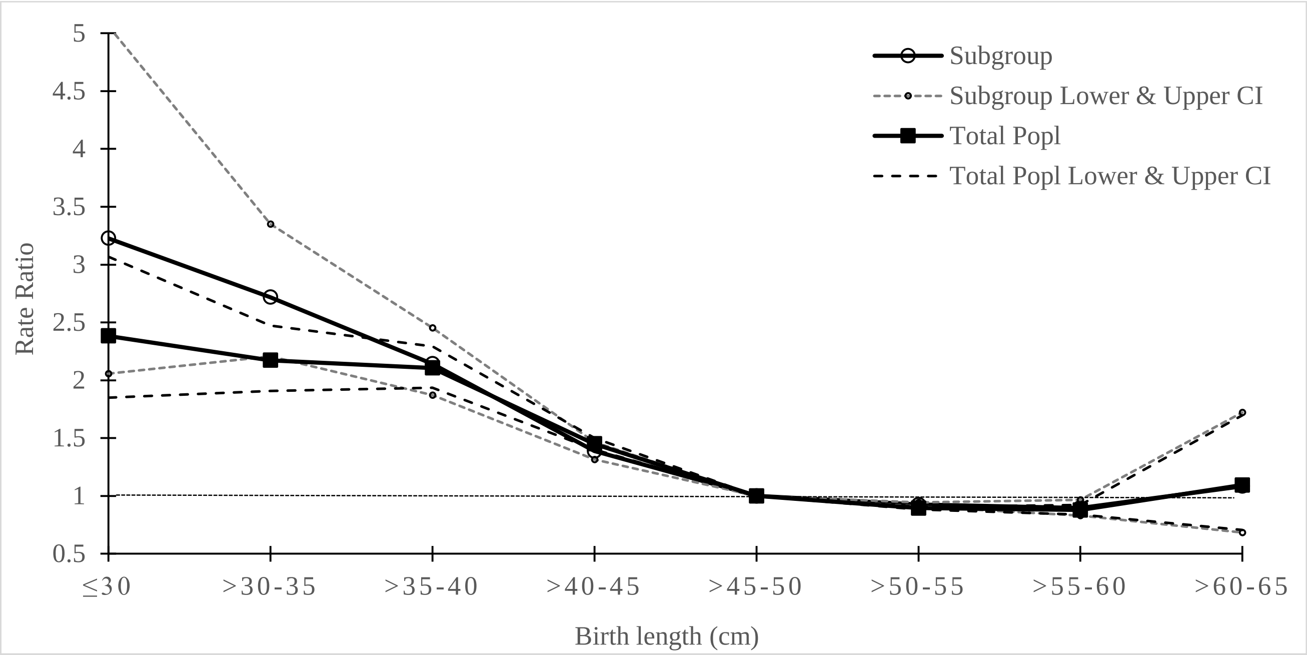

Figure adjusted for maternal age (<20,20-24,25-29,30-24, >=35 years), parity (no previous birth/yes previous birth), birth cohort (2 year blocks), season of birth, sex, mode of delivery (vaginal/caesarean), 5 minute APGAR score (0-7,8-10), bronchopulmonary dysplasia (present/absent), socioeconomic status (percentile: >90 (highest), 75-90, 50-75, 25-50, 10-25, <10).

Supplementary Figure

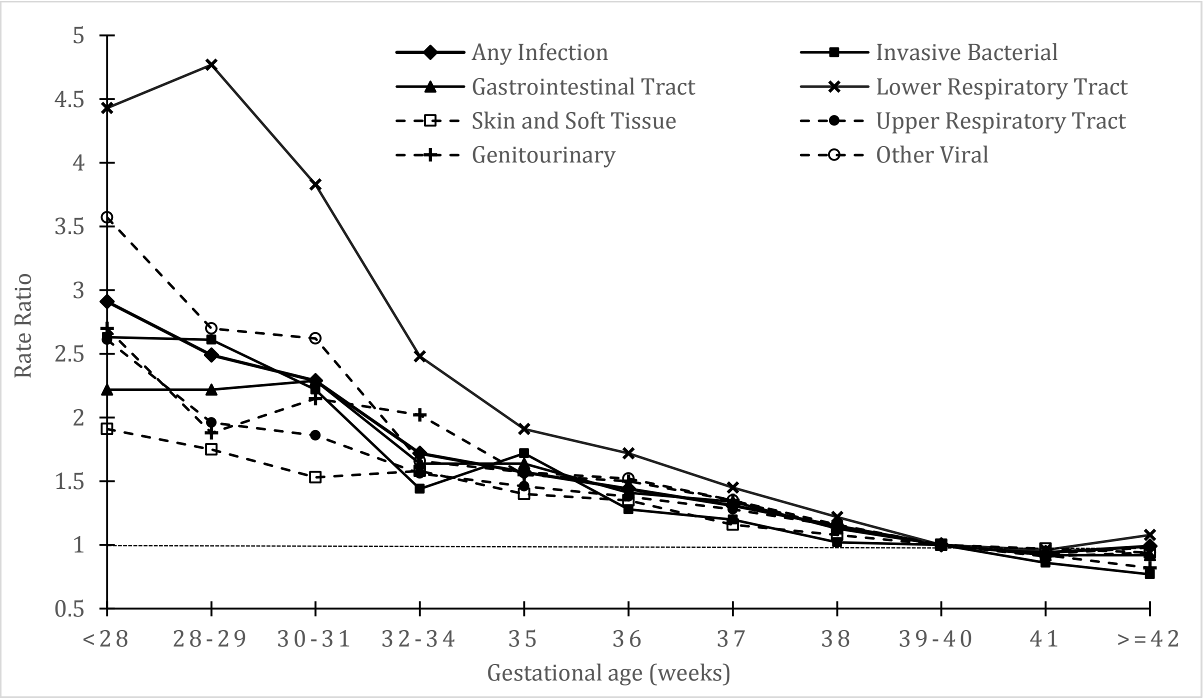

Figure adjusted for maternal age (<20, 20-24, 25-29, 30-34, >=35 years), parity (no previous birth/yes previous birth), birth cohort (2 year blocks), season of birth, sex, mode of delivery (vaginal/caesarean), 5 minute APGAR score (0-7,8-10), bronchopulmonary dysplasia (present/absent), socioeconomic status (percentile: >90 (highest), 75-90, 50-75, 25-50, 10-25, <10).

Supplementary Figure

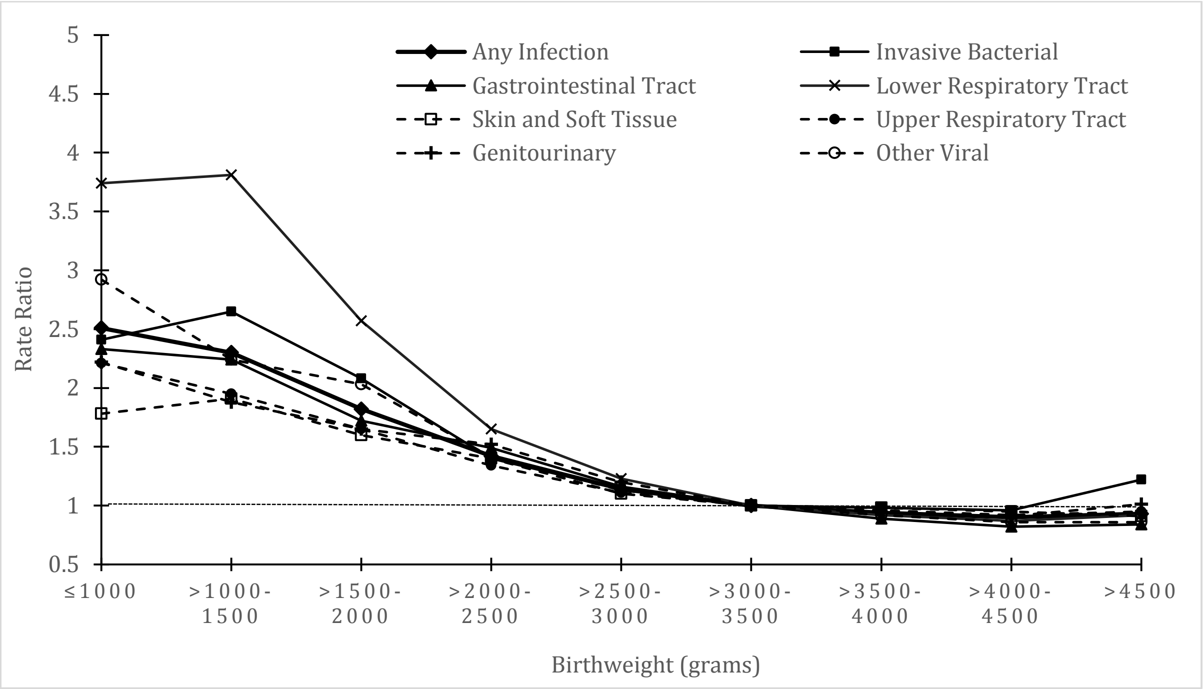

Figure adjusted for maternal age (<20, 20-24, 25-29, 30-34, >=35 years), parity (no previous birth/yes previous birth), birth cohort (2 year blocks), season of birth, sex, mode of delivery (vaginal/caesarean), 5 minute APGAR score (0-7,8-10), bronchopulmonary dysplasia (present/absent), socioeconomic status (percentile: >90 (highest), 75-90, 50-75, 25-50, 10-25, <10).

Supplementary Figure

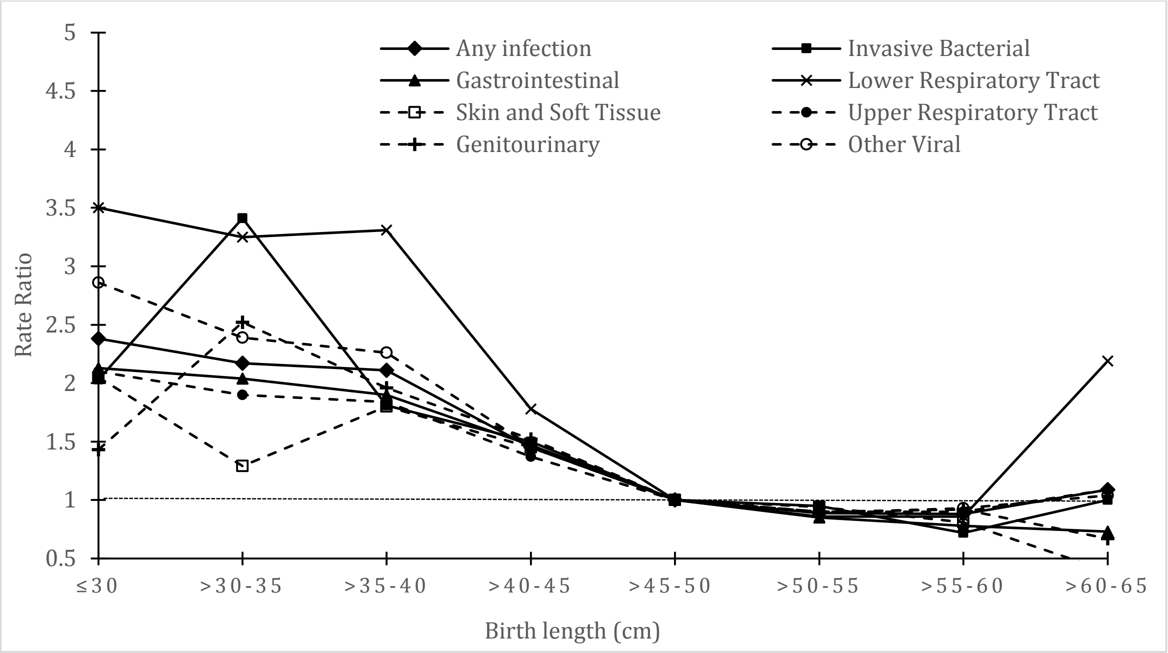

Figure adjusted for maternal age (<20, 20-24, 25-29, 30-34, >=35 years), parity (no previous birth/yes previous birth), birth cohort (2 year blocks), season of birth, sex, mode of delivery (vaginal/caesarean), 5 minute APGAR score (0-7,8-10), bronchopulmonary dysplasia (present/absent), socioeconomic status (percentile: >90 (highest), 75-90, 50-75, 25-50, 10-25, <10).

Supplementary Figure

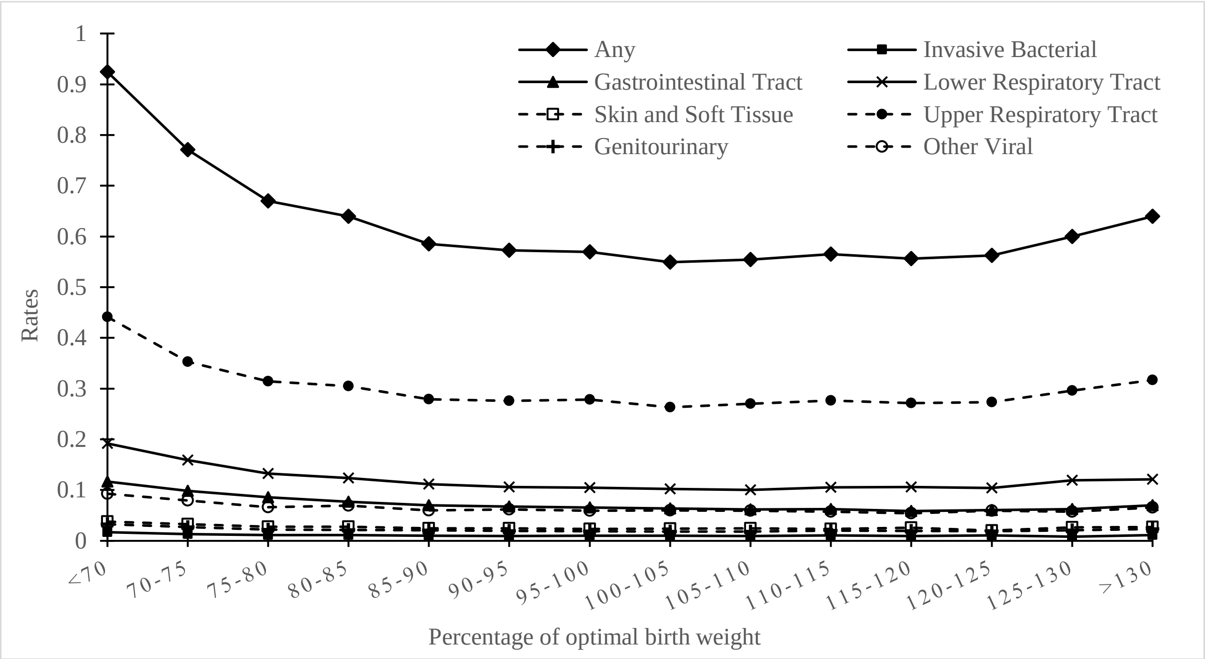

Percentage of optimal birth weight is the ratio, at each gestational age, of an individual's birth weight to that of the average birth weight born to a Western Australian woman of a similar height, age, and parity (based on a population cohort data from 1998-2002), excluding women with morbidities commonly affecting intrauterine growth (smoking, cardiovascular disease, birth defects, diabetes, congenital infections, and multiple pregnancies).20 A percentage of optimal birth weight value of <100 indicates that the baby was lighter than expected, whereas a value >100 indicates a baby heavier than expected, based on gestational age and mothers' characteristics.

Adjusted for maternal age (<20, 20-24, 25-29, 30-34, >=35 years), parity (no previous birth/yes previous birth), birth cohort (2 year blocks), season of birth, sex, mode of delivery (vaginal/caesarean), 5 minute APGAR score (0-7,8-10), bronchopulmonary dysplasia (present/absent), socioeconomic status (percentile: >90 (highest), 75-90, 50-75, 25-50, 10-25, <10).
